# MiRNA/mRNA network topology in hepatitis virus B-related liver cirrhosis reveals miR-20a-5p/340-5p as hubs initiating fibrosis

**DOI:** 10.1186/s12920-022-01390-x

**Published:** 2022-11-14

**Authors:** Heng Yao, Peng Li, Jiaojiao Xin, Xi Liang, Jing Jiang, Dongyan Shi, Jiang Li, Hozeifa Mohamed Hassan, Xin Chen, Jun Li

**Affiliations:** 1 State Key Laboratory for Diagnosis and Treatment of Infectious Diseases, National Medical Center for Infectious Diseases, National Clinical Research Center for Infectious Diseases, Collaborative Innovation Center for Diagnosis and Treatment of Infectious Diseases, The First Affiliated Hospital, Zhejiang University School of Medicine, 79 Qingchun Rd., Hangzhou, 310003 China; 2 Precision Medicine Center, Taizhou Central Hospital (Taizhou University Hospital), Taizhou, China; 3 Department of Infectious Disease, The First Affiliated Hospital of Anhui Medical University, Hefei, China; 4 Institute of Pharmaceutical Biotechnology and the First Affiliated Hospital Department of Radiation Oncology, Zhejiang University School of Medicine, Hangzhou, China; 5 Joint Institute for Genetics and Genome Medicine between Zhejiang University and University of Toronto, Zhejiang University, Hangzhou, China

**Keywords:** Liver cirrhosis, Hepatic fibrogenesis, Hepatitis B, miRNA-mRNA network, Topological analysis, Multiomics

## Abstract

**Background:**

The pathophysiology of hepatitis B-related liver cirrhosis (HBV-LC) remains unclear. This study aimed to explore the disease mechanisms using topological analysis of the miRNA/mRNA network.

**Methods:**

Paired miRNA/mRNA sequencing was performed with thirty-three peripheral blood mononuclear cell samples (LC, n = 9; chronic hepatitis B, n = 12; normal controls, n = 12) collected from a prospective cohort to identify the miRNA/mRNA network. Topological features and functional implications of the network were analyzed to capture pathophysiologically important miRNAs/mRNAs, whose expression patterns were confirmed in the validation group (LC, n = 15; chronic hepatitis B, n = 15; normal controls, n = 10), and functional potentials initiating fibrogenesis were demonstrated in vitro.

**Results:**

The miRNA/mRNA network contained 3121 interactions between 158 differentially expressed (DE) miRNAs and 442 DE-mRNAs. The topological analysis identified a core module containing 99 miRNA/mRNA interactions and two hub nodes (miR-20a-5p/miR-340-5p), which connected to 75 DE-mRNAs. The expression pattern along the disease progression of the core module was found associated with a continuous increase in wound healing, inflammation, and leukocyte migration but an inflection of immune response and lipid metabolic regulation, consistent with the pathophysiology of HBV-LC. MiR-20a-5p/miR-340-5p were found involved in macrophage polarization and hepatic stellate cell (HSC) activation in vitro (THP-1, LX-2 cell lines), and their expression levels were confirmed in the validation group independently.

**Conclusion:**

Topological analysis of the miRNA/mRNA network in HBV-LC revealed the association between fibrosis and miR-20a-5p/miR-340-5p involving initiating activations of macrophage and HSC. Further validations should be performed to confirm the HSC/macrophage activations and the interactions between miR-20a-5p/miR-340-5p and their potential targets, which may help to develop non-invasive prognostic markers or intervention targets for HBV-LC.

**Supplementary Information:**

The online version contains supplementary material available at 10.1186/s12920-022-01390-x.

## Introduction

Hepatitis B virus (HBV) infection is a global public health problem, and liver cirrhosis (LC) is a major sequela of chronic HBV infection [1, 2], in which current antiviral therapies cannot eradicate HBV, leading to persistent liver inflammation, developmental fibrogenesis and irreversible fibrosis [3, 4]. Recent studies have reported that hepatic fibrogenesis is a complex pathophysiological process involving several biological pathways including inflammation, tissue remodeling, viral/immune response and metabolic regulation [5–7], in which microRNAs (miRNAs) are regulatory hubs bridging and coordinating these pathways [8–10].

However, the molecular basis of the miRNA/mRNA regulatory network remains poorly understood in the context of clinical data from HBV-LC patients. The functional polymorphism [11–13] and dynamic feedback relationships [14, 15] of the miRNA/mRNA pairs forge the complexity of the regulatory network, in which traditional linear analytic strategies may fail to produce a unbiased systematic understanding of the miRNAs’ contributions in the disease progression. Not only in HBV-LC research, but also in other disease studies, deciphering the complexity of miRNA/mRNA regulatory networks is considered as an efficient analysis method to integrated miRNA and mRNA transcriptome with a biological meaningful network-based model instead of select candidate molecular for further validation by arbitrary cutoffs (e.g., isolated expression levels). Also, the network model of miRNA/mRNA fit well with the natural regulatory pattern of miRNA, which give the network-based analysis advantage in interpreting its discoveries [16–18]. Additionally, the lack of data from tissue samples of chronic patients of HBV-LC remains a problem because of the low utilization rate of liver biopsy [19–21] due to its high-risk of complications [22, 23], leading to a urgent need to develop analytic strategies that are able to take advantage of abundant data from non-invasive clinical samples.

Therefore, in this study, we presented a novel analytic strategy to solve the problems addressed above by using high-throughput real-world data, describing the miRNA/mRNA transcriptome based on network construction and applying topological analysis to identify the miRNA/mRNA network hubs. This analytic strategy allowed us to capture the essential of miRNA/mRNA regulation in HBV-LC progression since it integrated miRNA and mRNA transcriptome to form miRNA/mRNA pair as basic analysis element instead of separated miRNA or mRNA molecular selected by linear transcriptional evidence. Focusing on miRNA/mRNA pair, we can conduct the topological analysis to assesses the influence power of each miRNA/mRNA pair in the context of the entire miRNA/mRNA network to avoid observing miRNA or mRNA isolated based on separated transcriptional signals. Taken together, applying this network-based topological analysis, we revealed the association between miR-340-5p/miR-20a-5p module and the fibrosis processes and explored its potential regulatory patterns in vitro.

## Materials and methods

### Study design and sequencing data collection

**Fig. 1:**
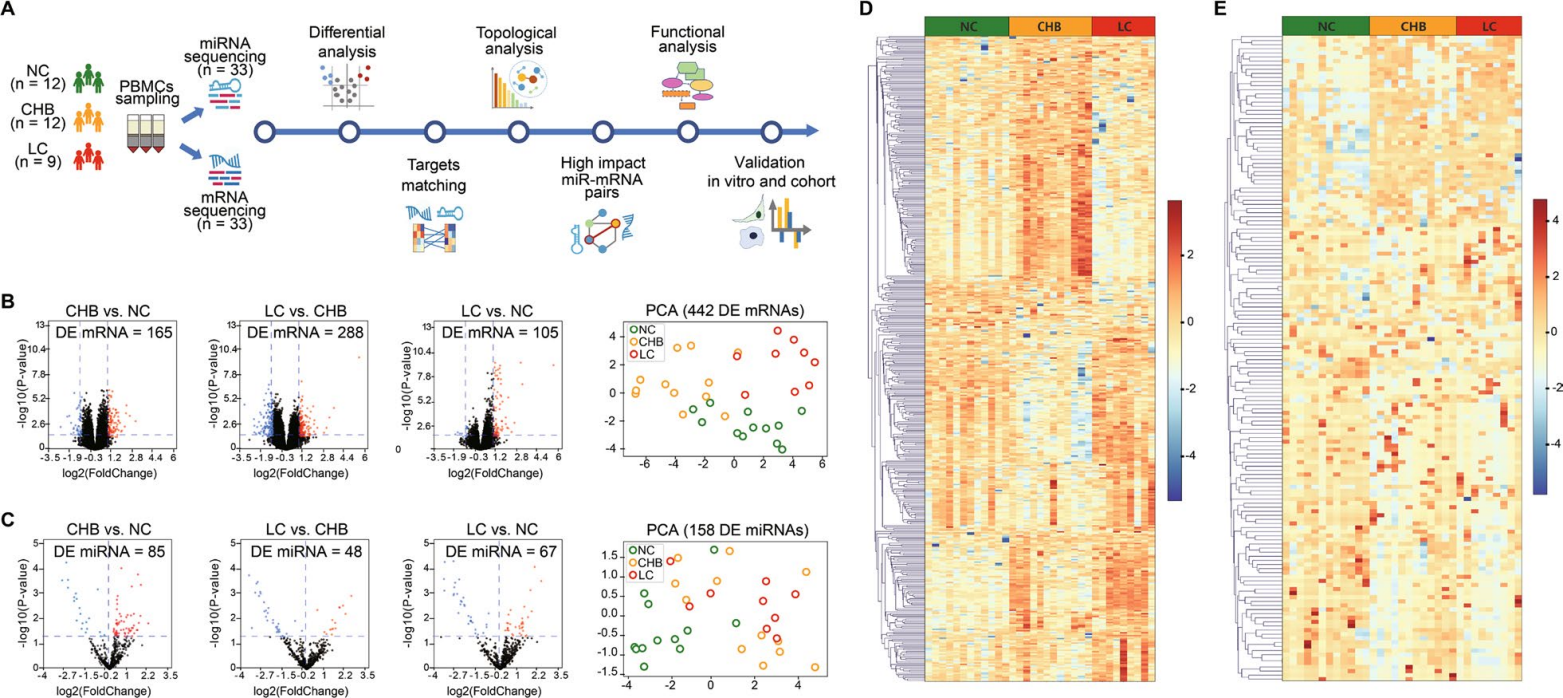
Transcriptomic characteristics of patients with HBV-LC. **A** Study design and flow chart of the network-based analysis strategy. **B** Volcano and PCA plots of the DE mRNAs (log_2_ |FC|≥1, padj ≤ 0.05) in the three pairwise comparison groups. **C** Volcano and PCA plots of the DE miRNAs (*p* ≤ 0.05) in the three pairwise comparison groups. **D** Heatmap showing the expression levels of the 442 mRNAs in the three pairwise comparison groups of PBMCs. **E** Heatmap showing the expression levels of the 158 mRNAs in the three pairwise comparison groups of PBMCs. Numbers of paired miRNA/mRNA sequencing samples (n = 9, 12, and 12 in the LC, CHB, and NC groups, respectively). *CHB* Chronic hepatitis B; *DE* Differentially expressed; *LC* Liver cirrhosis; *NC* Normal control; *PBMC* Peripheral blood mononuclear cell

Thirty-three subjects were selected from a multicenter, prospective cohort called Chinese group on the Study of Severe Hepatitis B (COSSH) [24]. The inclusion and exclusion criteria for patients were consistent with the COSSH. The subjects in derivation group were in one of three clinical groups: LC (n = 9), Chronic hepatitis B (CHB) (n = 12) and NC (n = 12) and the subjects in validation group were in one of three clinical groups: LC (n = 15), CHB (n = 15) and NC (n = 10). PBMC samples were collected from all subjects for both miRNA and mRNA sequencing to obtain paired miRNA and mRNA transcriptomic data for subsequent analyses (Fig. 1A). The details of the data collection and applied RNA sequencing processing are provided in the Additional file 1.

### Identification of the miRNA/mRNA network

The pairing scores for all DE miRNA and DE mRNA pairs were calculated based on both experimental evidence from miRBase [25] and predictive models from TargetScan [26] and microTCDs [27]. Subsequently, a weighted unidirectional scale-free miRNA/mRNA network was identified to include all potential pairs between DE miRNAs and mRNAs. Details of identification of miRNA/mRNA network are provided in the Additional file 1.

### Tissue atlas screening

Tissue atlas screening was performed to identify miRNA/mRNA pairs containing miRNAs with high expression abundance in human liver tissues, utilizing the human miRNA tissue atlas database [28], which was developed based on the miRNA microarray data of human tissue samples. Based on the miRNA distributions across different tissues, the miRNAs expressed in human liver tissues were selected to filter the potential miRNA/mRNA pairs. The detailed tissue atlas screening method is provided in the Additional file 1.

### Topological analysis of the miRNA/mRNA network

Eigenvector centrality was used to characterize the network centrality of nodes in the miRNA/mRNA network, which represents the influence of a node in the given network [29, 30]. The network centralities of both nodes in a miRNA/mRNA pair were used to calculate the topological strength of the pair. The calculation of eigenvector centrality was done with Cytoscape3.1 [31]. The detailed calculation methods of the eigenvector centrality and the topological strength are provided in the Additional file 1.

### Functional and pathway analysis

The functional analysis of core module and miRNA transfection induced differences in vitro were conducted with the gene set enrichment analysis function in R package called clusterProfiler [32]. The pathway analysis of miR-340-5p and miR-20a-5p was conducted by using gene set linkage analysis (GSLA) [33]. The biological pathway data applied in GSLA was retrieved from the Reactome database [34]. Details of the functional synergy analysis are provided in the Additional file 1.

### Statistical analysis

Principal component analysis (PCA) (Python, sklearn package) was performed with gene expression read count data. Differential expression analyses were performed by using the standard pipeline of the R package DESeq2 [35]. The multiple t-test was used in DEAs, and p-values were adjusted for multiple comparisons based on the Benjamini-Hochberg procedure. Detailed statistical methods are provided in the Additional file 1.

### Cell culture and treatment of miRNA mimics and inhibitors

The immortalized human HSC line LX-2 and human monocytic cell line THP-1 were cultured following the manufacturer’s (Cobioer Biosciences Co., Ltd. (Nanjing, China)) protocol. The mimics transfection of miR-340-5p and miR-20a-5p in LX-2 and THP-1 were performed using Hieff TransTM in vitro siRNA/miRNA Transfection Reagent (Yeasen Biotech Co. Ltd. (Shanghai, China)). The introduction of the miRNA inhibitors (40 nM, Thermo Fisher Scientific, Inc., Waltham, MA, USA) was performed following the manufacturer’s protocol. Details of cell culture, treatments of the miRNA mimics and inhibitors are provided in the Additional file 1.

### Determination of miRNA expression levels

QRT-PCR validation was performed to confirm the results of the expression levels of miRNA after transfection. Total RNA was extracted from LX-2 cells and THP-1 cells using TRIzol reagent (Invitrogen, Inc., Waltham, MA, USA). Then the RNA was applied for reverse transcription and quantitative real-time PCR using TaqMan Small RNA Assay kit (Thermo Fisher Scientific, Inc., Waltham, MA, USA) according to the manufacturer’s user guide on the ABI 7500 real time PCR system (Applied Biosystems, Foster City, CA). The fold changes of genes were calculated using 2^−ΔΔCt^ method. The miRNA expression was normalized to RNU6B. All primers were purchased from Applied Biosystems (Foster City, CA). More details are provided in the Additional file 1.

### Patient and public involvement

None.

## Results

### Identification of disease-related DE mRNAs and miRNAs

The clinical characteristics of all thirty-three study subjects are provided in Table 1. mRNA and miRNA expression levels in the study subjects were characterized using mRNA-Seq and small RNA-Seq, respectively (Fig. 1A) and a total of 14,938 mRNAs and 642 miRNAs were identified. Differential expression analyses (DEAs) were performed to identify significantly differentially expressed mRNAs and miRNAs in pairwise comparisons between the three clinical groups (CHB vs. NC, LC vs. CHB and LC vs. NC). In total, 442 DE mRNAs were identified (165 DE mRNAs in CHB vs. NC, 288 DE mRNAs in LC vs. CHB, 105 DE mRNAs in LC vs. NC, Fig. 1B, D, Additional file 2: Tables S1, S2, S3, S7); 158 DE miRNAs were identified (85 DE miRNAs in CHB vs. NC, 48 DE miRNAs in LC vs. CHB, 67 DE miRNAs in LC vs. NC, Fig. 1C, E, Additional file 2: Tables S4, S5, S6, S8).

**Table 1:** Clinical characteristics of the patients in the RNA sequencing cohort

Characteristic	LC (n = 9)	CHB (n = 12)	NC (n = 12)
Age (years), median [Q1, Q3]	49.0 [46.0, 53.0]	43.5 [37.8, 53.0]	49.0 [31.0, 50.8]
Female (%)	1 (11.1)	3 (25.0)	2 (16.7)
Male (%)	8 (88.9)	9 (75.0)	10 (83.3)
*HBV DNA level (IU/ml)*			
≤ 2 × 10^2	9	11	–
2 × 10^2–2 × 10^6	0	1	–
> 2 × 10^6	0	0	–
Antiviral therapy (%)	9 (100)	11 (91.7)	–
*Laboratory data, mean (SD)*			
Albumin (g/L)	46.6 (3.1)	49.7 (2.4)	48.8 (2.5)
Globulin (g/L)	26.6 (2.1)	27.3 (1.6)	25.6 (3.3)
Total bilirubin (µmol/L)	15.4 (4.2)**	16.2 (6.6)*	10.5 (2.1)
Direct bilirubin (µmol/L)	5.8 (1.9)**	5.8 (2.3)**	3.3 (0.7)
Indirect bilirubin (µmol/L)	9.7 (2.8)*	10.4 (4.8)*	7.2 (1.7)
Alanine aminotransferase (U/L)	20.1 (9.9)	31.0 (16.1)	24.3 (16.3)
Aspartate aminotransferase (U/L)	23.2 (4.9)	25.4 (7.9)	22.4 (7.4)
Alkaline phosphatase (U/L)	95.3 (42.9)	81.8 (29.4)	66.0 (24.6)
γ-Glutamyl transpeptidase (U/L)	32.4 (19.4)	27.2 (18.4)	29.6 (20.9)
Total bile acid (µmol/L)	9.0 (8.3)	5.2 (3.7)	4.6 (2.2)
Triglyceride (mmol/L)	1.3 (0.8)	1.2 (0.7)	1.5 (0.7)
Total cholesterol (mmol/L)	3.8 (0.4)**	3.9 (0.9)*	4.6 (0.5)
Creatinine (µmol/L)	71.2 (11.0)	72.8 (18.9)	80.8 (9.1)
Blood urea nitrogen (mmol/L)	6.1 (1.8)	4.8 (0.8)	4.9 (1.1)
K^+^ (mmol/L)	4.4 (0.3)	4.2 (0.3)	4.3 (0.6)
Na^+^ (mmol/L)	141.5 (2.6)	142.1 (2.4)	142.0 (2.8)
INR	1.0 (0.2)	1.0 (0.1)	–
White blood cells (*10^9/L)	4.9 (1.5)	5.5 (0.9)	6.4 (0.8)
Hemoglobin (g/L)	154.4 (10.8)	154.9 (14.1)	149.8 (16.8)
Platelets (*10^12/L)	149.2 (100.2)*	169.0 (66.8)*	238.6 (42.9)

*LC* Liver cirrhosis, *CHB* Chronic hepatitis B, *NC* Normal control

*p < 0.05, **p < 0.01

Principal component analysis showed that the three clinical groups (NC, CHB and LC) were well distinguished by the 442 DE mRNAs and 158 miRNAs (Fig. 1B, C). Thus, these RNAs were considered candidate DE miRNAs/mRNAs to represent the disease-related transcriptomic changes and were subsequently used to identify the miRNA/mRNA network.

### Topological analysis identified the core module of miRNA/mRNA network regulating fibrogenesis

The pairing scores between the 158 DE miRNAs and 442 DE mRNAs were calculated based on both experimental evidence and predictive models to identify their potential interactions, and a network containing 3121 miRNA/mRNA pairs were identified (Additional file 2: Table S9). We also performed a miRNA liver tissue atlas screening and found a subnetwork containing 2076 miRNA/mRNA pairs with high miRNA expression abundance in liver tissue (Additional file 2: Table S10). The network centralities of all nodes in these two networks were calculated to quantitatively determine the impact of each node on the entire network. A node with higher centrality has a stronger functional impact on mediating disease progression through the miRNA/mRNA network.

**Fig. 2:**
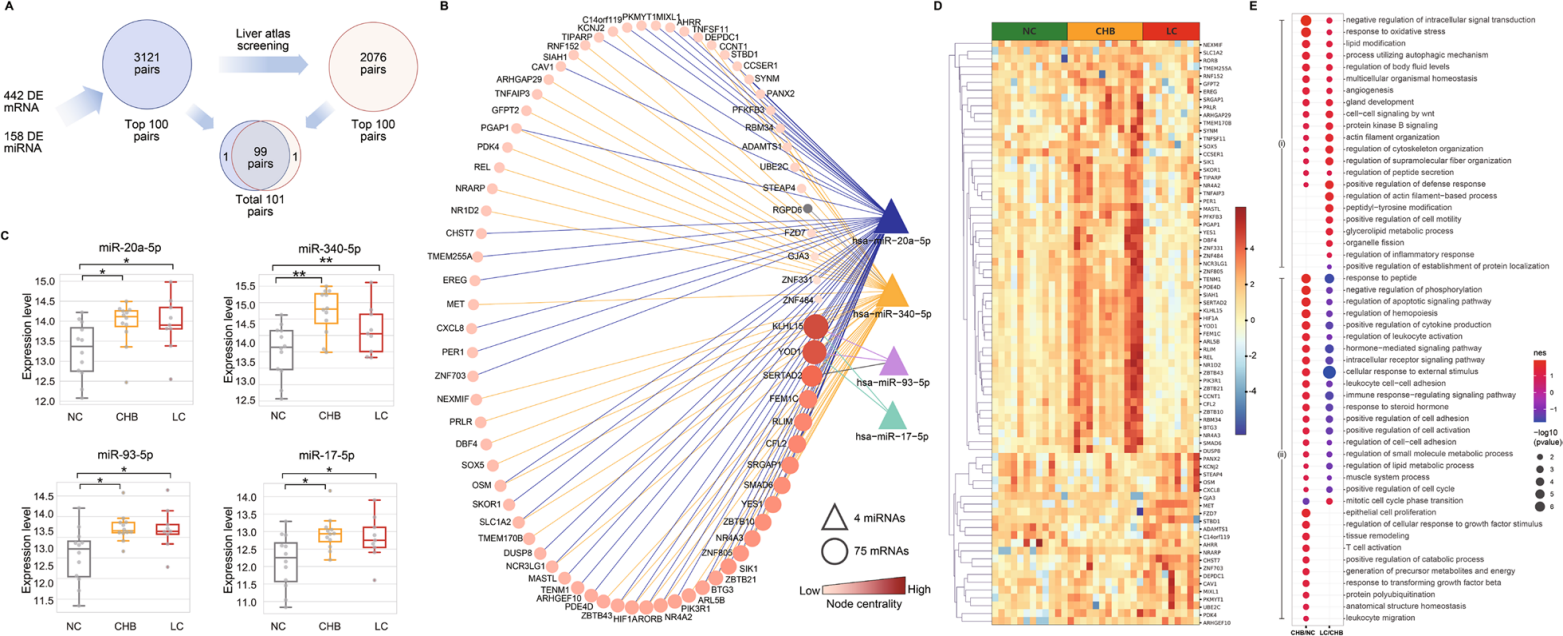
Identification and functional analysis of the core module in miRNA/mRNA network. **A** Flow chart of the identification of the miRNA/mRNA network and the core module. **B** Network layout of the core module containing four miRNAs and 75 mRNAs (the top 99 miRNA/mRNA pairs; the RGPD/miR-340-5p and SERTAD2/miR-93-5p pairs were not included, as shown in gray). **C** Expression levels of miRNA sequencing of the four miRNAs in the core module. **D** Heatmap showing the expression levels of the 75 mRNAs in the core module. **E** Dot plot showing the results of functional analysis of the core module in the comparison between different stages of the disease. Numbers of paired miRNA/mRNA sequencing samples (n = 9, 12, and 12 in the LC, CHB, and NC groups, respectively). *CHB* Chronic hepatitis B; *DE* Differentially expressed; *LC* Liver cirrhosis; *NC* Normal control; *PBMC* Peripheral blood mononuclear cell. **padj < 0.01, *padj < 0.05

Then, we filtered all the miRNA/mRNA pairs based on the result of miRNA liver tissue atlas screening and the network centralities to identify the top 100 miRNA/mRNA pairs with the highest topological strength in the global network and the liver tissue network. Most elements (99 of 100, Fig. 2A) were shared between these two groups of miRNA/mRNA pairs. This highly conserved subnetwork was identified as the core module of functional synergy to mediate disease-related pathways along the disease progression, which contained 4 miRNAs and 75 mRNAs (Fig. 2B, C, D, Additional file 2: Table S11).

The four miRNAs in the core modules were all upregulated significantly and maintained the high expression levels (Fig. 2C), which indicates their regulatory influence upon the disease-related miRNA/mRNA network surged in the initiating stage of the disease and kept the influence through the entire disease progression. The 75 mRNAs in the core module were subjected to functional analysis and the results were shown in Fig. 2E and the associated biological processes were separated into two groups. Group (i) contained associated biological processes that had a continuous increase trend along the disease progression, which reflected persistent pathophysiological progression including fibrogenesis (e.g., regulation of supramolecular fiber organization, actin filament organization, cell-cell signaling by wnt), wound healing (e.g., angiogenesis, multicellular organismal homeostasis) and inflammation (e.g., regulation of inflammatory response). Group (ii) contained associated biological processes that had an inflection point or stopped their increase in CHB stage, including cell adhesion (e.g., positive regulation of cell adhesion), immune response (e.g., positive regulation of cytokine production, immune response-regulating signaling pathway), metabolic regulation (e.g., positive regulation of catabolic process) and leukocyte migration.

### MiR-20a-5p and mir-340-5p are hub nodes in the core module regulating disease progression

**Fig. 3:**
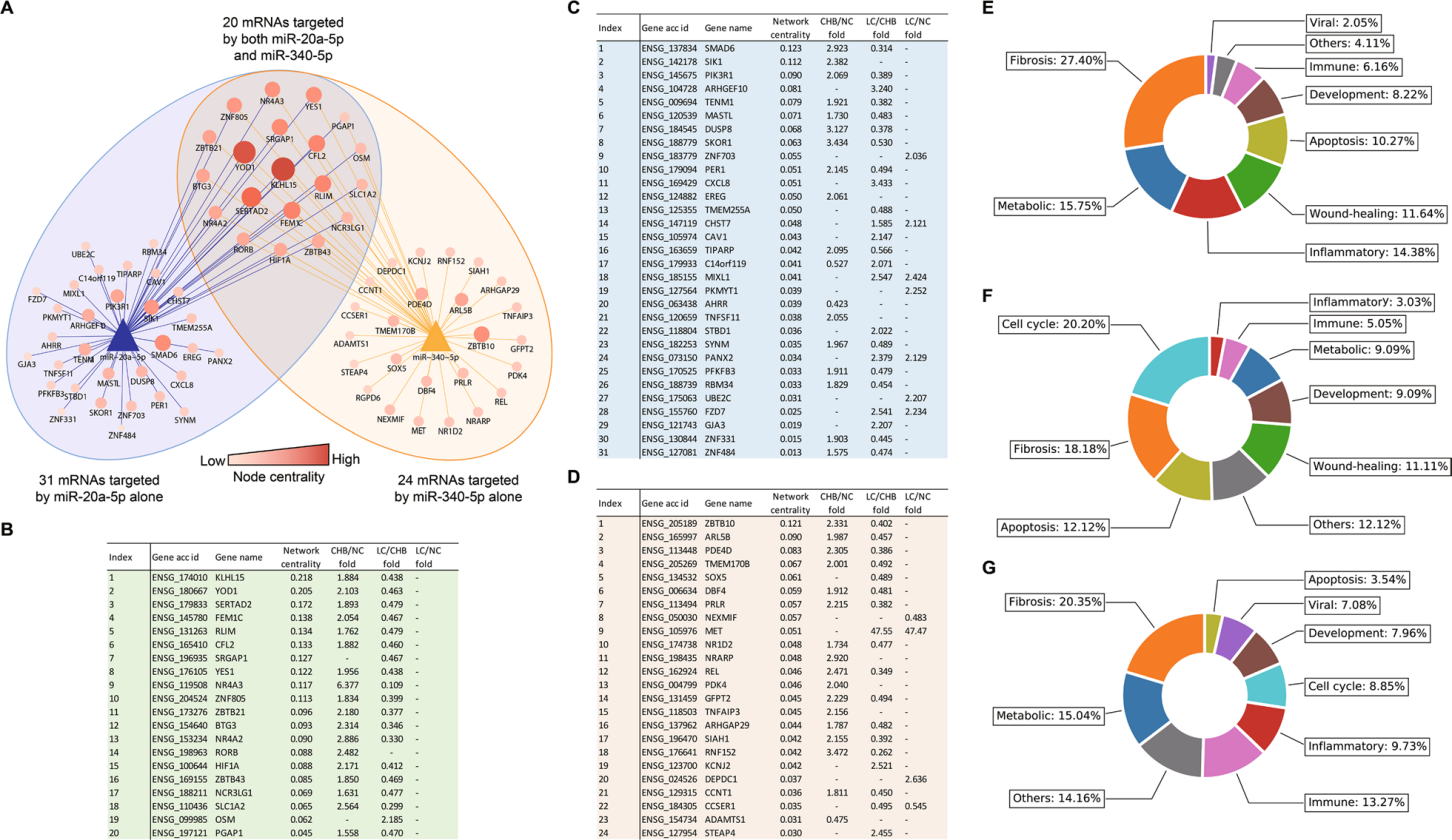
Transcriptional differences and pathway analysis of hub miRNAs. **A** Network layout of the two hub nodes (miR-20a-5p and miR-340-5p) and the 75 mRNAs in the core module. **B** Changes in the expression of the core module mRNAs connecting to miR-20a-5p in the PBMC comparison groups. **C** Changes in the expression of the core module mRNAs connecting to miR-340-5p in the PBMC comparison groups. **D** Changes in the expression of the core module mRNAs connecting to both miR-20a-5p and miR-340-5p in the PBMC comparison groups. **E**, **F**, **G** Pie charts of the magnitude of different functional categories of three groups (**E**: both connected, **F**: miR-20a-5p connected, **G**: miR-340-5p connected) of connected mRNAs in the core module. Numbers of paired miRNA/mRNA sequencing samples (n = 9, 12, and 12 in the LC, CHB, and NC groups, respectively). *CHB* Chronic hepatitis B; *DE* Differentially expressed; *LC* Liver cirrhosis; *NC* Normal control; *PBMC* Peripheral blood mononuclear cell

As shown in Fig. 2B, miRNA-20a-5p and miR-340-5p were the nodes with the 2 highest centralities in the core module. Moreover, their connecting mRNAs covered all 75 mRNAs in the core module, indicating that these two miRNAs were the hub nodes of the core module. Additionally, considering that miRNAs in the same precursor family perform similar biological functions, since miR-20a-5p, miR-93-5p, and miR-17-5p belong to the same miRNA family located in the 13q31.1 region, and miR-340-5p belongs to a different miRNA family located in the 5q35.3 region, the biological functions associated with all four miRNAs were well covered by further analysis when focusing on miRNA-20a-5p and miR-340-5p (Fig. 3A).

The seventy-five mRNAs connecting to miRNA-20a-5p and miR-340-5p were separated into three groups (20 mRNAs connecting to both hub miRNAs, 31 mRNAs connecting to only miR-20a-5p and 24 mRNAs connecting to only miR-340-5p) and subjected to pathway analysis to elucidate prominent pathophysiological pathways underlying disease progression (Fig. 3B, C, D). In total, 358 biological pathways were identified and summarized into nine functional categories related to disease progression (apoptosis, cell cycle, development, fibrosis, immune, inflammatory, metabolic, wound healing and viral; Additional file 2: Tables S12, S13, S14, S15).

As shown in the functional category plots (Fig. 3E, F, G), fibrotic pathways accounted for the highest percentage (27.4%) of the obtained biological pathways associated with the group of mRNAs connecting to both hub miRNAs, as well as for the second-highest percentage in the miR-20a-5p group (18.18%) and the and highest percentage in the miR-340-5p group (20.35%). These pathways thus represented the most prominent biological pathways underlying disease progression. After fibrotic pathways, metabolic and inflammatory pathways were the next most closely associated with the group of mRNAs connecting to both hub miRNAs, accounting for the second- and third-highest percentages (metabolic 15.75%; inflammatory 14.38%) of the obtained pathways. This pattern indicated the functional impacts these pathways had on disease progression, consistent with the known clinical manifestations of the development of LC. Also, viral, immune, and apoptotic pathways were identified, suggesting the participation of viral infection and liver damage in disease progression due to HBV reactivation and the associated immune response, while the identification of wound healing, developmental and cell cycle pathways suggested aspects of disease progression involving liver regeneration along with chronic liver injury. Based on the above observations, the core module participates in disease progression through the hub miRNAs, while fibrotic, inflammatory, and metabolic pathways involved, accompanied by the pathways of viral infection, immune response, and wound healing.

**Fig. 4:**
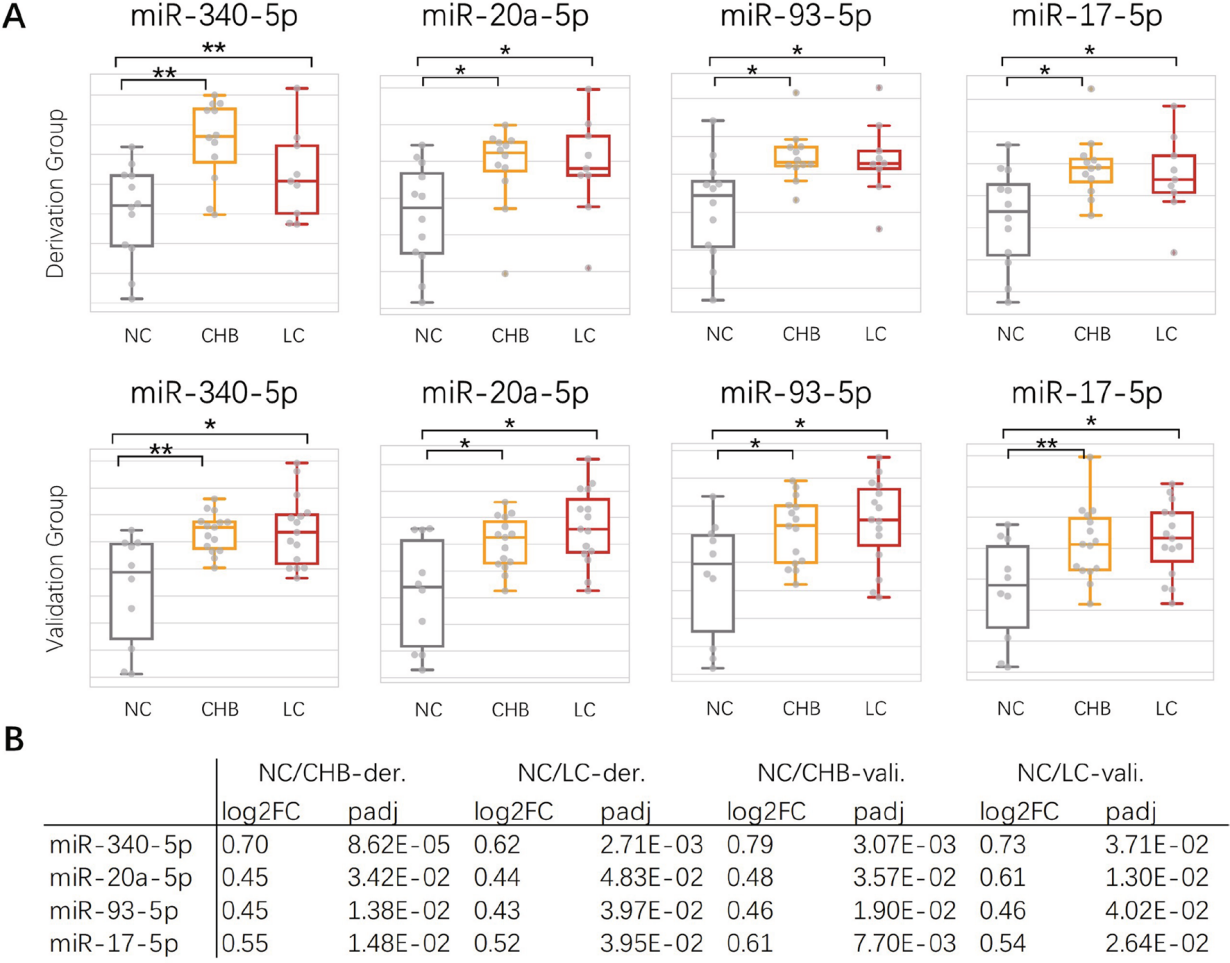
Confirmation of the expression patterns of hub miRNAs. **A** Box plot of the expression levels of four hub miRNAs in derivation group (n = 9, 12, and 12 in the LC, CHB, and NC groups) and validation group (n = 15, 15, and 10 in the LC, CHB, and NC groups). **B** Expression level sheets showing specifically expression level changes between different disease stages and healthy people (NC vs. LC, NC vs. CHB) in derivation group (n = 9, 12, and 12 in the LC, CHB, and NC groups) and validation group (n = 15, 15, and 10 in the LC, CHB, and NC groups). **padj < 0.01, *padj < 0.05

To confirm the expression patterns of the hub miRNAs in core module, we performed miRNA sequencing on an independent validation group enrolled from the same cohort, the characters of subjects enrolled were illustrated in Table 2. The expression changes of the miR-340a-5p, miR-20a-5p, miR-93-5p and miR-17-5p between different disease stages and NC group were consistent with the derivation group as shown in Fig. 4 with a significant increase from NC to CHB group and maintained this high expression level in LC group.

**Table 2:** Clinical characteristics of the patients in the RNA sequencing cohort

Characteristic	LC (n = 15)	CHB (n = 15)	NC (n = 10)
Age (years), median [Q1, Q3]	48.0 [43.5, 53.0]	42.0 [39.0, 47.5]	45.5 [35.8, 48.8]
Female (%)	3 (20.0)	3 (21.4)	1 (10.0)
Male (%)	12 (80.0)	11 (78.6)	9 (90.0)
*HBV DNA level (IU/ml)*			
≤ 2 × 10^2	15	13	–
2 × 10^2–2 × 10^6	0	2	–
> 2 × 10^6	0	0	–
Antiviral therapy (%)	15 (100)	13 (86.7)	–
*Laboratory data, mean (SD)*			
Albumin (g/L)	47.8 (3.4)	48.8 (2.1)	46.8 (2.7)
Globulin (g/L)	26.9 (3.6)	26.7 (2.2)	25.7 (2.4)
Total bilirubin (µmol/L)	17.6 (6.3)*	15.8 (6.2)*	13.1 (5.2)
Direct bilirubin (µmol/L)	6.0 (2.7)*	5.6 (3.2)*	3.6 (1.6)
Indirect bilirubin (µmol/L)	11.6 (4.8)	9.2 (3.4)	9.6 (4.7)
Alanine aminotransferase (U/L)	23.2 (11.9)	32.8 (15.0)**	30.8 (16.1)
Aspartate aminotransferase (U/L)	24.6 (8.1)	24.7 (6.9)	25.1 (9.1)
Alkaline phosphatase (U/L)	86.5 (30.0)	74.5 (16.1)	73.8 (20.5)
γ-Glutamyl transpeptidase (U/L)	36.4 (36.7)	19.9 (9.2)	31.6 (11.6)
Total bile acid (µmol/L)	7.4 (5.6)	4.2 (1.7)	5.6 (3.0)
Triglyceride (mmol/L)	1.1 (0.6)	1.1 (0.3)	2.8 (2.1)
Total cholesterol (mmol/L)	4.3 (0.9)*	4.4 (0.6)*	3.4 (1.9)
Creatinine (µmol/L)	70.9 (15.3)	76.3 (19.4)	74.8 (13.3)
Blood urea nitrogen (mmol/L)	5.2 (1.3)	4.9 (1.4)	5.3 (1.0)
K^+^ (mmol/L)	4.2 (0.4)	4.1 (0.2)	4.3 (0.3)
Na^+^ (mmol/L)	142.6 (2.1)	142.1 (1.8)	142.8 (3.5)
INR	1.0 (0.0)	0.9 (0.0)	–
White blood cells (*10^9/L)	5.5 (1.9)	5.5 (1.0)	5.5 (1.2)
Hemoglobin (g/L)	151.6 (13.2)	148.0 (13.1)	154.1 (12.4)
Platelets (*10^12/L)	160.7 (59.6)**	181.8 (53.7)*	220.9 (25.7)

*LC* Liver cirrhosis, *CHB* Chronic hepatitis B, *NC* Normal control

*p < 0.05, **p < 0.01

### Mimics transfection of miR-20a-5p and mir-340-5p initiated fibrotic processes in vitro

**Fig. 5:**
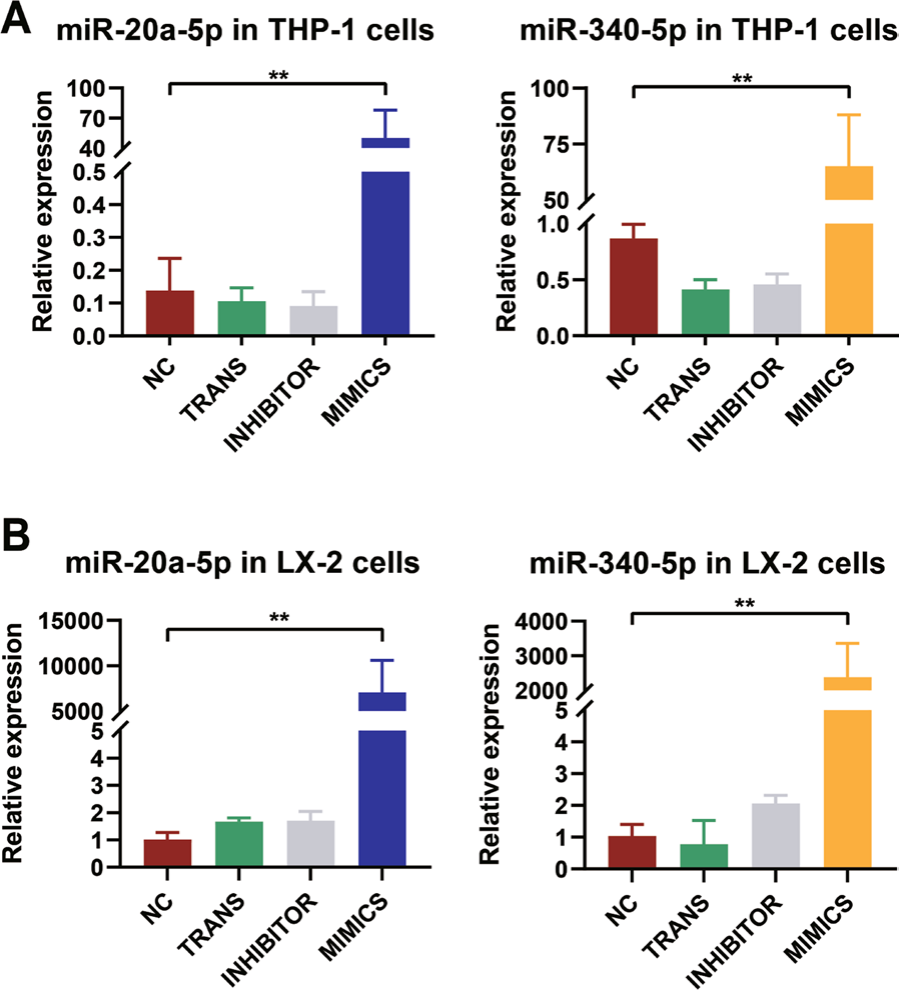
Transfection of miRNA mimics of the two hub miRNAs. **(A)** Transfection with miR-20a-5p and miR-340-5p mimics in THP-1 cells. **(B)** Transfection with miR-20a-5p and miR-340-5p mimics in LX-2 cells. The inhibitor and transfection reagent groups were used as the negative controls. The expression levels of miR-20a-5p and miR-340-5p were measured using a TaqMan assay after transfection. Numbers of replicates in each group for qRT-PCR (n = 5 in the MIMICS, INHIBITOR, TRANS and NC groups, respectively), *NC* Normal control, *TRANS* Transfection. **p < 0.001

Activation of HSCs by macrophages (Kupffer cells) is the main driver of hepatic fibrosis occurs in chronic liver fibrosis. To investigate the potentials that miRNA-20a-5p and miRNA-340-5p possessed to influence hepatic fibrosis, mimics of miRNA-20a-5p and miRNA-340-5p were transfected into a macrophage cell line (THP-1) and an HSC line (LX-2). The expression of miRNA-20a-5p and miRNA-340-5p was measured by using qRT-PCR, resulting in significantly upregulated compared to that in the normal control and transfection reagent groups after mimic transfection, while the introduction of inhibitors into these cell lines caused no significant changes in the expression levels of the two miRNAs (Fig. 5A, B), which suggested that the original expression level of miR-20a-5p and miR-340-5p in the LX-2/THP-1 are low when these cell lines were quiescent. Thus, in the following analysis, we focused on collecting the sequencing data from control (NC) and the group intervened by mRNA mimics.

**Fig. 6:**
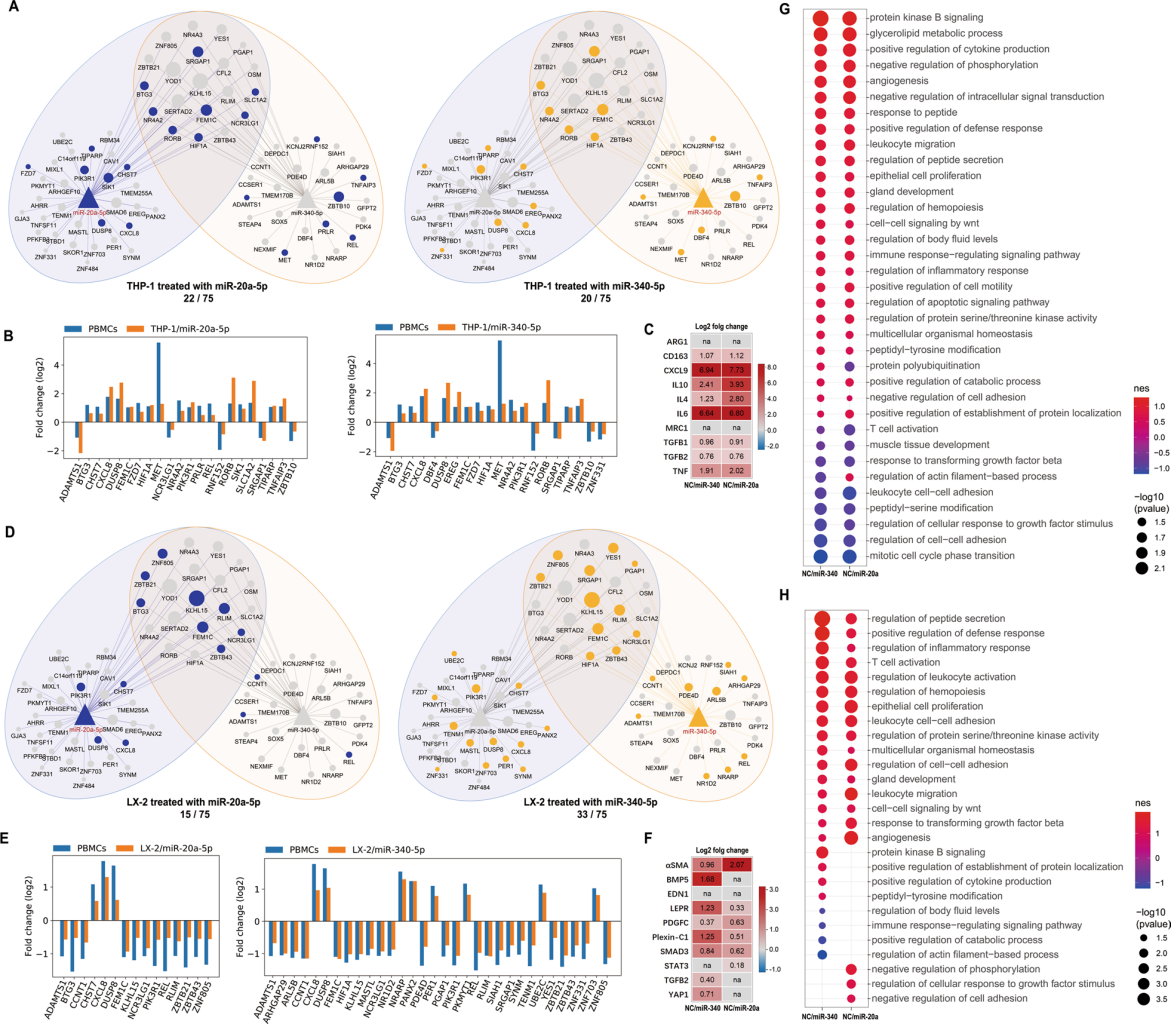
Transcriptional response of mRNAs in the core module in vitro. **A** Highlighted DE microRNAs (log_2_ |FC| ≥ 0.5, padj ≤ 0.05) having a consistent direction of the change in expression in PBMCs in THP-1/miR-20a-5p and THP-1/miR-340-5p groups. **B** Fold changes in the levels of DE microRNAs having a consistent direction of the change in expression in PBMCs in THP-1/miR-20a-5p and THP-1/miR-340-5p groups. **C** Fold changes of ten macrophage polarization marker genes before and after the mimics transfection (insignificant changes were marked as “na”). **D** Highlighted DE microRNAs (log_2_ |FC| ≥ 0.5, padj ≤ 0.05) having a consistent direction of the change in expression in PBMCs in LX-2/miR-20a-5p and LX-2/miR-340-5p groups. **E** Fold changes in the levels of DE microRNAs having a consistent direction of the change in expression in PBMCs between the LX-2/miR-20a-5p and LX-2/miR-340-5p groups. **F** Fold changes of ten HSC activation marker genes before and after the mimics transfection (insignificant changes were marked as “na”). **G** Dot plot showing the results of functional analysis of the expression changes of the core module in THP-1 after the mimic transfection of miR-20a-5p and miR-340-5p. **H** Dot plot showing the results of functional analysis of the expression changes of the core module in LX-2 after the mimic transfection of miR-20a-5p and miR-340-5p. Numbers of replicates for RNA-Seq in each group (n = 3 in the THP-1/miR-20a-5p, THP-1/miR-340-5p, LX-2/miR-20a-5p, LX-2/miR-340-5p and NC groups, respectively)

The mRNA transcriptomes of four miRNA mimic transfection groups (THP-1/miR-20a-5p, THP-1/miR-340-5p, LX-2/miR-20a-5p, and LX-2/miR-340-5p) and the corresponding normal control groups were obtained using RNA-Seq. The changes in the expression levels of the 75 mRNAs in the core module were calculated to identify the mRNAs that were differentially expressed due to miRNA mimic transfection in the four validation groups (Additional file 2: Table S16). Then, the DE miRNAs in each validation group were examined to identify the mRNAs with a change in expression in the same direction observed in at least one of the three PBMC groups (CHB/LC, LC/CHB and LC/NC). A total of 22, 20, 15 and 33 DE mRNAs in the core module were shown to have consistent expression changes between PBMCs and the THP-1/miR-20a-5p, THP-1/miR-340-5p, LX-2/miR-20a-5p, LX-2/miR-340-5p groups, respectively (Fig. 6A, D). The fold changes in their expression are shown in Fig. 6B and E.

The DE miRNAs in two cell lines were then subjected to functional analysis as shown in Fig. 6G, H. For the macrophage (THP-1), the mimics transfection of miR-20a-5p and miR-340-5p caused a significant increase of cell migration (e.g., positive regulation of cell motility), inflammation (e.g., regulation of inflammatory response), and suppression of cell-cell adhesion (e.g., regulation of cell-cell adhesion), indicating the ongoing polarization of macrophage in which cellular status transformation induced by miR-20a-5p and miR-340-5p increased the transepithelial migration capability that allow macrophage to move through the liver sinusoid to interact with HSC and participate in inflammatory response, which was confirmed by the significant upregulations of ten macrophage polarization marker genes [36, 37] according to the sequencing results (Fig. 6C). For the HSC (LX-2), the observed changes in biological processes indicated a typical HSC activation to initiate fibrotic process including collagen secretion (e.g., regulation of peptide secretion) and extracellular matrix remodeling (e.g., regulation of cell-cell adhesion, response to transforming growth factor beta), which was also confirmed by the expression differences of HSC activation marker genes (Fig. 6F) [38, 39]. In summary, the above observations demonstrated the potential of miR-20a-5p and miR-340-5p to participate in macrophage polarization and HSCs activation, which are the necessary cellular events initiating hepatic fibrogenesis in the microenvironment of liver sinusoid where the pathophysiological signals of early hepatic fibrosis can spread out to circulatory system.

## Discussion

HBV-LC is mediated by progressive pathophysiological processes involving persistent liver inflammation, creating a niche for the fibrotic process [40, 41], in which microRNAs contribute to connect a regulatory network. However, the complexity of the miRNA/mRNA network has long been neglected due to the lack of the studies designed to investigate the miRNA/mRNA regulations systematically via high-throughput methods, which provides the capability to capture disease-related signals within the real-world data from patients[13, 14, 16, 42].

In this study, we presented a network-driven approach to integrate paired miRNA and mRNA transcriptomic changes in PBMC, enabling us to observe transcriptional signals associated to hepatic fibrotic processes by investigating a cluster of closely connected miRNA/mRNA pairs, which can produce sizeable and robust functional impact on pathophysiologically important pathways mediating the disease progression, instead of a single miRNA/mRNA interaction, which commonly produce biased observations in isolation. Since a single miRNA has the potential to regulate hundreds of targets that enriched in various biological pathways, leading to the functional polymorphism of miRNA regulation, enabling a single miRNA and its potential targets to influence different pathways simultaneously. Additionally, with commonly existing feedback loops in miRNA/mRNA regulation, a miRNA can be regulator and target at the same time, forming an intricate network of miRNAs, miRNAs’ targets, and their regulators. Without considering the high variety of the relationships between miRNA and mRNA, the potential synergies in the miRNA/mRNA network have often been mis-estimated [11, 15, 43], leading to biased observations of the transcriptional status that do not properly reflect the pathophysiological processes in the long-term chronic progression of HBV-LC [44].

Therefore, the topological analysis upon the miRNA/mRNA network was designed to quantitatively measure miRNA/mRNA synergy strength based on not only their intrinsic correlations but also their impacts on the entire disease-related DE-miRNA/mRNA network to capture the miRNA/mRNA relationships that influence the pathophysiological processes most, no matter if they are classic miRNA/mRNA correlations in which miRNA suppresses their direct targets, or noncanonical miRNA/mRNA correlations produced by indirect or nonlinear interactions. Through the topological analysis, we identified the core module in the DE miRNA/mRNA network and features miR-20a-5p and miR-340-5p as its hub nodes. Further analysis revealed that the core module was involved in the continuous increase of fibrogenesis, inflammation, wound healing, and the perturbation of immune cell activation/migration. This result demonstrated the functional impact core module exerted on disease progression coupled with maintenance of fibrotic processes in which the immune response patterns changed drastically. The significant change from up to down regulation of leukocyte cell-cell adhesion and immune response-regulating signaling pathway have been observed (Fig. 2E), suggesting a potential transepithelial migration effect from PBMC to liver of leukocyte caused by the possible depletion of liver-resident immune cells in CHB exacerbation while hepatitis B virus reactivation happens. The expression level of miR-340-5p and miR-20a-5p were significantly up-regulated from NC to CHB but maintained a high level in both disease stages (CHB and LC) and had no significant changes, this result indicated a possible regulatory pattern of miR-340-5p and miR-20a-5p involved in the initiating stage of HBV-LC to start the fibrosis and maintain their regulatory ability in a steady intensity along the disease progression.

Though miR-20a-5p were reported as fibrotic and miR-340-5p involved in the HBV infection separately [45, 46], their contributions in regulating hepatic fibrosis have not been studied synergistically in the context of miRNA/mRNA network. MiR-20a-5p had the highest node centrality in the miRNA/mRNA network by topological analysis but ranked 65th in the DEA (CHB/NC, by the adjusted *p*-value (padj)), indicating the capability of topological analysis to penetrate the complexity of miRNA/mRNA network and identify its pivotal components, whose impact on disease progression might be underestimated since they might not be the most significantly differentially expressed transcripts in commonly applied analysis based merely on linear transcriptional signals.

The mimics transfections of miR-20a-5p and miR-340-5p in the THP1 and LX-2 cell lines demonstrated their potential involvements in macrophage polarization and HSC activation. Recent studies also showed that in chronically hepatic fibrosis, macrophages in the liver sinusoids aggregate and produce a series of cytokines, including transforming growth factor-β, fibroblast growth factors, platelet-derived growth factor, epidermal growth factor, etc., to activate HSCs and maintain their activations [47, 48]. In this process, the fragile balance between wound healing and fibrogenesis is regulated by a crosstalk network of cytokines and other signaling molecules [49, 50], consistent with our findings in the pathway analysis of the core module. Specifically, fibroblast growth factors receptor (FGFR) signaling pathways (e.g., R-1,226,099: Signaling by FGFR in disease), epidermal growth factor receptor (EGFR) signaling pathways (e.g., R-177,929: Signaling by EGFR), platelet-derived growth factor receptor (PDGFR) signaling pathways (e.g., R-9,671,555: Signaling by PDGFR in disease), and transforming growth factor-β signaling pathways (e.g., R-2,173,789: TGF-beta receptor signaling activates SMADs) were shown to be closely associated with the transcriptional changes in core module (Additional file 2: Tables S12, S13, S14). Though further validation and mechanism studies were required to fully clarify the molecular interactions within the core module, considering their synergetic functional impacts, miR-20a-5p and miR-340-5p can be vital components of a signaling relay to coordinate the cellular dynamics of different cell types in response to the pathophysiological changes in liver sinusoids of HBV-LC.

### Limitations

As a limitation of the present report, the association between fibrosis and miR-20a-5p/miR-340-5p should be confirmed in two additional categories of experiments, at both the RNA and protein level. Firstly, the expression of these miRNAs in activated HSC/Macrophages should be confirmed and further interventions with specific miRNA inhibitors should be performed to validate their role in the fibrotic process. Also, the interactions between miR-20a-5p/miR-340-5p and their inferred targets should be confirmed. Secondly, the fibrotic processes involving HSC activation and macrophage polarization should be confirmed with direct evidence such as functional assays.

## Conclusion

Collectively, the strategies of paired miRNA/mRNA sequencing, network-driven integration of miRNA/mRNA transcriptomic data and topological network analysis facilitated the exploration of new paradigms for deciphering complex bio-networks. And our findings of the roles miR-20a-5p/340-5p played in fibrosis helped to elucidate the molecular basis of HBV-LC, which may contribute to the development of new early warning markers of HBV-LC and treatment strategies focusing on interactions between macrophages and HSCs.

## Supplementary information

**Additional file 1.** Supplemental Materials.

**Additional file 2.** Supplemental Tables.

## Data Availability

All the data, analytic methods and study materials are available to other researchers. Data, that is raw reads from mRNA and miRNA sequencing in fastq files, are available in a public, open access repository without restrictions on the use or distribution of the data. The BioProject database project accession number for accessing the data produced for this study is PRJNA758728, and the data can be obtained from NCBI in webpage of the following link: https://www.ncbi.nlm.nih.gov/bioproject/PRJNA758728/ .

## Abbreviations

CHB: Chronic hepatitis B
DEA: Differentially expressed
EGFR: Epidermal growth factor receptor
FGFR: Fibroblast growth factors receptor
GSLA: Gene set linkage analysis
HBV: Hepatitis virus B
HSC: Hepatic stellate cell
LC: Liver cirrhosis
miRNA: MicroRNA
NC: Normal control
PBMC: Peripheral blood mononuclear cell
PDGFR: Platelet-derived growth factor receptor
TGF: Transforming growth factor
