# Supplementary material for: MiRNA/mRNA network topology in hepatitis virus B-related liver cirrhosis reveals miR-20a-5p/340-5p as hubs initiating fibrosis
Source: BMC Med Genomics. 2022 Nov 14;15:240. doi: 10.1186/s12920-022-01390-x (PMC9661777; doi:10.1186/s12920-022-01390-x)
Supplement: Supplementary file 1 — Additional file 1. Supplemental Materials. [file 12920_2022_1390_MOESM1_ESM.pdf]

Supplemental materials

Supplemental table index

Table S1. DE mRNA CHB/NC

Table S2. DE mRNA LC/CHB

Table S3. DE mRNA LC/NC

Table S4. DE miRNA CHB/NC

Table S5. DE miRNA LC/CHB

Table S6. DE miRNA LC/NC

Table S7. DE mRNA total index

Table S8. DE miRNA total index

Table S9. total miRNA/mRNA pairs

Table S10. liver tissue miRNA/mRNA pairs

Table S11. core module miRNA/mRNA pairs

Table S12. pathways both mRNA

Table S13. pathways 20a5p mRNA

Table S14. pathways 3405p mRNA

Table S15. functional Categories Assignment Map

Table S16. DE_mRNA_in_core_modules_within_THP1/LX2

Sample preparation for mRNA sequencing

PBMCs were collected from patients (CHB group and LC group) and volunteers (NC group) with Ficoll-PaqueTM PLUS medium (GE Healthcare, Uppsala, Sweden). Relevant clinical data were collected from the electronic medical records and case reports from the original subject sources. Antiviral nucleoside analogs were

immediately given to patients in the CHB and LC groups who were positive for HBV DNA. Total RNA was extracted using TRIzol reagent (Ambion, Carlsbad, CA). MicroRNA and mRNA sequencing libraries were prepared following the manufacturer's instructions (TruSeq® RNA LT Sample Prep Kit v2, Illumina, San Diego, CA) with the following steps: first-strand cDNA synthesis, second-strand cDNA synthesis, end repair by adenylation of the 3' ends, adapter ligation and DNA fragment enrichment. The sequences in the pooled library were approximately 250 nucleotides long. The pooled library was then subjected to sequencing on the HiSeq 2500 platform.

Calculation of miRNA/mRNA pairing scores

The pairing score of a miRNA/mRNA pair with a potential interaction was calculated according to the following equation:

$$\text{Comprehensive pairing score} = \sum_{k=0}^{n} I_n \times \text{Sequencing matching score.}$$

I_n is the indicator of the evidence for matching pairing—if evidence supporting its matching pairing score from any data source (miRBase, TargetScan, or microTCDs) exists, the indicator value is set to 1; otherwise, it is set to 0. The sequencing matching score from the supporting evidence was extracted from the corresponding data source. The comprehensive pairing score was defined as the sum of the product of the sequencing matching score and the corresponding evidence indicator value.

Tissue atlas screening

MiRNA data from the tissue atlas was collected from the human miRNA tissue atlas. The tissue specificity indexes of the DE miRNAs were extracted from the human miRNA tissue atlas database. The miRNAs with a tissue specificity index score greater

than 0.4 for liver tissue were selected to filter miRNA/mRNA pairs obtained from PBMC data.

Topological analysis of the miRNA/mRNA network

The eigenvector centrality was calculated to measure the influence of any given node in the miRNA/mRNA network. The eigenvector centrality can be calculated as a real number as described below.

In a graph (network) G: = (V, E), with a number of vertices |V|, the adjacency matrix of the graph is defined as $A = (a_{v,t})$, in which $a_{v,t} = 1$ if the vertex is linked to vertex t, and $a_{v,t} = 0$ otherwise.

Then, the relative centrality score of vertex v is calculated according to the following equation:

$$x_v = \frac{1}{\lambda}\sum_{t\in M(v)} x_t = \frac{1}{\lambda}\sum_{t\in G} a_{v,t}x_t \quad (1),$$

in which $M(v)$ is the set of neighbor vertices of vertex v. Equation (1) can be rearranged as follows:

$$Ax = \lambda x \quad (2).$$

According to equation (2), the eigenvector centrality is defined as the highest eigenvalue among all possible nonzero λ solutions that exist.

Cell culture and treatments

The immortalized human HSC line LX-2 and human monocytic cell line THP-1 were purchased from Cobioer Biosciences Co., Ltd. (Nanjing, China). LX-2 cells were cultured in DMEM (Thermo Fisher Scientific, Inc., Waltham, MA, USA) supplemented with 10% fetal bovine serum (FBS, Invitrogen) and 1% antibiotic solution (penicillin (100U/ml) and streptomycin (0.1mg/ml)), while THP-1 cells were cultured in RPMI

1640 medium (Thermo Fisher Scientific, Inc., Waltham, MA, USA) supplemented with 10% FBS and 0.05 mM 2-mercaptoethanol (VWR Life Science, PA, U.S.). All cells were maintained at 37°C in a humidified atmosphere containing 5% CO2. Cells were allowed to grow until 80–90% confluent and were then washed with phosphate-buffered saline (PBS). The culture medium was replaced with fresh culture medium at regular intervals. After proliferation, THP-1 cells were treated with phorbol-12-myristate-13-acetate (PMA, final concentration of 100 nM) in complete RPMI 1640 medium for 24 h and differentiated into macrophages (M0). Then, the culture medium was aspirated to remove nonadherent cells, and attached cells were rinsed three times with RPMI 1640 medium for subsequent transfection.

MiRNA mimic transfection and inhibitor introduction

Transfection of LX-2 and THP-1 cells was performed using Hieff TransTM In Vitro siRNA/miRNA Transfection Reagent (Yeasen Biotech Co. Ltd. (Shanghai, China)) following the manufacturer's protocol. Cells were seeded at a density of 5×10^5 cells/well in DMEM or complete RPMI 1640 medium and incubated for 24 h for proliferation. Then, serum-free medium and transfection cocktail were added to each well and mixed well through aspiration. MiR-20a-5p and miR-340-5p mimics (10 nM) were used to increase the expression of these miRNAs, and miR-20a-5p and miR-340-5p inhibitors (40 nM) were used as negative controls (purchased from Thermo Fisher Scientific, Inc., Waltham, MA, USA). After incubation for 6 h, the serum-free medium was discarded, and DMEM or complete RPMI 1640 medium was added to the cells and incubated for another 24 h. Normal control cells were treated with control medium.

Determination of the expression levels of miRNA

Total RNA was extracted from LX-2 cells and THP-1 cells using TRIzol reagent (Invitrogen, Inc., Waltham, MA, USA). Then the RNA was applied for reverse transcription and followed by quantitative real-time PCR using TaqMan Small RNA Assay kit (Thermo Fisher Scientific, Inc., Waltham, MA, USA) according to the manufacturer’s user guide. The qRT-PCR was performed on the ABI 7500 real time PCR system (Applied Biosystems, Foster City, CA) and the fold changes of genes were calculated using 2-ΔΔCt method normalized to RNU6B. All primers were purchased from Applied Biosystems (Foster City, CA).
