# Supplementary material for: MiRNA/mRNA network topology in hepatitis virus B-related liver cirrhosis reveals miR-20a-5p/340-5p as hubs initiating fibrosis
Source: BMC Med Genomics. 2022 Nov 14;15:240. doi: 10.1186/s12920-022-01390-x (PMC9661777; doi:10.1186/s12920-022-01390-x)
Supplement: Supplementary file 2 — Additional file 2. Supplemental Tables. [file 12920_2022_1390_MOESM2_ESM.pdf]

index	table_name
ST_1	DE_mRNA_CHB/NC
ST_2	DE_mRNA_LC/CHB
ST_3	DE_mRNA_LC/NC
ST_4	DE_miRNA_CHB/NC
ST_5	DE_miRNA_LC/CHB
ST_6	DE_miRNA_LC/NC
ST_7	DE_mRNA_total_index
ST_8	DE_miRNA_total_index
ST_9	total_miRNA/mRNA_pairs
ST_10	liver_tissue_miRNA/mRNA_pairs
ST_11	core_module_miRNA/mRNA_pairs
ST_12	pathways_both_mRNA
ST_13	pathways_20a5p_mRNA
ST_14	pathways_3405p_mRNA
ST_15	functionalCategoriesAssignmentMap
ST_16	DE_mRNA_in_core_modules_within_THP1/LX2

ST_1	DE_mRNA_CHB/NC		
gene_symbol	mRNA_acc	log2FoldChange	padj
MYBPH	ENSG00000133055	-2.617091334	0.004561479
FOLR3	ENSG00000110203	-2.11202346	0.000348266
MOCS1	ENSG00000124615	-1.961922289	0.000281305
PI3	ENSG00000124102	-1.840495937	0.023573783
FFAR2	ENSG00000126262	-1.835253277	0.001636867
HEY2	ENSG00000135547	-1.655950053	0.017476464
PPIAL4C	ENSG00000263464	-1.648511558	0.006941577
TMEM171	ENSG00000157111	-1.624994952	0.005493832
LRRN3	ENSG00000173114	-1.584379892	1.18E-06
FLRT1	ENSG00000126500	-1.504535304	0.002830921
CX3CR1	ENSG00000168329	-1.40608762	5.51E-05
KLHL34	ENSG00000185915	-1.391109599	0.009866226
TNFAIP6	ENSG00000123610	-1.370854291	0.006018601
VIT	ENSG00000205221	-1.338841096	0.008431697
GPR15	ENSG00000154165	-1.330062366	0.010034682
H2BC18	ENSG00000203814	-1.29927521	0.020970131
CISH	ENSG00000114737	-1.290472842	3.90E-05
JAM2	ENSG00000154721	-1.278099762	0.003394327
CHIT1	ENSG00000133063	-1.269472397	0.013065612
ZNF835	ENSG00000127903	-1.249265089	0.003682716
CACNA1E	ENSG00000198216	-1.24792315	0.025746648
SLC23A3	ENSG00000213901	-1.24360849	0.000629808
AHRR	ENSG00000063438	-1.240181178	0.029286238
IFIT2	ENSG00000119922	-1.230567981	0.027774357
ZNF404	ENSG00000176222	-1.228306506	2.33E-05
NPM2	ENSG00000158806	-1.21990704	0.027010774
IFIT3	ENSG00000119917	-1.202332315	0.027526643
TPO	ENSG00000115705	-1.153883777	0.035825974
LINGO2	ENSG00000174482	-1.151819199	0.031125249
NHLRC4	ENSG00000257108	-1.143698882	0.000896745
ZNF626	ENSG00000188171	-1.139006692	0.000172959
ZNF547	ENSG00000152433	-1.136754874	1.23E-05
CD180	ENSG00000134061	-1.128740305	0.000355766
BEND5	ENSG00000162373	-1.115347885	7.79E-06
CALHM1	ENSG00000185933	-1.114090818	0.04221497
GCSAM	ENSG00000174500	-1.112896175	7.25E-06
VPREB3	ENSG00000128218	-1.100651235	0.00092803
TLR10	ENSG00000174123	-1.094000579	0.000383939
ZNF781	ENSG00000196381	-1.091523299	0.000421391
PARS2	ENSG00000162396	-1.086622689	0.019707257
ADAMTS1	ENSG00000154734	-1.074797568	0.047188104
SMAD9	ENSG00000120693	-1.074292709	0.000235527
ZNF285	ENSG00000267508	-1.0736117	7.06E-05
PRICKLE2	ENSG00000163637	-1.072019527	0.014357514
PAQR8	ENSG00000170915	-1.047912424	0.012963485
TMEM169	ENSG00000163449	-1.047242988	8.16E-05
DEGS2	ENSG00000168350	-1.042458337	0.015786946
CCDC121	ENSG00000176714	-1.042373572	0.022425682

ESM1	ENSG00000164283	-1.040342712	0.011180628
GIMAP7	ENSG00000179144	-1.039171044	0.000469196
AVPR1A	ENSG00000166148	-1.025607175	0.02040243
ZNF613	ENSG00000176024	-1.005581586	3.25E-06
TMEM170B	ENSG00000205269	1.000680328	0.000114949
PRR7	ENSG00000131188	1.000696721	0.000355766
ZNF628	ENSG00000197483	1.008686479	2.33E-05
ERMN	ENSG00000136541	1.010527401	0.001276928
CDKN1C	ENSG00000129757	1.010879205	0.019117173
C3orf35	ENSG00000198590	1.013370888	0.001336633
CKAP2L	ENSG00000169607	1.020429832	0.004660463
P4HA2	ENSG00000072682	1.022199168	0.004668384
APOE	ENSG00000130203	1.02595262	0.04056662
B3GNT4	ENSG00000176383	1.028487926	0.002722389
SPRED3	ENSG00000188766	1.028828576	0.00140414
PDK4	ENSG00000004799	1.028906465	0.029455379
RGPD6	ENSG00000183054	1.036114887	0.022121923
FEM1C	ENSG00000145780	1.038251577	2.25E-06
TNFSF11	ENSG00000120659	1.03900875	0.025267865
IL13	ENSG00000169194	1.041970473	0.022290499
EREG	ENSG00000124882	1.043251719	0.004772706
NAT8L	ENSG00000185818	1.044614041	0.035184068
ATF3	ENSG00000162772	1.047908956	0.027521808
FNDC7	ENSG00000143107	1.048659379	0.018650574
PIK3R1	ENSG00000145675	1.04888521	0.000239187
SEC14L2	ENSG00000100003	1.05628529	0.015955109
TIPARP	ENSG00000163659	1.066887134	6.04E-07
IFITM10	ENSG00000244242	1.069405698	0.040762491
YOD1	ENSG00000180667	1.072638473	7.50E-06
CNDP1	ENSG00000150656	1.072926224	0.018682616
C15orf48	ENSG00000166920	1.075697462	0.006918565
CCDC74A	ENSG00000163040	1.085850761	0.040248169
ANKRD18A	ENSG00000180071	1.086257112	0.044810704
SPAG4	ENSG00000061656	1.096204654	0.004479659
GRIN2C	ENSG00000161509	1.099703056	5.51E-05
PER1	ENSG00000179094	1.100658968	0.009720587
PIM3	ENSG00000198355	1.101690045	0.000115708
HSPA12B	ENSG00000132622	1.102816014	0.004556755
RND1	ENSG00000172602	1.106320445	0.024323094
SYN1	ENSG00000008056	1.106820148	0.011117044
SIAH1	ENSG00000196470	1.107929743	2.32E-06
TNFAIP3	ENSG00000118503	1.108101877	0.006521157
RASGEF1B	ENSG00000138670	1.108636879	0.000286111
JUND	ENSG00000130522	1.10934096	5.16E-05
SLC7A5	ENSG00000103257	1.116669906	0.001401753
SCARF2	ENSG00000244486	1.117181961	0.006428757
HIF1A	ENSG00000100644	1.118188169	7.89E-05
CD83	ENSG00000112149	1.122797348	0.013775013
ZBTB21	ENSG00000173276	1.124373361	8.91E-05
LRRC32	ENSG00000137507	1.139426977	0.012941068

PRLR	ENSG00000113494	1.147495812	0.009505211
ENC1	ENSG00000171617	1.152915436	0.00080019
RHBDF1	ENSG00000007384	1.155630556	0.04737415
GFPT2	ENSG00000131459	1.156231774	0.001428577
PDXP	ENSG00000241360	1.171312403	0.006232297
HIC1	ENSG00000177374	1.18836677	0.002564519
GRIN1	ENSG00000176884	1.190386755	0.042236847
PPP1R1A	ENSG00000135447	1.195442037	0.010930523
PDE4D	ENSG00000113448	1.204719907	0.000484777
LRRC70	ENSG00000186105	1.209874637	0.00028316
BTG3	ENSG00000154640	1.210300452	0.000610222
TEX12	ENSG00000150783	1.215960952	0.001476078
ZBTB10	ENSG00000205189	1.221189265	3.25E-06
CNIH2	ENSG00000174871	1.224291411	0.000366675
ID1	ENSG00000125968	1.227686644	0.011652296
FTH1	ENSG00000167996	1.231524798	2.49E-06
ENSG00000203392	ENSG00000203392	1.237105998	0.000852916
ENSG00000228804	ENSG00000228804	1.242888806	0.018448926
HTD2	ENSG00000255154	1.244574618	0.001160303
SIK1	ENSG00000142178	1.252359555	0.008929215
TMEM160	ENSG00000130748	1.268871286	0.001644983
RHBDL1	ENSG00000103269	1.270338179	0.000164073
ENSG00000250644	ENSG00000250644	1.280030722	0.037710931
REL	ENSG00000162924	1.30530374	0.000156159
RORB	ENSG00000198963	1.311252627	0.016624612
U2AF1	ENSG00000160201	1.329100002	8.28E-07
TTC9B	ENSG00000174521	1.329981395	0.001123101
FAM81B	ENSG00000153347	1.337850291	0.016343826
IER5L	ENSG00000188483	1.338591721	0.000799262
SLC1A2	ENSG00000110436	1.358481104	0.038910882
NDUFV2	ENSG00000178127	1.385898121	5.51E-05
TMC3	ENSG00000188869	1.386672082	0.010979194
B3GNT7	ENSG00000156966	1.408900229	0.018398081
HES1	ENSG00000114315	1.412543066	0.004508371
GRASP	ENSG00000161835	1.421929488	0.00565972
MYO3B	ENSG00000071909	1.433436398	0.005418994
PDF	ENSG00000258429	1.443589169	0.01283722
KIR3DX1	ENSG00000104970	1.447198316	0.001666396
MTSS2	ENSG00000132613	1.451914241	0.000399106
NR4A2	ENSG00000153234	1.52910048	0.000274062
NRARP	ENSG00000198435	1.546028183	0.011583006
SMAD6	ENSG00000137834	1.547288923	0.008986168
SLC36A3	ENSG00000186334	1.564919297	0.048019508
TNFRSF12A	ENSG00000006327	1.569108412	6.31E-05
FAM217A	ENSG00000145975	1.575349238	0.007448908
FAM183BP	ENSG00000164556	1.579837424	0.00158447
SH3D19	ENSG00000109686	1.592724443	7.32E-05
CCIN	ENSG00000185972	1.626288104	0.000162804
DUSP8	ENSG00000184545	1.644879213	0.000138523
TCTE1	ENSG00000146221	1.651313002	0.003981023

NPTX2	ENSG00000106236	1.664578225	0.046627553
TKTL2	ENSG00000151005	1.699948936	0.027678117
ATP1B2	ENSG00000129244	1.707268617	0.002352686
GALNT5	ENSG00000136542	1.724610535	0.007389516
LMOD2	ENSG00000170807	1.779300039	0.000367584
SKOR1	ENSG00000188779	1.780072161	0.000137557
HES4	ENSG00000188290	1.788270548	0.000879232
RNF152	ENSG00000176641	1.7959528	0.009505211
ARHGAP8	ENSG00000241484	1.804728855	0.001091611
CCDC173	ENSG00000154479	1.910333033	0.00166635
UTF1	ENSG00000171794	1.98688635	0.012870587
TMEM238	ENSG00000233493	2.052653216	0.010717957
TEX45	ENSG00000198723	2.268282271	0.004104913
TEKT2	ENSG00000092850	2.368710095	0.000164746
C4orf48	ENSG00000243449	2.413846843	2.33E-05
NR4A3	ENSG00000119508	2.672914472	0.000291121
SLC12A1	ENSG00000074803	3.110045776	0.00109819

ST_2	DE_mRNA_LC/CHB		
gene_symbol	mRNA_acc	log2FoldChange	padj
NR4A3	ENSG00000119508	-3.197276725	1.95E-05
GALNT5	ENSG00000136542	-2.471655541	0.000635486
IGFBP2	ENSG00000115457	-2.449519292	0.016029972
ENSG00000258653	ENSG00000258653	-2.445085927	0.003351797
GRIK4	ENSG00000149403	-2.318579355	0.021680726
ARHGAP8	ENSG00000241484	-2.229167056	0.004254603
TKTL2	ENSG00000151005	-2.084804	0.001268759
RHCE	ENSG00000188672	-2.082972511	0.017288014
DEFA1	ENSG00000206047	-2.045127017	0.018550422
NLRP9	ENSG00000185792	-1.994101868	0.000138744
GRM3	ENSG00000198822	-1.980725392	0.024622537
LMOD2	ENSG00000170807	-1.933036396	0.000210772
RNF152	ENSG00000176641	-1.929746919	0.012799249
FAM217A	ENSG00000145975	-1.924652263	0.003095215
TCTE1	ENSG00000146221	-1.833271398	0.007019344
STEAP1B	ENSG00000105889	-1.815603209	0.038666318
C9orf131	ENSG00000174038	-1.744842894	0.005539258
SLC1A2	ENSG00000110436	-1.743368309	0.015350776
FAM183BP	ENSG00000164556	-1.73733987	0.000491463
SIGLEC1	ENSG00000088827	-1.736225822	0.011877555
TEX45	ENSG00000198723	-1.720396863	0.018715378
SH3D19	ENSG00000109686	-1.698323288	6.57E-06
ZBED2	ENSG00000177494	-1.696251534	0.000365539
GJB7	ENSG00000164411	-1.694413493	0.011233644
SMAD6	ENSG00000137834	-1.671580873	0.009828135
NDUFV2	ENSG00000178127	-1.660700578	2.43E-05
ATP1B2	ENSG00000129244	-1.652832097	0.003566582
GRASP	ENSG00000161835	-1.611563362	0.004499904
PRG2	ENSG00000186652	-1.609655472	0.005632436
B3GNT7	ENSG00000156966	-1.605509324	0.005852035
NR4A2	ENSG00000153234	-1.597887541	0.001445921
ENC1	ENSG00000171617	-1.56799722	1.30E-06
LRRC32	ENSG00000137507	-1.558597324	0.001951273
IFITM10	ENSG00000244242	-1.545558449	0.004731677
COL1A1	ENSG00000108821	-1.540225563	0.023462746
BTG3	ENSG00000154640	-1.531181785	0.000126087
REL	ENSG00000162924	-1.51899586	0.000165264
SEC14L2	ENSG00000100003	-1.47551492	0.003470537
RGPD6	ENSG00000183054	-1.466167237	0.003184812
RANBP17	ENSG00000204764	-1.445189169	0.035009915
MTSS2	ENSG00000132613	-1.442719918	0.000502493
PRH1	ENSG00000231887	-1.41762396	0.044806018
RASGEF1B	ENSG00000138670	-1.407512031	6.99E-05
ZNF483	ENSG00000173258	-1.407013364	0.00033478
ZBTB21	ENSG00000173276	-1.405652955	1.89E-05

DUSP8	ENSG00000184545	-1.402163223	0.005712937
PRLR	ENSG00000113494	-1.388492762	0.00439024
TENM1	ENSG00000009694	-1.387272085	1.89E-05
PDE4D	ENSG00000113448	-1.37518076	0.000366143
IL1RL1	ENSG00000115602	-1.371559182	0.021233658
OTOF	ENSG00000115155	-1.366858012	0.039783319
PIK3R1	ENSG00000145675	-1.362707038	1.95E-05
SIAH1	ENSG00000196470	-1.352251046	4.85E-07
MATN1	ENSG00000162510	-1.350908923	0.00481176
DDX47	ENSG00000213782	-1.336168943	1.68E-07
DOC2B	ENSG00000272636	-1.326113355	0.048287568
ZNF805	ENSG00000204524	-1.325127472	3.04E-05
U2AF1	ENSG00000160201	-1.31823499	0.001141267
CD83	ENSG00000112149	-1.315639478	0.001783706
ZBTB10	ENSG00000205189	-1.313957565	1.50E-05
ZNF14	ENSG00000105708	-1.312610473	3.28E-05
ERMN	ENSG00000136541	-1.312609229	0.000133353
ASB12	ENSG00000198881	-1.30986516	0.012242967
ENSG00000257545	ENSG00000257545	-1.301374008	0.030844035
RGPD5	ENSG00000015568	-1.297092049	0.000635672
RGS20	ENSG00000147509	-1.296296701	0.02602451
ARHGAP39	ENSG00000147799	-1.286593872	0.003832868
LRRC70	ENSG00000186105	-1.284106362	0.005280807
LINC01619	ENSG00000257242	-1.283276161	0.00038691
CA14	ENSG00000118298	-1.279662808	0.000136069
HIF1A	ENSG00000100644	-1.278831207	0.000125014
IL13	ENSG00000169194	-1.262093432	0.018366431
SPAG4	ENSG00000061656	-1.25996919	0.002003407
C4orf19	ENSG00000154274	-1.247688265	0.008179082
MYBPC2	ENSG00000086967	-1.24641337	0.009278335
GRAPL	ENSG00000189152	-1.235598483	0.028193026
NETO1	ENSG00000166342	-1.22965983	0.003778459
CCR6	ENSG00000112486	-1.216813168	0.02393678
ENSG00000227782	ENSG00000227782	-1.216502606	0.000550886
RBM44	ENSG00000177483	-1.208321965	0.000142869
HTD2	ENSG00000255154	-1.194757201	0.005898558
YES1	ENSG00000176105	-1.191702077	0.001322353
KLHL15	ENSG00000174010	-1.189631601	1.19E-06
ICOSLG	ENSG00000160223	-1.186388495	0.002314394
RBM11	ENSG00000185272	-1.184372997	0.001982196
ZNF331	ENSG00000130844	-1.166797048	0.003396706
CEMIP2	ENSG00000135048	-1.15461473	0.000240085
CCNT1	ENSG00000129315	-1.152627485	1.64E-05
CNIH2	ENSG00000174871	-1.148289555	0.00057801
RBM34	ENSG00000188739	-1.138637774	0.000119456
CCDC85C	ENSG00000205476	-1.13626603	6.16E-06
PPP1R9A	ENSG00000158528	-1.133538694	0.015153071

ARL5B	ENSG00000165997	-1.129154977	4.12E-05
EML5	ENSG00000165521	-1.128265964	0.005651472
SDE2	ENSG00000143751	-1.126736326	1.30E-06
ZMAT4	ENSG00000165061	-1.125074736	0.046904106
FNDC7	ENSG00000143107	-1.121729566	0.022659568
CFL2	ENSG00000165410	-1.121163035	0.000348929
SLC26A5	ENSG00000170615	-1.114706643	0.033346596
YOD1	ENSG00000180667	-1.112015013	3.63E-05
C3orf35	ENSG00000198590	-1.105071754	0.001516219
FEM1C	ENSG00000145780	-1.099925883	3.04E-05
SRGAP1	ENSG00000196935	-1.09719404	0.014481058
RBFOX3	ENSG00000167281	-1.09521236	0.000699726
ENSG00000269570	ENSG00000269570	-1.094303678	0.023395479
ZBTB43	ENSG00000169155	-1.091857722	1.43E-05
ANKRD18A	ENSG00000180071	-1.090649052	0.040114041
PGAP1	ENSG00000197121	-1.08957675	0.000752172
SCN3B	ENSG00000166257	-1.087093474	0.015756944
PIGA	ENSG00000165195	-1.082941283	1.45E-05
HSPA12B	ENSG00000132622	-1.079160631	0.019943455
ZBED6	ENSG00000257315	-1.079038034	0.010878038
ZNF484	ENSG00000127081	-1.077968068	0.001873449
ENSG00000152454	ENSG00000152454	-1.077374507	8.83E-06
NR1D2	ENSG00000174738	-1.069091438	0.000575202
NCR3LG1	ENSG00000188211	-1.066658955	0.003163908
RLIM	ENSG00000131263	-1.062575142	0.000111709
SERTAD2	ENSG00000179833	-1.061240812	6.27E-06
PFKFB3	ENSG00000170525	-1.060951404	0.006104082
SC5D	ENSG00000109929	-1.059415558	0.001963489
RFX8	ENSG00000196460	-1.056160664	0.010880607
CHD1	ENSG00000153922	-1.056148382	0.000670462
DBF4	ENSG00000006634	-1.054499251	0.001275927
IGFALS	ENSG00000099769	-1.051638205	0.034412491
ARHGAP29	ENSG00000137962	-1.05155325	0.013685491
ENSG00000262180	ENSG00000262180	-1.049026073	0.001272287
MASTL	ENSG00000120539	-1.04892864	8.49E-05
ACKR4	ENSG00000129048	-1.046866342	0.007193451
GCSAML	ENSG00000169224	-1.043558009	0.015976663
RNF103	ENSG00000239305	-1.040907846	0.000133353
C12orf71	ENSG00000214700	-1.03938525	0.005491073
TMEM255A	ENSG00000125355	-1.033930373	0.002081482
SOX5	ENSG00000134532	-1.03072164	0.034622706
SYNM	ENSG00000182253	-1.030719469	0.049382883
TMEM170B	ENSG00000205269	-1.024644561	0.000375396
ERRFI1	ENSG00000116285	-1.017619877	0.00027744
PER1	ENSG00000179094	-1.01760255	0.046662002
GFPT2	ENSG00000131459	-1.016359654	0.038903258
CCSER1	ENSG00000184305	-1.014819686	0.017288014

TNFSF9	ENSG00000125657	-1.012827499	0.02529364
GOLGA8M	ENSG00000188626	-1.01173792	0.039179563
SCML1	ENSG00000047634	-1.006540051	0.000233882
PKD2L2	ENSG00000078795	-1.002914112	0.000126087
HBQ1	ENSG00000086506	1.000892188	0.048171624
SEMA3G	ENSG00000010319	1.003685816	0.010926061
MON1A	ENSG00000164077	1.008745192	0.001539275
ANTKMT	ENSG00000103254	1.012452656	0.008656099
FCRLB	ENSG00000162746	1.015282929	0.004861075
STBD1	ENSG00000118804	1.015851449	0.028608593
FAM43A	ENSG00000185112	1.017323194	0.003937144
EFNA3	ENSG00000143590	1.018299642	0.028187505
TMEM204	ENSG00000131634	1.020140109	0.003444462
PARS2	ENSG00000162396	1.023849936	0.034886226
FKBPL	ENSG00000204315	1.025594565	0.000405859
GIMAP1	ENSG00000213203	1.0256629	0.000112024
ZNF547	ENSG00000152433	1.031303838	0.000946597
TREML2	ENSG00000112195	1.032774977	0.042067779
TICRR	ENSG00000140534	1.033954373	0.016068589
TXNDC5	ENSG00000239264	1.040863043	0.029325339
ASCL2	ENSG00000183734	1.046441332	0.005399291
C14orf119	ENSG00000179933	1.050052655	0.000126087
LRFN1	ENSG00000128011	1.050939616	8.49E-05
MYBL2	ENSG00000101057	1.052983347	0.020826579
COL9A3	ENSG00000092758	1.053597402	0.007576932
GPR68	ENSG00000119714	1.054325755	0.002567806
NEK2	ENSG00000117650	1.054430377	0.030574489
HSPA6	ENSG00000173110	1.064199722	0.048196645
NHLRC1	ENSG00000187566	1.067937744	0.018500264
ZNF837	ENSG00000152475	1.070166797	0.004146196
TNFRSF9	ENSG00000049249	1.07084865	0.000481117
BOLA1	ENSG00000178096	1.075872509	4.78E-05
TM4SF19	ENSG00000145107	1.076750371	0.037706359
GIMAP7	ENSG00000179144	1.077735664	0.002202577
MCM10	ENSG00000065328	1.078992449	0.012336662
ADGRE3	ENSG00000131355	1.080438037	0.005778789
AVPR1A	ENSG00000166148	1.080525077	0.008286019
PYCR3	ENSG00000104524	1.083757735	0.001882098
LRRIQ3	ENSG00000162620	1.093179413	0.018836323
RPP25L	ENSG00000164967	1.096756473	0.000203037
RTN4R	ENSG00000040608	1.099321273	0.014577337
TMEM160	ENSG00000130748	1.099868291	0.018196475
CD180	ENSG00000134061	1.101593091	0.000811293
CAV1	ENSG00000105974	1.102246026	0.03228537
BORCS6	ENSG00000196544	1.103778231	1.01E-05
BTN1A1	ENSG00000124557	1.105646383	0.037402351
B3GNT8	ENSG00000177191	1.111178799	0.00040083

DUSP1	ENSG00000120129	1.113027816	0.008805282
FCGR1CP	ENSG00000265531	1.119586028	0.017551485
SOWAHD	ENSG00000187808	1.12147268	0.000405979
OSM	ENSG00000099985	1.127799181	0.007759332
CCDC121	ENSG00000176714	1.128823025	0.009397284
CEP19	ENSG00000174007	1.130373996	0.006485643
ENSG00000258674	ENSG00000258674	1.131153056	0.003595587
AURKA	ENSG00000087586	1.134840376	0.001787753
TMEM250	ENSG00000238227	1.136503211	0.000534553
FASLG	ENSG00000117560	1.139303758	0.002830756
ZNF835	ENSG00000127903	1.139385963	0.001783706
GJA3	ENSG00000121743	1.142364553	0.037653334
DGAT2	ENSG00000062282	1.148261436	0.03381742
ARL2BP	ENSG00000102931	1.14859615	0.035597099
TNFAIP8L2	ENSG00000163154	1.153225463	0.000640433
NLRP6	ENSG00000174885	1.15773505	0.010641737
ISL2	ENSG00000159556	1.165628386	0.003152013
SLC22A1	ENSG00000175003	1.167520035	0.033517933
ZNF696	ENSG00000185730	1.174031331	7.40E-08
FCGR1B	ENSG00000198019	1.177215689	0.00692177
CTU1	ENSG00000142544	1.177274749	0.004497261
FAM50B	ENSG00000145945	1.17908008	6.27E-06
SLC23A3	ENSG00000213901	1.182479232	0.005464582
CILP	ENSG00000138615	1.184291981	0.000399054
ASB2	ENSG00000100628	1.186675633	6.77E-06
C4orf48	ENSG00000243449	1.186679538	0.028488537
DLGAP5	ENSG00000126787	1.189137681	0.024721606
S1PR4	ENSG00000125910	1.189735251	9.84E-05
CDC42EP2	ENSG00000149798	1.190189051	0.001458414
HSPA1A	ENSG00000204389	1.191928756	0.000439943
KISS1R	ENSG00000116014	1.19901142	0.035951094
FPR3	ENSG00000187474	1.199843202	0.015374572
SSPN	ENSG00000123096	1.199890051	0.002512384
S100A3	ENSG00000188015	1.209484829	0.01358657
KIAA1324	ENSG00000116299	1.211799847	0.002253824
CEBPD	ENSG00000221869	1.213077096	0.000551768
RHOB	ENSG00000143878	1.224938269	2.68E-05
FUT7	ENSG00000180549	1.233174295	2.65E-06
JCHAIN	ENSG00000132465	1.233669272	0.04348626
H2AC18	ENSG00000203812	1.239317594	0.004526418
CISH	ENSG00000114737	1.239712305	0.000286419
BATF2	ENSG00000168062	1.243597236	0.008267693
ABCB9	ENSG00000150967	1.24687051	0.001413519
PANX2	ENSG00000073150	1.250141099	0.003925732
FBLN5	ENSG00000140092	1.260476936	0.00137068
OXCT2	ENSG00000198754	1.270793966	0.01825714
CALHM1	ENSG00000185933	1.273264694	0.021967136

ACTL10	ENSG00000182584	1.288233549	0.005754597
XCR1	ENSG00000173578	1.290760438	0.002686701
ENDOG	ENSG00000167136	1.294410916	0.014586361
CCN3	ENSG00000136999	1.294673607	0.044593229
STEAP4	ENSG00000127954	1.295629992	0.02761204
AOC2	ENSG00000131480	1.313927	0.00258717
BHLHA15	ENSG00000180535	1.321777047	0.021956421
KCNJ2	ENSG00000123700	1.333854342	0.034995099
FOS	ENSG00000170345	1.337942682	0.001041009
FZD7	ENSG00000155760	1.345192863	0.003060651
MIXL1	ENSG00000185155	1.34877605	0.015980962
MMP25	ENSG00000008516	1.350817422	0.03547025
AOC3	ENSG00000131471	1.361440187	0.001751038
NPM2	ENSG00000158806	1.393478555	0.018715378
GPR15	ENSG00000154165	1.39622477	0.002667795
VIT	ENSG00000205221	1.407422543	0.025169889
SKA3	ENSG00000165480	1.408961011	0.0026154
PYCR1	ENSG00000183010	1.444239558	0.011383169
FA2H	ENSG00000103089	1.444911393	0.015835486
LRG1	ENSG00000171236	1.454612337	0.047629184
NHLRC4	ENSG00000257108	1.459306999	0.000254269
CHI3L1	ENSG00000133048	1.50190214	0.048227396
CX3CR1	ENSG00000168329	1.523925908	0.001327596
ANGPTL6	ENSG00000130812	1.525799382	0.000294101
H2BC18	ENSG00000203814	1.545804402	0.005789121
ENSG00000253633	ENSG00000253633	1.606126991	0.047907464
DHRS9	ENSG00000073737	1.606697164	4.89E-05
IDO1	ENSG00000131203	1.614638968	0.000409523
PDF	ENSG00000258429	1.620370811	0.004080493
TMEM171	ENSG00000157111	1.655336339	0.024721606
GGT5	ENSG00000099998	1.664668827	0.008815458
ARHGEF10	ENSG00000104728	1.696040391	0.045543375
GPRC5D	ENSG00000111291	1.697390344	0.003686961
SDC1	ENSG00000115884	1.716685275	0.001250017
MANSC1	ENSG00000111261	1.717799585	0.012807371
ROR2	ENSG00000169071	1.729363334	0.02458143
GLDC	ENSG00000178445	1.743288742	0.006263672
TNFRSF10C	ENSG00000173535	1.744508038	0.043685789
CXCL8	ENSG00000169429	1.779324455	0.036516315
CDC20	ENSG00000117399	1.790321424	0.000509921
SRXN1	ENSG00000271303	1.804030023	0.023031324
APOBEC3B	ENSG00000179750	1.811963522	0.02521673
NT5DC4	ENSG00000144130	1.856062184	0.005299476
SMIM11A	ENSG00000205670	1.858198887	0.000110691
TNFAIP6	ENSG00000123610	2.047556771	3.04E-05
ENSG00000268170	ENSG00000268170	2.110922747	0.012654409
GDF10	ENSG00000266524	2.111960525	0.00213343

TNFRSF17	ENSG00000048462	2.139548018	0.000887943
OTX1	ENSG00000115507	2.207195382	0.023359636
KRT23	ENSG00000108244	2.374867594	0.005338328
SPP1	ENSG00000118785	2.885826642	0.017335305
BTBD17	ENSG00000204347	3.074910862	0.005418732
AOC1	ENSG00000002726	3.301162047	0.000446293
PI3	ENSG00000124102	3.639675714	4.36E-05
MET	ENSG00000105976	5.571245501	2.26E-10

ST_3	DE_mRNA_LC/NC		
mRNA_gene_symbol	mRNA_acc	log2FoldChange	padj
DEFA1	ENSG00000206047	-1.922580243	0.026943532
ZMAT4	ENSG00000165061	-1.733200324	0.015455987
DOC2B	ENSG00000272636	-1.588016342	0.021377947
GTSCR1	ENSG00000263417	-1.421191261	0.02152063
PRECSIT	ENSG00000255874	-1.33748914	0.048220749
CHRNB2	ENSG00000160716	-1.175389863	0.034053302
MCOLN3	ENSG00000055732	-1.101337057	0.003531477
NETO1	ENSG00000166342	-1.089002543	0.006635362
TOM1L1	ENSG00000141198	-1.088766345	0.045772269
ZNF135	ENSG00000176293	-1.061271636	0.005080481
NIPAL4	ENSG00000172548	-1.054774153	0.005508168
SORCS3	ENSG00000156395	-1.05411559	0.029508602
NEXMIF	ENSG00000050030	-1.05043395	0.020895601
RASGRF1	ENSG00000058335	-1.040035652	0.043212532
CD24	ENSG00000272398	-1.008504034	0.004138321
COMTD1	ENSG00000165644	1.000908083	5.88E-05
ATP5F1D	ENSG00000099624	1.013219263	3.37E-05
MZB1	ENSG00000170476	1.013822846	0.031221431
MRPL12	ENSG00000262814	1.01889574	0.048265109
PRR7	ENSG00000131188	1.022596738	0.001127429
ZNF703	ENSG00000183779	1.025785233	4.71E-05
GLTPD2	ENSG00000182327	1.033557677	0.027360318
SFN	ENSG00000175793	1.043308224	0.04287296
IDO1	ENSG00000131203	1.043562387	0.022471155
ACTL10	ENSG00000182584	1.043923275	0.036576078
CDKN1C	ENSG00000129757	1.057861595	0.04505308
H2AX	ENSG00000188486	1.058301018	5.48E-07
CKAP2L	ENSG00000169607	1.060619704	0.039043375
CLEC11A	ENSG00000105472	1.064520731	0.015347955
ZNF579	ENSG00000218891	1.07093105	2.59E-09
BUB1	ENSG00000169679	1.072660986	0.029169329
SCX	ENSG00000260428	1.073016173	0.004069256
HJURP	ENSG00000123485	1.075291097	0.032402153
HES1	ENSG00000114315	1.083265531	0.047880582
CHST7	ENSG00000147119	1.084503814	9.96E-09
KLF2	ENSG00000127528	1.106266076	8.39E-10
ENSG00000258130	ENSG00000258130	1.11079841	0.006068728
KIF18B	ENSG00000186185	1.113658212	0.036641195
INAFM1	ENSG00000257704	1.124904668	1.83E-08
CACNG6	ENSG00000130433	1.130302336	0.035015219
UBE2C	ENSG00000175063	1.142127145	0.022471155
JUND	ENSG00000130522	1.142792595	9.32E-05
ISL2	ENSG00000159556	1.150068219	0.038560749
NDUFAF8	ENSG00000224877	1.158003452	5.62E-09
FZD7	ENSG00000155760	1.159395185	0.025741581

PKMYT1	ENSG00000127564	1.170974384	0.013466193
JSRP1	ENSG00000167476	1.172404872	0.00066569
DOCK6	ENSG00000130158	1.179852344	0.029833564
CFD	ENSG00000197766	1.183418138	2.75E-06
ABCB9	ENSG00000150967	1.184316757	0.002634942
ADAT3	ENSG00000213638	1.185575821	3.96E-05
IGLL5	ENSG00000254709	1.191636422	0.018241067
MBLAC1	ENSG00000214309	1.193509176	0.002831971
ANGPTL6	ENSG00000130812	1.19950406	0.009114319
MMP23B	ENSG00000189409	1.207030885	0.001970255
DLGAP5	ENSG00000126787	1.221105811	0.038560749
E2F8	ENSG00000129173	1.221221532	0.024876973
SKA3	ENSG00000165480	1.227649907	0.015501507
OXCT2	ENSG00000198754	1.229930717	0.030515447
ANTKMT	ENSG00000103254	1.266430897	1.77E-08
CENPA	ENSG00000115163	1.271750035	0.005154471
MIXL1	ENSG00000185155	1.277237149	0.046776688
CTU1	ENSG00000142544	1.277319326	4.44E-06
BIRC5	ENSG00000089685	1.288435858	0.013610764
CHCHD10	ENSG00000250479	1.293566122	9.74E-07
TXNDC5	ENSG00000239264	1.302977246	0.00777402
C2CD4D	ENSG00000225556	1.311190262	0.017014015
CCDC85B	ENSG00000175602	1.316504003	4.76E-09
SPC25	ENSG00000152253	1.31787244	0.032999824
DIAPH3	ENSG00000139734	1.329793434	0.046893865
DDT	ENSG00000099977	1.334360634	4.37E-08
ADRB1	ENSG00000043591	1.345051494	0.005072089
ASCL2	ENSG00000183734	1.349147582	3.88E-08
DEPDC1	ENSG00000024526	1.398183784	0.033461778
TNFRSF17	ENSG00000048462	1.40001708	0.019851709
H1-10	ENSG00000184897	1.462043221	3.42E-09
IER5L	ENSG00000188483	1.465140945	5.77E-05
RAB5IF	ENSG00000101084	1.481049744	1.61E-09
RHOF	ENSG00000139725	1.500384999	9.96E-09
EVA1B	ENSG00000142694	1.503078623	1.86E-05
CITED4	ENSG00000179862	1.515557775	2.00E-06
SOCS1	ENSG00000185338	1.531751128	0.007815435
ZNF804A	ENSG00000170396	1.539513635	8.10E-14
CEBPD	ENSG00000221869	1.544311415	1.32E-07
CEBPB	ENSG00000172216	1.556069033	4.40E-08
SHISA8	ENSG00000234965	1.582718925	2.60E-06
NEK2	ENSG00000117650	1.613639684	0.001071371
FA2H	ENSG00000103089	1.613649909	0.010306183
BHLHA15	ENSG00000180535	1.658169771	0.00386691
PCSK1N	ENSG00000102109	1.661497537	0.001071371
TNNT1	ENSG00000105048	1.67578066	0.006969618
ENDOG	ENSG00000167136	1.689575518	9.45E-06

CDC20	ENSG00000117399	1.91653159	0.002203407
ARL2BP	ENSG00000102931	1.935819177	0.000438588
NT5DC4	ENSG00000144130	2.018455425	0.005059672
HES4	ENSG00000188290	2.11650278	3.68E-06
C17orf97	ENSG00000187624	2.128303565	0.005613041
FTH1	ENSG00000167996	2.217232617	3.07E-32
TMEM160	ENSG00000130748	2.373483418	4.00E-26
APOBEC3B	ENSG00000179750	2.387610553	0.014572103
ROR2	ENSG00000169071	2.607878731	0.001554045
PDF	ENSG00000258429	3.070018646	8.39E-10
TMEM238	ENSG00000233493	3.208034272	1.53E-07
C4orf48	ENSG00000243449	3.606172731	8.01E-19
MET	ENSG00000105976	5.569063718	1.58E-09

ST_4	DE_miRNA_CHB/NC	
miRNA_acc	log2FoldChange	pvalue
hsa-miR-101-3p	-13.41742217	1.51E-23
hsa-miR-124-5p	-4.215794334	5.26E-09
hsa-miR-877-5p	-4.67362073	3.12E-07
hsa-miR-3613-5p	0.960908532	9.17E-07
hsa-miR-129-5p	-2.605390624	5.05E-05
hsa-miR-340-5p	0.700430426	8.62E-05
hsa-miR-4425	1.711632125	0.000157155
hsa-miR-664b-3p	-2.859878225	0.00026392
hsa-miR-3605-5p	-2.515665418	0.000254543
hsa-miR-548s	1.880452059	0.000404553
hsa-miR-4802-5p	1.33550536	0.000468075
hsa-miR-1291	-2.522189504	0.000526499
hsa-miR-99b-5p	-0.717268975	0.000630096
hsa-miR-1234-3p	-2.035448615	0.000896771
hsa-miR-190b	0.479380341	0.00116072
hsa-miR-619-5p	-2.129835682	0.001307203
hsa-miR-3064-5p	-1.998346358	0.001483244
hsa-miR-500a-5p	0.425074464	0.001646372
hsa-miR-548ah-3p	-2.129778284	0.001833064
hsa-miR-32-5p	0.456837489	0.002389203
hsa-miR-513b-5p	-1.816877492	0.003465152
hsa-miR-574-5p	0.436304073	0.003849596
hsa-miR-641	0.609553998	0.005878151
hsa-miR-4474-5p	1.450855772	0.00595299
hsa-miR-206	-1.773188972	0.006013798
hsa-miR-580-5p	1.097100146	0.006611299
hsa-miR-1262	1.293317609	0.006824485
hsa-miR-1908-5p	1.872382495	0.007109579
hsa-miR-196a-5p	0.675079964	0.007416598
hsa-miR-144-5p	1.012684043	0.008083408
hsa-miR-4661-5p	-1.234717358	0.009220138
hsa-miR-651-5p	0.405794625	0.010431725
hsa-miR-590-5p	0.701531796	0.011320781
hsa-miR-218-5p	-1.979980417	0.011037981
hsa-miR-103a-2-5p	0.422645636	0.014647234
hsa-miR-93-5p	0.452742303	0.013847589
hsa-miR-17-5p	0.545804342	0.014826886
hsa-miR-660-5p	0.595924758	0.012785673
hsa-miR-1537-5p	0.856568421	0.012373888
hsa-miR-708-5p	2.348625651	0.014174349
hsa-miR-103a-3p	0.422994238	0.014735281
hsa-miR-219a-5p	-1.424880471	0.012825922
hsa-miR-6837-5p	1.770998029	0.013706439
hsa-miR-204-5p	-1.644306687	0.014463269
hsa-miR-374a-5p	0.465898171	0.01683403

hsa-miR-511-5p	1.463164129	0.017768601
hsa-miR-21-5p	0.261937914	0.023598457
hsa-miR-190a-5p	1.005799479	0.022701557
hsa-miR-4632-5p	1.403605996	0.022365378
hsa-miR-4482-5p	-1.699518234	0.023487357
hsa-miR-1179	1.33319428	0.022102071
hsa-miR-548x-5p	-1.359255168	0.023311784
hsa-miR-616-5p	1.152886715	0.024076045
hsa-miR-501-5p	0.257994255	0.026641834
hsa-miR-7854-3p	0.83056638	0.025525381
hsa-miR-556-5p	0.699641128	0.026454203
hsa-miR-6770-5p	1.629110321	0.026880764
hsa-miR-4717-5p	1.681078556	0.02649641
hsa-miR-4709-5p	1.276465005	0.027874846
hsa-miR-4775	1.175490616	0.027686086
hsa-miR-4645-5p	0.411498316	0.028705251
hsa-miR-942-5p	0.233584714	0.030804458
hsa-miR-371b-5p	1.226431385	0.030406948
hsa-miR-625-5p	-0.599016874	0.031706733
hsa-miR-20a-5p	0.448888206	0.034193469
hsa-miR-15b-5p	0.365900039	0.034096847
hsa-miR-624-5p	0.914174627	0.034996066
hsa-miR-301b-5p	1.134278525	0.034811299
hsa-miR-454-5p	0.406884355	0.033161654
hsa-miR-6837-3p	-1.442885173	0.033137898
hsa-miR-1303	1.499774903	0.035577414
hsa-miR-6513-5p	0.566923811	0.036123231
hsa-miR-98-5p	0.304560117	0.038173434
hsa-miR-628-5p	0.431201955	0.037571229
hsa-miR-188-5p	0.92921344	0.037715766
hsa-miR-502-5p	0.377417191	0.04076653
hsa-miR-199b-5p	0.392503063	0.043905392
hsa-miR-19a-5p	0.33157269	0.043496165
hsa-miR-1249-5p	0.427989943	0.043587408
hsa-miR-92b-5p	-0.311576914	0.042709707
hsa-miR-362-5p	0.529228259	0.044743149
hsa-miR-10a-5p	-0.535515365	0.04714053
hsa-miR-1228-5p	1.366929013	0.048022863
hsa-miR-3176	0.910184369	0.048100098
hsa-miR-935	1.071371641	0.048154263

ST_5	DE_miRNA_LC/CHB	
miRNA_acc	log2FoldChange	pvalue
hsa-miR-4999-5p	-3.486526407	0.000165739
hsa-miR-6837-5p	-2.950082775	0.000310659
hsa-miR-376a-2-5p	-3.039714853	0.000362614
hsa-miR-6770-5p	-2.813135474	0.000786734
hsa-miR-1299	-3.108484682	0.000853494
hsa-miR-3611	-2.776483738	0.000916023
hsa-miR-6813-5p	2.67972438	0.001125939
hsa-miR-122-5p	-2.897393248	0.001153086
hsa-miR-4781-5p	-2.598244403	0.002040094
hsa-miR-204-5p	2.190963067	0.002732086
hsa-miR-3150a-5p	1.954936158	0.003181086
hsa-miR-1234-3p	1.983909952	0.003367029
hsa-miR-6812-5p	-2.300946978	0.003510185
hsa-miR-4772-5p	0.642338648	0.004175196
hsa-miR-548ba	-2.460769725	0.00441525
hsa-miR-3180-5p	-2.550956991	0.004641923
hsa-miR-544b	-2.355668058	0.005437478
hsa-miR-4746-5p	2.335215173	0.005969643
hsa-miR-548a-5p	-2.460918689	0.007764288
hsa-miR-5695	1.530526405	0.010665962
hsa-miR-490-5p	-2.04792782	0.010721772
hsa-miR-889-5p	-2.378089599	0.011083659
hsa-miR-4802-5p	-0.723803779	0.011296714
hsa-miR-3144-5p	1.718083203	0.013797292
hsa-miR-6515-5p	-2.04748457	0.013871182
hsa-miR-506-5p	-1.70796409	0.014964764
hsa-miR-4485-5p	-1.785701509	0.019873417
hsa-miR-4659b-5p	-1.869354115	0.020572281
hsa-miR-1972	-3.300211356	0.022005052
hsa-miR-4433a-5p	1.730814567	0.023216369
hsa-miR-539-5p	-1.780586428	0.023668074
hsa-miR-4724-5p	1.589191082	0.024045781
hsa-miR-4494	1.501460482	0.024917298
hsa-miR-3684	-1.671260621	0.025272428
hsa-miR-449a	1.410998451	0.026081553
hsa-miR-3945	-1.7432231	0.027832187
hsa-miR-4677-5p	-0.287569394	0.028833305
hsa-miR-1228-5p	-1.632893275	0.031019413
hsa-miR-432-5p	1.067738038	0.031680512
hsa-miR-193b-3p	-1.718229305	0.032283063
hsa-miR-876-5p	-1.806539971	0.03269724
hsa-miR-4424	-1.21764068	0.036986896
hsa-miR-504-5p	1.196532786	0.038336191
hsa-miR-3143	1.502442582	0.040078613
hsa-miR-7852-3p	-1.464652812	0.045125476

hsa-miR-7110-5p	-1.445317188	0.048777666
hsa-miR-513a-5p	-1.562198397	0.049117182
hsa-miR-551b-5p	-1.347875952	0.049685541

ST_6	DE_miRNA_LC/NC	
miRNA_acc	log2FoldChange	pvalue
hsa-miR-1299	-3.986262828	4.25E-05
hsa-miR-3611	-2.814450078	4.61E-05
hsa-miR-544b	-3.348510022	4.92E-05
hsa-miR-548s	2.08287148	7.69E-05
hsa-miR-889-5p	-3.377099212	0.000232978
hsa-miR-548ai	-2.474432105	0.00024217
hsa-miR-4717-5p	2.488035221	0.000289699
hsa-miR-6837-3p	-2.700251983	0.000330769
hsa-miR-3605-5p	-2.765113313	0.000502578
hsa-miR-1179	1.830031214	0.000658917
hsa-miR-877-5p	-3.350983528	0.000682438
hsa-miR-1291	-2.786095627	0.00095972
hsa-miR-122-5p	-2.590225151	0.000960684
hsa-miR-664b-3p	-2.805829956	0.001074101
hsa-miR-5684	-2.206081026	0.001335839
hsa-miR-619-5p	-2.374190441	0.001824742
hsa-miR-539-5p	-2.320715445	0.001931429
hsa-miR-548ah-3p	-2.384922126	0.002447814
hsa-miR-129-5p	-2.168364716	0.002515664
hsa-miR-340-5p	0.621723668	2.71E-03
hsa-miR-124-5p	-2.519336579	0.00557415
hsa-miR-9-5p	-0.689365211	0.005784076
hsa-miR-190b	0.504254869	0.006536701
hsa-miR-758-5p	-1.904152046	0.007123506
hsa-miR-410-5p	1.699636652	0.008077397
hsa-miR-506-5p	-1.772051012	0.00816011
hsa-miR-301b-5p	1.347859196	0.00916487
hsa-miR-147b	-1.793144085	0.011267964
hsa-miR-548ah-5p	-1.754949878	0.011432142
hsa-miR-548x-5p	-1.765378431	0.011597095
hsa-miR-187-5p	-1.758500668	0.011693123
hsa-miR-3065-5p	1.360658886	0.01302526
hsa-miR-1256	-1.669549222	0.013429244
hsa-miR-641	1.32396516	0.013729237
hsa-miR-30c-5p	0.420505423	0.013874623
hsa-miR-365b-5p	0.495826582	0.014664046
hsa-miR-371b-5p	1.381526103	0.014901281
hsa-miR-548a-5p	-1.911131502	0.015251089
hsa-miR-5680	1.289400982	0.015543542
hsa-miR-24-1-5p	0.61574096	0.015577832
hsa-miR-10a-5p	-0.816612809	0.016463346
hsa-miR-885-5p	1.159131888	0.019113488
hsa-miR-144-5p	0.93099215	0.019988779
hsa-miR-4709-5p	1.366592988	0.019991034
hsa-miR-125b-5p	-0.803579019	0.020387462

hsa-miR-3074-5p	0.472739157	0.021772475
hsa-miR-5582-5p	1.311173795	0.021850885
hsa-miR-6733-5p	1.187227739	0.022642615
hsa-miR-365a-5p	0.474271195	0.023181723
hsa-miR-943	-1.497016307	0.02609531
hsa-miR-574-5p	0.448084605	0.026712484
hsa-miR-199b-5p	0.510542579	0.027285713
hsa-miR-942-5p	0.309949949	0.027867998
hsa-miR-4423-5p	1.396986435	0.028246482
hsa-miR-153-3p	-1.020853341	0.035439982
hsa-miR-6737-5p	1.183434466	0.035492851
hsa-miR-6502-5p	-1.498039905	0.035984665
hsa-miR-17-5p	0.519312448	0.039458995
hsa-miR-93-5p	0.428483618	0.039731675
hsa-miR-193a-5p	0.73383893	0.039795794
hsa-miR-552-5p	-1.332687371	0.040569746
hsa-miR-6734-5p	1.282624922	0.042893605
hsa-miR-597-5p	0.797592134	0.043255624
hsa-miR-1908-5p	1.599215938	0.043910311
hsa-miR-4491	1.540663114	0.047055413
hsa-miR-20a-5p	0.439787206	0.04828776
hsa-miR-490-5p	-1.409109107	0.049813645

ST_7	DE_mRNA_total_index
mRNA_gene_symbol	mRNA_acc
MYBPH	ENSG00000133055
FOLR3	ENSG00000110203
MOCS1	ENSG00000124615
PI3	ENSG00000124102
FFAR2	ENSG00000126262
HEY2	ENSG00000135547
PPIAL4C	ENSG00000263464
TMEM171	ENSG00000157111
LRRN3	ENSG00000173114
FLRT1	ENSG00000126500
CX3CR1	ENSG00000168329
KLHL34	ENSG00000185915
TNFAIP6	ENSG00000123610
VIT	ENSG00000205221
GPR15	ENSG00000154165
H2BC18	ENSG00000203814
CISH	ENSG00000114737
JAM2	ENSG00000154721
CHIT1	ENSG00000133063
ZNF835	ENSG00000127903
CACNA1E	ENSG00000198216
SLC23A3	ENSG00000213901
AHRR	ENSG00000063438
IFIT2	ENSG00000119922
ZNF404	ENSG00000176222
NPM2	ENSG00000158806
IFIT3	ENSG00000119917
TPO	ENSG00000115705
LINGO2	ENSG00000174482
NHLRC4	ENSG00000257108
ZNF626	ENSG00000188171
ZNF547	ENSG00000152433
CD180	ENSG00000134061
BEND5	ENSG00000162373
CALHM1	ENSG00000185933
GCSAM	ENSG00000174500
VPREB3	ENSG00000128218
TLR10	ENSG00000174123
ZNF781	ENSG00000196381
PARS2	ENSG00000162396
ADAMTS1	ENSG00000154734
SMAD9	ENSG00000120693
ZNF285	ENSG00000267508
PRICKLE2	ENSG00000163637
PAQR8	ENSG00000170915

TMEM169	ENSG00000163449
DEGS2	ENSG00000168350
CCDC121	ENSG00000176714
ESM1	ENSG00000164283
GIMAP7	ENSG00000179144
AVPR1A	ENSG00000166148
ZNF613	ENSG00000176024
TMEM170B	ENSG00000205269
PRR7	ENSG00000131188
ZNF628	ENSG00000197483
ERMN	ENSG00000136541
CDKN1C	ENSG00000129757
C3orf35	ENSG00000198590
CKAP2L	ENSG00000169607
P4HA2	ENSG00000072682
APOE	ENSG00000130203
B3GNT4	ENSG00000176383
SPRED3	ENSG00000188766
PDK4	ENSG00000004799
RGPD6	ENSG00000183054
FEM1C	ENSG00000145780
TNFSF11	ENSG00000120659
IL13	ENSG00000169194
EREG	ENSG00000124882
NAT8L	ENSG00000185818
ATF3	ENSG00000162772
FNDC7	ENSG00000143107
PIK3R1	ENSG00000145675
SEC14L2	ENSG00000100003
TIPARP	ENSG00000163659
IFITM10	ENSG00000244242
YOD1	ENSG00000180667
CNDP1	ENSG00000150656
C15orf48	ENSG00000166920
CCDC74A	ENSG00000163040
ANKRD18A	ENSG00000180071
SPAG4	ENSG00000061656
GRIN2C	ENSG00000161509
PER1	ENSG00000179094
PIM3	ENSG00000198355
HSPA12B	ENSG00000132622
RND1	ENSG00000172602
SYN1	ENSG00000008056
SIAH1	ENSG00000196470
TNFAIP3	ENSG00000118503
RASGEF1B	ENSG00000138670
JUND	ENSG00000130522

SLC7A5	ENSG00000103257
SCARF2	ENSG00000244486
HIF1A	ENSG00000100644
CD83	ENSG00000112149
ZBTB21	ENSG00000173276
LRRC32	ENSG00000137507
PRLR	ENSG00000113494
ENC1	ENSG00000171617
RHBDF1	ENSG00000007384
GFPT2	ENSG00000131459
PDXP	ENSG00000241360
HIC1	ENSG00000177374
GRIN1	ENSG00000176884
PPP1R1A	ENSG00000135447
PDE4D	ENSG00000113448
LRRC70	ENSG00000186105
BTG3	ENSG00000154640
TEX12	ENSG00000150783
ZBTB10	ENSG00000205189
CNIH2	ENSG00000174871
ID1	ENSG00000125968
FTH1	ENSG00000167996
ENSG00000203392	ENSG00000203392
ENSG00000228804	ENSG00000228804
HTD2	ENSG00000255154
SIK1	ENSG00000142178
TMEM160	ENSG00000130748
RHBDL1	ENSG00000103269
ENSG00000250644	ENSG00000250644
REL	ENSG00000162924
RORB	ENSG00000198963
U2AF1	ENSG00000160201
TTC9B	ENSG00000174521
FAM81B	ENSG00000153347
IER5L	ENSG00000188483
SLC1A2	ENSG00000110436
NDUFV2	ENSG00000178127
TMC3	ENSG00000188869
B3GNT7	ENSG00000156966
HES1	ENSG00000114315
GRASP	ENSG00000161835
MYO3B	ENSG00000071909
PDF	ENSG00000258429
KIR3DX1	ENSG00000104970
MTSS2	ENSG00000132613
NR4A2	ENSG00000153234
NRARP	ENSG00000198435

SMAD6	ENSG00000137834
SLC36A3	ENSG00000186334
TNFRSF12A	ENSG00000006327
FAM217A	ENSG00000145975
FAM183BP	ENSG00000164556
SH3D19	ENSG00000109686
CCIN	ENSG00000185972
DUSP8	ENSG00000184545
TCTE1	ENSG00000146221
NPTX2	ENSG00000106236
TKTL2	ENSG00000151005
ATP1B2	ENSG00000129244
GALNT5	ENSG00000136542
LMOD2	ENSG00000170807
SKOR1	ENSG00000188779
HES4	ENSG00000188290
RNF152	ENSG00000176641
ARHGAP8	ENSG00000241484
CCDC173	ENSG00000154479
UTF1	ENSG00000171794
TMEM238	ENSG00000233493
TEX45	ENSG00000198723
TEKT2	ENSG00000092850
C4orf48	ENSG00000243449
NR4A3	ENSG00000119508
SLC12A1	ENSG00000074803
DEFA1	ENSG00000206047
ZMAT4	ENSG00000165061
DOC2B	ENSG00000272636
GTSCR1	ENSG00000263417
PRECSIT	ENSG00000255874
CHRNB2	ENSG00000160716
MCOLN3	ENSG00000055732
NETO1	ENSG00000166342
TOM1L1	ENSG00000141198
ZNF135	ENSG00000176293
NIPAL4	ENSG00000172548
SORCS3	ENSG00000156395
NEXMIF	ENSG00000050030
RASGRF1	ENSG00000058335
CD24	ENSG00000272398
COMTD1	ENSG00000165644
ATP5F1D	ENSG00000099624
MZB1	ENSG00000170476
MRPL12	ENSG00000262814
ZNF703	ENSG00000183779
GLTPD2	ENSG00000182327

SFN	ENSG00000175793
IDO1	ENSG00000131203
ACTL10	ENSG00000182584
H2AX	ENSG00000188486
CLEC11A	ENSG00000105472
ZNF579	ENSG00000218891
BUB1	ENSG00000169679
SCX	ENSG00000260428
HJURP	ENSG00000123485
CHST7	ENSG00000147119
KLF2	ENSG00000127528
ENSG00000258130	ENSG00000258130
KIF18B	ENSG00000186185
INAFM1	ENSG00000257704
CACNG6	ENSG00000130433
UBE2C	ENSG00000175063
ISL2	ENSG00000159556
NDUFAF8	ENSG00000224877
FZD7	ENSG00000155760
PKMYT1	ENSG00000127564
JSRP1	ENSG00000167476
DOCK6	ENSG00000130158
CFD	ENSG00000197766
ABCB9	ENSG00000150967
ADAT3	ENSG00000213638
IGLL5	ENSG00000254709
MBLAC1	ENSG00000214309
ANGPTL6	ENSG00000130812
MMP23B	ENSG00000189409
DLGAP5	ENSG00000126787
E2F8	ENSG00000129173
SKA3	ENSG00000165480
OXCT2	ENSG00000198754
ANTKMT	ENSG00000103254
CENPA	ENSG00000115163
MIXL1	ENSG00000185155
CTU1	ENSG00000142544
BIRC5	ENSG00000089685
CHCHD10	ENSG00000250479
TXNDC5	ENSG00000239264
C2CD4D	ENSG00000225556
CCDC85B	ENSG00000175602
SPC25	ENSG00000152253
DIAPH3	ENSG00000139734
DDT	ENSG00000099977
ADRB1	ENSG00000043591
ASCL2	ENSG00000183734

DEPDC1	ENSG00000024526
TNFRSF17	ENSG00000048462
H1-10	ENSG00000184897
RAB5IF	ENSG00000101084
RHOF	ENSG00000139725
EVA1B	ENSG00000142694
CITED4	ENSG00000179862
SOCS1	ENSG00000185338
ZNF804A	ENSG00000170396
CEBPD	ENSG00000221869
CEBPB	ENSG00000172216
SHISA8	ENSG00000234965
NEK2	ENSG00000117650
FA2H	ENSG00000103089
BHLHA15	ENSG00000180535
PCSK1N	ENSG00000102109
TNNT1	ENSG00000105048
ENDOG	ENSG00000167136
CDC20	ENSG00000117399
ARL2BP	ENSG00000102931
NT5DC4	ENSG00000144130
C17orf97	ENSG00000187624
APOBEC3B	ENSG00000179750
ROR2	ENSG00000169071
MET	ENSG00000105976
IGFBP2	ENSG00000115457
ENSG00000258653	ENSG00000258653
GRIK4	ENSG00000149403
RHCE	ENSG00000188672
NLRP9	ENSG00000185792
GRM3	ENSG00000198822
STEAP1B	ENSG00000105889
C9orf131	ENSG00000174038
SIGLEC1	ENSG00000088827
ZBED2	ENSG00000177494
GJB7	ENSG00000164411
PRG2	ENSG00000186652
COL1A1	ENSG00000108821
RANBP17	ENSG00000204764
PRH1	ENSG00000231887
ZNF483	ENSG00000173258
TENM1	ENSG00000009694
IL1RL1	ENSG00000115602
OTOF	ENSG00000115155
MATN1	ENSG00000162510
DDX47	ENSG00000213782
ZNF805	ENSG00000204524

ZNF14	ENSG00000105708
ASB12	ENSG00000198881
ENSG00000257545	ENSG00000257545
RGPD5	ENSG00000015568
RGS20	ENSG00000147509
ARHGAP39	ENSG00000147799
LINC01619	ENSG00000257242
CA14	ENSG00000118298
C4orf19	ENSG00000154274
MYBPC2	ENSG00000086967
GRAPL	ENSG00000189152
CCR6	ENSG00000112486
ENSG00000227782	ENSG00000227782
RBM44	ENSG00000177483
YES1	ENSG00000176105
KLHL15	ENSG00000174010
ICOSLG	ENSG00000160223
RBM11	ENSG00000185272
ZNF331	ENSG00000130844
CEMIP2	ENSG00000135048
CCNT1	ENSG00000129315
RBM34	ENSG00000188739
CCDC85C	ENSG00000205476
PPP1R9A	ENSG00000158528
ARL5B	ENSG00000165997
EML5	ENSG00000165521
SDE2	ENSG00000143751
CFL2	ENSG00000165410
SLC26A5	ENSG00000170615
SRGAP1	ENSG00000196935
RBFOX3	ENSG00000167281
ENSG00000269570	ENSG00000269570
ZBTB43	ENSG00000169155
PGAP1	ENSG00000197121
SCN3B	ENSG00000166257
PIGA	ENSG00000165195
ZBED6	ENSG00000257315
ZNF484	ENSG00000127081
ENSG00000152454	ENSG00000152454
NR1D2	ENSG00000174738
NCR3LG1	ENSG00000188211
RLIM	ENSG00000131263
SERTAD2	ENSG00000179833
PFKFB3	ENSG00000170525
SC5D	ENSG00000109929
RFX8	ENSG00000196460
CHD1	ENSG00000153922

DBF4	ENSG00000006634
IGFALS	ENSG00000099769
ARHGAP29	ENSG00000137962
ENSG00000262180	ENSG00000262180
MASTL	ENSG00000120539
ACKR4	ENSG00000129048
GCSAML	ENSG00000169224
RNF103	ENSG00000239305
C12orf71	ENSG00000214700
TMEM255A	ENSG00000125355
SOX5	ENSG00000134532
SYNM	ENSG00000182253
ERRFI1	ENSG00000116285
CCSER1	ENSG00000184305
TNFSF9	ENSG00000125657
GOLGA8M	ENSG00000188626
SCML1	ENSG00000047634
PKD2L2	ENSG00000078795
HBQ1	ENSG00000086506
SEMA3G	ENSG00000010319
MON1A	ENSG00000164077
FCRLB	ENSG00000162746
STBD1	ENSG00000118804
FAM43A	ENSG00000185112
EFNA3	ENSG00000143590
TMEM204	ENSG00000131634
FKBPL	ENSG00000204315
GIMAP1	ENSG00000213203
TREML2	ENSG00000112195
TICRR	ENSG00000140534
C14orf119	ENSG00000179933
LRFN1	ENSG00000128011
MYBL2	ENSG00000101057
COL9A3	ENSG00000092758
GPR68	ENSG00000119714
HSPA6	ENSG00000173110
NHLRC1	ENSG00000187566
ZNF837	ENSG00000152475
TNFRSF9	ENSG00000049249
BOLA1	ENSG00000178096
TM4SF19	ENSG00000145107
MCM10	ENSG00000065328
ADGRE3	ENSG00000131355
PYCR3	ENSG00000104524
LRRIQ3	ENSG00000162620
RPP25L	ENSG00000164967
RTN4R	ENSG00000040608

CAV1	ENSG00000105974
BORCS6	ENSG00000196544
BTN1A1	ENSG00000124557
B3GNT8	ENSG00000177191
DUSP1	ENSG00000120129
FCGR1CP	ENSG00000265531
SOWAHD	ENSG00000187808
OSM	ENSG00000099985
CEP19	ENSG00000174007
ENSG00000258674	ENSG00000258674
AURKA	ENSG00000087586
TMEM250	ENSG00000238227
FASLG	ENSG00000117560
GJA3	ENSG00000121743
DGAT2	ENSG00000062282
TNFAIP8L2	ENSG00000163154
NLRP6	ENSG00000174885
SLC22A1	ENSG00000175003
ZNF696	ENSG00000185730
FCGR1B	ENSG00000198019
FAM50B	ENSG00000145945
CILP	ENSG00000138615
ASB2	ENSG00000100628
S1PR4	ENSG00000125910
CDC42EP2	ENSG00000149798
HSPA1A	ENSG00000204389
KISS1R	ENSG00000116014
FPR3	ENSG00000187474
SSPN	ENSG00000123096
S100A3	ENSG00000188015
KIAA1324	ENSG00000116299
RHOB	ENSG00000143878
FUT7	ENSG00000180549
JCHAIN	ENSG00000132465
H2AC18	ENSG00000203812
BATF2	ENSG00000168062
PANX2	ENSG00000073150
FBLN5	ENSG00000140092
XCR1	ENSG00000173578
CCN3	ENSG00000136999
STEAP4	ENSG00000127954
AOC2	ENSG00000131480
KCNJ2	ENSG00000123700
FOS	ENSG00000170345
MMP25	ENSG00000008516
AOC3	ENSG00000131471
PYCR1	ENSG00000183010

LRG1	ENSG00000171236
CHI3L1	ENSG00000133048
ENSG00000253633	ENSG00000253633
DHRS9	ENSG00000073737
GGT5	ENSG00000099998
ARHGEF10	ENSG00000104728
GPRC5D	ENSG00000111291
SDC1	ENSG00000115884
MANSC1	ENSG00000111261
GLDC	ENSG00000178445
TNFRSF10C	ENSG00000173535
CXCL8	ENSG00000169429
SRXN1	ENSG00000271303
SMIM11A	ENSG00000205670
ENSG00000268170	ENSG00000268170
GDF10	ENSG00000266524
OTX1	ENSG00000115507
KRT23	ENSG00000108244
SPP1	ENSG00000118785
BTBD17	ENSG00000204347
AOC1	ENSG00000002726

ST_8

Total DE miRNA index
hsa-miR-101-3p
hsa-miR-877-5p
hsa-miR-124-5p
hsa-miR-664b-3p
hsa-miR-129-5p
hsa-miR-1291
hsa-miR-3605-5p
hsa-miR-619-5p
hsa-miR-548ah-3p
hsa-miR-1234-3p
hsa-miR-3064-5p
hsa-miR-218-5p
hsa-miR-513b-5p
hsa-miR-206
hsa-miR-4482-5p
hsa-miR-204-5p
hsa-miR-6837-3p
hsa-miR-219a-5p
hsa-miR-548x-5p
hsa-miR-4661-5p
hsa-miR-99b-5p
hsa-miR-625-5p
hsa-miR-10a-5p
hsa-miR-92b-5p
hsa-miR-942-5p
hsa-miR-501-5p
hsa-miR-21-5p
hsa-miR-98-5p
hsa-miR-19a-5p
hsa-miR-15b-5p
hsa-miR-502-5p
hsa-miR-199b-5p
hsa-miR-651-5p
hsa-miR-454-5p
hsa-miR-4645-5p
hsa-miR-103a-2-5p
hsa-miR-103a-3p
hsa-miR-500a-5p
hsa-miR-1249-5p
hsa-miR-628-5p
hsa-miR-574-5p
hsa-miR-20a-5p
hsa-miR-93-5p
hsa-miR-32-5p
hsa-miR-374a-5p

hsa-miR-190b
hsa-miR-362-5p
hsa-miR-17-5p
hsa-miR-6513-5p
hsa-miR-660-5p
hsa-miR-641
hsa-miR-196a-5p
hsa-miR-556-5p
hsa-miR-340-5p
hsa-miR-590-5p
hsa-miR-7854-3p
hsa-miR-1537-5p
hsa-miR-3176
hsa-miR-624-5p
hsa-miR-188-5p
hsa-miR-3613-5p
hsa-miR-190a-5p
hsa-miR-144-5p
hsa-miR-935
hsa-miR-580-5p
hsa-miR-301b-5p
hsa-miR-616-5p
hsa-miR-4775
hsa-miR-371b-5p
hsa-miR-4709-5p
hsa-miR-1262
hsa-miR-1179
hsa-miR-4802-5p
hsa-miR-1228-5p
hsa-miR-4632-5p
hsa-miR-4474-5p
hsa-miR-511-5p
hsa-miR-1303
hsa-miR-6770-5p
hsa-miR-4717-5p
hsa-miR-4425
hsa-miR-6837-5p
hsa-miR-1908-5p
hsa-miR-548s
hsa-miR-708-5p
hsa-miR-1299
hsa-miR-889-5p
hsa-miR-544b
hsa-miR-3611
hsa-miR-122-5p
hsa-miR-548ai
hsa-miR-539-5p

hsa-miR-5684
hsa-miR-548a-5p
hsa-miR-758-5p
hsa-miR-147b
hsa-miR-506-5p
hsa-miR-187-5p
hsa-miR-548ah-5p
hsa-miR-1256
hsa-miR-6502-5p
hsa-miR-943
hsa-miR-490-5p
hsa-miR-552-5p
hsa-miR-153-3p
hsa-miR-125b-5p
hsa-miR-9-5p
hsa-miR-30c-5p
hsa-miR-3074-5p
hsa-miR-365a-5p
hsa-miR-365b-5p
hsa-miR-24-1-5p
hsa-miR-193a-5p
hsa-miR-597-5p
hsa-miR-885-5p
hsa-miR-6737-5p
hsa-miR-6733-5p
hsa-miR-6734-5p
hsa-miR-5680
hsa-miR-5582-5p
hsa-miR-3065-5p
hsa-miR-4423-5p
hsa-miR-4491
hsa-miR-410-5p
hsa-miR-3179
hsa-miR-4999-5p
hsa-miR-1972
hsa-miR-376a-2-5p
hsa-miR-4781-5p
hsa-miR-3180-5p
hsa-miR-548ba
hsa-miR-6812-5p
hsa-miR-6515-5p
hsa-miR-4659b-5p
hsa-miR-876-5p
hsa-miR-4485-5p
hsa-miR-3945
hsa-miR-193b-3p
hsa-miR-3684

hsa-miR-513a-5p
hsa-miR-7852-3p
hsa-miR-7110-5p
hsa-miR-551b-5p
hsa-miR-4424
hsa-miR-4677-5p
hsa-miR-4772-5p
hsa-miR-432-5p
hsa-miR-504-5p
hsa-miR-449a
hsa-miR-4494
hsa-miR-3143
hsa-miR-5695
hsa-miR-4724-5p
hsa-miR-3144-5p
hsa-miR-4433a-5p
hsa-miR-3150a-5p
hsa-miR-4746-5p
hsa-miR-6813-5p

ST_9	total_miRNA/mRNA_pairs		
miRNA	mRNA_gene_symbol	mRNA_acc	pairScore
hsa-miR-101-3p	BIRC5	ENSG00000089685	1
hsa-miR-101-3p	DUSP1	ENSG00000120129	1
hsa-miR-101-3p	GFPT2	ENSG00000131459	1
hsa-miR-101-3p	ZBTB21	ENSG00000173276	1
hsa-miR-101-3p	TMEM170B	ENSG00000205269	1
hsa-miR-101-3p	RNF152	ENSG00000176641	1
hsa-miR-101-3p	MET	ENSG00000105976	1
hsa-miR-101-3p	REL	ENSG00000162924	1
hsa-miR-103a-2-5p	CEBPD	ENSG00000221869	1
hsa-miR-103a-2-5p	EREG	ENSG00000124882	1
hsa-miR-103a-3p	CCNT1	ENSG00000129315	1
hsa-miR-103a-3p	PIK3R1	ENSG00000145675	1
hsa-miR-103a-3p	CAV1	ENSG00000105974	1
hsa-miR-103a-3p	PER1	ENSG00000179094	1
hsa-miR-103a-3p	FEM1C	ENSG00000145780	1
hsa-miR-103a-3p	PDE4D	ENSG00000113448	1
hsa-miR-103a-3p	REL	ENSG00000162924	1
hsa-miR-103a-3p	CD180	ENSG00000134061	1
hsa-miR-103a-3p	ZBTB10	ENSG00000205189	1
hsa-miR-103a-3p	PDK4	ENSG00000004799	1
hsa-miR-103a-3p	TMEM255A	ENSG00000125355	1
hsa-miR-10a-5p	RLIM	ENSG00000131263	1
hsa-miR-10a-5p	YES1	ENSG00000176105	1
hsa-miR-10a-5p	LRFN1	ENSG00000128011	1
hsa-miR-10a-5p	ZBTB10	ENSG00000205189	1
hsa-miR-10a-5p	ERMN	ENSG00000136541	1
hsa-miR-10a-5p	BIRC5	ENSG00000089685	1
hsa-miR-10a-5p	NR1D2	ENSG00000174738	1
hsa-miR-10a-5p	YOD1	ENSG00000180667	1
hsa-miR-1179	ATF3	ENSG00000162772	1
hsa-miR-1179	ARHGAP39	ENSG00000147799	1
hsa-miR-1179	ARL5B	ENSG00000165997	1
hsa-miR-122-5p	TMEM250	ENSG00000238227	1
hsa-miR-122-5p	REL	ENSG00000162924	1
hsa-miR-122-5p	SOCS1	ENSG00000185338	1
hsa-miR-122-5p	BIRC5	ENSG00000089685	1
hsa-miR-122-5p	CLEC11A	ENSG00000105472	1
hsa-miR-122-5p	CD83	ENSG00000112149	1
hsa-miR-122-5p	CNDP1	ENSG00000150656	1
hsa-miR-122-5p	BATF2	ENSG00000168062	1
hsa-miR-122-5p	MASTL	ENSG00000120539	1
hsa-miR-122-5p	SLC7A5	ENSG00000103257	1
hsa-miR-122-5p	CCR6	ENSG00000112486	1
hsa-miR-1228-3p	KLF2	ENSG00000127528	1
hsa-miR-1228-3p	TCTE1	ENSG00000146221	1

hsa-miR-1228-3p	CEBPD	ENSG00000221869	1
hsa-miR-1228-3p	PER1	ENSG00000179094	1
hsa-miR-1234-3p	FOS	ENSG00000170345	1
hsa-miR-1234-3p	ZBTB43	ENSG00000169155	1
hsa-miR-124-3p	AHRR	ENSG00000063438	1
hsa-miR-124-3p	ZNF483	ENSG00000173258	1
hsa-miR-124-3p	ADAMTS1	ENSG00000154734	1
hsa-miR-124-3p	AURKA	ENSG00000087586	1
hsa-miR-124-3p	COL1A1	ENSG00000108821	1
hsa-miR-124-3p	ID1	ENSG00000125968	1
hsa-miR-124-3p	IFIT3	ENSG00000119917	1
hsa-miR-124-3p	EREG	ENSG00000124882	1
hsa-miR-124-3p	TOM1L1	ENSG00000141198	1
hsa-miR-124-3p	GFPT2	ENSG00000131459	1
hsa-miR-124-3p	ZNF626	ENSG00000188171	1
hsa-miR-124-3p	GCSAML	ENSG00000169224	1
hsa-miR-124-3p	TNFRSF12A	ENSG00000006327	1
hsa-miR-124-3p	VIT	ENSG00000205221	1
hsa-miR-124-3p	SDE2	ENSG00000143751	1
hsa-miR-124-3p	SC5D	ENSG00000109929	1
hsa-miR-124-3p	DGAT2	ENSG00000062282	1
hsa-miR-124-3p	CAV1	ENSG00000105974	1
hsa-miR-124-3p	DEPDC1	ENSG00000024526	1
hsa-miR-124-3p	RHBDF1	ENSG00000007384	1
hsa-miR-124-3p	ARHGAP29	ENSG00000137962	1
hsa-miR-124-3p	KLF2	ENSG00000127528	1
hsa-miR-124-3p	CCDC121	ENSG00000176714	1
hsa-miR-124-3p	BTG3	ENSG00000154640	1
hsa-miR-124-3p	SERTAD2	ENSG00000179833	1
hsa-miR-124-3p	NR4A3	ENSG00000119508	1
hsa-miR-124-3p	ARL5B	ENSG00000165997	1
hsa-miR-124-3p	GDF10	ENSG00000266524	1
hsa-miR-124-3p	RPP25L	ENSG00000164967	1
hsa-miR-124-3p	IFIT2	ENSG00000119922	1
hsa-miR-124-3p	CXCL8	ENSG00000169429	1
hsa-miR-1249-3p	PRLR	ENSG00000113494	1
hsa-miR-1249-5p	ARL5B	ENSG00000165997	1
hsa-miR-1249-5p	SLC7A5	ENSG00000103257	1
hsa-miR-125b-5p	TNFAIP3	ENSG00000118503	1
hsa-miR-125b-5p	YOD1	ENSG00000180667	1
hsa-miR-125b-5p	GJB7	ENSG00000164411	1
hsa-miR-125b-5p	NRARP	ENSG00000198435	1
hsa-miR-125b-5p	AHRR	ENSG00000063438	1
hsa-miR-125b-5p	ZNF483	ENSG00000173258	1
hsa-miR-129-5p	KLHL15	ENSG00000174010	1
hsa-miR-129-5p	DUSP1	ENSG00000120129	1
hsa-miR-129-5p	CCR6	ENSG00000112486	1

hsa-miR-129-5p	YES1	ENSG00000176105	1
hsa-miR-129-5p	ZNF285	ENSG00000267508	1
hsa-miR-129-5p	ICOSLG	ENSG00000160223	1
hsa-miR-129-5p	ZNF703	ENSG00000183779	1
hsa-miR-129-5p	CKAP2L	ENSG00000169607	1
hsa-miR-1299	KLHL15	ENSG00000174010	1
hsa-miR-1299	YOD1	ENSG00000180667	1
hsa-miR-1303	MET	ENSG00000105976	1
hsa-miR-1303	NLRP9	ENSG00000185792	1
hsa-miR-1303	KIR3DX1	ENSG00000104970	1
hsa-miR-1303	YOD1	ENSG00000180667	1
hsa-miR-144-3p	ARL5B	ENSG00000165997	1
hsa-miR-144-3p	MET	ENSG00000105976	1
hsa-miR-144-3p	YOD1	ENSG00000180667	1
hsa-miR-144-5p	PIGA	ENSG00000165195	1
hsa-miR-153-3p	ZBTB43	ENSG00000169155	1
hsa-miR-153-3p	ZNF703	ENSG00000183779	1
hsa-miR-153-3p	FEM1C	ENSG00000145780	1
hsa-miR-15a-5p	TNFSF9	ENSG00000125657	1
hsa-miR-15a-5p	SLC7A5	ENSG00000103257	1
hsa-miR-15a-5p	PRICKLE2	ENSG00000163637	1
hsa-miR-15a-5p	PDE4D	ENSG00000113448	1
hsa-miR-15a-5p	HSPA1A	ENSG00000204389	1
hsa-miR-15a-5p	CD180	ENSG00000134061	1
hsa-miR-15a-5p	CCNT1	ENSG00000129315	1
hsa-miR-15a-5p	SOX5	ENSG00000134532	1
hsa-miR-15a-5p	KLHL15	ENSG00000174010	1
hsa-miR-15a-5p	REL	ENSG00000162924	1
hsa-miR-15a-5p	SIK1	ENSG00000142178	1
hsa-miR-15a-5p	RGPD5	ENSG00000015568	1
hsa-miR-15a-5p	ZBTB10	ENSG00000205189	1
hsa-miR-15a-5p	PIK3R1	ENSG00000145675	1
hsa-miR-15b-5p	TNFSF9	ENSG00000125657	1
hsa-miR-15b-5p	KLHL15	ENSG00000174010	1
hsa-miR-15b-5p	SIK1	ENSG00000142178	1
hsa-miR-15b-5p	REL	ENSG00000162924	1
hsa-miR-15b-5p	RLIM	ENSG00000131263	1
hsa-miR-15b-5p	CD180	ENSG00000134061	1
hsa-miR-15b-5p	PDE4D	ENSG00000113448	1
hsa-miR-15b-5p	PIK3R1	ENSG00000145675	1
hsa-miR-15b-5p	CCNT1	ENSG00000129315	1
hsa-miR-15b-5p	ZBTB10	ENSG00000205189	1
hsa-miR-15b-5p	PRICKLE2	ENSG00000163637	1
hsa-miR-15b-5p	SLC7A5	ENSG00000103257	1
hsa-miR-17-5p	SMAD6	ENSG00000137834	1
hsa-miR-17-5p	C14orf119	ENSG00000179933	1
hsa-miR-17-5p	YOD1	ENSG00000180667	1

hsa-miR-17-5p	YES1	ENSG00000176105	1
hsa-miR-17-5p	HIF1A	ENSG00000100644	1
hsa-miR-17-5p	SIK1	ENSG00000142178	1
hsa-miR-17-5p	NAT8L	ENSG00000185818	1
hsa-miR-17-5p	BTG3	ENSG00000154640	1
hsa-miR-17-5p	CFL2	ENSG00000165410	1
hsa-miR-17-5p	RLIM	ENSG00000131263	1
hsa-miR-17-5p	ATF3	ENSG00000162772	1
hsa-miR-17-5p	UBE2C	ENSG00000175063	1
hsa-miR-17-5p	PER1	ENSG00000179094	1
hsa-miR-17-5p	KLHL15	ENSG00000174010	1
hsa-miR-17-5p	DEPDC1	ENSG00000024526	1
hsa-miR-17-5p	MASTL	ENSG00000120539	1
hsa-miR-17-5p	MIXL1	ENSG00000185155	1
hsa-miR-17-5p	PKMYT1	ENSG00000127564	1
hsa-miR-17-5p	FEM1C	ENSG00000145780	1
hsa-miR-17-5p	ZNF805	ENSG00000204524	1
hsa-miR-17-5p	FTH1	ENSG00000167996	1
hsa-miR-17-5p	CAV1	ENSG00000105974	1
hsa-miR-187-3p	CENPA	ENSG00000115163	1
hsa-miR-187-5p	FOS	ENSG00000170345	1
hsa-miR-187-5p	ZNF805	ENSG00000204524	1
hsa-miR-188-5p	TMEM170B	ENSG00000205269	1
hsa-miR-188-5p	CDC20	ENSG00000117399	1
hsa-miR-188-5p	ADRB1	ENSG00000043591	1
hsa-miR-188-5p	REL	ENSG00000162924	1
hsa-miR-193a-3p	BUB1	ENSG00000169679	1
hsa-miR-193a-3p	SLC7A5	ENSG00000103257	1
hsa-miR-193a-3p	AURKA	ENSG00000087586	1
hsa-miR-193b-3p	BUB1	ENSG00000169679	1
hsa-miR-193b-3p	CKAP2L	ENSG00000169607	1
hsa-miR-193b-3p	TICRR	ENSG00000140534	1
hsa-miR-193b-3p	CHCHD10	ENSG00000250479	1
hsa-miR-193b-3p	CDC20	ENSG00000117399	1
hsa-miR-193b-3p	NAT8L	ENSG00000185818	1
hsa-miR-193b-3p	NRARP	ENSG00000198435	1
hsa-miR-193b-3p	UBE2C	ENSG00000175063	1
hsa-miR-193b-3p	MCM10	ENSG00000065328	1
hsa-miR-193b-3p	SKA3	ENSG00000165480	1
hsa-miR-193b-3p	ZBTB43	ENSG00000169155	1
hsa-miR-193b-3p	TMEM204	ENSG00000131634	1
hsa-miR-193b-3p	SLC7A5	ENSG00000103257	1
hsa-miR-193b-3p	APOBEC3B	ENSG00000179750	1
hsa-miR-193b-3p	PKMYT1	ENSG00000127564	1
hsa-miR-193b-3p	ERRFI1	ENSG00000116285	1
hsa-miR-193b-3p	SPC25	ENSG00000152253	1
hsa-miR-196a-5p	KCNJ2	ENSG00000123700	1

hsa-miR-196a-5p	UBE2C	ENSG00000175063	1
hsa-miR-196a-5p	BUB1	ENSG00000169679	1
hsa-miR-196a-5p	YOD1	ENSG00000180667	1
hsa-miR-196a-5p	KIF18B	ENSG00000186185	1
hsa-miR-196a-5p	GRIK4	ENSG00000149403	1
hsa-miR-196a-5p	CKAP2L	ENSG00000169607	1
hsa-miR-199a-5p	APOE	ENSG00000130203	1
hsa-miR-199a-5p	HIF1A	ENSG00000100644	1
hsa-miR-199a-5p	CAV1	ENSG00000105974	1
hsa-miR-199a-5p	RND1	ENSG00000172602	1
hsa-miR-199b-5p	HIF1A	ENSG00000100644	1
hsa-miR-199b-5p	HES1	ENSG00000114315	1
hsa-miR-19a-5p	RLIM	ENSG00000131263	1
hsa-miR-19a-5p	NR4A3	ENSG00000119508	1
hsa-miR-19a-5p	FOS	ENSG00000170345	1
hsa-miR-19a-5p	KLHL15	ENSG00000174010	1
hsa-miR-19a-5p	TNFAIP6	ENSG00000123610	1
hsa-miR-204-5p	ARHGAP29	ENSG00000137962	1
hsa-miR-204-5p	PRLR	ENSG00000113494	1
hsa-miR-204-5p	CXCL8	ENSG00000169429	1
hsa-miR-204-5p	STEAP4	ENSG00000127954	1
hsa-miR-204-5p	RORB	ENSG00000198963	1
hsa-miR-204-5p	SRGAP1	ENSG00000196935	1
hsa-miR-204-5p	ZBTB43	ENSG00000169155	1
hsa-miR-204-5p	PDF	ENSG00000258429	1
hsa-miR-204-5p	SMAD6	ENSG00000137834	1
hsa-miR-206	MET	ENSG00000105976	1
hsa-miR-206	KCNJ2	ENSG00000123700	1
hsa-miR-20a-5p	ZNF331	ENSG00000130844	1
hsa-miR-20a-5p	MIXL1	ENSG00000185155	1
hsa-miR-20a-5p	CAV1	ENSG00000105974	1
hsa-miR-20a-5p	YOD1	ENSG00000180667	1
hsa-miR-20a-5p	KLHL15	ENSG00000174010	1
hsa-miR-20a-5p	C14orf119	ENSG00000179933	1
hsa-miR-20a-5p	SIK1	ENSG00000142178	1
hsa-miR-20a-5p	MASTL	ENSG00000120539	1
hsa-miR-20a-5p	RLIM	ENSG00000131263	1
hsa-miR-20a-5p	BTG3	ENSG00000154640	1
hsa-miR-20a-5p	HIF1A	ENSG00000100644	1
hsa-miR-20a-5p	ZNF805	ENSG00000204524	1
hsa-miR-20a-5p	SMAD6	ENSG00000137834	1
hsa-miR-20a-5p	FEM1C	ENSG00000145780	1
hsa-miR-20a-5p	UBE2C	ENSG00000175063	1
hsa-miR-20a-5p	CFL2	ENSG00000165410	1
hsa-miR-20a-5p	PKMYT1	ENSG00000127564	1
hsa-miR-21-5p	PIK3R1	ENSG00000145675	1
hsa-miR-21-5p	YOD1	ENSG00000180667	1

hsa-miR-21-5p	SOX5	ENSG00000134532	1
hsa-miR-21-5p	RNF103	ENSG00000239305	1
hsa-miR-21-5p	DUSP8	ENSG00000184545	1
hsa-miR-21-5p	PRICKLE2	ENSG00000163637	1
hsa-miR-21-5p	HIF1A	ENSG00000100644	1
hsa-miR-21-5p	FASLG	ENSG00000117560	1
hsa-miR-21-5p	KLHL15	ENSG00000174010	1
hsa-miR-21-5p	RHOB	ENSG00000143878	1
hsa-miR-218-5p	CCDC74A	ENSG00000163040	1
hsa-miR-218-5p	ASB2	ENSG00000100628	1
hsa-miR-218-5p	SEMA3G	ENSG00000010319	1
hsa-miR-218-5p	BIRC5	ENSG00000089685	1
hsa-miR-218-5p	MET	ENSG00000105976	1
hsa-miR-218-5p	RHOB	ENSG00000143878	1
hsa-miR-218-5p	HJURP	ENSG00000123485	1
hsa-miR-218-5p	PRLR	ENSG00000113494	1
hsa-miR-218-5p	DGAT2	ENSG00000062282	1
hsa-miR-219a-5p	CEP19	ENSG00000174007	1
hsa-miR-24-1-5p	SLC23A3	ENSG00000213901	1
hsa-miR-24-3p	YOD1	ENSG00000180667	1
hsa-miR-24-3p	SCML1	ENSG00000047634	1
hsa-miR-24-3p	RHOF	ENSG00000139725	1
hsa-miR-24-3p	MCM10	ENSG00000065328	1
hsa-miR-24-3p	YES1	ENSG00000176105	1
hsa-miR-24-3p	DBF4	ENSG00000006634	1
hsa-miR-24-3p	PDF	ENSG00000258429	1
hsa-miR-24-3p	UBE2C	ENSG00000175063	1
hsa-miR-24-3p	SLC1A2	ENSG00000110436	1
hsa-miR-24-3p	AURKA	ENSG00000087586	1
hsa-miR-24-3p	KLHL15	ENSG00000174010	1
hsa-miR-24-3p	KIF18B	ENSG00000186185	1
hsa-miR-24-3p	TNFAIP3	ENSG00000118503	1
hsa-miR-24-3p	HBQ1	ENSG00000086506	1
hsa-miR-24-3p	DEPDC1	ENSG00000024526	1
hsa-miR-24-3p	ARHGAP39	ENSG00000147799	1
hsa-miR-24-3p	ATF3	ENSG00000162772	1
hsa-miR-301a-3p	DEPDC1	ENSG00000024526	1
hsa-miR-301a-3p	MASTL	ENSG00000120539	1
hsa-miR-301a-3p	CFL2	ENSG00000165410	1
hsa-miR-301a-3p	CCR6	ENSG00000112486	1
hsa-miR-301a-3p	SIK1	ENSG00000142178	1
hsa-miR-301a-3p	PIGA	ENSG00000165195	1
hsa-miR-301a-3p	RLIM	ENSG00000131263	1
hsa-miR-3074-3p	PER1	ENSG00000179094	1
hsa-miR-3074-3p	HIF1A	ENSG00000100644	1
hsa-miR-3074-3p	TMEM170B	ENSG00000205269	1
hsa-miR-30a-3p	ATF3	ENSG00000162772	1

hsa-miR-30a-3p	HSPA6	ENSG00000173110	1
hsa-miR-30a-3p	CACNA1E	ENSG00000198216	1
hsa-miR-30a-3p	SLC1A2	ENSG00000110436	1
hsa-miR-30a-3p	ARL2BP	ENSG00000102931	1
hsa-miR-30c-5p	BIRC5	ENSG00000089685	1
hsa-miR-30c-5p	SDE2	ENSG00000143751	1
hsa-miR-30c-5p	GCSAM	ENSG00000174500	1
hsa-miR-30c-5p	TXNDC5	ENSG00000239264	1
hsa-miR-30c-5p	CFL2	ENSG00000165410	1
hsa-miR-30c-5p	MYBL2	ENSG00000101057	1
hsa-miR-30c-5p	KLHL15	ENSG00000174010	1
hsa-miR-30c-5p	FAM81B	ENSG00000153347	1
hsa-miR-30c-5p	DBF4	ENSG00000006634	1
hsa-miR-30c-5p	SOCS1	ENSG00000185338	1
hsa-miR-30c-5p	SLC7A5	ENSG00000103257	1
hsa-miR-30c-5p	CHD1	ENSG00000153922	1
hsa-miR-3150a-3p	ICOSLG	ENSG00000160223	1
hsa-miR-3150a-3p	ASCL2	ENSG00000183734	1
hsa-miR-3150a-5p	PIM3	ENSG00000198355	1
hsa-miR-3176	SLC1A2	ENSG00000110436	1
hsa-miR-3180	ASCL2	ENSG00000183734	1
hsa-miR-3180	FAM43A	ENSG00000185112	1
hsa-miR-3180-5p	ATF3	ENSG00000162772	1
hsa-miR-3180-5p	TMEM169	ENSG00000163449	1
hsa-miR-3180-5p	ARL5B	ENSG00000165997	1
hsa-miR-32-5p	NLRP9	ENSG00000185792	1
hsa-miR-32-5p	RNF103	ENSG00000239305	1
hsa-miR-32-5p	REL	ENSG00000162924	1
hsa-miR-32-5p	GFPT2	ENSG00000131459	1
hsa-miR-32-5p	SERTAD2	ENSG00000179833	1
hsa-miR-32-5p	SMAD6	ENSG00000137834	1
hsa-miR-32-5p	SIK1	ENSG00000142178	1
hsa-miR-32-5p	AURKA	ENSG00000087586	1
hsa-miR-32-5p	KLHL15	ENSG00000174010	1
hsa-miR-32-5p	FASLG	ENSG00000117560	1
hsa-miR-340-3p	MRPL12	ENSG00000262814	1
hsa-miR-340-5p	SERTAD2	ENSG00000179833	1
hsa-miR-340-5p	ARL5B	ENSG00000165997	1
hsa-miR-340-5p	BTG3	ENSG00000154640	1
hsa-miR-340-5p	MET	ENSG00000105976	1
hsa-miR-340-5p	PDE4D	ENSG00000113448	1
hsa-miR-340-5p	BUB1	ENSG00000169679	1
hsa-miR-340-5p	KLHL15	ENSG00000174010	1
hsa-miR-340-5p	DEPDC1	ENSG00000024526	1
hsa-miR-340-5p	PIM3	ENSG00000198355	1
hsa-miR-3605-5p	MRPL12	ENSG00000262814	1
hsa-miR-3605-5p	DDT	ENSG00000099977	1

hsa-miR-3613-5p	NCR3LG1	ENSG00000188211	1
hsa-miR-362-5p	CCNT1	ENSG00000129315	1
hsa-miR-362-5p	CHD1	ENSG00000153922	1
hsa-miR-362-5p	ZBTB10	ENSG00000205189	1
hsa-miR-365a-3p	KLHL15	ENSG00000174010	1
hsa-miR-365a-3p	REL	ENSG00000162924	1
hsa-miR-365a-5p	SPRED3	ENSG00000188766	1
hsa-miR-365a-5p	MASTL	ENSG00000120539	1
hsa-miR-365b-5p	MASTL	ENSG00000120539	1
hsa-miR-365b-5p	SPRED3	ENSG00000188766	1
hsa-miR-371a-3p	SYNM	ENSG00000182253	1
hsa-miR-371b-5p	CEBPB	ENSG00000172216	1
hsa-miR-371b-5p	ZNF285	ENSG00000267508	1
hsa-miR-371b-5p	SH3D19	ENSG00000109686	1
hsa-miR-371b-5p	CCDC121	ENSG00000176714	1
hsa-miR-371b-5p	MCM10	ENSG00000065328	1
hsa-miR-371b-5p	KLF2	ENSG00000127528	1
hsa-miR-374a-5p	TNFSF9	ENSG00000125657	1
hsa-miR-374a-5p	TNFAIP3	ENSG00000118503	1
hsa-miR-374a-5p	CEBPB	ENSG00000172216	1
hsa-miR-374a-5p	AVPR1A	ENSG00000166148	1
hsa-miR-374a-5p	DUSP8	ENSG00000184545	1
hsa-miR-374a-5p	YOD1	ENSG00000180667	1
hsa-miR-374a-5p	SPC25	ENSG00000152253	1
hsa-miR-374a-5p	KLHL15	ENSG00000174010	1
hsa-miR-376a-2-5p	ZNF805	ENSG00000204524	1
hsa-miR-376a-2-5p	ARL5B	ENSG00000165997	1
hsa-miR-3945	DDX47	ENSG00000213782	1
hsa-miR-432-5p	CENPA	ENSG00000115163	1
hsa-miR-4423-5p	DEPDC1	ENSG00000024526	1
hsa-miR-4433a-3p	MIXL1	ENSG00000185155	1
hsa-miR-4433a-3p	MRPL12	ENSG00000262814	1
hsa-miR-4433a-3p	NLRP6	ENSG00000174885	1
hsa-miR-4433a-3p	ZNF805	ENSG00000204524	1
hsa-miR-4433a-3p	SRGAP1	ENSG00000196935	1
hsa-miR-4433a-3p	CEBPD	ENSG00000221869	1
hsa-miR-4433a-5p	REL	ENSG00000162924	1
hsa-miR-4433a-5p	KLHL15	ENSG00000174010	1
hsa-miR-4482-5p	SDE2	ENSG00000143751	1
hsa-miR-4482-5p	REL	ENSG00000162924	1
hsa-miR-4485-5p	CNDP1	ENSG00000150656	1
hsa-miR-4485-5p	SH3D19	ENSG00000109686	1
hsa-miR-4494	PIM3	ENSG00000198355	1
hsa-miR-4494	GIMAP1	ENSG00000213203	1
hsa-miR-449a	MET	ENSG00000105976	1
hsa-miR-454-3p	DEPDC1	ENSG00000024526	1
hsa-miR-454-3p	PIGA	ENSG00000165195	1

hsa-miR-454-3p	ZNF805	ENSG00000204524	1
hsa-miR-454-3p	MASTL	ENSG00000120539	1
hsa-miR-454-3p	CCR6	ENSG00000112486	1
hsa-miR-454-3p	SIK1	ENSG00000142178	1
hsa-miR-454-3p	CFL2	ENSG00000165410	1
hsa-miR-454-3p	RLIM	ENSG00000131263	1
hsa-miR-454-3p	KLHL15	ENSG00000174010	1
hsa-miR-454-5p	KLF2	ENSG00000127528	1
hsa-miR-4632-3p	NHLRC4	ENSG00000257108	1
hsa-miR-4632-5p	ZMAT4	ENSG00000165061	1
hsa-miR-4632-5p	AURKA	ENSG00000087586	1
hsa-miR-4645-5p	CEBPD	ENSG00000221869	1
hsa-miR-4645-5p	CAV1	ENSG00000105974	1
hsa-miR-4645-5p	TMEM170B	ENSG00000205269	1
hsa-miR-4659b-5p	NR4A3	ENSG00000119508	1
hsa-miR-4717-5p	KLHL15	ENSG00000174010	1
hsa-miR-4746-5p	PIGA	ENSG00000165195	1
hsa-miR-4775	CTU1	ENSG00000142544	1
hsa-miR-4775	GRIK4	ENSG00000149403	1
hsa-miR-4775	SSPN	ENSG00000123096	1
hsa-miR-490-3p	ICOSLG	ENSG00000160223	1
hsa-miR-490-5p	FOS	ENSG00000170345	1
hsa-miR-500a-3p	KLHL15	ENSG00000174010	1
hsa-miR-500a-3p	ZBTB43	ENSG00000169155	1
hsa-miR-500a-3p	MIXL1	ENSG00000185155	1
hsa-miR-500a-5p	SLC1A2	ENSG00000110436	1
hsa-miR-500a-5p	REL	ENSG00000162924	1
hsa-miR-500a-5p	CCNT1	ENSG00000129315	1
hsa-miR-500a-5p	CKAP2L	ENSG00000169607	1
hsa-miR-501-5p	CHD1	ENSG00000153922	1
hsa-miR-501-5p	REL	ENSG00000162924	1
hsa-miR-501-5p	ZMAT4	ENSG00000165061	1
hsa-miR-501-5p	SLC1A2	ENSG00000110436	1
hsa-miR-502-5p	SSPN	ENSG00000123096	1
hsa-miR-502-5p	BHLHA15	ENSG00000180535	1
hsa-miR-506-3p	ZNF626	ENSG00000188171	1
hsa-miR-506-3p	SDE2	ENSG00000143751	1
hsa-miR-513a-5p	RNF152	ENSG00000176641	1
hsa-miR-513a-5p	ARL5B	ENSG00000165997	1
hsa-miR-513a-5p	DDT	ENSG00000099977	1
hsa-miR-513a-5p	BTG3	ENSG00000154640	1
hsa-miR-513a-5p	TXNDC5	ENSG00000239264	1
hsa-miR-513a-5p	NR1D2	ENSG00000174738	1
hsa-miR-513b-5p	NCR3LG1	ENSG00000188211	1
hsa-miR-513b-5p	KLHL15	ENSG00000174010	1
hsa-miR-513b-5p	GIMAP7	ENSG00000179144	1
hsa-miR-513b-5p	BTG3	ENSG00000154640	1

hsa-miR-539-5p	ZNF579	ENSG00000218891	1
hsa-miR-539-5p	BUB1	ENSG00000169679	1
hsa-miR-548a-5p	YOD1	ENSG00000180667	1
hsa-miR-548a-5p	FEM1C	ENSG00000145780	1
hsa-miR-548a-5p	ZBTB43	ENSG00000169155	1
hsa-miR-548a-5p	SIK1	ENSG00000142178	1
hsa-miR-548a-5p	SPC25	ENSG00000152253	1
hsa-miR-548ah-3p	ZBTB10	ENSG00000205189	1
hsa-miR-548ah-3p	YES1	ENSG00000176105	1
hsa-miR-548ah-3p	RHOB	ENSG00000143878	1
hsa-miR-548ah-3p	KIAA1324	ENSG00000116299	1
hsa-miR-548ah-3p	GRIK4	ENSG00000149403	1
hsa-miR-548ah-3p	FEM1C	ENSG00000145780	1
hsa-miR-548ah-3p	SPC25	ENSG00000152253	1
hsa-miR-548ah-3p	TNFAIP3	ENSG00000118503	1
hsa-miR-548ah-3p	ICOSLG	ENSG00000160223	1
hsa-miR-548ah-5p	BTG3	ENSG00000154640	1
hsa-miR-548ah-5p	FEM1C	ENSG00000145780	1
hsa-miR-548ah-5p	ZNF805	ENSG00000204524	1
hsa-miR-548ah-5p	CAV1	ENSG00000105974	1
hsa-miR-548ah-5p	HIF1A	ENSG00000100644	1
hsa-miR-548ah-5p	RLIM	ENSG00000131263	1
hsa-miR-548ah-5p	DGAT2	ENSG00000062282	1
hsa-miR-548ah-5p	KLHL15	ENSG00000174010	1
hsa-miR-548ah-5p	PRICKLE2	ENSG00000163637	1
hsa-miR-548ah-5p	SIK1	ENSG00000142178	1
hsa-miR-548ai	FTH1	ENSG00000167996	1
hsa-miR-548ba	FTH1	ENSG00000167996	1
hsa-miR-548s	KLF2	ENSG00000127528	1
hsa-miR-548s	SOX5	ENSG00000134532	1
hsa-miR-548s	ZNF285	ENSG00000267508	1
hsa-miR-548s	MRPL12	ENSG00000262814	1
hsa-miR-548s	PDF	ENSG00000258429	1
hsa-miR-548x-5p	E2F8	ENSG00000129173	1
hsa-miR-548x-5p	KCNJ2	ENSG00000123700	1
hsa-miR-548x-5p	CFL2	ENSG00000165410	1
hsa-miR-548x-5p	AVPR1A	ENSG00000166148	1
hsa-miR-548x-5p	ADRB1	ENSG00000043591	1
hsa-miR-548x-5p	ARL5B	ENSG00000165997	1
hsa-miR-551b-5p	BUB1	ENSG00000169679	1
hsa-miR-551b-5p	YOD1	ENSG00000180667	1
hsa-miR-551b-5p	FTH1	ENSG00000167996	1
hsa-miR-551b-5p	SLC7A5	ENSG00000103257	1
hsa-miR-551b-5p	ZBTB10	ENSG00000205189	1
hsa-miR-556-5p	CAV1	ENSG00000105974	1
hsa-miR-574-5p	SLC7A5	ENSG00000103257	1
hsa-miR-574-5p	BUB1	ENSG00000169679	1

hsa-miR-574-5p	TCTE1	ENSG00000146221	1
hsa-miR-574-5p	TREML2	ENSG00000112195	1
hsa-miR-574-5p	RNF152	ENSG00000176641	1
hsa-miR-574-5p	SSPN	ENSG00000123096	1
hsa-miR-574-5p	SOX5	ENSG00000134532	1
hsa-miR-574-5p	GIMAP1	ENSG00000213203	1
hsa-miR-574-5p	E2F8	ENSG00000129173	1
hsa-miR-574-5p	SMAD9	ENSG00000120693	1
hsa-miR-574-5p	NEXMIF	ENSG00000050030	1
hsa-miR-590-5p	KLHL15	ENSG00000174010	1
hsa-miR-616-3p	SIK1	ENSG00000142178	1
hsa-miR-616-5p	KLF2	ENSG00000127528	1
hsa-miR-616-5p	CEBPB	ENSG00000172216	1
hsa-miR-616-5p	MCM10	ENSG00000065328	1
hsa-miR-616-5p	ZNF285	ENSG00000267508	1
hsa-miR-616-5p	SH3D19	ENSG00000109686	1
hsa-miR-616-5p	CCDC121	ENSG00000176714	1
hsa-miR-624-3p	ARL5B	ENSG00000165997	1
hsa-miR-625-5p	JUND	ENSG00000130522	1
hsa-miR-625-5p	CITED4	ENSG00000179862	1
hsa-miR-625-5p	EFNA3	ENSG00000143590	1
hsa-miR-651-5p	HSPA12B	ENSG00000132622	1
hsa-miR-6513-5p	MANSC1	ENSG00000111261	1
hsa-miR-6513-5p	ZNF703	ENSG00000183779	1
hsa-miR-6513-5p	RHOF	ENSG00000139725	1
hsa-miR-6513-5p	PDF	ENSG00000258429	1
hsa-miR-6513-5p	SLC1A2	ENSG00000110436	1
hsa-miR-664b-3p	ZNF331	ENSG00000130844	1
hsa-miR-664b-3p	TENM1	ENSG00000009694	1
hsa-miR-664b-3p	SYNM	ENSG00000182253	1
hsa-miR-708-5p	SLC7A5	ENSG00000103257	1
hsa-miR-708-5p	BIRC5	ENSG00000089685	1
hsa-miR-877-5p	YOD1	ENSG00000180667	1
hsa-miR-877-5p	HSPA12B	ENSG00000132622	1
hsa-miR-877-5p	TMEM170B	ENSG00000205269	1
hsa-miR-877-5p	DDX47	ENSG00000213782	1
hsa-miR-885-5p	BUB1	ENSG00000169679	1
hsa-miR-885-5p	SYNM	ENSG00000182253	1
hsa-miR-9-5p	KCNJ2	ENSG00000123700	1
hsa-miR-9-5p	ZMAT4	ENSG00000165061	1
hsa-miR-9-5p	ARHGEF10	ENSG00000104728	1
hsa-miR-9-5p	P4HA2	ENSG00000072682	1
hsa-miR-9-5p	SDC1	ENSG00000115884	1
hsa-miR-92a-3p	RNF103	ENSG00000239305	1
hsa-miR-92a-3p	FKBPL	ENSG00000204315	1
hsa-miR-92a-3p	SERTAD2	ENSG00000179833	1
hsa-miR-92a-3p	KLF2	ENSG00000127528	1

hsa-miR-92a-3p	HES1	ENSG00000114315	1
hsa-miR-92a-3p	ZNF703	ENSG00000183779	1
hsa-miR-92a-3p	ADAMTS1	ENSG00000154734	1
hsa-miR-92a-3p	SMAD6	ENSG00000137834	1
hsa-miR-92a-3p	KLHL15	ENSG00000174010	1
hsa-miR-92a-3p	NLRP9	ENSG00000185792	1
hsa-miR-92a-3p	SIK1	ENSG00000142178	1
hsa-miR-92a-3p	GFPT2	ENSG00000131459	1
hsa-miR-92a-3p	TOM1L1	ENSG00000141198	1
hsa-miR-92a-3p	TMEM160	ENSG00000130748	1
hsa-miR-92a-3p	NEK2	ENSG00000117650	1
hsa-miR-92a-3p	REL	ENSG00000162924	1
hsa-miR-92a-3p	AURKA	ENSG00000087586	1
hsa-miR-92a-3p	KIF18B	ENSG00000186185	1
hsa-miR-92a-3p	TLR10	ENSG00000174123	1
hsa-miR-92a-3p	CHCHD10	ENSG00000250479	1
hsa-miR-92a-3p	MYBL2	ENSG00000101057	1
hsa-miR-92a-3p	CDC20	ENSG00000117399	1
hsa-miR-92a-3p	FASLG	ENSG00000117560	1
hsa-miR-92b-5p	SRGAP1	ENSG00000196935	1
hsa-miR-92b-5p	DDT	ENSG00000099977	1
hsa-miR-92b-5p	ARHGAP39	ENSG00000147799	1
hsa-miR-92b-5p	SLC7A5	ENSG00000103257	1
hsa-miR-93-5p	FEM1C	ENSG00000145780	1
hsa-miR-93-5p	ZNF805	ENSG00000204524	1
hsa-miR-93-5p	HIF1A	ENSG00000100644	1
hsa-miR-93-5p	PKMYT1	ENSG00000127564	1
hsa-miR-93-5p	ZBTB21	ENSG00000173276	1
hsa-miR-93-5p	SERTAD2	ENSG00000179833	1
hsa-miR-93-5p	MASTL	ENSG00000120539	1
hsa-miR-93-5p	KLHL15	ENSG00000174010	1
hsa-miR-93-5p	EREG	ENSG00000124882	1
hsa-miR-93-5p	YOD1	ENSG00000180667	1
hsa-miR-93-5p	BIRC5	ENSG00000089685	1
hsa-miR-93-5p	CXCL8	ENSG00000169429	1
hsa-miR-93-5p	BTG3	ENSG00000154640	1
hsa-miR-93-5p	RLIM	ENSG00000131263	1
hsa-miR-93-5p	SMAD6	ENSG00000137834	1
hsa-miR-93-5p	MIXL1	ENSG00000185155	1
hsa-miR-93-5p	CFL2	ENSG00000165410	1
hsa-miR-93-5p	SIK1	ENSG00000142178	1
hsa-miR-93-5p	C14orf119	ENSG00000179933	1
hsa-miR-93-5p	CAV1	ENSG00000105974	1
hsa-miR-93-5p	DUSP8	ENSG00000184545	1
hsa-miR-98-5p	ZBTB10	ENSG00000205189	1
hsa-miR-98-5p	YOD1	ENSG00000180667	1
hsa-miR-98-5p	SOCS1	ENSG00000185338	1

hsa-miR-98-5p	CEBPD	ENSG00000221869	1
hsa-miR-98-5p	ICOSLG	ENSG00000160223	1
hsa-miR-98-5p	SERTAD2	ENSG00000179833	1
hsa-miR-98-5p	DBF4	ENSG00000006634	1
hsa-miR-98-5p	NAT8L	ENSG00000185818	1
hsa-miR-98-5p	REL	ENSG00000162924	1
hsa-miR-98-5p	TNFSF9	ENSG00000125657	1
hsa-miR-98-5p	IL13	ENSG00000169194	1
hsa-miR-98-5p	SEMA3G	ENSG00000010319	1
hsa-miR-98-5p	FAM43A	ENSG00000185112	1
hsa-miR-98-5p	CHD1	ENSG00000153922	1
hsa-miR-98-5p	DUSP1	ENSG00000120129	1
hsa-miR-98-5p	TNFRSF9	ENSG00000049249	1
hsa-miR-98-5p	MRPL12	ENSG00000262814	1
hsa-miR-99a-5p	STEAP4	ENSG00000127954	1
hsa-miR-101-3p	NR4A3	ENSG00000119508	0.725
hsa-miR-101-3p	PRLR	ENSG00000113494	0.985
hsa-miR-101-3p	NR1D2	ENSG00000174738	0.87
hsa-miR-101-3p	REL	ENSG00000162924	0.793
hsa-miR-101-3p	TENM1	ENSG00000009694	0.97
hsa-miR-101-3p	ZBTB10	ENSG00000205189	0.995
hsa-miR-101-3p	YES1	ENSG00000176105	0.808
hsa-miR-101-3p	SCN3B	ENSG00000166257	0.831
hsa-miR-101-3p	FA2H	ENSG00000103089	0.717
hsa-miR-101-3p	SKOR1	ENSG00000188779	0.689
hsa-miR-101-3p	ZNF804A	ENSG00000170396	0.593
hsa-miR-101-3p	FEM1C	ENSG00000145780	0.964
hsa-miR-101-3p	FOS	ENSG00000170345	0.312
hsa-miR-101-3p	PDE4D	ENSG00000113448	0.792
hsa-miR-101-3p	ADRB1	ENSG00000043591	0.443
hsa-miR-101-3p	NAT8L	ENSG00000185818	0.834
hsa-miR-101-3p	NEXMIF	ENSG00000050030	0.891
hsa-miR-101-3p	DUSP1	ENSG00000120129	0.319
hsa-miR-101-3p	IL13	ENSG00000169194	0.661
hsa-miR-101-3p	ZBTB21	ENSG00000173276	0.715
hsa-miR-101-3p	KLF2	ENSG00000127528	0.607
hsa-miR-101-3p	ATP1B2	ENSG00000129244	0.962
hsa-miR-101-3p	RASGRF1	ENSG00000058335	0.916
hsa-miR-101-3p	CHD1	ENSG00000153922	0.976
hsa-miR-101-3p	SORCS3	ENSG00000156395	0.815
hsa-miR-101-3p	TMEM170B	ENSG00000205269	0.835
hsa-miR-101-3p	SRGAP1	ENSG00000196935	0.802
hsa-miR-101-3p	E2F8	ENSG00000129173	0.776
hsa-miR-101-3p	MET	ENSG00000105976	0.97
hsa-miR-101-3p	HEY2	ENSG00000135547	0.955
hsa-miR-101-3p	TNFSF11	ENSG00000120659	0.747
hsa-miR-101-3p	PPP1R9A	ENSG00000158528	0.995

hsa-miR-103a-3p	ISL2	ENSG00000159556	0.987
hsa-miR-103a-3p	NR1D2	ENSG00000174738	0.922
hsa-miR-103a-3p	SKOR1	ENSG00000188779	0.763
hsa-miR-103a-3p	RNF152	ENSG00000176641	0.874
hsa-miR-103a-3p	RORB	ENSG00000198963	0.787
hsa-miR-103a-3p	SRGAP1	ENSG00000196935	0.823
hsa-miR-103a-3p	NR4A3	ENSG00000119508	0.892
hsa-miR-103a-3p	EML5	ENSG00000165521	0.814
hsa-miR-103a-3p	PIK3R1	ENSG00000145675	0.833
hsa-miR-103a-3p	PDE4D	ENSG00000113448	0.82
hsa-miR-103a-3p	NAT8L	ENSG00000185818	0.827
hsa-miR-103a-3p	CHD1	ENSG00000153922	0.532
hsa-miR-103a-3p	TNFAIP3	ENSG00000118503	0.855
hsa-miR-103a-3p	PDK4	ENSG00000004799	0.652
hsa-miR-103a-3p	ZBTB10	ENSG00000205189	0.628
hsa-miR-103a-3p	ATP1B2	ENSG00000129244	0.925
hsa-miR-103a-3p	TMEM250	ENSG00000238227	0.893
hsa-miR-103a-3p	CNDP1	ENSG00000150656	0.759
hsa-miR-10a-5p	NR4A3	ENSG00000119508	0.767
hsa-miR-10a-5p	SH3D19	ENSG00000109686	0.765
hsa-miR-10a-5p	ZBTB43	ENSG00000169155	0.956
hsa-miR-10a-5p	RORB	ENSG00000198963	0.672
hsa-miR-10a-5p	SDC1	ENSG00000115884	0.632
hsa-miR-10a-5p	TMEM170B	ENSG00000205269	0.863
hsa-miR-122-5p	ZNF703	ENSG00000183779	1
hsa-miR-122-5p	PDK4	ENSG00000004799	0.592
hsa-miR-124-3p	GOLGA8M	ENSG00000188626	0.645
hsa-miR-124-3p	JAM2	ENSG00000154721	0.568
hsa-miR-124-3p	PIM3	ENSG00000198355	0.598
hsa-miR-124-3p	GFPT2	ENSG00000131459	0.693
hsa-miR-124-3p	TENM1	ENSG00000009694	0.853
hsa-miR-124-3p	PAQR8	ENSG00000170915	0.347
hsa-miR-124-3p	TXNDC5	ENSG00000239264	0.749
hsa-miR-124-3p	EML5	ENSG00000165521	0.687
hsa-miR-124-3p	NR1D2	ENSG00000174738	0.929
hsa-miR-124-3p	SIK1	ENSG00000142178	0.838
hsa-miR-124-3p	CFL2	ENSG00000165410	0.628
hsa-miR-124-3p	P4HA2	ENSG00000072682	0.571
hsa-miR-124-3p	YOD1	ENSG00000180667	0.445
hsa-miR-124-3p	KCNJ2	ENSG00000123700	0.65
hsa-miR-124-3p	SERTAD2	ENSG00000179833	0.657
hsa-miR-124-3p	TMEM170B	ENSG00000205269	0.873
hsa-miR-124-3p	DEPDC1	ENSG00000024526	0.683
hsa-miR-124-3p	NR4A3	ENSG00000119508	0.896
hsa-miR-124-3p	PRLR	ENSG00000113494	0.873
hsa-miR-124-3p	PPP1R9A	ENSG00000158528	0.845
hsa-miR-124-3p	PRICKLE2	ENSG00000163637	0.98

hsa-miR-124-3p	NAT8L	ENSG00000185818	0.449
hsa-miR-124-3p	CHD1	ENSG00000153922	0.472
hsa-miR-124-3p	CAV1	ENSG00000105974	0.767
hsa-miR-124-3p	CACNA1E	ENSG00000198216	0.852
hsa-miR-124-3p	AHRR	ENSG00000063438	0.766
hsa-miR-124-3p	RLIM	ENSG00000131263	0.545
hsa-miR-124-3p	GRASP	ENSG00000161835	0.721
hsa-miR-124-3p	HIC1	ENSG00000177374	0.643
hsa-miR-124-3p	DGAT2	ENSG00000062282	0.938
hsa-miR-124-3p	LRFN1	ENSG00000128011	0.583
hsa-miR-124-3p	ARHGAP39	ENSG00000147799	0.771
hsa-miR-124-3p	RHBDF1	ENSG00000007384	0.622
hsa-miR-124-3p	SSPN	ENSG00000123096	0.994
hsa-miR-124-3p	SRGAP1	ENSG00000196935	0.717
hsa-miR-124-3p	MOCS1	ENSG00000124615	0.698
hsa-miR-124-3p	PDE4D	ENSG00000113448	0.905
hsa-miR-124-3p	MCM10	ENSG00000065328	0.841
hsa-miR-124-3p	TNFSF11	ENSG00000120659	0.68
hsa-miR-124-3p	ARL5B	ENSG00000165997	0.818
hsa-miR-124-3p	PGAP1	ENSG00000197121	0.907
hsa-miR-124-3p	ROR2	ENSG00000169071	0.684
hsa-miR-1249-3p	SKOR1	ENSG00000188779	0.457
hsa-miR-125b-5p	YES1	ENSG00000176105	0.85
hsa-miR-125b-5p	TNFAIP3	ENSG00000118503	0.515
hsa-miR-125b-5p	KIF18B	ENSG00000186185	0.793
hsa-miR-125b-5p	RORB	ENSG00000198963	0.772
hsa-miR-125b-5p	YOD1	ENSG00000180667	0.833
hsa-miR-125b-5p	RASGRF1	ENSG00000058335	0.716
hsa-miR-125b-5p	TMEM170B	ENSG00000205269	0.874
hsa-miR-125b-5p	SCARF2	ENSG00000244486	0.98
hsa-miR-125b-5p	MOCS1	ENSG00000124615	0.864
hsa-miR-125b-5p	NIPAL4	ENSG00000172548	0.609
hsa-miR-125b-5p	C4orf19	ENSG00000154274	0.765
hsa-miR-125b-5p	BORCS6	ENSG00000196544	0.702
hsa-miR-125b-5p	CCDC85C	ENSG00000205476	0.869
hsa-miR-129-5p	COL1A1	ENSG00000108821	0.98
hsa-miR-129-5p	CDKN1C	ENSG00000129757	0.724
hsa-miR-129-5p	TMEM250	ENSG00000238227	0.882
hsa-miR-129-5p	NR4A2	ENSG00000153234	0.747
hsa-miR-129-5p	RBFOX3	ENSG00000167281	0.829
hsa-miR-129-5p	KLHL15	ENSG00000174010	0.953
hsa-miR-129-5p	TXNDC5	ENSG00000239264	1
hsa-miR-129-5p	ZMAT4	ENSG00000165061	0.98
hsa-miR-129-5p	TIPARP	ENSG00000163659	0.821
hsa-miR-129-5p	ZBTB10	ENSG00000205189	0.96
hsa-miR-129-5p	ZNF703	ENSG00000183779	0.968
hsa-miR-129-5p	OTX1	ENSG00000115507	0.82

hsa-miR-129-5p	SIAH1	ENSG00000196470	0.969
hsa-miR-129-5p	P4HA2	ENSG00000072682	0.99
hsa-miR-144-3p	MET	ENSG00000105976	0.99
hsa-miR-144-3p	E2F8	ENSG00000129173	0.698
hsa-miR-144-3p	RORB	ENSG00000198963	0.812
hsa-miR-144-3p	RASGRF1	ENSG00000058335	0.958
hsa-miR-144-3p	FOS	ENSG00000170345	0.855
hsa-miR-144-3p	SORCS3	ENSG00000156395	0.716
hsa-miR-144-3p	ZBTB21	ENSG00000173276	0.873
hsa-miR-144-3p	ADRB1	ENSG00000043591	0.884
hsa-miR-144-3p	DUSP1	ENSG00000120129	0.89
hsa-miR-144-3p	NR1D2	ENSG00000174738	0.987
hsa-miR-144-3p	TNFSF11	ENSG00000120659	0.552
hsa-miR-144-3p	PDE4D	ENSG00000113448	0.937
hsa-miR-144-3p	HEY2	ENSG00000135547	0.982
hsa-miR-144-3p	TENM1	ENSG00000009694	0.97
hsa-miR-144-3p	SRGAP1	ENSG00000196935	0.992
hsa-miR-144-3p	ATP1B2	ENSG00000129244	0.986
hsa-miR-144-3p	CFL2	ENSG00000165410	0.71
hsa-miR-153-3p	EFNA3	ENSG00000143590	0.631
hsa-miR-153-3p	PDE4D	ENSG00000113448	0.882
hsa-miR-153-3p	ZNF703	ENSG00000183779	0.892
hsa-miR-153-3p	CFL2	ENSG00000165410	0.562
hsa-miR-153-3p	NRARP	ENSG00000198435	0.925
hsa-miR-153-3p	PIGA	ENSG00000165195	0.811
hsa-miR-153-3p	FEM1C	ENSG00000145780	0.499
hsa-miR-153-3p	PIK3R1	ENSG00000145675	0.791
hsa-miR-153-3p	ZBTB10	ENSG00000205189	0.868
hsa-miR-153-3p	SYN1	ENSG00000008056	0.786
hsa-miR-153-3p	CHD1	ENSG00000153922	0.976
hsa-miR-153-3p	SERTAD2	ENSG00000179833	0.497
hsa-miR-153-3p	GFPT2	ENSG00000131459	0.462
hsa-miR-153-3p	ZBTB43	ENSG00000169155	0.802
hsa-miR-153-3p	RNF152	ENSG00000176641	0.956
hsa-miR-153-3p	CCNT1	ENSG00000129315	0.875
hsa-miR-153-3p	HEY2	ENSG00000135547	0.865
hsa-miR-153-3p	TMEM170B	ENSG00000205269	0.936
hsa-miR-153-3p	SCML1	ENSG00000047634	0.7
hsa-miR-153-3p	GRIK4	ENSG00000149403	0.678
hsa-miR-153-3p	ZBTB21	ENSG00000173276	0.742
hsa-miR-15a-5p	CCDC85C	ENSG00000205476	0.773
hsa-miR-15a-5p	CDC42EP2	ENSG00000149798	0.371
hsa-miR-15a-5p	RNF152	ENSG00000176641	0.876
hsa-miR-15a-5p	BORCS6	ENSG00000196544	0.357
hsa-miR-15a-5p	NAT8L	ENSG00000185818	0.819
hsa-miR-15a-5p	OTX1	ENSG00000115507	0.667
hsa-miR-15a-5p	PIM3	ENSG00000198355	0.868

hsa-miR-15a-5p	SLC23A3	ENSG00000213901	0.824
hsa-miR-15a-5p	CNIH2	ENSG00000174871	0.719
hsa-miR-15a-5p	NRARP	ENSG00000198435	0.973
hsa-miR-15a-5p	ARL5B	ENSG00000165997	0.816
hsa-miR-15a-5p	BTN1A1	ENSG00000124557	0.447
hsa-miR-15a-5p	GRIN1	ENSG00000176884	0.98
hsa-miR-15a-5p	AOC1	ENSG00000002726	0.805
hsa-miR-15a-5p	ZBTB10	ENSG00000205189	0.786
hsa-miR-15a-5p	TMEM255A	ENSG00000125355	0.648
hsa-miR-15a-5p	SOX5	ENSG00000134532	0.739
hsa-miR-15a-5p	ZBTB43	ENSG00000169155	0.792
hsa-miR-15a-5p	TMEM170B	ENSG00000205269	0.834
hsa-miR-15a-5p	LRRN3	ENSG00000173114	0.764
hsa-miR-15a-5p	YOD1	ENSG00000180667	0.957
hsa-miR-15a-5p	RLIM	ENSG00000131263	0.945
hsa-miR-15a-5p	RASGEF1B	ENSG00000138670	0.14
hsa-miR-15a-5p	SIAH1	ENSG00000196470	0.709
hsa-miR-15a-5p	MOCS1	ENSG00000124615	0.952
hsa-miR-15a-5p	TMEM250	ENSG00000238227	0.878
hsa-miR-15a-5p	RGPD6	ENSG00000183054	0.755
hsa-miR-15a-5p	SKOR1	ENSG00000188779	0.82
hsa-miR-15a-5p	RORB	ENSG00000198963	0.819
hsa-miR-15a-5p	CACNA1E	ENSG00000198216	0.97
hsa-miR-15a-5p	KLHL15	ENSG00000174010	0.811
hsa-miR-15a-5p	CD180	ENSG00000134061	1
hsa-miR-15a-5p	KCNJ2	ENSG00000123700	0.501
hsa-miR-15a-5p	SIK1	ENSG00000142178	0.602
hsa-miR-15a-5p	SRGAP1	ENSG00000196935	0.987
hsa-miR-15a-5p	TCTE1	ENSG00000146221	0.765
hsa-miR-15a-5p	PIK3R1	ENSG00000145675	0.775
hsa-miR-15a-5p	PDK4	ENSG00000004799	0.434
hsa-miR-15b-5p	SIK1	ENSG00000142178	0.613
hsa-miR-15b-5p	SLC23A3	ENSG00000213901	0.813
hsa-miR-15b-5p	PIM3	ENSG00000198355	0.868
hsa-miR-15b-5p	CDC42EP2	ENSG00000149798	0.36
hsa-miR-15b-5p	OTX1	ENSG00000115507	0.667
hsa-miR-15b-5p	CACNA1E	ENSG00000198216	0.97
hsa-miR-15b-5p	MOCS1	ENSG00000124615	0.958
hsa-miR-15b-5p	NRARP	ENSG00000198435	0.972
hsa-miR-15b-5p	RGPD6	ENSG00000183054	0.755
hsa-miR-15b-5p	PIK3R1	ENSG00000145675	0.775
hsa-miR-15b-5p	SOX5	ENSG00000134532	0.739
hsa-miR-15b-5p	SRGAP1	ENSG00000196935	0.987
hsa-miR-15b-5p	KLHL15	ENSG00000174010	0.811
hsa-miR-15b-5p	CCDC85C	ENSG00000205476	0.782
hsa-miR-15b-5p	SIAH1	ENSG00000196470	0.709
hsa-miR-15b-5p	CD180	ENSG00000134061	1

hsa-miR-15b-5p	TMEM250	ENSG00000238227	0.878
hsa-miR-15b-5p	RORB	ENSG00000198963	0.835
hsa-miR-15b-5p	TCTE1	ENSG00000146221	0.765
hsa-miR-15b-5p	LRRN3	ENSG00000173114	0.764
hsa-miR-15b-5p	TMEM170B	ENSG00000205269	0.834
hsa-miR-15b-5p	SKOR1	ENSG00000188779	0.82
hsa-miR-15b-5p	RNF152	ENSG00000176641	0.885
hsa-miR-15b-5p	GRIN1	ENSG00000176884	0.98
hsa-miR-15b-5p	KCNJ2	ENSG00000123700	0.489
hsa-miR-15b-5p	RLIM	ENSG00000131263	0.956
hsa-miR-15b-5p	YOD1	ENSG00000180667	0.957
hsa-miR-15b-5p	ARL5B	ENSG00000165997	0.816
hsa-miR-15b-5p	ZBTB43	ENSG00000169155	0.781
hsa-miR-15b-5p	CNIH2	ENSG00000174871	0.719
hsa-miR-15b-5p	RASGEF1B	ENSG00000138670	0.148
hsa-miR-15b-5p	BTN1A1	ENSG00000124557	0.455
hsa-miR-15b-5p	PDK4	ENSG00000004799	0.456
hsa-miR-15b-5p	BORCS6	ENSG00000196544	0.39
hsa-miR-15b-5p	ZBTB10	ENSG00000205189	0.786
hsa-miR-15b-5p	TMEM255A	ENSG00000125355	0.659
hsa-miR-15b-5p	AOC1	ENSG00000002726	0.794
hsa-miR-15b-5p	NAT8L	ENSG00000185818	0.819
hsa-miR-17-5p	SERTAD2	ENSG00000179833	0.825
hsa-miR-17-5p	OSM	ENSG00000099985	0.604
hsa-miR-17-5p	BTG3	ENSG00000154640	0.332
hsa-miR-17-5p	ZBTB43	ENSG00000169155	0.799
hsa-miR-17-5p	TIPARP	ENSG00000163659	0.863
hsa-miR-17-5p	PER1	ENSG00000179094	0.964
hsa-miR-17-5p	SMAD6	ENSG00000137834	0.969
hsa-miR-17-5p	TNFSF11	ENSG00000120659	0.724
hsa-miR-17-5p	SRGAP1	ENSG00000196935	0.894
hsa-miR-17-5p	MASTL	ENSG00000120539	0.468
hsa-miR-17-5p	SLC1A2	ENSG00000110436	0.98
hsa-miR-17-5p	SIK1	ENSG00000142178	0.72
hsa-miR-17-5p	FEM1C	ENSG00000145780	0.647
hsa-miR-17-5p	DUSP8	ENSG00000184545	0.895
hsa-miR-17-5p	ARHGEF10	ENSG00000104728	0.759
hsa-miR-17-5p	YOD1	ENSG00000180667	0.85
hsa-miR-17-5p	KLHL15	ENSG00000174010	0.767
hsa-miR-17-5p	YES1	ENSG00000176105	0.827
hsa-miR-17-5p	HIF1A	ENSG00000100644	0.796
hsa-miR-17-5p	PIK3R1	ENSG00000145675	0.981
hsa-miR-17-5p	PANX2	ENSG00000073150	0.816
hsa-miR-17-5p	TENM1	ENSG00000009694	0.874
hsa-miR-17-5p	SYNM	ENSG00000182253	0.924
hsa-miR-17-5p	RLIM	ENSG00000131263	0.668
hsa-miR-17-5p	AHRR	ENSG00000063438	0.983

hsa-miR-17-5p	RORB	ENSG00000198963	0.917
hsa-miR-17-5p	CHST7	ENSG00000147119	0.999
hsa-miR-17-5p	ZNF703	ENSG00000183779	1
hsa-miR-17-5p	PFKFB3	ENSG00000170525	0.822
hsa-miR-17-5p	ZBTB21	ENSG00000173276	0.91
hsa-miR-17-5p	SKOR1	ENSG00000188779	0.695
hsa-miR-17-5p	NR4A2	ENSG00000153234	0.692
hsa-miR-17-5p	CXCL8	ENSG00000169429	0.572
hsa-miR-17-5p	CFL2	ENSG00000165410	0.559
hsa-miR-17-5p	NR4A3	ENSG00000119508	0.58
hsa-miR-17-5p	EREG	ENSG00000124882	0.671
hsa-miR-17-5p	ZNF805	ENSG00000204524	0.935
hsa-miR-187-3p	LRFN1	ENSG00000128011	0.556
hsa-miR-188-5p	ISL2	ENSG00000159556	1
hsa-miR-188-5p	SPRED3	ENSG00000188766	0.974
hsa-miR-188-5p	ARL5B	ENSG00000165997	0.827
hsa-miR-190a-5p	CCSER1	ENSG00000184305	0.98
hsa-miR-190a-5p	RLIM	ENSG00000131263	0.925
hsa-miR-190a-5p	TENM1	ENSG00000009694	0.958
hsa-miR-190b	TENM1	ENSG00000009694	0.969
hsa-miR-190b	RLIM	ENSG00000131263	0.892
hsa-miR-190b	CCSER1	ENSG00000184305	0.939
hsa-miR-193a-3p	SOX5	ENSG00000134532	0.777
hsa-miR-193a-3p	KLHL15	ENSG00000174010	0.926
hsa-miR-193a-3p	SIAH1	ENSG00000196470	0.601
hsa-miR-193a-3p	SYN1	ENSG00000008056	0.852
hsa-miR-193a-3p	KCNJ2	ENSG00000123700	0.529
hsa-miR-193a-3p	ARHGAP39	ENSG00000147799	0.812
hsa-miR-193a-5p	COL1A1	ENSG00000108821	0.824
hsa-miR-193a-5p	SMAD9	ENSG00000120693	0.928
hsa-miR-193a-5p	SPRED3	ENSG00000188766	0.776
hsa-miR-193a-5p	GOLGA8M	ENSG00000188626	0.82
hsa-miR-193b-3p	KCNJ2	ENSG00000123700	0.521
hsa-miR-193b-3p	ARHGAP39	ENSG00000147799	0.812
hsa-miR-193b-3p	SYN1	ENSG00000008056	0.852
hsa-miR-193b-3p	SIAH1	ENSG00000196470	0.601
hsa-miR-193b-3p	KLHL15	ENSG00000174010	0.926
hsa-miR-193b-3p	SOX5	ENSG00000134532	0.789
hsa-miR-196a-5p	PRLR	ENSG00000113494	0.925
hsa-miR-196a-5p	SMAD6	ENSG00000137834	0.668
hsa-miR-196a-5p	FLRT1	ENSG00000126500	0.712
hsa-miR-196a-5p	KLHL15	ENSG00000174010	0.979
hsa-miR-196a-5p	OTX1	ENSG00000115507	0.776
hsa-miR-196a-5p	KCNJ2	ENSG00000123700	0.529
hsa-miR-196a-5p	B3GNT7	ENSG00000156966	0.831
hsa-miR-196a-5p	YOD1	ENSG00000180667	0.718
hsa-miR-196a-5p	ZBTB10	ENSG00000205189	0.922

hsa-miR-196a-5p	ABCB9	ENSG00000150967	0.639
hsa-miR-196a-5p	COL1A1	ENSG00000108821	0.777
hsa-miR-199a-5p	PPP1R9A	ENSG00000158528	0.757
hsa-miR-199a-5p	RLIM	ENSG00000131263	0.648
hsa-miR-199a-5p	ZNF579	ENSG00000218891	0.514
hsa-miR-199a-5p	RORB	ENSG00000198963	0.849
hsa-miR-199a-5p	HIF1A	ENSG00000100644	0.758
hsa-miR-199a-5p	ARHGAP29	ENSG00000137962	0.813
hsa-miR-199a-5p	CDKN1C	ENSG00000129757	0.73
hsa-miR-199a-5p	PDE4D	ENSG00000113448	0.708
hsa-miR-199a-5p	SPRED3	ENSG00000188766	0.918
hsa-miR-199a-5p	ZNF547	ENSG00000152433	-0.413
hsa-miR-199a-5p	SORCS3	ENSG00000156395	0.615
hsa-miR-199a-5p	CAV1	ENSG00000105974	0.819
hsa-miR-199a-5p	ZNF703	ENSG00000183779	1
hsa-miR-199a-5p	OTX1	ENSG00000115507	0.698
hsa-miR-199b-5p	ZNF703	ENSG00000183779	1
hsa-miR-199b-5p	ZNF547	ENSG00000152433	-0.412
hsa-miR-199b-5p	RORB	ENSG00000198963	0.838
hsa-miR-199b-5p	HIF1A	ENSG00000100644	0.769
hsa-miR-199b-5p	PDE4D	ENSG00000113448	0.708
hsa-miR-199b-5p	RLIM	ENSG00000131263	0.648
hsa-miR-199b-5p	ARHGAP29	ENSG00000137962	0.813
hsa-miR-199b-5p	CDKN1C	ENSG00000129757	0.73
hsa-miR-199b-5p	PPP1R9A	ENSG00000158528	0.757
hsa-miR-199b-5p	OTX1	ENSG00000115507	0.706
hsa-miR-199b-5p	SPRED3	ENSG00000188766	0.939
hsa-miR-199b-5p	SORCS3	ENSG00000156395	0.623
hsa-miR-199b-5p	ZNF579	ENSG00000218891	0.514
hsa-miR-199b-5p	CAV1	ENSG00000105974	0.819
hsa-miR-204-5p	CCSER1	ENSG00000184305	0.828
hsa-miR-204-5p	ARHGAP29	ENSG00000137962	0.759
hsa-miR-204-5p	PPP1R9A	ENSG00000158528	0.977
hsa-miR-204-5p	NEXMIF	ENSG00000050030	0.888
hsa-miR-204-5p	NR4A2	ENSG00000153234	0.689
hsa-miR-204-5p	ISL2	ENSG00000159556	0.978
hsa-miR-204-5p	TMEM255A	ENSG00000125355	0.86
hsa-miR-204-5p	SMAD6	ENSG00000137834	1
hsa-miR-204-5p	RORB	ENSG00000198963	0.98
hsa-miR-204-5p	PDF	ENSG00000258429	1
hsa-miR-204-5p	TENM1	ENSG00000009694	0.92
hsa-miR-204-5p	ARL5B	ENSG00000165997	0.926
hsa-miR-204-5p	NRARP	ENSG00000198435	0.97
hsa-miR-204-5p	ZBTB21	ENSG00000173276	0.898
hsa-miR-204-5p	CD180	ENSG00000134061	1
hsa-miR-206	FZD7	ENSG00000155760	0.775
hsa-miR-206	KLHL15	ENSG00000174010	0.849

hsa-miR-206	ZBTB21	ENSG00000173276	0.846
hsa-miR-206	KCNJ2	ENSG00000123700	0.767
hsa-miR-206	ISL2	ENSG00000159556	0.893
hsa-miR-206	YES1	ENSG00000176105	0.864
hsa-miR-206	NETO1	ENSG00000166342	0.779
hsa-miR-206	SRXN1	ENSG00000271303	0.914
hsa-miR-206	NEXMIF	ENSG00000050030	0.897
hsa-miR-206	SOX5	ENSG00000134532	0.963
hsa-miR-206	PPP1R9A	ENSG00000158528	0.926
hsa-miR-206	MET	ENSG00000105976	0.73
hsa-miR-206	JUND	ENSG00000130522	0.722
hsa-miR-206	NR4A2	ENSG00000153234	0.76
hsa-miR-206	NR4A3	ENSG00000119508	0.834
hsa-miR-20a-5p	EREG	ENSG00000124882	0.657
hsa-miR-20a-5p	CHST7	ENSG00000147119	0.999
hsa-miR-20a-5p	TENM1	ENSG00000009694	0.86
hsa-miR-20a-5p	AHRR	ENSG00000063438	0.983
hsa-miR-20a-5p	PFKFB3	ENSG00000170525	0.808
hsa-miR-20a-5p	CXCL8	ENSG00000169429	0.593
hsa-miR-20a-5p	NR4A3	ENSG00000119508	0.563
hsa-miR-20a-5p	SYNM	ENSG00000182253	0.915
hsa-miR-20a-5p	PANX2	ENSG00000073150	0.802
hsa-miR-20a-5p	YES1	ENSG00000176105	0.814
hsa-miR-20a-5p	BTG3	ENSG00000154640	0.329
hsa-miR-20a-5p	ZNF805	ENSG00000204524	0.949
hsa-miR-20a-5p	PIK3R1	ENSG00000145675	0.976
hsa-miR-20a-5p	YOD1	ENSG00000180667	0.844
hsa-miR-20a-5p	ZNF703	ENSG00000183779	1
hsa-miR-20a-5p	SERTAD2	ENSG00000179833	0.828
hsa-miR-20a-5p	ARHGEF10	ENSG00000104728	0.778
hsa-miR-20a-5p	SKOR1	ENSG00000188779	0.73
hsa-miR-20a-5p	PER1	ENSG00000179094	0.96
hsa-miR-20a-5p	SLC1A2	ENSG00000110436	0.98
hsa-miR-20a-5p	ZBTB21	ENSG00000173276	0.923
hsa-miR-20a-5p	SMAD6	ENSG00000137834	0.967
hsa-miR-20a-5p	KLHL15	ENSG00000174010	0.753
hsa-miR-20a-5p	NR4A2	ENSG00000153234	0.69
hsa-miR-20a-5p	ZBTB43	ENSG00000169155	0.752
hsa-miR-20a-5p	HIF1A	ENSG00000100644	0.761
hsa-miR-20a-5p	SIK1	ENSG00000142178	0.741
hsa-miR-20a-5p	MASTL	ENSG00000120539	0.481
hsa-miR-20a-5p	RLIM	ENSG00000131263	0.632
hsa-miR-20a-5p	FEM1C	ENSG00000145780	0.667
hsa-miR-20a-5p	CFL2	ENSG00000165410	0.536
hsa-miR-20a-5p	DUSP8	ENSG00000184545	0.883
hsa-miR-20a-5p	SRGAP1	ENSG00000196935	0.885
hsa-miR-20a-5p	TNFSF11	ENSG00000120659	0.721

hsa-miR-20a-5p	RORB	ENSG00000198963	0.925
hsa-miR-20a-5p	TIPARP	ENSG00000163659	0.849
hsa-miR-20a-5p	OSM	ENSG00000099985	0.633
hsa-miR-21-5p	PIK3R1	ENSG00000145675	0.778
hsa-miR-21-5p	SOX5	ENSG00000134532	0.591
hsa-miR-21-5p	KLHL15	ENSG00000174010	0.947
hsa-miR-21-5p	ESM1	ENSG00000164283	0.826
hsa-miR-21-5p	RHOB	ENSG00000143878	0.877
hsa-miR-21-5p	YOD1	ENSG00000180667	0.583
hsa-miR-21-5p	FASLG	ENSG00000117560	0.36
hsa-miR-21-5p	DUSP8	ENSG00000184545	0.656
hsa-miR-218-5p	GRM3	ENSG00000198822	0.729
hsa-miR-218-5p	NEXMIF	ENSG00000050030	0.856
hsa-miR-218-5p	ARL5B	ENSG00000165997	0.917
hsa-miR-218-5p	RORB	ENSG00000198963	0.773
hsa-miR-218-5p	NR1D2	ENSG00000174738	0.929
hsa-miR-218-5p	ADRB1	ENSG00000043591	0.859
hsa-miR-218-5p	NAT8L	ENSG00000185818	0.863
hsa-miR-218-5p	ZBTB10	ENSG00000205189	0.939
hsa-miR-218-5p	TXNDC5	ENSG00000239264	0.784
hsa-miR-218-5p	COL1A1	ENSG00000108821	0.742
hsa-miR-218-5p	RNF152	ENSG00000176641	0.885
hsa-miR-218-5p	RLIM	ENSG00000131263	0.782
hsa-miR-218-5p	PIK3R1	ENSG00000145675	0.779
hsa-miR-218-5p	SERTAD2	ENSG00000179833	0.625
hsa-miR-218-5p	SH3D19	ENSG00000109686	0.761
hsa-miR-218-5p	SOX5	ENSG00000134532	0.793
hsa-miR-218-5p	RNF103	ENSG00000239305	0.461
hsa-miR-218-5p	PRLR	ENSG00000113494	0.906
hsa-miR-218-5p	ZMAT4	ENSG00000165061	0.727
hsa-miR-218-5p	RGS20	ENSG00000147509	0.886
hsa-miR-219a-5p	DIAPH3	ENSG00000139734	0.874
hsa-miR-219a-5p	KCNJ2	ENSG00000123700	0.713
hsa-miR-219a-5p	CFL2	ENSG00000165410	0.991
hsa-miR-219a-5p	C4orf19	ENSG00000154274	0.804
hsa-miR-219a-5p	CACNA1E	ENSG00000198216	0.872
hsa-miR-219a-5p	SOX5	ENSG00000134532	0.822
hsa-miR-219a-5p	RNF152	ENSG00000176641	0.887
hsa-miR-219a-5p	RORB	ENSG00000198963	0.115
hsa-miR-219a-5p	PDE4D	ENSG00000113448	0.807
hsa-miR-24-3p	KCNJ2	ENSG00000123700	0.771
hsa-miR-24-3p	CACNA1E	ENSG00000198216	0.97
hsa-miR-24-3p	YOD1	ENSG00000180667	0.73
hsa-miR-24-3p	COMTD1	ENSG00000165644	0.994
hsa-miR-24-3p	SCML1	ENSG00000047634	0.439
hsa-miR-24-3p	PER1	ENSG00000179094	0.781
hsa-miR-24-3p	CHI3L1	ENSG00000133048	0.786

hsa-miR-24-3p	KLHL15	ENSG00000174010	0.96
hsa-miR-24-3p	MOCS1	ENSG00000124615	0.97
hsa-miR-24-3p	DUSP8	ENSG00000184545	0.791
hsa-miR-24-3p	ATP1B2	ENSG00000129244	0.601
hsa-miR-24-3p	HIC1	ENSG00000177374	0.889
hsa-miR-24-3p	FASLG	ENSG00000117560	0.673
hsa-miR-24-3p	CCDC85C	ENSG00000205476	0.893
hsa-miR-24-3p	SLC23A3	ENSG00000213901	0.777
hsa-miR-24-3p	ABCB9	ENSG00000150967	0.718
hsa-miR-24-3p	RHBDL1	ENSG00000103269	0.589
hsa-miR-24-3p	CDKN1C	ENSG00000129757	0.762
hsa-miR-24-3p	TNFSF9	ENSG00000125657	0.587
hsa-miR-24-3p	CITED4	ENSG00000179862	0.418
hsa-miR-24-3p	FCRLB	ENSG00000162746	0.865
hsa-miR-24-3p	TENM1	ENSG00000009694	0.901
hsa-miR-301a-3p	NEXMIF	ENSG00000050030	0.743
hsa-miR-301a-3p	TMEM170B	ENSG00000205269	0.651
hsa-miR-301a-3p	PPP1R9A	ENSG00000158528	0.936
hsa-miR-301a-3p	DEPDC1	ENSG00000024526	0.786
hsa-miR-301a-3p	PDE4D	ENSG00000113448	0.824
hsa-miR-301a-3p	CFL2	ENSG00000165410	0.905
hsa-miR-301a-3p	DIAPH3	ENSG00000139734	0.845
hsa-miR-301a-3p	COL9A3	ENSG00000092758	0.986
hsa-miR-301a-3p	KLHL15	ENSG00000174010	0.884
hsa-miR-301a-3p	TENM1	ENSG00000009694	0.97
hsa-miR-301a-3p	RLIM	ENSG00000131263	0.807
hsa-miR-301a-3p	RORB	ENSG00000198963	0.94
hsa-miR-301a-3p	PFKFB3	ENSG00000170525	0.904
hsa-miR-301a-3p	TMEM250	ENSG00000238227	0.502
hsa-miR-301a-3p	PRICKLE2	ENSG00000163637	0.85
hsa-miR-301a-3p	EREG	ENSG00000124882	0.406
hsa-miR-301a-3p	SIK1	ENSG00000142178	0.817
hsa-miR-301a-3p	NRARP	ENSG00000198435	0.866
hsa-miR-301a-3p	FAM43A	ENSG00000185112	0.694
hsa-miR-301a-3p	PIGA	ENSG00000165195	0.657
hsa-miR-301a-3p	MET	ENSG00000105976	0.596
hsa-miR-301a-3p	SYNM	ENSG00000182253	0.878
hsa-miR-301a-3p	SH3D19	ENSG00000109686	0.394
hsa-miR-301a-3p	SOX5	ENSG00000134532	0.937
hsa-miR-3064-5p	CCNT1	ENSG00000129315	0.957
hsa-miR-3064-5p	RORB	ENSG00000198963	0.905
hsa-miR-3064-5p	CHST7	ENSG00000147119	0.993
hsa-miR-3064-5p	CHD1	ENSG00000153922	0.985
hsa-miR-3064-5p	TCTE1	ENSG00000146221	0.934
hsa-miR-3064-5p	MOCS1	ENSG00000124615	0.917
hsa-miR-30c-5p	GLDC	ENSG00000178445	0.757
hsa-miR-30c-5p	RLIM	ENSG00000131263	0.776

hsa-miR-30c-5p	SEC14L2	ENSG00000100003	0.947
hsa-miR-30c-5p	SORCS3	ENSG00000156395	0.905
hsa-miR-30c-5p	PDE4D	ENSG00000113448	0.858
hsa-miR-30c-5p	ADRB1	ENSG00000043591	0.861
hsa-miR-30c-5p	GFPT2	ENSG00000131459	0.713
hsa-miR-30c-5p	RASGEF1B	ENSG00000138670	0.852
hsa-miR-30c-5p	ZBTB10	ENSG00000205189	0.813
hsa-miR-30c-5p	MYBL2	ENSG00000101057	0.653
hsa-miR-30c-5p	PPP1R9A	ENSG00000158528	0.857
hsa-miR-30c-5p	CCSER1	ENSG00000184305	1
hsa-miR-30c-5p	TENM1	ENSG00000009694	0.97
hsa-miR-30c-5p	NR4A2	ENSG00000153234	0.585
hsa-miR-30c-5p	SOCS1	ENSG00000185338	0.48
hsa-miR-30c-5p	ERRFI1	ENSG00000116285	0.855
hsa-miR-30c-5p	RHOB	ENSG00000143878	0.814
hsa-miR-30c-5p	RTN4R	ENSG00000040608	0.724
hsa-miR-30c-5p	EFNA3	ENSG00000143590	0.697
hsa-miR-30c-5p	P4HA2	ENSG00000072682	0.699
hsa-miR-30c-5p	CFL2	ENSG00000165410	0.685
hsa-miR-30c-5p	FAM43A	ENSG00000185112	0.522
hsa-miR-30c-5p	COL9A3	ENSG00000092758	0.618
hsa-miR-30c-5p	PRLR	ENSG00000113494	0.908
hsa-miR-30c-5p	TXNDC5	ENSG00000239264	0.784
hsa-miR-30c-5p	YES1	ENSG00000176105	0.85
hsa-miR-30c-5p	GRM3	ENSG00000198822	0.86
hsa-miR-30c-5p	JAM2	ENSG00000154721	0.921
hsa-miR-30c-5p	DBF4	ENSG00000006634	0.558
hsa-miR-30c-5p	PIGA	ENSG00000165195	0.772
hsa-miR-30c-5p	YOD1	ENSG00000180667	0.665
hsa-miR-30c-5p	TMEM170B	ENSG00000205269	0.761
hsa-miR-30c-5p	CHD1	ENSG00000153922	0.547
hsa-miR-30c-5p	C4orf19	ENSG00000154274	0.743
hsa-miR-32-5p	CDC42EP2	ENSG00000149798	0.838
hsa-miR-32-5p	CDKN1C	ENSG00000129757	0.597
hsa-miR-32-5p	TENM1	ENSG00000009694	0.97
hsa-miR-32-5p	SH3D19	ENSG00000109686	0.811
hsa-miR-32-5p	SKOR1	ENSG00000188779	0.729
hsa-miR-32-5p	CHCHD10	ENSG00000250479	0.51
hsa-miR-32-5p	ARHGEF10	ENSG00000104728	0.767
hsa-miR-32-5p	SMAD6	ENSG00000137834	0.956
hsa-miR-32-5p	CFL2	ENSG00000165410	0.868
hsa-miR-32-5p	NR4A3	ENSG00000119508	0.672
hsa-miR-32-5p	SIK1	ENSG00000142178	0.77
hsa-miR-32-5p	KLHL15	ENSG00000174010	0.593
hsa-miR-32-5p	TMEM255A	ENSG00000125355	0.731
hsa-miR-32-5p	ARHGAP29	ENSG00000137962	0.724
hsa-miR-32-5p	PPP1R9A	ENSG00000158528	0.968

hsa-miR-32-5p	ZBTB10	ENSG00000205189	0.908
hsa-miR-32-5p	ARL5B	ENSG00000165997	0.957
hsa-miR-32-5p	KLF2	ENSG00000127528	0.487
hsa-miR-32-5p	ZNF804A	ENSG00000170396	0.778
hsa-miR-32-5p	DUSP1	ENSG00000120129	0.831
hsa-miR-32-5p	ADRB1	ENSG00000043591	0.522
hsa-miR-32-5p	NETO1	ENSG00000166342	0.797
hsa-miR-32-5p	GOLGA8M	ENSG00000188626	0.812
hsa-miR-32-5p	AURKA	ENSG00000087586	0.634
hsa-miR-32-5p	GFPT2	ENSG00000131459	0.662
hsa-miR-32-5p	CHST7	ENSG00000147119	0.518
hsa-miR-32-5p	SERTAD2	ENSG00000179833	0.799
hsa-miR-32-5p	SORCS3	ENSG00000156395	0.94
hsa-miR-32-5p	FASLG	ENSG00000117560	0.749
hsa-miR-32-5p	INAFM1	ENSG00000257704	0.817
hsa-miR-340-5p	SRGAP1	ENSG00000196935	0.981
hsa-miR-340-5p	SERTAD2	ENSG00000179833	0.975
hsa-miR-340-5p	RNF103	ENSG00000239305	0.891
hsa-miR-340-5p	RGPD6	ENSG00000183054	0.97
hsa-miR-340-5p	AVPR1A	ENSG00000166148	0.991
hsa-miR-340-5p	RLIM	ENSG00000131263	0.98
hsa-miR-340-5p	PDK4	ENSG00000004799	0.949
hsa-miR-340-5p	DBF4	ENSG00000006634	0.921
hsa-miR-340-5p	GFPT2	ENSG00000131459	0.99
hsa-miR-340-5p	YOD1	ENSG00000180667	0.995
hsa-miR-340-5p	NR1D2	ENSG00000174738	0.988
hsa-miR-340-5p	FA2H	ENSG00000103089	0.99
hsa-miR-340-5p	HIF1A	ENSG00000100644	0.99
hsa-miR-340-5p	ARL5B	ENSG00000165997	0.979
hsa-miR-340-5p	YES1	ENSG00000176105	0.99
hsa-miR-340-5p	OSM	ENSG00000099985	0.944
hsa-miR-340-5p	NRARP	ENSG00000198435	0.995
hsa-miR-340-5p	FEM1C	ENSG00000145780	0.981
hsa-miR-340-5p	MET	ENSG00000105976	0.99
hsa-miR-340-5p	ZBTB10	ENSG00000205189	0.99
hsa-miR-340-5p	ARHGAP29	ENSG00000137962	0.983
hsa-miR-340-5p	TMEM170B	ENSG00000205269	0.955
hsa-miR-340-5p	GPR15	ENSG00000154165	0.97
hsa-miR-340-5p	ZBTB43	ENSG00000169155	0.955
hsa-miR-340-5p	NEXMIF	ENSG00000050030	0.95
hsa-miR-340-5p	RNF152	ENSG00000176641	0.995
hsa-miR-340-5p	KLHL15	ENSG00000174010	0.998
hsa-miR-340-5p	NPTX2	ENSG00000106236	0.993
hsa-miR-340-5p	ADAMTS1	ENSG00000154734	0.978
hsa-miR-340-5p	NR4A3	ENSG00000119508	0.92
hsa-miR-340-5p	RORB	ENSG00000198963	0.99
hsa-miR-340-5p	ZBTB21	ENSG00000173276	0.993

hsa-miR-340-5p	CFL2	ENSG00000165410	0.998
hsa-miR-362-5p	ZBTB10	ENSG00000205189	0.855
hsa-miR-362-5p	CHD1	ENSG00000153922	0.989
hsa-miR-362-5p	PAQR8	ENSG00000170915	0.709
hsa-miR-362-5p	RLIM	ENSG00000131263	0.9
hsa-miR-362-5p	CCSER1	ENSG00000184305	0.801
hsa-miR-365a-3p	SRGAP1	ENSG00000196935	0.745
hsa-miR-365a-3p	REL	ENSG00000162924	0.99
hsa-miR-365a-3p	KCNJ2	ENSG00000123700	0.665
hsa-miR-365a-3p	RGS20	ENSG00000147509	0.926
hsa-miR-365a-3p	NR1D2	ENSG00000174738	0.766
hsa-miR-365a-3p	CCSER1	ENSG00000184305	0.814
hsa-miR-365a-3p	PDE4D	ENSG00000113448	0.882
hsa-miR-365a-3p	ADAMTS1	ENSG00000154734	0.648
hsa-miR-365a-3p	RNF152	ENSG00000176641	0.641
hsa-miR-365a-3p	SERTAD2	ENSG00000179833	0.869
hsa-miR-365a-3p	NR4A2	ENSG00000153234	0.746
hsa-miR-374a-5p	ARL2BP	ENSG00000102931	0.945
hsa-miR-374a-5p	ZBTB10	ENSG00000205189	0.98
hsa-miR-374a-5p	TENM1	ENSG00000009694	0.98
hsa-miR-374a-5p	SMAD6	ENSG00000137834	0.697
hsa-miR-374a-5p	NR4A3	ENSG00000119508	0.981
hsa-miR-374a-5p	JAM2	ENSG00000154721	0.903
hsa-miR-374a-5p	CEBPB	ENSG00000172216	0.782
hsa-miR-374a-5p	ADRB1	ENSG00000043591	0.952
hsa-miR-374a-5p	YOD1	ENSG00000180667	0.95
hsa-miR-374a-5p	PDE4D	ENSG00000113448	0.98
hsa-miR-374a-5p	SRGAP1	ENSG00000196935	0.987
hsa-miR-374a-5p	SERTAD2	ENSG00000179833	0.996
hsa-miR-374a-5p	RORB	ENSG00000198963	0.98
hsa-miR-374a-5p	DGAT2	ENSG00000062282	0.868
hsa-miR-374a-5p	NR4A2	ENSG00000153234	0.98
hsa-miR-374a-5p	ZBTB43	ENSG00000169155	0.97
hsa-miR-374a-5p	NPTX2	ENSG00000106236	0.916
hsa-miR-374a-5p	HES1	ENSG00000114315	0.852
hsa-miR-449a	CHD1	ENSG00000153922	0.693
hsa-miR-449a	ZNF579	ENSG00000218891	0.797
hsa-miR-449a	CDKN1C	ENSG00000129757	0.727
hsa-miR-449a	SLC23A3	ENSG00000213901	0.745
hsa-miR-449a	RNF152	ENSG00000176641	0.877
hsa-miR-449a	MET	ENSG00000105976	0.367
hsa-miR-449a	CACNA1E	ENSG00000198216	0.825
hsa-miR-449a	NR4A2	ENSG00000153234	0.635
hsa-miR-449a	EML5	ENSG00000165521	0.605
hsa-miR-449a	NETO1	ENSG00000166342	0.536
hsa-miR-449a	TMEM250	ENSG00000238227	0.526
hsa-miR-449a	TMEM255A	ENSG00000125355	0.967

hsa-miR-454-3p	PRICKLE2	ENSG00000163637	0.825
hsa-miR-454-3p	RORB	ENSG00000198963	0.915
hsa-miR-454-3p	PPP1R9A	ENSG00000158528	0.915
hsa-miR-454-3p	SOX5	ENSG00000134532	0.946
hsa-miR-454-3p	PIGA	ENSG00000165195	0.608
hsa-miR-454-3p	KLHL15	ENSG00000174010	0.894
hsa-miR-454-3p	TMEM250	ENSG00000238227	0.539
hsa-miR-454-3p	NEXMIF	ENSG00000050030	0.776
hsa-miR-454-3p	FAM43A	ENSG00000185112	0.702
hsa-miR-454-3p	TMEM170B	ENSG00000205269	0.672
hsa-miR-454-3p	DEPDC1	ENSG00000024526	0.794
hsa-miR-454-3p	NRARP	ENSG00000198435	0.864
hsa-miR-454-3p	DIAPH3	ENSG00000139734	0.849
hsa-miR-454-3p	MET	ENSG00000105976	0.625
hsa-miR-454-3p	SH3D19	ENSG00000109686	0.422
hsa-miR-454-3p	TENM1	ENSG00000009694	0.97
hsa-miR-454-3p	PFKFB3	ENSG00000170525	0.868
hsa-miR-454-3p	SYNM	ENSG00000182253	0.843
hsa-miR-454-3p	COL9A3	ENSG00000092758	0.987
hsa-miR-454-3p	EREG	ENSG00000124882	0.411
hsa-miR-454-3p	SIK1	ENSG00000142178	0.803
hsa-miR-454-3p	CFL2	ENSG00000165410	0.909
hsa-miR-454-3p	RLIM	ENSG00000131263	0.815
hsa-miR-454-3p	PDE4D	ENSG00000113448	0.853
hsa-miR-490-3p	TMEM170B	ENSG00000205269	0.864
hsa-miR-490-3p	AURKA	ENSG00000087586	0.749
hsa-miR-504-5p	SDC1	ENSG00000115884	0.836
hsa-miR-504-5p	RORB	ENSG00000198963	0.861
hsa-miR-504-5p	NR4A3	ENSG00000119508	0.843
hsa-miR-504-5p	AOC3	ENSG00000131471	0.91
hsa-miR-504-5p	ZBTB43	ENSG00000169155	0.659
hsa-miR-504-5p	FZD7	ENSG00000155760	0.882
hsa-miR-506-3p	JAM2	ENSG00000154721	0.902
hsa-miR-506-3p	PPP1R9A	ENSG00000158528	0.863
hsa-miR-506-3p	RLIM	ENSG00000131263	0.679
hsa-miR-506-3p	PRLR	ENSG00000113494	0.902
hsa-miR-506-3p	GOLGA8M	ENSG00000188626	0.729
hsa-miR-506-3p	LRFN1	ENSG00000128011	0.903
hsa-miR-506-3p	CHD1	ENSG00000153922	0.72
hsa-miR-506-3p	CFL2	ENSG00000165410	0.646
hsa-miR-506-3p	YOD1	ENSG00000180667	0.657
hsa-miR-506-3p	EML5	ENSG00000165521	0.838
hsa-miR-506-3p	PAQR8	ENSG00000170915	0.833
hsa-miR-506-3p	PDE4D	ENSG00000113448	0.979
hsa-miR-506-3p	HIC1	ENSG00000177374	0.953
hsa-miR-506-3p	DEPDC1	ENSG00000024526	0.719
hsa-miR-506-3p	MOCS1	ENSG00000124615	0.98

hsa-miR-506-3p	TXNDC5	ENSG00000239264	0.845
hsa-miR-506-3p	MCM10	ENSG00000065328	0.871
hsa-miR-506-3p	NR1D2	ENSG00000174738	0.936
hsa-miR-506-3p	P4HA2	ENSG00000072682	0.791
hsa-miR-506-3p	PRICKLE2	ENSG00000163637	0.98
hsa-miR-506-3p	KCNJ2	ENSG00000123700	0.775
hsa-miR-590-5p	SOX5	ENSG00000134532	0.659
hsa-miR-590-5p	YOD1	ENSG00000180667	0.601
hsa-miR-590-5p	KLHL15	ENSG00000174010	0.962
hsa-miR-590-5p	FASLG	ENSG00000117560	0.444
hsa-miR-590-5p	RHOB	ENSG00000143878	0.912
hsa-miR-590-5p	PIK3R1	ENSG00000145675	0.893
hsa-miR-590-5p	DUSP8	ENSG00000184545	0.721
hsa-miR-590-5p	ESM1	ENSG00000164283	0.91
hsa-miR-708-5p	SRGAP1	ENSG00000196935	0.937
hsa-miR-708-5p	CD180	ENSG00000134061	1
hsa-miR-708-5p	OTOF	ENSG00000115155	0.814
hsa-miR-758-3p	DUSP1	ENSG00000120129	0.909
hsa-miR-758-3p	ZNF613	ENSG00000176024	0.78
hsa-miR-758-3p	RBFOX3	ENSG00000167281	0.884
hsa-miR-758-3p	CFL2	ENSG00000165410	0.972
hsa-miR-758-3p	SYNM	ENSG00000182253	0.953
hsa-miR-758-3p	OTX1	ENSG00000115507	0.961
hsa-miR-877-5p	RNF152	ENSG00000176641	0.833
hsa-miR-877-5p	OTOF	ENSG00000115155	0.98
hsa-miR-877-5p	NR4A3	ENSG00000119508	0.965
hsa-miR-9-5p	PRLR	ENSG00000113494	0.969
hsa-miR-9-5p	GJA3	ENSG00000121743	0.748
hsa-miR-9-5p	KCNJ2	ENSG00000123700	0.38
hsa-miR-9-5p	MCM10	ENSG00000065328	0.926
hsa-miR-9-5p	RORB	ENSG00000198963	0.901
hsa-miR-9-5p	TMEM170B	ENSG00000205269	0.791
hsa-miR-9-5p	SIK1	ENSG00000142178	0.752
hsa-miR-9-5p	ZBTB21	ENSG00000173276	0.924
hsa-miR-9-5p	TENM1	ENSG00000009694	0.944
hsa-miR-9-5p	SDC1	ENSG00000115884	0.836
hsa-miR-9-5p	PIM3	ENSG00000198355	0.865
hsa-miR-9-5p	TXNDC5	ENSG00000239264	0.865
hsa-miR-9-5p	EML5	ENSG00000165521	0.934
hsa-miR-9-5p	P4HA2	ENSG00000072682	0.689
hsa-miR-9-5p	CCSER1	ENSG00000184305	0.909
hsa-miR-9-5p	ISL2	ENSG00000159556	1
hsa-miR-9-5p	ARHGAP39	ENSG00000147799	0.98
hsa-miR-9-5p	HES1	ENSG00000114315	0.799
hsa-miR-9-5p	RANBP17	ENSG00000204764	0.466
hsa-miR-9-5p	PGAP1	ENSG00000197121	0.985
hsa-miR-9-5p	PDK4	ENSG00000004799	0.459

hsa-miR-9-5p	CACNA1E	ENSG00000198216	0.972
hsa-miR-9-5p	CCNT1	ENSG00000129315	0.9
hsa-miR-9-5p	HIC1	ENSG00000177374	0.819
hsa-miR-9-5p	DUSP8	ENSG00000184545	0.982
hsa-miR-92a-3p	SERTAD2	ENSG00000179833	0.805
hsa-miR-92a-3p	NETO1	ENSG00000166342	0.845
hsa-miR-92a-3p	SKOR1	ENSG00000188779	0.729
hsa-miR-92a-3p	ARHGAP29	ENSG00000137962	0.779
hsa-miR-92a-3p	TENM1	ENSG00000009694	0.97
hsa-miR-92a-3p	INAFM1	ENSG00000257704	0.793
hsa-miR-92a-3p	GOLGA8M	ENSG00000188626	0.78
hsa-miR-92a-3p	ARHGEF10	ENSG00000104728	0.743
hsa-miR-92a-3p	CDC42EP2	ENSG00000149798	0.802
hsa-miR-92a-3p	NR4A3	ENSG00000119508	0.672
hsa-miR-92a-3p	ARL5B	ENSG00000165997	0.957
hsa-miR-92a-3p	CHCHD10	ENSG00000250479	0.499
hsa-miR-92a-3p	GFPT2	ENSG00000131459	0.67
hsa-miR-92a-3p	TMEM255A	ENSG00000125355	0.707
hsa-miR-92a-3p	CDKN1C	ENSG00000129757	0.608
hsa-miR-92a-3p	FASLG	ENSG00000117560	0.771
hsa-miR-92a-3p	SH3D19	ENSG00000109686	0.811
hsa-miR-92a-3p	AURKA	ENSG00000087586	0.642
hsa-miR-92a-3p	PPP1R9A	ENSG00000158528	0.975
hsa-miR-92a-3p	SORCS3	ENSG00000156395	0.916
hsa-miR-92a-3p	SMAD6	ENSG00000137834	0.963
hsa-miR-92a-3p	DUSP1	ENSG00000120129	0.819
hsa-miR-92a-3p	KLHL15	ENSG00000174010	0.617
hsa-miR-92a-3p	CHST7	ENSG00000147119	0.54
hsa-miR-92a-3p	SIK1	ENSG00000142178	0.758
hsa-miR-92a-3p	KLF2	ENSG00000127528	0.503
hsa-miR-92a-3p	ZNF804A	ENSG00000170396	0.826
hsa-miR-92a-3p	ZBTB10	ENSG00000205189	0.98
hsa-miR-92a-3p	CFL2	ENSG00000165410	0.856
hsa-miR-92a-3p	ADRB1	ENSG00000043591	0.522
hsa-miR-93-5p	FEM1C	ENSG00000145780	0.647
hsa-miR-93-5p	YES1	ENSG00000176105	0.848
hsa-miR-93-5p	TNFSF11	ENSG00000120659	0.757
hsa-miR-93-5p	ZNF703	ENSG00000183779	1
hsa-miR-93-5p	KLHL15	ENSG00000174010	0.767
hsa-miR-93-5p	BTG3	ENSG00000154640	0.332
hsa-miR-93-5p	RLIM	ENSG00000131263	0.701
hsa-miR-93-5p	SYNM	ENSG00000182253	0.931
hsa-miR-93-5p	NR4A2	ENSG00000153234	0.725
hsa-miR-93-5p	SLC1A2	ENSG00000110436	0.98
hsa-miR-93-5p	AHRR	ENSG00000063438	0.983
hsa-miR-93-5p	OSM	ENSG00000099985	0.628
hsa-miR-93-5p	HIF1A	ENSG00000100644	0.796

hsa-miR-93-5p	SMAD6	ENSG00000137834	0.969
hsa-miR-93-5p	PER1	ENSG00000179094	0.964
hsa-miR-93-5p	SRGAP1	ENSG00000196935	0.894
hsa-miR-93-5p	CFL2	ENSG00000165410	0.559
hsa-miR-93-5p	TIPARP	ENSG00000163659	0.863
hsa-miR-93-5p	SKOR1	ENSG00000188779	0.707
hsa-miR-93-5p	ZBTB21	ENSG00000173276	0.91
hsa-miR-93-5p	SERTAD2	ENSG00000179833	0.825
hsa-miR-93-5p	MASTL	ENSG00000120539	0.476
hsa-miR-93-5p	PANX2	ENSG00000073150	0.805
hsa-miR-93-5p	ZNF805	ENSG00000204524	0.935
hsa-miR-93-5p	TENM1	ENSG00000009694	0.874
hsa-miR-93-5p	EREG	ENSG00000124882	0.693
hsa-miR-93-5p	NR4A3	ENSG00000119508	0.624
hsa-miR-93-5p	CXCL8	ENSG00000169429	0.572
hsa-miR-93-5p	SIK1	ENSG00000142178	0.72
hsa-miR-93-5p	ZBTB43	ENSG00000169155	0.799
hsa-miR-93-5p	PFKFB3	ENSG00000170525	0.822
hsa-miR-93-5p	RORB	ENSG00000198963	0.961
hsa-miR-93-5p	ARHGEF10	ENSG00000104728	0.773
hsa-miR-93-5p	CHST7	ENSG00000147119	0.999
hsa-miR-93-5p	YOD1	ENSG00000180667	0.852
hsa-miR-93-5p	PIK3R1	ENSG00000145675	0.981
hsa-miR-93-5p	DUSP8	ENSG00000184545	0.905
hsa-miR-98-5p	ZBTB10	ENSG00000205189	0.784
hsa-miR-98-5p	TNFAIP3	ENSG00000118503	0.887
hsa-miR-98-5p	YOD1	ENSG00000180667	0.205
hsa-miR-98-5p	ARL5B	ENSG00000165997	0.969
hsa-miR-98-5p	ADAMTS1	ENSG00000154734	0.823
hsa-miR-98-5p	P4HA2	ENSG00000072682	0.771
hsa-miR-98-5p	NAT8L	ENSG00000185818	0.686
hsa-miR-98-5p	PANX2	ENSG00000073150	0.758
hsa-miR-98-5p	TNFSF9	ENSG00000125657	0.363
hsa-miR-98-5p	IL13	ENSG00000169194	0.478
hsa-miR-98-5p	CACNA1E	ENSG00000198216	0.851
hsa-miR-98-5p	TMEM255A	ENSG00000125355	0.757
hsa-miR-98-5p	B3GNT7	ENSG00000156966	0.7
hsa-miR-98-5p	SRGAP1	ENSG00000196935	0.866
hsa-miR-98-5p	NIPAL4	ENSG00000172548	0.722
hsa-miR-98-5p	CFL2	ENSG00000165410	0.703
hsa-miR-98-5p	FASLG	ENSG00000117560	0.571
hsa-miR-98-5p	OTOF	ENSG00000115155	0.887
hsa-miR-98-5p	SOCS1	ENSG00000185338	0.583
hsa-miR-98-5p	SPRED3	ENSG00000188766	0.795
hsa-miR-98-5p	PRLR	ENSG00000113494	0.729
hsa-miR-98-5p	COL9A3	ENSG00000092758	0.727
hsa-miR-98-5p	PIGA	ENSG00000165195	0.589

hsa-miR-98-5p	CEBPD	ENSG00000221869	0.691
hsa-miR-98-5p	HIC1	ENSG00000177374	0.777
hsa-miR-98-5p	RNF152	ENSG00000176641	0.806
hsa-miR-98-5p	REL	ENSG00000162924	0.97
hsa-miR-98-5p	COL1A1	ENSG00000108821	0.727
hsa-miR-98-5p	FAM43A	ENSG00000185112	0.801
hsa-miR-98-5p	NEXMIF	ENSG00000050030	0.783
hsa-miR-98-5p	ABCB9	ENSG00000150967	0.556
hsa-miR-98-5p	DUSP1	ENSG00000120129	0.662
hsa-miR-98-5p	ADRB1	ENSG00000043591	0.633
hsa-miR-98-5p	YOD1	ENSG00000180667	1
hsa-miR-449a	TMEM255A	ENSG00000125355	1
hsa-miR-30c-5p	TMEM170B	ENSG00000205269	1
hsa-miR-548x-5p	TENM1	ENSG00000009694	1
hsa-miR-30c-5p	SOCS1	ENSG00000185338	1
hsa-miR-20a-5p	SKOR1	ENSG00000188779	1
hsa-miR-1299	SKA3	ENSG00000165480	1
hsa-miR-20a-5p	SIK1	ENSG00000142178	1
hsa-miR-153-3p	SERTAD2	ENSG00000179833	1
hsa-miR-129-5p	RBFOX3	ENSG00000167281	1
hsa-miR-513a-5p	PKD2L2	ENSG00000078795	1
hsa-miR-454-3p	MET	ENSG00000105976	1
hsa-miR-153-3p	GFPT2	ENSG00000131459	1
hsa-miR-20a-5p	CXCL8	ENSG00000169429	1
hsa-miR-20a-5p	BTG3	ENSG00000154640	1
hsa-miR-20a-5p	YOD1	ENSG00000180667	0.999
hsa-miR-21-5p	YOD1	ENSG00000180667	0.999
hsa-miR-147a	TCTE1	ENSG00000146221	0.999
hsa-miR-21-5p	SOX5	ENSG00000134532	0.999
hsa-miR-219a-5p	SOX5	ENSG00000134532	0.999
hsa-miR-513b-5p	SOX5	ENSG00000134532	0.999
hsa-miR-454-3p	SH3D19	ENSG00000109686	0.999
hsa-miR-30c-5p	RHOB	ENSG00000143878	0.999
hsa-miR-20a-5p	OSM	ENSG00000099985	0.999
hsa-miR-30c-5p	NR4A2	ENSG00000153234	0.999
hsa-miR-365a-3p	NCR3LG1	ENSG00000188211	0.999
hsa-miR-4717-5p	NCR3LG1	ENSG00000188211	0.999
hsa-miR-30c-5p	MYBL2	ENSG00000101057	0.999
hsa-miR-4775	LRG1	ENSG00000171236	0.999
hsa-miR-98-5p	IL13	ENSG00000169194	0.999
hsa-miR-101-3p	ZBTB21	ENSG00000173276	0.998
hsa-miR-374a-5p	YOD1	ENSG00000180667	0.998
hsa-miR-574-5p	TM4SF19	ENSG00000145107	0.998
hsa-miR-340-5p	SRGAP1	ENSG00000196935	0.998
hsa-miR-144-3p	SMAD9	ENSG00000120693	0.998
hsa-miR-92a-3p	SIK1	ENSG00000142178	0.998
hsa-miR-371b-5p	RLIM	ENSG00000131263	0.998

hsa-miR-616-5p	RLIM	ENSG00000131263	0.998
hsa-miR-144-3p	REL	ENSG00000162924	0.998
hsa-miR-1299	PRLR	ENSG00000113494	0.998
hsa-miR-193a-3p	PIGA	ENSG00000165195	0.998
hsa-miR-147a	NR4A2	ENSG00000153234	0.998
hsa-miR-125b-5p	NCR3LG1	ENSG00000188211	0.998
hsa-miR-20a-5p	NCR3LG1	ENSG00000188211	0.998
hsa-miR-9-5p	KCNJ2	ENSG00000123700	0.998
hsa-miR-153-3p	HEY2	ENSG00000135547	0.998
hsa-miR-30c-5p	GLDC	ENSG00000178445	0.998
hsa-miR-625-5p	GIMAP1	ENSG00000213203	0.998
hsa-miR-30c-5p	GFPT2	ENSG00000131459	0.998
hsa-miR-32-5p	GFPT2	ENSG00000131459	0.998
hsa-miR-101-3p	FEM1C	ENSG00000145780	0.998
hsa-miR-147a	C3orf35	ENSG00000198590	0.998
hsa-miR-340-5p	ADAMTS1	ENSG00000154734	0.998
hsa-miR-144-3p	ZBTB21	ENSG00000173276	0.997
hsa-miR-513a-5p	SLC1A2	ENSG00000110436	0.997
hsa-miR-548ah-3p	SERTAD2	ENSG00000179833	0.997
hsa-miR-101-3p	PDE4D	ENSG00000113448	0.997
hsa-miR-374a-5p	NCR3LG1	ENSG00000188211	0.997
hsa-miR-92a-3p	GFPT2	ENSG00000131459	0.997
hsa-miR-101-3p	DUSP1	ENSG00000120129	0.997
hsa-miR-147a	AOC1	ENSG00000002726	0.997
hsa-miR-590-5p	YOD1	ENSG00000180667	0.996
hsa-miR-9-5p	TENM1	ENSG00000009694	0.996
hsa-miR-4717-5p	KIAA1324	ENSG00000116299	0.996
hsa-miR-9-5p	GCSAM	ENSG00000174500	0.996
hsa-miR-30c-5p	EFNA3	ENSG00000143590	0.996
hsa-miR-624-5p	YES1	ENSG00000176105	0.995
hsa-miR-129-5p	TNFSF11	ENSG00000120659	0.995
hsa-miR-92a-3p	TMEM255A	ENSG00000125355	0.995
hsa-miR-144-3p	SORCS3	ENSG00000156395	0.995
hsa-miR-340-5p	NCR3LG1	ENSG00000188211	0.995
hsa-miR-551b-5p	ZNF805	ENSG00000204524	0.994
hsa-miR-9-5p	RGPD6	ENSG00000183054	0.994
hsa-miR-30c-5p	PIGA	ENSG00000165195	0.994
hsa-miR-548s	PIGA	ENSG00000165195	0.994
hsa-miR-340-5p	NEXMIF	ENSG00000050030	0.994
hsa-miR-548ah-3p	EREG	ENSG00000124882	0.994
hsa-miR-454-3p	DIAPH3	ENSG00000139734	0.994
hsa-miR-548ah-3p	SOX5	ENSG00000134532	0.993
hsa-miR-548x-5p	PRLR	ENSG00000113494	0.993
hsa-miR-193b-3p	PIGA	ENSG00000165195	0.993
hsa-miR-20a-5p	NR4A2	ENSG00000153234	0.993
hsa-miR-301a-3p	MET	ENSG00000105976	0.993
hsa-miR-513a-5p	ENC1	ENSG00000171617	0.993

hsa-miR-551b-5p	ZBTB21	ENSG00000173276	0.992
hsa-miR-4775	YOD1	ENSG00000180667	0.992
hsa-miR-506-3p	SERTAD2	ENSG00000179833	0.992
hsa-miR-17-5p	NR4A3	ENSG00000119508	0.992
hsa-miR-20a-5p	MASTL	ENSG00000120539	0.992
hsa-miR-758-3p	EML5	ENSG00000165521	0.992
hsa-miR-32-5p	ARL5B	ENSG00000165997	0.992
hsa-miR-30c-5p	YOD1	ENSG00000180667	0.991
hsa-miR-24-3p	TENM1	ENSG00000009694	0.991
hsa-miR-199b-5p	SORCS3	ENSG00000156395	0.991
hsa-miR-32-5p	SIK1	ENSG00000142178	0.991
hsa-miR-548ah-3p	PDE4D	ENSG00000113448	0.991
hsa-miR-1299	MANSC1	ENSG00000111261	0.991
hsa-miR-101-3p	FOS	ENSG00000170345	0.991
hsa-miR-30c-5p	FAM43A	ENSG00000185112	0.991
hsa-miR-24-3p	CITED4	ENSG00000179862	0.991
hsa-miR-206	ZNF547	ENSG00000152433	0.99
hsa-miR-30c-5p	TENM1	ENSG00000009694	0.99
hsa-miR-4423-5p	SKA3	ENSG00000165480	0.99
hsa-miR-92a-3p	SH3D19	ENSG00000109686	0.99
hsa-miR-500a-5p	SERTAD2	ENSG00000179833	0.99
hsa-miR-30c-5p	RTN4R	ENSG00000040608	0.99
hsa-miR-206	NEXMIF	ENSG00000050030	0.99
hsa-miR-374a-5p	NETO1	ENSG00000166342	0.99
hsa-miR-548ah-3p	KLHL15	ENSG00000174010	0.99
hsa-miR-20a-5p	FEM1C	ENSG00000145780	0.99
hsa-miR-4645-5p	EREG	ENSG00000124882	0.99
hsa-miR-92a-3p	ARL5B	ENSG00000165997	0.99
hsa-miR-218-5p	SERTAD2	ENSG00000179833	0.989
hsa-miR-9-5p	RGPD5	ENSG00000015568	0.989
hsa-miR-218-5p	PRLR	ENSG00000113494	0.989
hsa-miR-20a-5p	ARHGEF10	ENSG00000104728	0.989
hsa-miR-15b-5p	PIK3R1	ENSG00000145675	0.988
hsa-miR-20a-5p	NR4A3	ENSG00000119508	0.988
hsa-miR-3150a-3p	ICOSLG	ENSG00000160223	0.988
hsa-miR-548ah-3p	CCNT1	ENSG00000129315	0.988
hsa-miR-340-5p	ZBTB10	ENSG00000205189	0.987
hsa-miR-124-3p	SERTAD2	ENSG00000179833	0.987
hsa-miR-548a-5p	SERTAD2	ENSG00000179833	0.987
hsa-miR-15a-5p	PIK3R1	ENSG00000145675	0.987
hsa-miR-374a-5p	PGAP1	ENSG00000197121	0.987
hsa-miR-340-5p	KLHL15	ENSG00000174010	0.987
hsa-miR-193a-3p	KCNJ2	ENSG00000123700	0.987
hsa-miR-98-5p	IFITM10	ENSG00000244242	0.987
hsa-miR-374a-5p	FPR3	ENSG00000187474	0.987
hsa-miR-454-3p	EREG	ENSG00000124882	0.987
hsa-miR-628-3p	ADAMTS1	ENSG00000154734	0.987

hsa-miR-92a-3p	ZNF804A	ENSG00000170396	0.986
hsa-miR-147a	TREML2	ENSG00000112195	0.986
hsa-miR-144-3p	TNFSF11	ENSG00000120659	0.986
hsa-miR-1972	TNFAIP3	ENSG00000118503	0.986
hsa-miR-548ah-3p	SIK1	ENSG00000142178	0.986
hsa-miR-432-5p	RASGRF1	ENSG00000058335	0.986
hsa-miR-30c-5p	PRLR	ENSG00000113494	0.986
hsa-miR-340-5p	NRARP	ENSG00000198435	0.986
hsa-miR-3074-5p	NR4A3	ENSG00000119508	0.986
hsa-miR-93-5p	NR4A3	ENSG00000119508	0.986
hsa-miR-513b-5p	NEXMIF	ENSG00000050030	0.986
hsa-miR-548ah-5p	NCR3LG1	ENSG00000188211	0.986
hsa-miR-548ah-3p	KCNJ2	ENSG00000123700	0.986
hsa-miR-4775	FEM1C	ENSG00000145780	0.986
hsa-miR-513b-5p	FASLG	ENSG00000117560	0.986
hsa-miR-20a-5p	CFL2	ENSG00000165410	0.986
hsa-miR-129-5p	CDKN1C	ENSG00000129757	0.986
hsa-miR-4775	ARL5B	ENSG00000165997	0.986
hsa-miR-98-5p	TNFSF9	ENSG00000125657	0.985
hsa-miR-124-3p	PGAP1	ENSG00000197121	0.985
hsa-miR-548ai	NCR3LG1	ENSG00000188211	0.985
hsa-miR-32-5p	KLF2	ENSG00000127528	0.985
hsa-miR-92a-3p	ZBTB10	ENSG00000205189	0.984
hsa-miR-24-3p	U2AF1	ENSG00000160201	0.984
hsa-miR-101-3p	SRGAP1	ENSG00000196935	0.984
hsa-miR-625-5p	SERTAD2	ENSG00000179833	0.984
hsa-miR-3074-5p	PDE4D	ENSG00000113448	0.984
hsa-miR-10a-5p	NR4A3	ENSG00000119508	0.984
hsa-miR-513b-5p	FA2H	ENSG00000103089	0.984
hsa-miR-449a	CACNA1E	ENSG00000198216	0.984
hsa-miR-3150a-3p	ARHGAP39	ENSG00000147799	0.984
hsa-miR-32-5p	ZBTB10	ENSG00000205189	0.983
hsa-miR-628-3p	NEXMIF	ENSG00000050030	0.983
hsa-miR-30c-5p	CFL2	ENSG00000165410	0.983
hsa-miR-664b-3p	YOD1	ENSG00000180667	0.982
hsa-miR-340-5p	PDE4D	ENSG00000113448	0.982
hsa-miR-4775	NR1D2	ENSG00000174738	0.982
hsa-miR-125b-5p	FPR3	ENSG00000187474	0.982
hsa-miR-301a-3p	EREG	ENSG00000124882	0.982
hsa-miR-10a-5p	TMEM170B	ENSG00000205269	0.981
hsa-miR-551b-5p	PPP1R9A	ENSG00000158528	0.981
hsa-miR-548x-5p	NR4A3	ENSG00000119508	0.981
hsa-miR-513a-5p	GALNT5	ENSG00000136542	0.981
hsa-miR-548s	EML5	ENSG00000165521	0.981
hsa-miR-199a-5p	SORCS3	ENSG00000156395	0.98
hsa-miR-301a-3p	PIGA	ENSG00000165195	0.98
hsa-miR-9-5p	PDK4	ENSG00000004799	0.98

hsa-miR-1299	NCR3LG1	ENSG00000188211	0.98
hsa-miR-30c-5p	GRM3	ENSG00000198822	0.98
hsa-miR-340-5p	SIAH1	ENSG00000196470	0.979
hsa-miR-21-5p	FASLG	ENSG00000117560	0.979
hsa-miR-153-3p	EFNA3	ENSG00000143590	0.979
hsa-miR-4775	E2F8	ENSG00000129173	0.979
hsa-miR-664a-3p	TMEM255A	ENSG00000125355	0.978
hsa-miR-24-3p	SCML1	ENSG00000047634	0.978
hsa-miR-4775	NEXMIF	ENSG00000050030	0.978
hsa-miR-188-5p	STEAP4	ENSG00000127954	0.977
hsa-miR-340-5p	PGAP1	ENSG00000197121	0.977
hsa-miR-506-3p	PDE4D	ENSG00000113448	0.977
hsa-miR-3064-5p	NCR3LG1	ENSG00000188211	0.977
hsa-miR-548a-5p	FZD7	ENSG00000155760	0.977
hsa-miR-340-5p	YOD1	ENSG00000180667	0.976
hsa-miR-30c-5p	NCR3LG1	ENSG00000188211	0.976
hsa-miR-21-5p	KLHL15	ENSG00000174010	0.976
hsa-miR-92a-3p	KLF2	ENSG00000127528	0.976
hsa-miR-432-5p	KIAA1324	ENSG00000116299	0.976
hsa-miR-548ah-3p	GCSAML	ENSG00000169224	0.976
hsa-miR-340-5p	CCSER1	ENSG00000184305	0.976
hsa-miR-125b-5p	TNFAIP3	ENSG00000118503	0.975
hsa-miR-144-3p	PDE4D	ENSG00000113448	0.975
hsa-miR-548a-5p	HEY2	ENSG00000135547	0.975
hsa-miR-147a	E2F8	ENSG00000129173	0.975
hsa-miR-548x-5p	TMEM255A	ENSG00000125355	0.974
hsa-miR-32-5p	SKOR1	ENSG00000188779	0.974
hsa-miR-20a-5p	SERTAD2	ENSG00000179833	0.974
hsa-miR-551b-5p	RLIM	ENSG00000131263	0.974
hsa-miR-506-3p	PGAP1	ENSG00000197121	0.974
hsa-miR-1303	OTOF	ENSG00000115155	0.974
hsa-miR-129-5p	NCR3LG1	ENSG00000188211	0.974
hsa-miR-365b-5p	NCR3LG1	ENSG00000188211	0.974
hsa-miR-129-5p	ZBTB10	ENSG00000205189	0.973
hsa-miR-4775	TNFSF11	ENSG00000120659	0.973
hsa-miR-501-5p	NEXMIF	ENSG00000050030	0.973
hsa-miR-301a-3p	FAM43A	ENSG00000185112	0.973
hsa-miR-548ah-3p	BTG3	ENSG00000154640	0.973
hsa-miR-548ah-3p	ZNF805	ENSG00000204524	0.972
hsa-miR-551b-5p	YOD1	ENSG00000180667	0.972
hsa-miR-551b-5p	RNF152	ENSG00000176641	0.972
hsa-miR-500a-5p	ZNF805	ENSG00000204524	0.971
hsa-miR-664a-3p	ZNF781	ENSG00000196381	0.971
hsa-miR-340-5p	ZBTB21	ENSG00000173276	0.971
hsa-miR-32-5p	SERTAD2	ENSG00000179833	0.971
hsa-miR-501-5p	NR4A3	ENSG00000119508	0.971
hsa-miR-9-5p	NCR3LG1	ENSG00000188211	0.971

hsa-miR-664a-3p	NAT2	ENSG00000156006	0.971
hsa-miR-548ah-3p	COL1A1	ENSG00000108821	0.971
hsa-miR-548ah-3p	YES1	ENSG00000176105	0.97
hsa-miR-4482-5p	RORB	ENSG00000198963	0.97
hsa-miR-124-3p	PDE4D	ENSG00000113448	0.97
hsa-miR-3613-5p	NCR3LG1	ENSG00000188211	0.97
hsa-miR-365a-5p	NCR3LG1	ENSG00000188211	0.97
hsa-miR-664a-3p	LRRIQ3	ENSG00000162620	0.97
hsa-miR-500a-5p	TMEM170B	ENSG00000205269	0.969
hsa-miR-301a-3p	SH3D19	ENSG00000109686	0.969
hsa-miR-32-5p	SH3D19	ENSG00000109686	0.969
hsa-miR-30c-5p	P4HA2	ENSG00000072682	0.969
hsa-miR-103a-3p	SRGAP1	ENSG00000196935	0.968
hsa-miR-539-5p	SLC1A2	ENSG00000110436	0.968
hsa-miR-548a-5p	SCML1	ENSG00000047634	0.968
hsa-miR-506-3p	MOCS1	ENSG00000124615	0.968
hsa-miR-24-3p	KCNJ2	ENSG00000123700	0.968
hsa-miR-513b-5p	IER5L	ENSG00000188483	0.968
hsa-miR-374a-5p	CEBPB	ENSG00000172216	0.968
hsa-miR-548ah-3p	ARHGAP29	ENSG00000137962	0.968
hsa-miR-1299	ZNF331	ENSG00000130844	0.967
hsa-miR-340-5p	TMEM170B	ENSG00000205269	0.967
hsa-miR-153-3p	GRIK4	ENSG00000149403	0.967
hsa-miR-4775	CFL2	ENSG00000165410	0.967
hsa-miR-20a-5p	ZBTB21	ENSG00000173276	0.966
hsa-miR-30c-5p	SCML1	ENSG00000047634	0.966
hsa-miR-449a	MET	ENSG00000105976	0.966
hsa-miR-32-5p	KLHL15	ENSG00000174010	0.966
hsa-miR-500a-3p	ZBTB43	ENSG00000169155	0.965
hsa-miR-664a-3p	YES1	ENSG00000176105	0.965
hsa-miR-4775	TMEM255A	ENSG00000125355	0.965
hsa-miR-539-5p	SOX5	ENSG00000134532	0.965
hsa-miR-660-5p	SOX5	ENSG00000134532	0.965
hsa-miR-129-5p	NEXMIF	ENSG00000050030	0.965
hsa-miR-124-3p	MOCS1	ENSG00000124615	0.965
hsa-miR-340-5p	IER5L	ENSG00000188483	0.965
hsa-miR-144-3p	E2F8	ENSG00000129173	0.965
hsa-miR-30c-5p	DBF4	ENSG00000006634	0.965
hsa-miR-20a-5p	ZNF805	ENSG00000204524	0.964
hsa-miR-664b-3p	ZBTB10	ENSG00000205189	0.964
hsa-miR-153-3p	PIK3R1	ENSG00000145675	0.964
hsa-miR-101-3p	NEXMIF	ENSG00000050030	0.964
hsa-miR-4775	KCNJ2	ENSG00000123700	0.964
hsa-miR-548ah-5p	YES1	ENSG00000176105	0.963
hsa-miR-340-5p	SLC1A2	ENSG00000110436	0.963
hsa-miR-664a-3p	GCSAML	ENSG00000169224	0.963
hsa-miR-616-5p	ZNF805	ENSG00000204524	0.962

hsa-miR-548a-5p	PRLR	ENSG00000113494	0.962
hsa-miR-92a-3p	PPP1R9A	ENSG00000158528	0.962
hsa-miR-187-3p	GRIN1	ENSG00000176884	0.962
hsa-miR-3180-5p	EML5	ENSG00000165521	0.962
hsa-miR-32-5p	ZNF804A	ENSG00000170396	0.961
hsa-miR-374a-5p	SMAD6	ENSG00000137834	0.961
hsa-miR-129-5p	SIAH1	ENSG00000196470	0.961
hsa-miR-1228-3p	RBFOX3	ENSG00000167281	0.961
hsa-miR-193b-3p	KLHL15	ENSG00000174010	0.961
hsa-miR-3074-5p	EML5	ENSG00000165521	0.961
hsa-miR-513b-5p	ZBTB21	ENSG00000173276	0.96
hsa-miR-548a-5p	SOX5	ENSG00000134532	0.96
hsa-miR-24-3p	RBM11	ENSG00000185272	0.96
hsa-miR-664b-3p	PRLR	ENSG00000113494	0.96
hsa-miR-4659b-5p	NCR3LG1	ENSG00000188211	0.96
hsa-miR-3074-5p	MET	ENSG00000105976	0.96
hsa-miR-548x-5p	KCNJ2	ENSG00000123700	0.96
hsa-miR-548ah-5p	GJA3	ENSG00000121743	0.96
hsa-miR-32-5p	CDKN1C	ENSG00000129757	0.96
hsa-miR-17-5p	SRGAP1	ENSG00000196935	0.959
hsa-miR-590-5p	SOX5	ENSG00000134532	0.959
hsa-miR-9-5p	RANBP17	ENSG00000204764	0.959
hsa-miR-3150a-3p	NCR3LG1	ENSG00000188211	0.959
hsa-miR-301a-3p	DIAPH3	ENSG00000139734	0.959
hsa-miR-625-5p	COL1A1	ENSG00000108821	0.959
hsa-miR-664b-3p	NR4A3	ENSG00000119508	0.958
hsa-miR-9-5p	CCNT1	ENSG00000129315	0.958
hsa-miR-144-3p	TENM1	ENSG00000009694	0.957
hsa-miR-20a-5p	SRGAP1	ENSG00000196935	0.957
hsa-miR-4645-5p	OSM	ENSG00000099985	0.957
hsa-miR-103a-2-5p	IFITM10	ENSG00000244242	0.957
hsa-miR-340-5p	ZBED6	ENSG00000257315	0.956
hsa-miR-93-5p	STBD1	ENSG00000118804	0.956
hsa-miR-1299	RLIM	ENSG00000131263	0.956
hsa-miR-30c-5p	RASGEF1B	ENSG00000138670	0.956
hsa-miR-32-5p	PPP1R9A	ENSG00000158528	0.956
hsa-miR-454-3p	PIGA	ENSG00000165195	0.956
hsa-miR-664b-3p	CD180	ENSG00000134061	0.956
hsa-miR-340-5p	SOX5	ENSG00000134532	0.955
hsa-miR-92a-3p	KLHL15	ENSG00000174010	0.955
hsa-miR-548ah-3p	ARL5B	ENSG00000165997	0.955
hsa-miR-190a-5p	TENM1	ENSG00000009694	0.954
hsa-miR-218-5p	SOX5	ENSG00000134532	0.954
hsa-miR-4645-5p	SOCS1	ENSG00000185338	0.954
hsa-miR-1972	SCARF2	ENSG00000244486	0.954
hsa-miR-513a-5p	GCSAM	ENSG00000174500	0.954
hsa-miR-548ah-5p	DGAT2	ENSG00000062282	0.954

hsa-miR-125b-5p	BORCS6	ENSG00000196544	0.954
hsa-miR-340-5p	ARL5B	ENSG00000165997	0.954
hsa-miR-340-5p	ZNF805	ENSG00000204524	0.953
hsa-miR-129-5p	PPP1R9A	ENSG00000158528	0.953
hsa-miR-548ah-3p	PDK4	ENSG00000004799	0.953
hsa-miR-664a-3p	CXCL8	ENSG00000169429	0.953
hsa-miR-513b-5p	ZBTB43	ENSG00000169155	0.952
hsa-miR-664a-3p	RASGRF1	ENSG00000058335	0.952
hsa-miR-340-5p	PRLR	ENSG00000113494	0.952
hsa-miR-1303	MANSC1	ENSG00000111261	0.952
hsa-miR-590-5p	FASLG	ENSG00000117560	0.952
hsa-miR-153-3p	CFL2	ENSG00000165410	0.952
hsa-miR-548a-5p	RASGEF1B	ENSG00000138670	0.951
hsa-miR-548a-5p	PDE4D	ENSG00000113448	0.951
hsa-miR-506-3p	JAM2	ENSG00000154721	0.951
hsa-miR-21-5p	DUSP8	ENSG00000184545	0.951
hsa-miR-340-5p	DBF4	ENSG00000006634	0.951
hsa-miR-1299	CCDC173	ENSG00000154479	0.951
hsa-miR-548a-5p	TENM1	ENSG00000009694	0.95
hsa-miR-20a-5p	STBD1	ENSG00000118804	0.95
hsa-miR-548ah-3p	SORCS3	ENSG00000156395	0.95
hsa-miR-15a-5p	BORCS6	ENSG00000196544	0.95
hsa-miR-190b	ADGRE3	ENSG00000131355	0.95
hsa-miR-548a-5p	ADAMTS1	ENSG00000154734	0.95
hsa-miR-371b-5p	ZNF805	ENSG00000204524	0.949
hsa-miR-4494	SRGAP1	ENSG00000196935	0.949
hsa-miR-548ah-3p	RHOB	ENSG00000143878	0.949
hsa-miR-506-3p	LRFN1	ENSG00000128011	0.949
hsa-miR-15b-5p	KCNJ2	ENSG00000123700	0.949
hsa-miR-15b-5p	BORCS6	ENSG00000196544	0.949
hsa-miR-340-5p	TNFAIP3	ENSG00000118503	0.948
hsa-miR-664b-3p	TMEM255A	ENSG00000125355	0.948
hsa-miR-548ah-3p	RLIM	ENSG00000131263	0.948
hsa-miR-199a-5p	P4HA2	ENSG00000072682	0.948
hsa-miR-548ah-3p	SLC1A2	ENSG00000110436	0.947
hsa-miR-30c-5p	PDE4D	ENSG00000113448	0.947
hsa-miR-129-5p	NR4A2	ENSG00000153234	0.947
hsa-miR-124-3p	JAM2	ENSG00000154721	0.947
hsa-miR-4775	ARHGEF10	ENSG00000104728	0.947
hsa-miR-4775	TM4SF19	ENSG00000145107	0.946
hsa-miR-10a-5p	RLIM	ENSG00000131263	0.946
hsa-miR-513b-5p	PGAP1	ENSG00000197121	0.946
hsa-miR-103a-3p	PDK4	ENSG00000004799	0.946
hsa-miR-513a-5p	CD83	ENSG00000112149	0.946
hsa-miR-340-5p	BTG3	ENSG00000154640	0.946
hsa-miR-93-5p	SRGAP1	ENSG00000196935	0.945
hsa-miR-548ah-3p	SIAH1	ENSG00000196470	0.945

hsa-miR-548ai	PPP1R9A	ENSG00000158528	0.945
hsa-miR-92a-3p	NR4A3	ENSG00000119508	0.945
hsa-miR-301a-3p	MASTL	ENSG00000120539	0.945
hsa-miR-21-5p	LRRIQ3	ENSG00000162620	0.945
hsa-miR-15a-5p	KCNJ2	ENSG00000123700	0.945
hsa-miR-1299	GJA3	ENSG00000121743	0.945
hsa-miR-4775	TMEM170B	ENSG00000205269	0.944
hsa-miR-664a-3p	SLC1A2	ENSG00000110436	0.944
hsa-miR-17-3p	RLIM	ENSG00000131263	0.944
hsa-miR-129-5p	PIK3R1	ENSG00000145675	0.944
hsa-miR-1299	NDUFV2	ENSG00000178127	0.944
hsa-miR-17-5p	STBD1	ENSG00000118804	0.943
hsa-miR-3605-5p	RNF152	ENSG00000176641	0.943
hsa-miR-20a-5p	RLIM	ENSG00000131263	0.943
hsa-miR-92a-3p	SERTAD2	ENSG00000179833	0.942
hsa-miR-10a-5p	SDC1	ENSG00000115884	0.942
hsa-miR-551b-5p	PRLR	ENSG00000113494	0.942
hsa-miR-204-5p	PPP1R9A	ENSG00000158528	0.942
hsa-miR-199b-5p	P4HA2	ENSG00000072682	0.942
hsa-miR-374a-5p	NEXMIF	ENSG00000050030	0.942
hsa-miR-625-5p	CX3CR1	ENSG00000168329	0.942
hsa-miR-129-5p	SLC1A2	ENSG00000110436	0.941
hsa-miR-548ah-3p	PIK3R1	ENSG00000145675	0.941
hsa-miR-4423-5p	GIMAP1	ENSG00000213203	0.941
hsa-miR-15a-5p	RLIM	ENSG00000131263	0.94
hsa-miR-4775	LRRIQ3	ENSG00000162620	0.94
hsa-miR-196a-5p	KCNJ2	ENSG00000123700	0.94
hsa-miR-340-5p	FEM1C	ENSG00000145780	0.94
hsa-miR-432-5p	RASGEF1B	ENSG00000138670	0.939
hsa-miR-340-5p	MET	ENSG00000105976	0.939
hsa-miR-432-5p	E2F8	ENSG00000129173	0.939
hsa-miR-190a-5p	ADGRE3	ENSG00000131355	0.938
hsa-miR-4645-5p	IFITM10	ENSG00000244242	0.937
hsa-miR-551b-5p	TMEM170B	ENSG00000205269	0.936
hsa-miR-548ah-5p	PGAP1	ENSG00000197121	0.936
hsa-miR-15a-5p	NCR3LG1	ENSG00000188211	0.936
hsa-miR-3150a-3p	COL1A1	ENSG00000108821	0.936
hsa-miR-513a-5p	ACKR4	ENSG00000129048	0.936
hsa-miR-432-5p	CD83	ENSG00000112149	0.935
hsa-miR-103a-3p	BTN1A1	ENSG00000124557	0.935
hsa-miR-513b-5p	RANBP17	ENSG00000204764	0.934
hsa-miR-4775	KLHL15	ENSG00000174010	0.934
hsa-miR-374a-5p	HES1	ENSG00000114315	0.934
hsa-miR-24-3p	CISH	ENSG00000114737	0.934
hsa-miR-1299	CACNA1E	ENSG00000198216	0.934
hsa-miR-190b	TENM1	ENSG00000009694	0.933
hsa-miR-93-5p	SERTAD2	ENSG00000179833	0.933

hsa-miR-340-5p	NR1D2	ENSG00000174738	0.933
hsa-miR-548ai	NR1D2	ENSG00000174738	0.933
hsa-miR-3144-5p	COL1A1	ENSG00000108821	0.933
hsa-miR-340-5p	TLR10	ENSG00000174123	0.932
hsa-miR-539-5p	TENM1	ENSG00000009694	0.932
hsa-miR-15b-5p	RLIM	ENSG00000131263	0.932
hsa-miR-340-5p	NR4A2	ENSG00000153234	0.932
hsa-miR-15b-5p	NCR3LG1	ENSG00000188211	0.932
hsa-miR-548ah-3p	CCSER1	ENSG00000184305	0.932
hsa-miR-4717-5p	TNFAIP3	ENSG00000118503	0.931
hsa-miR-616-5p	SERTAD2	ENSG00000179833	0.931
hsa-miR-4775	PRLR	ENSG00000113494	0.931
hsa-miR-548ah-3p	HEY2	ENSG00000135547	0.931
hsa-miR-548ah-5p	GIMAP1	ENSG00000213203	0.931
hsa-miR-4659b-5p	CCSER1	ENSG00000184305	0.931
hsa-miR-513b-5p	ZNF804A	ENSG00000170396	0.93
hsa-miR-506-3p	TNFSF11	ENSG00000120659	0.93
hsa-miR-628-3p	LINGO2	ENSG00000174482	0.93
hsa-miR-432-5p	DEPDC1	ENSG00000024526	0.93
hsa-miR-664a-3p	CFL2	ENSG00000165410	0.93
hsa-miR-3150a-3p	CCR6	ENSG00000112486	0.93
hsa-miR-371b-5p	BTG3	ENSG00000154640	0.93
hsa-miR-4717-5p	ATP1B2	ENSG00000129244	0.93
hsa-miR-454-3p	TMEM250	ENSG00000238227	0.929
hsa-miR-449a	TENM1	ENSG00000009694	0.929
hsa-miR-92b-5p	SLC7A5	ENSG00000103257	0.929
hsa-miR-9-5p	PRLR	ENSG00000113494	0.929
hsa-miR-3150a-3p	PER1	ENSG00000179094	0.929
hsa-miR-1299	MCOLN3	ENSG00000055732	0.929
hsa-miR-548s	SCARF2	ENSG00000244486	0.928
hsa-miR-708-5p	PKD2L2	ENSG00000078795	0.928
hsa-miR-24-3p	NCR3LG1	ENSG00000188211	0.928
hsa-miR-548x-5p	FAM217A	ENSG00000145975	0.927
hsa-miR-92a-3p	AURKA	ENSG00000087586	0.927
hsa-miR-92a-3p	ADRB1	ENSG00000043591	0.927
hsa-miR-193a-5p	ZNF628	ENSG00000197483	0.926
hsa-miR-196a-5p	PFKFB3	ENSG00000170525	0.926
hsa-miR-371a-3p	NCR3LG1	ENSG00000188211	0.926
hsa-miR-20a-5p	KLHL15	ENSG00000174010	0.926
hsa-miR-24-3p	FASLG	ENSG00000117560	0.926
hsa-miR-93-5p	ARHGEF10	ENSG00000104728	0.926
hsa-miR-4717-5p	YES1	ENSG00000176105	0.925
hsa-miR-21-5p	SC5D	ENSG00000109929	0.925
hsa-miR-548ah-3p	PIGA	ENSG00000165195	0.925
hsa-miR-548s	NCR3LG1	ENSG00000188211	0.925
hsa-miR-365a-3p	KCNJ2	ENSG00000123700	0.925
hsa-miR-548x-5p	FAM43A	ENSG00000185112	0.925

hsa-miR-103a-3p	CDKN1C	ENSG00000129757	0.925
hsa-miR-204-5p	ADGRE3	ENSG00000131355	0.925
hsa-miR-513a-5p	ZBTB10	ENSG00000205189	0.924
hsa-miR-548ah-3p	ZBTB10	ENSG00000205189	0.924
hsa-miR-30c-5p	SLC1A2	ENSG00000110436	0.924
hsa-miR-17-5p	SERTAD2	ENSG00000179833	0.924
hsa-miR-4775	PDE4D	ENSG00000113448	0.924
hsa-miR-664b-3p	RASGEF1B	ENSG00000138670	0.923
hsa-miR-10a-5p	PRLR	ENSG00000113494	0.923
hsa-miR-129-5p	PGAP1	ENSG00000197121	0.923
hsa-miR-548a-5p	PGAP1	ENSG00000197121	0.923
hsa-miR-513a-5p	BTN1A1	ENSG00000124557	0.923
hsa-miR-98-5p	ARHGAP8	ENSG00000241484	0.923
hsa-miR-193b-3p	KCNJ2	ENSG00000123700	0.922
hsa-miR-4659b-5p	KCNJ2	ENSG00000123700	0.922
hsa-miR-32-5p	IFIT2	ENSG00000119922	0.922
hsa-miR-129-5p	IER5L	ENSG00000188483	0.922
hsa-miR-30c-5p	GCSAM	ENSG00000174500	0.922
hsa-miR-92a-3p	CHST7	ENSG00000147119	0.922
hsa-miR-504-5p	YES1	ENSG00000176105	0.921
hsa-miR-500a-3p	TMEM170B	ENSG00000205269	0.921
hsa-miR-144-3p	RASGRF1	ENSG00000058335	0.921
hsa-miR-490-3p	AURKA	ENSG00000087586	0.921
hsa-miR-30a-3p	TENM1	ENSG00000009694	0.92
hsa-miR-548x-5p	RBM11	ENSG00000185272	0.92
hsa-miR-124-5p	RANBP17	ENSG00000204764	0.92
hsa-miR-98-5p	NCR3LG1	ENSG00000188211	0.92
hsa-miR-3180	JUND	ENSG00000130522	0.92
hsa-miR-1299	ZNF285	ENSG00000267508	0.919
hsa-miR-196a-5p	TKTL2	ENSG00000151005	0.919
hsa-miR-551b-5p	KLHL15	ENSG00000174010	0.919
hsa-miR-574-5p	C3orf35	ENSG00000198590	0.919
hsa-miR-513a-5p	TMEM170B	ENSG00000205269	0.918
hsa-miR-340-5p	SERTAD2	ENSG00000179833	0.918
hsa-miR-454-3p	MASTL	ENSG00000120539	0.918
hsa-miR-153-3p	IDO1	ENSG00000131203	0.918
hsa-miR-153-3p	SCML1	ENSG00000047634	0.917
hsa-miR-885-5p	DEPDC1	ENSG00000024526	0.917
hsa-miR-548a-5p	ARL5B	ENSG00000165997	0.917
hsa-miR-32-5p	ARHGEF10	ENSG00000104728	0.917
hsa-miR-103a-3p	ZBTB10	ENSG00000205189	0.916
hsa-miR-548ah-3p	TMEM170B	ENSG00000205269	0.916
hsa-miR-153-3p	PIGA	ENSG00000165195	0.916
hsa-miR-3150a-3p	NETO1	ENSG00000166342	0.916
hsa-miR-1299	KCNJ2	ENSG00000123700	0.916
hsa-miR-98-5p	ERRFI1	ENSG00000116285	0.916
hsa-miR-664a-3p	YOD1	ENSG00000180667	0.915

hsa-miR-502-5p	TNFRSF9	ENSG00000049249	0.915
hsa-miR-147a	TIPARP	ENSG00000163659	0.915
hsa-miR-17-3p	TENM1	ENSG00000009694	0.915
hsa-miR-539-5p	SYN1	ENSG00000008056	0.915
hsa-miR-624-5p	SOX5	ENSG00000134532	0.915
hsa-miR-199a-5p	RASGEF1B	ENSG00000138670	0.915
hsa-miR-513a-5p	PPP1R9A	ENSG00000158528	0.915
hsa-miR-4645-5p	NCR3LG1	ENSG00000188211	0.915
hsa-miR-4775	DBF4	ENSG00000006634	0.915
hsa-miR-362-5p	ZNF805	ENSG00000204524	0.914
hsa-miR-628-5p	SH3D19	ENSG00000109686	0.914
hsa-miR-548ah-5p	SERTAD2	ENSG00000179833	0.914
hsa-miR-628-5p	ERRFI1	ENSG00000116285	0.914
hsa-miR-101-3p	ATP1B2	ENSG00000129244	0.914
hsa-miR-124-5p	TMEM170B	ENSG00000205269	0.913
hsa-miR-204-5p	STEAP4	ENSG00000127954	0.913
hsa-miR-301a-3p	STEAP4	ENSG00000127954	0.913
hsa-miR-3064-5p	SLC23A3	ENSG00000213901	0.913
hsa-miR-362-5p	SERTAD2	ENSG00000179833	0.913
hsa-miR-1303	RNF152	ENSG00000176641	0.913
hsa-miR-101-3p	PRLR	ENSG00000113494	0.913
hsa-miR-625-5p	PRLR	ENSG00000113494	0.913
hsa-miR-374a-5p	TNFAIP3	ENSG00000118503	0.912
hsa-miR-548a-5p	NR4A3	ENSG00000119508	0.912
hsa-miR-340-5p	KCNJ2	ENSG00000123700	0.912
hsa-miR-664a-3p	FAM81B	ENSG00000153347	0.912
hsa-miR-125b-5p	CTU1	ENSG00000142544	0.912
hsa-miR-616-5p	CFL2	ENSG00000165410	0.912
hsa-miR-3074-3p	ZNF804A	ENSG00000170396	0.911
hsa-miR-340-5p	STEAP4	ENSG00000127954	0.911
hsa-miR-153-3p	SLC26A5	ENSG00000170615	0.911
hsa-miR-660-5p	KCNJ2	ENSG00000123700	0.911
hsa-miR-664b-3p	DIAPH3	ENSG00000139734	0.911
hsa-miR-362-5p	CCSER1	ENSG00000184305	0.911
hsa-miR-15a-5p	CACNA1E	ENSG00000198216	0.911
hsa-miR-30c-5p	C4orf19	ENSG00000154274	0.911
hsa-miR-574-5p	BATF2	ENSG00000168062	0.911
hsa-miR-24-3p	RANBP17	ENSG00000204764	0.91
hsa-miR-664b-3p	NEXMIF	ENSG00000050030	0.91
hsa-miR-122-5p	BEND5	ENSG00000162373	0.91
hsa-miR-664b-3p	PRICKLE2	ENSG00000163637	0.909
hsa-miR-539-5p	GALNT5	ENSG00000136542	0.909
hsa-miR-432-5p	CCDC74A	ENSG00000163040	0.909
hsa-miR-548ah-3p	BIRC5	ENSG00000089685	0.909
hsa-miR-4775	YES1	ENSG00000176105	0.908
hsa-miR-574-5p	TCTE1	ENSG00000146221	0.908
hsa-miR-548ah-5p	RLIM	ENSG00000131263	0.908

hsa-miR-3074-5p	RGPD6	ENSG00000183054	0.908
hsa-miR-193a-3p	EML5	ENSG00000165521	0.908
hsa-miR-548a-5p	CFL2	ENSG00000165410	0.908
hsa-miR-17-5p	ARHGEF10	ENSG00000104728	0.908
hsa-miR-9-5p	ZBTB21	ENSG00000173276	0.907
hsa-miR-193a-3p	KLHL15	ENSG00000174010	0.907
hsa-miR-548a-5p	HES1	ENSG00000114315	0.907
hsa-miR-664b-3p	GCSAML	ENSG00000169224	0.907
hsa-miR-501-5p	DBF4	ENSG00000006634	0.907
hsa-miR-340-5p	YES1	ENSG00000176105	0.906
hsa-miR-432-5p	SYN1	ENSG00000008056	0.906
hsa-miR-374a-5p	STEAP4	ENSG00000127954	0.906
hsa-miR-3180-5p	SRGAP1	ENSG00000196935	0.906
hsa-miR-454-3p	NEXMIF	ENSG00000050030	0.906
hsa-miR-93-5p	FEM1C	ENSG00000145780	0.906
hsa-miR-187-5p	CCDC173	ENSG00000154479	0.906
hsa-miR-15b-5p	CACNA1E	ENSG00000198216	0.906
hsa-miR-374a-5p	ARL5B	ENSG00000165997	0.906
hsa-miR-301a-3p	SIK1	ENSG00000142178	0.905
hsa-miR-129-5p	SC5D	ENSG00000109929	0.905
hsa-miR-548ah-3p	ZNF626	ENSG00000188171	0.904
hsa-miR-500a-3p	RHBDF1	ENSG00000007384	0.904
hsa-miR-9-5p	PGAP1	ENSG00000197121	0.904
hsa-miR-122-5p	MIXL1	ENSG00000185155	0.904
hsa-miR-3176	KCNJ2	ENSG00000123700	0.904
hsa-miR-513b-5p	FEM1C	ENSG00000145780	0.904
hsa-miR-144-3p	ZNF804A	ENSG00000170396	0.903
hsa-miR-3074-5p	U2AF1	ENSG00000160201	0.903
hsa-miR-548ah-5p	TMEM170B	ENSG00000205269	0.903
hsa-miR-92a-3p	SMIM11A	ENSG00000205670	0.903
hsa-miR-664b-3p	SCN3B	ENSG00000166257	0.903
hsa-miR-124-3p	LRFN1	ENSG00000128011	0.903
hsa-miR-1303	JAM2	ENSG00000154721	0.903
hsa-miR-449a	EML5	ENSG00000165521	0.903
hsa-miR-548x-5p	NR1D2	ENSG00000174738	0.902
hsa-miR-124-3p	KCNJ2	ENSG00000123700	0.902
hsa-miR-32-5p	ADRB1	ENSG00000043591	0.902
hsa-miR-4775	ZBTB43	ENSG00000169155	0.901
hsa-miR-432-5p	NCR3LG1	ENSG00000188211	0.901
hsa-miR-103a-3p	BORCS6	ENSG00000196544	0.901
hsa-miR-101-3p	ZNF804A	ENSG00000170396	0.9
hsa-miR-103a-2-5p	SOX5	ENSG00000134532	0.9
hsa-miR-147a	SMAD9	ENSG00000120693	0.9
hsa-miR-3150a-3p	SLC1A2	ENSG00000110436	0.9
hsa-miR-9-5p	SIK1	ENSG00000142178	0.9
hsa-miR-1303	SCN3B	ENSG00000166257	0.9
hsa-miR-548a-5p	RLIM	ENSG00000131263	0.9

hsa-miR-548ah-3p	PGAP1	ENSG00000197121	0.9
hsa-miR-574-5p	NCR3LG1	ENSG00000188211	0.9
hsa-miR-500a-5p	KLHL15	ENSG00000174010	0.9
hsa-miR-371b-5p	GCSAML	ENSG00000169224	0.9
hsa-miR-4775	GCSAML	ENSG00000169224	0.9
hsa-miR-548a-5p	BTG3	ENSG00000154640	0.9
hsa-miR-490-3p	SLC26A5	ENSG00000170615	0.899
hsa-miR-374a-5p	PDE4D	ENSG00000113448	0.899
hsa-miR-129-5p	LRRIQ3	ENSG00000162620	0.899
hsa-miR-129-5p	LINGO2	ENSG00000174482	0.899
hsa-miR-625-5p	OTX1	ENSG00000115507	0.898
hsa-miR-340-5p	NR4A3	ENSG00000119508	0.898
hsa-miR-624-5p	CNDP1	ENSG00000150656	0.898
hsa-miR-548ah-3p	RNF152	ENSG00000176641	0.897
hsa-miR-3064-5p	NR4A3	ENSG00000119508	0.897
hsa-miR-144-5p	NCR3LG1	ENSG00000188211	0.897
hsa-miR-32-5p	TMEM255A	ENSG00000125355	0.896
hsa-miR-144-3p	PRLR	ENSG00000113494	0.896
hsa-miR-449a	NR4A2	ENSG00000153234	0.896
hsa-miR-513a-5p	CEP19	ENSG00000174007	0.896
hsa-miR-124-3p	SRGAP1	ENSG00000196935	0.895
hsa-miR-103a-2-5p	RLIM	ENSG00000131263	0.895
hsa-miR-3150a-3p	PRLR	ENSG00000113494	0.895
hsa-miR-20a-5p	PGAP1	ENSG00000197121	0.895
hsa-miR-144-3p	NEXMIF	ENSG00000050030	0.895
hsa-miR-449a	MBLAC1	ENSG00000214309	0.895
hsa-miR-454-3p	KRT23	ENSG00000108244	0.895
hsa-miR-616-5p	GCSAML	ENSG00000169224	0.895
hsa-miR-432-5p	CACNA1E	ENSG00000198216	0.895
hsa-miR-340-5p	ASCL2	ENSG00000183734	0.895
hsa-miR-548ah-3p	ZBTB21	ENSG00000173276	0.894
hsa-miR-548a-5p	TMEM255A	ENSG00000125355	0.894
hsa-miR-548ai	SORCS3	ENSG00000156395	0.894
hsa-miR-502-5p	RGS20	ENSG00000147509	0.894
hsa-miR-548ah-3p	HJURP	ENSG00000123485	0.894
hsa-miR-153-3p	FEM1C	ENSG00000145780	0.894
hsa-miR-374a-5p	DEPDC1	ENSG00000024526	0.894
hsa-miR-449a	CNIH2	ENSG00000174871	0.894
hsa-miR-4659b-5p	CFL2	ENSG00000165410	0.894
hsa-miR-92a-3p	ARHGEF10	ENSG00000104728	0.894
hsa-miR-506-3p	SRGAP1	ENSG00000196935	0.893
hsa-miR-616-5p	SKA3	ENSG00000165480	0.893
hsa-miR-513a-5p	RNF152	ENSG00000176641	0.893
hsa-miR-93-5p	RBM34	ENSG00000188739	0.893
hsa-miR-32-5p	CHST7	ENSG00000147119	0.893
hsa-miR-548a-5p	CAV1	ENSG00000105974	0.893
hsa-miR-101-3p	SMAD9	ENSG00000120693	0.892

hsa-miR-3074-3p	NCR3LG1	ENSG00000188211	0.892
hsa-miR-206	MET	ENSG00000105976	0.892
hsa-miR-144-3p	DUSP1	ENSG00000120129	0.892
hsa-miR-365b-5p	CACNA1E	ENSG00000198216	0.892
hsa-miR-454-3p	STEAP4	ENSG00000127954	0.891
hsa-miR-502-5p	SLC26A5	ENSG00000170615	0.891
hsa-miR-625-5p	SIAH1	ENSG00000196470	0.891
hsa-miR-548a-5p	PPP1R9A	ENSG00000158528	0.891
hsa-miR-628-5p	NAT2	ENSG00000156006	0.891
hsa-miR-340-5p	IL1RL1	ENSG00000115602	0.891
hsa-miR-144-3p	ATP1B2	ENSG00000129244	0.891
hsa-miR-3074-5p	CACNA1E	ENSG00000198216	0.89
hsa-miR-196a-5p	SMAD6	ENSG00000137834	0.889
hsa-miR-3074-5p	RGPD5	ENSG00000015568	0.889
hsa-miR-17-5p	RBM34	ENSG00000188739	0.889
hsa-miR-664b-3p	PPP1R9A	ENSG00000158528	0.889
hsa-miR-371b-5p	KLHL15	ENSG00000174010	0.889
hsa-miR-15a-5p	TICRR	ENSG00000140534	0.888
hsa-miR-4717-5p	SLC23A3	ENSG00000213901	0.888
hsa-miR-365a-3p	RNF152	ENSG00000176641	0.888
hsa-miR-21-5p	PIK3R1	ENSG00000145675	0.888
hsa-miR-193b-3p	EML5	ENSG00000165521	0.888
hsa-miR-539-5p	DBF4	ENSG00000006634	0.888
hsa-miR-15b-5p	TICRR	ENSG00000140534	0.886
hsa-miR-548ah-3p	SMAD6	ENSG00000137834	0.886
hsa-miR-371b-5p	SERTAD2	ENSG00000179833	0.886
hsa-miR-4482-5p	NCR3LG1	ENSG00000188211	0.886
hsa-miR-490-3p	NCR3LG1	ENSG00000188211	0.886
hsa-miR-574-5p	IL1RL1	ENSG00000115602	0.886
hsa-miR-490-5p	ADGRE3	ENSG00000131355	0.886
hsa-miR-15b-5p	YOD1	ENSG00000180667	0.885
hsa-miR-206	KCNJ2	ENSG00000123700	0.885
hsa-miR-1228-3p	IFIT2	ENSG00000119922	0.885
hsa-miR-664b-3p	EREG	ENSG00000124882	0.885
hsa-miR-340-5p	ARHGAP29	ENSG00000137962	0.885
hsa-miR-15a-5p	YOD1	ENSG00000180667	0.884
hsa-miR-616-5p	KLHL15	ENSG00000174010	0.884
hsa-miR-3064-5p	IL1RL1	ENSG00000115602	0.884
hsa-miR-513b-5p	DBF4	ENSG00000006634	0.884
hsa-miR-193a-5p	CHD1	ENSG00000153922	0.884
hsa-miR-513a-5p	ATF3	ENSG00000162772	0.884
hsa-miR-30c-5p	ASB2	ENSG00000100628	0.884
hsa-miR-9-5p	TXNDC5	ENSG00000239264	0.883
hsa-miR-628-5p	RGPD6	ENSG00000183054	0.883
hsa-miR-664b-3p	PIK3R1	ENSG00000145675	0.883
hsa-miR-101-3p	IL13	ENSG00000169194	0.883
hsa-miR-20a-5p	GJA3	ENSG00000121743	0.883

hsa-miR-20a-5p	EREG	ENSG00000124882	0.883
hsa-miR-1234-3p	CDKN1C	ENSG00000129757	0.883
hsa-miR-365a-5p	CACNA1E	ENSG00000198216	0.883
hsa-miR-374a-5p	RASGRF1	ENSG00000058335	0.882
hsa-miR-3605-5p	NIPAL4	ENSG00000172548	0.882
hsa-miR-219a-5p	MCOLN3	ENSG00000055732	0.882
hsa-miR-506-3p	KCNJ2	ENSG00000123700	0.882
hsa-miR-513b-5p	SRGAP1	ENSG00000196935	0.881
hsa-miR-4775	PGAP1	ENSG00000197121	0.881
hsa-miR-1228-3p	P4HA2	ENSG00000072682	0.881
hsa-miR-556-5p	OTOF	ENSG00000115155	0.881
hsa-miR-374a-5p	CCSER1	ENSG00000184305	0.881
hsa-miR-548ah-5p	CAV1	ENSG00000105974	0.881
hsa-miR-122-5p	BATF2	ENSG00000168062	0.881
hsa-miR-513a-5p	RORB	ENSG00000198963	0.88
hsa-miR-500a-5p	PDK4	ENSG00000004799	0.88
hsa-miR-196a-5p	YOD1	ENSG00000180667	0.879
hsa-miR-199b-5p	RASGEF1B	ENSG00000138670	0.879
hsa-miR-758-3p	FPR3	ENSG00000187474	0.879
hsa-miR-708-5p	FNDC7	ENSG00000143107	0.879
hsa-miR-30c-5p	COL9A3	ENSG00000092758	0.879
hsa-miR-548ah-3p	CFL2	ENSG00000165410	0.879
hsa-miR-103a-2-5p	CD83	ENSG00000112149	0.879
hsa-miR-1299	ASB12	ENSG00000198881	0.879
hsa-miR-24-1-5p	ZBTB43	ENSG00000169155	0.878
hsa-miR-664b-3p	LRRIQ3	ENSG00000162620	0.878
hsa-miR-374a-5p	GCSAML	ENSG00000169224	0.878
hsa-miR-17-5p	FEM1C	ENSG00000145780	0.878
hsa-miR-129-5p	ZNF703	ENSG00000183779	0.877
hsa-miR-20a-5p	ZNF484	ENSG00000127081	0.877
hsa-miR-664b-3p	TENM1	ENSG00000009694	0.877
hsa-miR-758-3p	NCR3LG1	ENSG00000188211	0.877
hsa-miR-548ah-3p	GRM3	ENSG00000198822	0.877
hsa-miR-4494	ERMN	ENSG00000136541	0.877
hsa-miR-3150a-3p	TCTE1	ENSG00000146221	0.876
hsa-miR-20a-5p	RBM34	ENSG00000188739	0.876
hsa-miR-129-5p	GIMAP1	ENSG00000213203	0.876
hsa-miR-129-5p	COL1A1	ENSG00000108821	0.876
hsa-miR-340-5p	CCNT1	ENSG00000129315	0.876
hsa-miR-500a-3p	ARHGAP29	ENSG00000137962	0.876
hsa-miR-199a-5p	ZNF547	ENSG00000152433	0.875
hsa-miR-500a-5p	ZBTB10	ENSG00000205189	0.875
hsa-miR-15b-5p	SIAH1	ENSG00000196470	0.875
hsa-miR-513a-5p	SFN	ENSG00000175793	0.875
hsa-miR-548ah-3p	FEM1C	ENSG00000145780	0.875
hsa-miR-129-5p	DBF4	ENSG00000006634	0.875
hsa-miR-539-5p	CCSER1	ENSG00000184305	0.875

hsa-miR-365a-3p	ADAMTS1	ENSG00000154734	0.875
hsa-miR-101-3p	SORCS3	ENSG00000156395	0.874
hsa-miR-548x-5p	SH3D19	ENSG00000109686	0.874
hsa-miR-660-5p	SDC1	ENSG00000115884	0.874
hsa-miR-651-5p	PRLR	ENSG00000113494	0.874
hsa-miR-513b-5p	EML5	ENSG00000165521	0.874
hsa-miR-548ah-3p	EML5	ENSG00000165521	0.874
hsa-miR-500a-5p	CCNT1	ENSG00000129315	0.874
hsa-miR-551b-5p	TLR10	ENSG00000174123	0.873
hsa-miR-548a-5p	STEAP4	ENSG00000127954	0.873
hsa-miR-362-5p	PAQR8	ENSG00000170915	0.873
hsa-miR-4645-5p	NEXMIF	ENSG00000050030	0.873
hsa-miR-3074-3p	LRRIQ3	ENSG00000162620	0.873
hsa-miR-3074-5p	CHST7	ENSG00000147119	0.873
hsa-miR-513a-5p	CCNT1	ENSG00000129315	0.873
hsa-miR-664a-3p	STBD1	ENSG00000118804	0.872
hsa-miR-193a-3p	SKA3	ENSG00000165480	0.872
hsa-miR-15a-5p	NAT8L	ENSG00000185818	0.872
hsa-miR-371b-5p	CFL2	ENSG00000165410	0.872
hsa-miR-199b-5p	ZNF547	ENSG00000152433	0.871
hsa-miR-625-5p	KRT23	ENSG00000108244	0.871
hsa-miR-206	ANKRD18A	ENSG00000180071	0.871
hsa-miR-548x-5p	YOD1	ENSG00000180667	0.87
hsa-miR-153-3p	VIT	ENSG00000205221	0.87
hsa-miR-454-3p	PGAP1	ENSG00000197121	0.87
hsa-miR-708-5p	NR4A3	ENSG00000119508	0.87
hsa-miR-4494	NCR3LG1	ENSG00000188211	0.87
hsa-miR-3064-5p	IL13	ENSG00000169194	0.87
hsa-miR-664a-3p	ARHGAP29	ENSG00000137962	0.87
hsa-miR-20a-5p	YES1	ENSG00000176105	0.869
hsa-miR-664b-3p	SLC1A2	ENSG00000110436	0.869
hsa-miR-3150a-3p	SIGLEC1	ENSG00000088827	0.869
hsa-miR-374a-5p	NR4A3	ENSG00000119508	0.869
hsa-miR-502-5p	NEXMIF	ENSG00000050030	0.869
hsa-miR-374a-5p	FEM1C	ENSG00000145780	0.869
hsa-miR-371b-5p	ERMN	ENSG00000136541	0.869
hsa-miR-548a-5p	EREG	ENSG00000124882	0.869
hsa-miR-144-3p	ARL5B	ENSG00000165997	0.869
hsa-miR-548x-5p	YES1	ENSG00000176105	0.868
hsa-miR-365a-3p	SERTAD2	ENSG00000179833	0.868
hsa-miR-590-5p	LRRIQ3	ENSG00000162620	0.868
hsa-miR-877-5p	GLDC	ENSG00000178445	0.868
hsa-miR-4645-5p	PER1	ENSG00000179094	0.867
hsa-miR-885-5p	PAQR8	ENSG00000170915	0.867
hsa-miR-98-5p	OTOF	ENSG00000115155	0.867
hsa-miR-129-5p	EML5	ENSG00000165521	0.867
hsa-miR-506-3p	CHD1	ENSG00000153922	0.867

hsa-miR-32-5p	AURKA	ENSG00000087586	0.867
hsa-miR-30c-5p	ZNF805	ENSG00000204524	0.866
hsa-miR-548ah-3p	YOD1	ENSG00000180667	0.866
hsa-miR-624-3p	P4HA2	ENSG00000072682	0.866
hsa-miR-15b-5p	NAT8L	ENSG00000185818	0.866
hsa-miR-129-5p	LRRN3	ENSG00000173114	0.866
hsa-miR-548ah-5p	KLHL15	ENSG00000174010	0.866
hsa-miR-371b-5p	FBLN5	ENSG00000140092	0.866
hsa-miR-17-3p	TKTL2	ENSG00000151005	0.865
hsa-miR-885-5p	REL	ENSG00000162924	0.865
hsa-miR-199b-5p	PPP1R9A	ENSG00000158528	0.865
hsa-miR-125b-5p	ICOSLG	ENSG00000160223	0.865
hsa-miR-98-5p	B3GNT7	ENSG00000156966	0.865
hsa-miR-551b-5p	ZBTB10	ENSG00000205189	0.864
hsa-miR-664b-3p	PDE4D	ENSG00000113448	0.864
hsa-miR-103a-2-5p	NCR3LG1	ENSG00000188211	0.864
hsa-miR-548ah-5p	MYO3B	ENSG00000071909	0.864
hsa-miR-4659b-5p	LRG1	ENSG00000171236	0.864
hsa-miR-548ai	JAM2	ENSG00000154721	0.864
hsa-miR-548a-5p	C4orf19	ENSG00000154274	0.864
hsa-miR-144-3p	ZNF805	ENSG00000204524	0.863
hsa-miR-340-3p	ZNF805	ENSG00000204524	0.863
hsa-miR-449a	ZNF285	ENSG00000267508	0.863
hsa-miR-664a-3p	ZBTB10	ENSG00000205189	0.863
hsa-miR-101-3p	PGAP1	ENSG00000197121	0.863
hsa-miR-124-3p	CHD1	ENSG00000153922	0.863
hsa-miR-548a-5p	TMEM170B	ENSG00000205269	0.862
hsa-miR-129-5p	SERTAD2	ENSG00000179833	0.862
hsa-miR-3074-5p	RASGRF1	ENSG00000058335	0.862
hsa-miR-501-5p	NHLRC1	ENSG00000187566	0.862
hsa-miR-92a-3p	IFIT2	ENSG00000119922	0.862
hsa-miR-9-5p	HES1	ENSG00000114315	0.862
hsa-miR-1299	GRM3	ENSG00000198822	0.862
hsa-miR-548ah-5p	TNFAIP3	ENSG00000118503	0.861
hsa-miR-548ah-3p	STEAP4	ENSG00000127954	0.861
hsa-miR-1228-3p	PRR7	ENSG00000131188	0.861
hsa-miR-664a-3p	P4HA2	ENSG00000072682	0.861
hsa-miR-548ah-3p	NR4A3	ENSG00000119508	0.861
hsa-miR-103a-2-5p	NDUFV2	ENSG00000178127	0.861
hsa-miR-628-5p	NCR3LG1	ENSG00000188211	0.861
hsa-miR-513a-5p	KIAA1324	ENSG00000116299	0.861
hsa-miR-144-3p	GLDC	ENSG00000178445	0.861
hsa-miR-501-5p	CCNT1	ENSG00000129315	0.861
hsa-miR-340-5p	SMAD9	ENSG00000120693	0.86
hsa-miR-199b-5p	RORB	ENSG00000198963	0.86
hsa-miR-664b-3p	RGPD6	ENSG00000183054	0.86
hsa-miR-449a	RFX8	ENSG00000196460	0.86

hsa-miR-9-5p	P4HA2	ENSG00000072682	0.86
hsa-miR-103a-3p	NAT8L	ENSG00000185818	0.86
hsa-miR-513a-5p	GFPT2	ENSG00000131459	0.86
hsa-miR-513a-5p	EFNA3	ENSG00000143590	0.86
hsa-miR-9-5p	CCSER1	ENSG00000184305	0.86
hsa-miR-3176	CCDC74A	ENSG00000163040	0.86
hsa-miR-374a-5p	NR4A2	ENSG00000153234	0.859
hsa-miR-3945	NCR3LG1	ENSG00000188211	0.859
hsa-miR-1299	GJB7	ENSG00000164411	0.859
hsa-miR-20a-5p	FZD7	ENSG00000155760	0.859
hsa-miR-548ah-3p	FZD7	ENSG00000155760	0.859
hsa-miR-513b-5p	DHRS9	ENSG00000073737	0.859
hsa-miR-129-5p	CCR6	ENSG00000112486	0.859
hsa-miR-17-3p	ZNF331	ENSG00000130844	0.858
hsa-miR-187-5p	TIPARP	ENSG00000163659	0.858
hsa-miR-885-5p	TENM1	ENSG00000009694	0.858
hsa-miR-551b-5p	SKA3	ENSG00000165480	0.858
hsa-miR-3613-5p	PGAP1	ENSG00000197121	0.858
hsa-miR-19a-5p	KCNJ2	ENSG00000123700	0.858
hsa-miR-3180-5p	CEP19	ENSG00000174007	0.858
hsa-miR-190b	CCSER1	ENSG00000184305	0.858
hsa-miR-193a-3p	SIAH1	ENSG00000196470	0.857
hsa-miR-196a-5p	RPP25L	ENSG00000164967	0.857
hsa-miR-374a-5p	JAM2	ENSG00000154721	0.857
hsa-miR-144-3p	HEY2	ENSG00000135547	0.857
hsa-miR-590-5p	DUSP8	ENSG00000184545	0.857
hsa-miR-548ah-3p	TNFSF11	ENSG00000120659	0.856
hsa-miR-454-3p	TMEM170B	ENSG00000205269	0.856
hsa-miR-129-5p	SOX5	ENSG00000134532	0.856
hsa-miR-199a-5p	PPP1R9A	ENSG00000158528	0.856
hsa-miR-708-5p	PDK4	ENSG00000004799	0.856
hsa-miR-187-5p	KRT23	ENSG00000108244	0.856
hsa-miR-98-5p	COL1A1	ENSG00000108821	0.856
hsa-miR-3074-3p	CCNT1	ENSG00000129315	0.856
hsa-miR-188-5p	SEMA3G	ENSG00000010319	0.855
hsa-miR-340-5p	SDE2	ENSG00000143751	0.855
hsa-miR-24-3p	RLIM	ENSG00000131263	0.855
hsa-miR-628-5p	RGPD5	ENSG00000015568	0.855
hsa-miR-877-5p	OTOF	ENSG00000115155	0.855
hsa-miR-125b-5p	NIPAL4	ENSG00000172548	0.855
hsa-miR-548ah-3p	DUSP1	ENSG00000120129	0.855
hsa-miR-3176	TENM1	ENSG00000009694	0.854
hsa-miR-548x-5p	SIAH1	ENSG00000196470	0.854
hsa-miR-3064-5p	ICOSLG	ENSG00000160223	0.854
hsa-miR-551b-5p	CCSER1	ENSG00000184305	0.854
hsa-miR-101-3p	ARHGEF10	ENSG00000104728	0.854
hsa-miR-4775	ZBTB10	ENSG00000205189	0.853

hsa-miR-3150a-3p	TNFRSF10C	ENSG00000173535	0.853
hsa-miR-101-3p	HEY2	ENSG00000135547	0.853
hsa-miR-147a	GIMAP1	ENSG00000213203	0.853
hsa-miR-15a-5p	TMEM255A	ENSG00000125355	0.852
hsa-miR-1303	SOX5	ENSG00000134532	0.852
hsa-miR-877-5p	NCR3LG1	ENSG00000188211	0.852
hsa-miR-548ah-3p	CXCL8	ENSG00000169429	0.852
hsa-miR-628-3p	ZBTB10	ENSG00000205189	0.851
hsa-miR-98-5p	DUSP1	ENSG00000120129	0.851
hsa-miR-501-5p	ZNF805	ENSG00000204524	0.85
hsa-miR-129-5p	ZBTB43	ENSG00000169155	0.85
hsa-miR-17-5p	YES1	ENSG00000176105	0.85
hsa-miR-193b-3p	SIAH1	ENSG00000196470	0.85
hsa-miR-548s	PRICKLE2	ENSG00000163637	0.85
hsa-miR-3150a-3p	PRG2	ENSG00000186652	0.85
hsa-miR-624-5p	MET	ENSG00000105976	0.85
hsa-miR-3613-5p	FLRT1	ENSG00000126500	0.85
hsa-miR-1228-3p	TCTE1	ENSG00000146221	0.849
hsa-miR-4645-5p	SOX5	ENSG00000134532	0.849
hsa-miR-92a-3p	FASLG	ENSG00000117560	0.849
hsa-miR-144-5p	CCNT1	ENSG00000129315	0.849
hsa-miR-3144-5p	B3GNT7	ENSG00000156966	0.849
hsa-miR-501-5p	TMEM170B	ENSG00000205269	0.848
hsa-miR-4775	SH3D19	ENSG00000109686	0.848
hsa-miR-4775	PDK4	ENSG00000004799	0.848
hsa-miR-548ah-3p	LRRN3	ENSG00000173114	0.848
hsa-miR-199b-5p	GPR68	ENSG00000119714	0.848
hsa-miR-374a-5p	GJA3	ENSG00000121743	0.848
hsa-miR-371b-5p	GCSAM	ENSG00000174500	0.848
hsa-miR-17-3p	DBF4	ENSG00000006634	0.848
hsa-miR-548s	BORCS6	ENSG00000196544	0.848
hsa-miR-129-5p	TIPARP	ENSG00000163659	0.847
hsa-miR-4433a-3p	SYNM	ENSG00000182253	0.847
hsa-miR-513a-5p	NEXMIF	ENSG00000050030	0.847
hsa-miR-4775	TNFAIP3	ENSG00000118503	0.846
hsa-miR-548ah-3p	SKOR1	ENSG00000188779	0.846
hsa-miR-371b-5p	SKA3	ENSG00000165480	0.846
hsa-miR-539-5p	SCN3B	ENSG00000166257	0.846
hsa-miR-101-3p	RNF152	ENSG00000176641	0.846
hsa-miR-101-3p	REL	ENSG00000162924	0.846
hsa-miR-432-5p	ARL5B	ENSG00000165997	0.846
hsa-miR-574-5p	TNFSF9	ENSG00000125657	0.845
hsa-miR-548x-5p	TMEM170B	ENSG00000205269	0.845
hsa-miR-9-5p	NR4A3	ENSG00000119508	0.845
hsa-miR-371b-5p	NR1D2	ENSG00000174738	0.845
hsa-miR-190a-5p	CCSER1	ENSG00000184305	0.845
hsa-miR-513a-5p	B3GNT7	ENSG00000156966	0.845

hsa-miR-101-3p	TMEM170B	ENSG00000205269	0.844
hsa-miR-4775	RLIM	ENSG00000131263	0.844
hsa-miR-548x-5p	PFKFB3	ENSG00000170525	0.844
hsa-miR-501-5p	NCR3LG1	ENSG00000188211	0.844
hsa-miR-301a-3p	KRT23	ENSG00000108244	0.844
hsa-miR-449a	IL1RL1	ENSG00000115602	0.844
hsa-miR-301a-3p	CFL2	ENSG00000165410	0.844
hsa-miR-4775	C14orf119	ENSG00000179933	0.844
hsa-miR-1256	KLHL15	ENSG00000174010	1
hsa-miR-1291	CHRNB2	ENSG00000160716	1
hsa-miR-1291	PFKFB3	ENSG00000170525	1
hsa-miR-1537-5p	OTX1	ENSG00000115507	1
hsa-miR-1908-5p	RTN4R	ENSG00000040608	1
hsa-miR-1908-5p	APOE	ENSG00000130203	1
hsa-miR-1908-5p	JUND	ENSG00000130522	1
hsa-miR-1908-5p	SLC1A2	ENSG00000110436	1
hsa-miR-3065-5p	STEAP4	ENSG00000127954	1
hsa-miR-3143	ZNF703	ENSG00000183779	1
hsa-miR-3143	NRARP	ENSG00000198435	1
hsa-miR-3143	PDF	ENSG00000258429	1
hsa-miR-3143	KLHL15	ENSG00000174010	1
hsa-miR-3179	KLHL15	ENSG00000174010	1
hsa-miR-3611	CCNT1	ENSG00000129315	1
hsa-miR-3611	SLC7A5	ENSG00000103257	1
hsa-miR-3611	PDE4D	ENSG00000113448	1
hsa-miR-3611	NR1D2	ENSG00000174738	1
hsa-miR-3611	REL	ENSG00000162924	1
hsa-miR-410-3p	ARHGAP29	ENSG00000137962	1
hsa-miR-410-3p	CKAP2L	ENSG00000169607	1
hsa-miR-410-3p	DUSP8	ENSG00000184545	1
hsa-miR-410-3p	BTG3	ENSG00000154640	1
hsa-miR-410-3p	MET	ENSG00000105976	1
hsa-miR-410-3p	SC5D	ENSG00000109929	1
hsa-miR-410-3p	TNFSF9	ENSG00000125657	1
hsa-miR-410-3p	KLHL15	ENSG00000174010	1
hsa-miR-4424	HSPA1A	ENSG00000204389	1
hsa-miR-4424	GOLGA8M	ENSG00000188626	1
hsa-miR-4425	PDF	ENSG00000258429	1
hsa-miR-4425	ARL5B	ENSG00000165997	1
hsa-miR-4677-5p	CEBPB	ENSG00000172216	1
hsa-miR-4677-5p	ICOSLG	ENSG00000160223	1
hsa-miR-4709-5p	REL	ENSG00000162924	1
hsa-miR-4709-5p	CEBPD	ENSG00000221869	1
hsa-miR-4724-5p	KLHL15	ENSG00000174010	1
hsa-miR-4724-5p	PRICKLE2	ENSG00000163637	1
hsa-miR-4772-5p	KLHL15	ENSG00000174010	1
hsa-miR-4772-5p	ZBTB21	ENSG00000173276	1

hsa-miR-4772-5p	YOD1	ENSG00000180667	1
hsa-miR-4781-5p	FAM43A	ENSG00000185112	1
hsa-miR-4781-5p	ICOSLG	ENSG00000160223	1
hsa-miR-4781-5p	HIC1	ENSG00000177374	1
hsa-miR-4802-5p	SRGAP1	ENSG00000196935	1
hsa-miR-4802-5p	CD180	ENSG00000134061	1
hsa-miR-4802-5p	CCNT1	ENSG00000129315	1
hsa-miR-4999-5p	OTX1	ENSG00000115507	1
hsa-miR-511-5p	SC5D	ENSG00000109929	1
hsa-miR-511-5p	TNFAIP8L2	ENSG00000163154	1
hsa-miR-511-5p	REL	ENSG00000162924	1
hsa-miR-544a	ZNF483	ENSG00000173258	1
hsa-miR-544a	CCSER1	ENSG00000184305	1
hsa-miR-544a	RLIM	ENSG00000131263	1
hsa-miR-544a	ARL5B	ENSG00000165997	1
hsa-miR-544b	HSPA6	ENSG00000173110	1
hsa-miR-552-3p	DDX47	ENSG00000213782	1
hsa-miR-552-5p	BTG3	ENSG00000154640	1
hsa-miR-552-5p	PER1	ENSG00000179094	1
hsa-miR-552-5p	MASTL	ENSG00000120539	1
hsa-miR-552-5p	TIPARP	ENSG00000163659	1
hsa-miR-5582-5p	DGAT2	ENSG00000062282	1
hsa-miR-5582-5p	SDC1	ENSG00000115884	1
hsa-miR-5582-5p	SDE2	ENSG00000143751	1
hsa-miR-5680	TNFSF9	ENSG00000125657	1
hsa-miR-5680	ZNF696	ENSG00000185730	1
hsa-miR-5680	BUB1	ENSG00000169679	1
hsa-miR-5680	TENM1	ENSG00000009694	1
hsa-miR-5680	KLHL15	ENSG00000174010	1
hsa-miR-5680	ARL5B	ENSG00000165997	1
hsa-miR-5680	C15orf48	ENSG00000166920	1
hsa-miR-5680	FEM1C	ENSG00000145780	1
hsa-miR-5680	PRLR	ENSG00000113494	1
hsa-miR-580-3p	SPRED3	ENSG00000188766	1
hsa-miR-580-5p	ZBTB43	ENSG00000169155	1
hsa-miR-580-5p	TNFAIP3	ENSG00000118503	1
hsa-miR-619-3p	ICOSLG	ENSG00000160223	1
hsa-miR-619-5p	ZNF626	ENSG00000188171	1
hsa-miR-619-5p	PER1	ENSG00000179094	1
hsa-miR-619-5p	IFIT3	ENSG00000119917	1
hsa-miR-619-5p	SLC7A5	ENSG00000103257	1
hsa-miR-619-5p	FOS	ENSG00000170345	1
hsa-miR-619-5p	COMTD1	ENSG00000165644	1
hsa-miR-619-5p	RNF152	ENSG00000176641	1
hsa-miR-641	SDE2	ENSG00000143751	1
hsa-miR-6733-5p	ENC1	ENSG00000171617	1
hsa-miR-6733-5p	CENPA	ENSG00000115163	1

hsa-miR-6734-5p	AURKA	ENSG00000087586	1
hsa-miR-6734-5p	SLC7A5	ENSG00000103257	1
hsa-miR-6770-5p	KLHL15	ENSG00000174010	1
hsa-miR-6770-5p	FBLN5	ENSG00000140092	1
hsa-miR-6813-5p	CCDC121	ENSG00000176714	1
hsa-miR-6837-3p	NR1D2	ENSG00000174738	1
hsa-miR-6837-3p	TNFAIP3	ENSG00000118503	1
hsa-miR-6837-5p	CCDC85C	ENSG00000205476	1
hsa-miR-7110-5p	RTN4R	ENSG00000040608	1
hsa-miR-7854-3p	CFL2	ENSG00000165410	1
hsa-miR-7854-3p	FBLN5	ENSG00000140092	1
hsa-miR-876-5p	PER1	ENSG00000179094	1
hsa-miR-889-3p	ZBED2	ENSG00000177494	1
hsa-miR-889-3p	ZNF703	ENSG00000183779	1
hsa-miR-889-3p	HIF1A	ENSG00000100644	1
hsa-miR-935	HIF1A	ENSG00000100644	1
hsa-miR-942-5p	ESM1	ENSG00000164283	1
hsa-miR-942-5p	KLF2	ENSG00000127528	1
hsa-miR-942-5p	ANKRD18A	ENSG00000180071	1
hsa-miR-410-3p	NETO1	ENSG00000166342	0.793
hsa-miR-410-3p	NEXMIF	ENSG00000050030	0.98
hsa-miR-410-3p	DEPDC1	ENSG00000024526	0.99
hsa-miR-410-3p	RORB	ENSG00000198963	0.97
hsa-miR-410-3p	FASLG	ENSG00000117560	0.99
hsa-miR-410-3p	TMEM170B	ENSG00000205269	0.99
hsa-miR-410-3p	ATP1B2	ENSG00000129244	0.995
hsa-miR-410-3p	NPTX2	ENSG00000106236	0.993
hsa-miR-410-3p	PPP1R9A	ENSG00000158528	0.994
hsa-miR-410-3p	SMAD6	ENSG00000137834	0.896
hsa-miR-410-3p	SLC36A3	ENSG00000186334	0.859
hsa-miR-410-3p	CFL2	ENSG00000165410	0.98
hsa-miR-410-3p	BTG3	ENSG00000154640	0.99
hsa-miR-410-3p	CCNT1	ENSG00000129315	0.991
hsa-miR-410-3p	KLHL15	ENSG00000174010	0.998
hsa-miR-410-3p	ESM1	ENSG00000164283	0.99
hsa-miR-410-3p	ARHGAP39	ENSG00000147799	0.977
hsa-miR-876-5p	CEP19	ENSG00000174007	0.817
hsa-miR-876-5p	PER1	ENSG00000179094	0.992
hsa-miR-876-5p	SRGAP1	ENSG00000196935	0.992
hsa-miR-876-5p	PDF	ENSG00000258429	1
hsa-miR-876-5p	RORB	ENSG00000198963	0.907
hsa-miR-876-5p	TCTE1	ENSG00000146221	0.99
hsa-miR-3143	ZBTB43	ENSG00000169155	1
hsa-miR-4677-5p	YOD1	ENSG00000180667	0.998
hsa-miR-511-5p	NCR3LG1	ENSG00000188211	0.997
hsa-miR-641	KCNJ2	ENSG00000123700	0.996
hsa-miR-544b	NCR3LG1	ENSG00000188211	0.995

hsa-miR-5680	FEM1C	ENSG00000145780	0.995
hsa-miR-5582-5p	LINGO2	ENSG00000174482	0.994
hsa-miR-889-3p	EREG	ENSG00000124882	0.993
hsa-miR-4425	CKAP2L	ENSG00000169607	0.993
hsa-miR-4425	CHIT1	ENSG00000133063	0.991
hsa-miR-544a	DBF4	ENSG00000006634	0.99
hsa-miR-641	RNF152	ENSG00000176641	0.987
hsa-miR-876-5p	CHD1	ENSG00000153922	0.987
hsa-miR-4677-5p	ZMAT4	ENSG00000165061	0.986
hsa-miR-5582-5p	NR4A2	ENSG00000153234	0.984
hsa-miR-4425	SLC36A3	ENSG00000186334	0.983
hsa-miR-3179	CCDC85C	ENSG00000205476	0.983
hsa-miR-3065-5p	ARHGAP29	ENSG00000137962	0.983
hsa-miR-410-3p	SMAD6	ENSG00000137834	0.982
hsa-miR-580-3p	YES1	ENSG00000176105	0.981
hsa-miR-3143	KLHL15	ENSG00000174010	0.981
hsa-miR-580-3p	PGAP1	ENSG00000197121	0.98
hsa-miR-511-5p	LRRIQ3	ENSG00000162620	0.98
hsa-miR-942-5p	CALHM1	ENSG00000185933	0.98
hsa-miR-4724-5p	ARHGAP29	ENSG00000137962	0.98
hsa-miR-3611	PKD2L2	ENSG00000078795	0.979
hsa-miR-641	NCR3LG1	ENSG00000188211	0.979
hsa-miR-410-3p	TMEM170B	ENSG00000205269	0.978
hsa-miR-597-5p	GJB7	ENSG00000164411	0.978
hsa-miR-410-3p	TNFSF11	ENSG00000120659	0.976
hsa-miR-597-5p	PRLR	ENSG00000113494	0.976
hsa-miR-3179	DBF4	ENSG00000006634	0.973
hsa-miR-4677-5p	ADAMTS1	ENSG00000154734	0.973
hsa-miR-3065-5p	NEXMIF	ENSG00000050030	0.968
hsa-miR-889-3p	NETO1	ENSG00000166342	0.968
hsa-miR-3065-5p	SOX5	ENSG00000134532	0.967
hsa-miR-4772-5p	RBM34	ENSG00000188739	0.966
hsa-miR-4709-5p	RANBP17	ENSG00000204764	0.966
hsa-miR-4709-5p	FEM1C	ENSG00000145780	0.966
hsa-miR-4474-5p	EREG	ENSG00000124882	0.966
hsa-miR-942-5p	DBF4	ENSG00000006634	0.965
hsa-miR-3179	RHBDF1	ENSG00000007384	0.962
hsa-miR-1256	PRLR	ENSG00000113494	0.962
hsa-miR-3065-5p	NR4A2	ENSG00000153234	0.962
hsa-miR-889-3p	SDE2	ENSG00000143751	0.961
hsa-miR-1291	NPTX2	ENSG00000106236	0.961
hsa-miR-3143	TNFSF11	ENSG00000120659	0.96
hsa-miR-4724-5p	KLHL15	ENSG00000174010	0.96
hsa-miR-942-5p	CD83	ENSG00000112149	0.96
hsa-miR-3179	EFNA3	ENSG00000143590	0.959
hsa-miR-410-3p	SOX5	ENSG00000134532	0.958
hsa-miR-5680	NR1D2	ENSG00000174738	0.958

hsa-miR-889-3p	GRM3	ENSG00000198822	0.958
hsa-miR-889-3p	LRRIQ3	ENSG00000162620	0.957
hsa-miR-641	SDE2	ENSG00000143751	0.956
hsa-miR-889-3p	PGAP1	ENSG00000197121	0.956
hsa-miR-4677-5p	GCSAM	ENSG00000174500	0.956
hsa-miR-1256	CFL2	ENSG00000165410	0.956
hsa-miR-4474-5p	RLIM	ENSG00000131263	0.955
hsa-miR-410-3p	SERTAD2	ENSG00000179833	0.954
hsa-miR-4999-5p	OTX1	ENSG00000115507	0.953
hsa-miR-5680	HEY2	ENSG00000135547	0.952
hsa-miR-5680	TMEM255A	ENSG00000125355	0.951
hsa-miR-942-5p	EML5	ENSG00000165521	0.95
hsa-miR-4677-5p	PPP1R9A	ENSG00000158528	0.949
hsa-miR-410-3p	PDE4D	ENSG00000113448	0.945
hsa-miR-3065-5p	FZD7	ENSG00000155760	0.945
hsa-miR-410-3p	ZBTB10	ENSG00000205189	0.944
hsa-miR-4677-5p	NR4A3	ENSG00000119508	0.944
hsa-miR-942-5p	BOLA1	ENSG00000178096	0.943
hsa-miR-410-3p	NR4A3	ENSG00000119508	0.942
hsa-miR-889-3p	CXCL8	ENSG00000169429	0.941
hsa-miR-5680	ZMAT4	ENSG00000165061	0.94
hsa-miR-4999-5p	SMAD9	ENSG00000120693	0.94
hsa-miR-544b	FOLR3	ENSG00000110203	0.94
hsa-miR-3065-5p	SORCS3	ENSG00000156395	0.938
hsa-miR-410-3p	LRRIQ3	ENSG00000162620	0.938
hsa-miR-942-5p	PRLR	ENSG00000113494	0.937
hsa-miR-3065-5p	PGAP1	ENSG00000197121	0.936
hsa-miR-4709-5p	NR4A2	ENSG00000153234	0.936
hsa-miR-935	SRGAP1	ENSG00000196935	0.935
hsa-miR-410-3p	SLC36A3	ENSG00000186334	0.934
hsa-miR-3065-5p	RGS20	ENSG00000147509	0.934
hsa-miR-3611	ENC1	ENSG00000171617	0.934
hsa-miR-4425	AHRR	ENSG00000063438	0.933
hsa-miR-511-5p	SORCS3	ENSG00000156395	0.931
hsa-miR-942-5p	CACNA1E	ENSG00000198216	0.931
hsa-miR-3065-5p	PDE4D	ENSG00000113448	0.93
hsa-miR-511-5p	NR4A2	ENSG00000153234	0.93
hsa-miR-876-5p	MCOLN3	ENSG00000055732	0.93
hsa-miR-5680	DUSP1	ENSG00000120129	0.93
hsa-miR-5680	ZNF805	ENSG00000204524	0.928
hsa-miR-942-5p	RANBP17	ENSG00000204764	0.928
hsa-miR-410-3p	GALNT5	ENSG00000136542	0.928
hsa-miR-3065-5p	TMEM170B	ENSG00000205269	0.927
hsa-miR-889-3p	SMAD6	ENSG00000137834	0.927
hsa-miR-619-3p	RASGEF1B	ENSG00000138670	0.927
hsa-miR-3065-5p	KCNJ2	ENSG00000123700	0.927
hsa-miR-3065-5p	ZNF14	ENSG00000105708	0.925

hsa-miR-4999-5p	STBD1	ENSG00000118804	0.925
hsa-miR-876-5p	PRLR	ENSG00000113494	0.925
hsa-miR-942-5p	ZBTB43	ENSG00000169155	0.924
hsa-miR-3065-5p	SPP1	ENSG00000118785	0.924
hsa-miR-619-3p	RANBP17	ENSG00000204764	0.924
hsa-miR-4709-5p	ZNF805	ENSG00000204524	0.923
hsa-miR-5680	SLC1A2	ENSG00000110436	0.923
hsa-miR-5695	REL	ENSG00000162924	0.923
hsa-miR-4491	PRLR	ENSG00000113494	0.923
hsa-miR-580-3p	NR4A2	ENSG00000153234	0.923
hsa-miR-410-3p	CCDC121	ENSG00000176714	0.922
hsa-miR-4474-5p	TXNDC5	ENSG00000239264	0.921
hsa-miR-410-3p	RORB	ENSG00000198963	0.92
hsa-miR-511-5p	LINGO2	ENSG00000174482	0.918
hsa-miR-3065-5p	IER5L	ENSG00000188483	0.918
hsa-miR-410-3p	BTG3	ENSG00000154640	0.918
hsa-miR-3065-5p	RBM44	ENSG00000177483	0.917
hsa-miR-889-3p	RLIM	ENSG00000131263	0.916
hsa-miR-3065-5p	CFL2	ENSG00000165410	0.916
hsa-miR-4424	NCR3LG1	ENSG00000188211	0.915
hsa-miR-580-3p	SLC12A1	ENSG00000074803	0.914
hsa-miR-4724-5p	RANBP17	ENSG00000204764	0.914
hsa-miR-4677-5p	ZBTB10	ENSG00000205189	0.913
hsa-miR-942-5p	TEKT2	ENSG00000092850	0.913
hsa-miR-876-5p	PGAP1	ENSG00000197121	0.912
hsa-miR-889-3p	DBF4	ENSG00000006634	0.912
hsa-miR-641	ZNF805	ENSG00000204524	0.911
hsa-miR-942-5p	PPP1R1A	ENSG00000135447	0.911
hsa-miR-5582-5p	TCTE1	ENSG00000146221	0.91
hsa-miR-580-3p	SOX5	ENSG00000134532	0.91
hsa-miR-544a	PIK3R1	ENSG00000145675	0.91
hsa-miR-511-5p	SIK1	ENSG00000142178	0.909
hsa-miR-3143	DGAT2	ENSG00000062282	0.908
hsa-miR-410-3p	ARL5B	ENSG00000165997	0.903
hsa-miR-3611	ZNF781	ENSG00000196381	0.902
hsa-miR-511-5p	SLC1A2	ENSG00000110436	0.902
hsa-miR-3684	ZNF484	ENSG00000127081	0.9
hsa-miR-580-3p	TMEM170B	ENSG00000205269	0.9
hsa-miR-4677-5p	RGPD6	ENSG00000183054	0.9
hsa-miR-544a	NCR3LG1	ENSG00000188211	0.9
hsa-miR-889-3p	TPO	ENSG00000115705	0.899
hsa-miR-3065-5p	ESM1	ENSG00000164283	0.899
hsa-miR-889-3p	NR4A3	ENSG00000119508	0.897
hsa-miR-5680	LRRIQ3	ENSG00000162620	0.897
hsa-miR-3143	CCNT1	ENSG00000129315	0.897
hsa-miR-3143	OSM	ENSG00000099985	0.895
hsa-miR-410-3p	KCNJ2	ENSG00000123700	0.895

hsa-miR-641	SORCS3	ENSG00000156395	0.893
hsa-miR-4677-5p	SLC1A2	ENSG00000110436	0.893
hsa-miR-410-3p	NETO1	ENSG00000166342	0.893
hsa-miR-4677-5p	PRLR	ENSG00000113494	0.891
hsa-miR-876-5p	NCR3LG1	ENSG00000188211	0.891
hsa-miR-410-3p	EREG	ENSG00000124882	0.891
hsa-miR-4724-5p	SKOR1	ENSG00000188779	0.89
hsa-miR-889-3p	ZBTB10	ENSG00000205189	0.889
hsa-miR-641	NR4A3	ENSG00000119508	0.888
hsa-miR-3143	SLC1A2	ENSG00000110436	0.887
hsa-miR-580-3p	PAQR8	ENSG00000170915	0.887
hsa-miR-641	KLHL15	ENSG00000174010	0.887
hsa-miR-641	ZBTB43	ENSG00000169155	0.886
hsa-miR-5695	P4HA2	ENSG00000072682	0.886
hsa-miR-3143	ARL5B	ENSG00000165997	0.886
hsa-miR-4999-5p	CAV1	ENSG00000105974	0.885
hsa-miR-4724-5p	YES1	ENSG00000176105	0.884
hsa-miR-5582-5p	ZBED2	ENSG00000177494	0.883
hsa-miR-5582-5p	SERTAD2	ENSG00000179833	0.883
hsa-miR-3065-5p	PIK3R1	ENSG00000145675	0.883
hsa-miR-511-5p	GFPT2	ENSG00000131459	0.883
hsa-miR-5680	TMEM170B	ENSG00000205269	0.882
hsa-miR-5695	ANKRD18A	ENSG00000180071	0.882
hsa-miR-935	SLC1A2	ENSG00000110436	0.881
hsa-miR-544a	RLIM	ENSG00000131263	0.88
hsa-miR-3143	ARHGEF10	ENSG00000104728	0.88
hsa-miR-4474-5p	TMEM255A	ENSG00000125355	0.879
hsa-miR-4999-5p	SMAD6	ENSG00000137834	0.878
hsa-miR-4999-5p	ZBTB10	ENSG00000205189	0.877
hsa-miR-889-3p	TMEM255A	ENSG00000125355	0.877
hsa-miR-5680	TENM1	ENSG00000009694	0.877
hsa-miR-4677-5p	RGPD5	ENSG00000015568	0.877
hsa-miR-4724-5p	PGAP1	ENSG00000197121	0.876
hsa-miR-552-3p	C9orf131	ENSG00000174038	0.876
hsa-miR-3143	ZNF696	ENSG00000185730	0.874
hsa-miR-511-5p	TXNDC5	ENSG00000239264	0.873
hsa-miR-3065-5p	CX3CR1	ENSG00000168329	0.873
hsa-miR-3065-5p	ZBTB10	ENSG00000205189	0.872
hsa-miR-4677-5p	SOX5	ENSG00000134532	0.872
hsa-miR-5680	SOX5	ENSG00000134532	0.871
hsa-miR-5680	RGPD5	ENSG00000015568	0.871
hsa-miR-4677-5p	NCR3LG1	ENSG00000188211	0.871
hsa-miR-1291	MYBL2	ENSG00000101057	0.87
hsa-miR-4677-5p	KRT23	ENSG00000108244	0.87
hsa-miR-935	GPR68	ENSG00000119714	0.869
hsa-miR-544a	SOCS1	ENSG00000185338	0.868
hsa-miR-889-3p	PDE4D	ENSG00000113448	0.868

hsa-miR-3065-5p	XCR1	ENSG00000173578	0.867
hsa-miR-5680	RGPD6	ENSG00000183054	0.867
hsa-miR-410-3p	PIGA	ENSG00000165195	0.867
hsa-miR-3143	TENM1	ENSG00000009694	0.866
hsa-miR-4802-5p	PDE4D	ENSG00000113448	0.866
hsa-miR-5680	ADGRE3	ENSG00000131355	0.866
hsa-miR-1537-3p	SPC25	ENSG00000152253	0.865
hsa-miR-1908-5p	ZNF703	ENSG00000183779	0.864
hsa-miR-876-5p	SLC1A2	ENSG00000110436	0.864
hsa-miR-4677-5p	NT5DC4	ENSG00000144130	0.864
hsa-miR-876-5p	SRGAP1	ENSG00000196935	0.862
hsa-miR-3143	HEY2	ENSG00000135547	0.861
hsa-miR-4709-5p	FAM43A	ENSG00000185112	0.861
hsa-miR-3684	TLR10	ENSG00000174123	0.86
hsa-miR-942-5p	CHIT1	ENSG00000133063	0.86
hsa-miR-410-3p	ARHGAP29	ENSG00000137962	0.859
hsa-miR-4709-5p	RLIM	ENSG00000131263	0.858
hsa-miR-942-5p	ENC1	ENSG00000171617	0.858
hsa-miR-935	EML5	ENSG00000165521	0.858
hsa-miR-410-3p	CEP19	ENSG00000174007	0.858
hsa-miR-889-3p	FCRLB	ENSG00000162746	0.857
hsa-miR-1256	NEXMIF	ENSG00000050030	0.856
hsa-miR-410-3p	NEXMIF	ENSG00000050030	0.856
hsa-miR-641	TMEM170B	ENSG00000205269	0.855
hsa-miR-4724-5p	NDUFV2	ENSG00000178127	0.855
hsa-miR-4474-5p	ERMN	ENSG00000136541	0.855
hsa-miR-4724-5p	MASTL	ENSG00000120539	0.854
hsa-miR-410-3p	PRLR	ENSG00000113494	0.853
hsa-miR-935	PGAP1	ENSG00000197121	0.853
hsa-miR-889-3p	NRARP	ENSG00000198435	0.853
hsa-miR-580-3p	NCR3LG1	ENSG00000188211	0.853
hsa-miR-5680	GIMAP1	ENSG00000213203	0.852
hsa-miR-3143	ZBTB10	ENSG00000205189	0.851
hsa-miR-3065-5p	CALHM1	ENSG00000185933	0.851
hsa-miR-3143	NRARP	ENSG00000198435	0.85
hsa-miR-889-3p	JAM2	ENSG00000154721	0.85
hsa-miR-889-3p	GCSAM	ENSG00000174500	0.85
hsa-miR-4724-5p	ZBTB43	ENSG00000169155	0.849
hsa-miR-410-3p	SCML1	ENSG00000047634	0.849
hsa-miR-5680	PGAP1	ENSG00000197121	0.849
hsa-miR-3179	GPR68	ENSG00000119714	0.849
hsa-miR-580-3p	SIAH1	ENSG00000196470	0.848
hsa-miR-876-5p	KRT23	ENSG00000108244	0.848
hsa-miR-4999-5p	EFNA3	ENSG00000143590	0.848
hsa-miR-4474-5p	PRLR	ENSG00000113494	0.847
hsa-miR-410-3p	PGAP1	ENSG00000197121	0.847
hsa-miR-876-5p	IDO1	ENSG00000131203	0.847

hsa-miR-5680	SCN3B	ENSG00000166257	0.846
hsa-miR-942-5p	NEXMIF	ENSG00000050030	0.846
hsa-miR-511-5p	ERMN	ENSG00000136541	0.846
hsa-miR-3065-5p	ZNF805	ENSG00000204524	0.845
hsa-miR-410-3p	ZNF805	ENSG00000204524	0.845
hsa-miR-3065-5p	ZNF547	ENSG00000152433	0.845
hsa-miR-5582-5p	SRGAP1	ENSG00000196935	0.845
hsa-miR-410-3p	NRARP	ENSG00000198435	0.845
hsa-miR-5680	NEXMIF	ENSG00000050030	0.845
hsa-miR-1256	LRRIQ3	ENSG00000162620	0.845
hsa-miR-5695	ARL5B	ENSG00000165997	0.844
hsa-miR-371b-5p	ZNF14	ENSG00000105708	0.843
hsa-miR-93-5p	YES1	ENSG00000176105	0.843
hsa-miR-4775	SOX5	ENSG00000134532	0.843
hsa-miR-4659b-5p	PPP1R9A	ENSG00000158528	0.843
hsa-miR-19a-5p	NR4A3	ENSG00000119508	0.843
hsa-miR-544a	C4orf19	ENSG00000154274	0.843
hsa-miR-4802-5p	ZNF805	ENSG00000204524	0.842
hsa-miR-942-5p	TPO	ENSG00000115705	0.842
hsa-miR-4659b-5p	PKD2L2	ENSG00000078795	0.842
hsa-miR-15a-5p	PDK4	ENSG00000004799	0.842
hsa-miR-942-5p	MCM10	ENSG00000065328	0.842
hsa-miR-103a-3p	KIF18B	ENSG00000186185	0.842
hsa-miR-758-3p	RBFOX3	ENSG00000167281	0.841
hsa-miR-206	PRLR	ENSG00000113494	0.841
hsa-miR-3064-5p	PDE4D	ENSG00000113448	0.841
hsa-miR-4775	OTX1	ENSG00000115507	0.841
hsa-miR-942-5p	KLHL15	ENSG00000174010	0.841
hsa-miR-758-3p	FZD7	ENSG00000155760	0.841
hsa-miR-664a-3p	DEPDC1	ENSG00000024526	0.841
hsa-miR-199a-5p	ZNF781	ENSG00000196381	0.84
hsa-miR-199b-5p	ZNF781	ENSG00000196381	0.84
hsa-miR-4474-5p	SRGAP1	ENSG00000196935	0.84
hsa-miR-301a-3p	RANBP17	ENSG00000204764	0.84
hsa-miR-365b-5p	PRLR	ENSG00000113494	0.84
hsa-miR-340-5p	PDK4	ENSG00000004799	0.84
hsa-miR-3065-5p	OTX1	ENSG00000115507	0.84
hsa-miR-548ah-3p	KIF18B	ENSG00000186185	0.84
hsa-miR-664b-3p	GALNT5	ENSG00000136542	0.84
hsa-miR-30c-5p	ARHGAP29	ENSG00000137962	0.84
hsa-miR-513b-5p	ARHGAP29	ENSG00000137962	0.84
hsa-miR-5582-5p	ARHGAP29	ENSG00000137962	0.84
hsa-miR-548x-5p	TMEM250	ENSG00000238227	0.839
hsa-miR-4724-5p	TMEM170B	ENSG00000205269	0.839
hsa-miR-641	SLC1A2	ENSG00000110436	0.839
hsa-miR-1249-3p	SKOR1	ENSG00000188779	0.839
hsa-miR-4677-5p	RLIM	ENSG00000131263	0.839

hsa-miR-19a-5p	OTX1	ENSG00000115507	0.839
hsa-miR-4677-5p	ESM1	ENSG00000164283	0.839
hsa-miR-199a-5p	RORB	ENSG00000198963	0.838
hsa-miR-5680	PRLR	ENSG00000113494	0.838
hsa-miR-641	PIK3R1	ENSG00000145675	0.838
hsa-miR-147a	NEXMIF	ENSG00000050030	0.838
hsa-miR-664b-3p	MET	ENSG00000105976	0.838
hsa-miR-556-5p	RFX8	ENSG00000196460	0.837
hsa-miR-30a-3p	PPP1R9A	ENSG00000158528	0.837
hsa-miR-301a-3p	PGAP1	ENSG00000197121	0.837
hsa-miR-4677-5p	GRM3	ENSG00000198822	0.837
hsa-miR-641	CCNT1	ENSG00000129315	0.837
hsa-miR-410-3p	ZBTB43	ENSG00000169155	0.836
hsa-miR-129-5p	TMEM170B	ENSG00000205269	0.836
hsa-miR-15b-5p	SOX5	ENSG00000134532	0.836
hsa-miR-513b-5p	PIGA	ENSG00000165195	0.836
hsa-miR-98-5p	PARS2	ENSG00000162396	0.836
hsa-miR-3065-5p	PAQR8	ENSG00000170915	0.836
hsa-miR-942-5p	NT5DC4	ENSG00000144130	0.836
hsa-miR-3945	KLHL15	ENSG00000174010	0.836
hsa-miR-616-5p	FBLN5	ENSG00000140092	0.836
hsa-miR-942-5p	XCR1	ENSG00000173578	0.835
hsa-miR-3143	TMEM255A	ENSG00000125355	0.835
hsa-miR-574-5p	REL	ENSG00000162924	0.835
hsa-miR-98-5p	PRLR	ENSG00000113494	0.835
hsa-miR-548ah-3p	GRIK4	ENSG00000149403	0.835
hsa-miR-3179	TMEM170B	ENSG00000205269	0.834
hsa-miR-616-5p	TMEM170B	ENSG00000205269	0.834
hsa-miR-1299	TENM1	ENSG00000009694	0.834
hsa-miR-15a-5p	SOX5	ENSG00000134532	0.834
hsa-miR-92a-3p	SORCS3	ENSG00000156395	0.834
hsa-miR-15a-5p	SIAH1	ENSG00000196470	0.834
hsa-miR-3150a-3p	SH3D19	ENSG00000109686	0.834
hsa-miR-548ah-3p	PRLR	ENSG00000113494	0.834
hsa-miR-301a-3p	PRICKLE2	ENSG00000163637	0.834
hsa-miR-4494	PIM3	ENSG00000198355	0.834
hsa-miR-92a-3p	PDE4D	ENSG00000113448	0.834
hsa-miR-15b-5p	NRARP	ENSG00000198435	0.834
hsa-miR-504-5p	MOCS1	ENSG00000124615	0.834
hsa-miR-410-3p	MCM10	ENSG00000065328	0.834
hsa-miR-548x-5p	CFL2	ENSG00000165410	0.834
hsa-miR-129-5p	CCNT1	ENSG00000129315	0.834
hsa-miR-32-5p	ZBTB21	ENSG00000173276	0.833
hsa-miR-4999-5p	ZBTB21	ENSG00000173276	0.833
hsa-miR-365a-5p	TREML2	ENSG00000112195	0.833
hsa-miR-15b-5p	TMEM255A	ENSG00000125355	0.833
hsa-miR-4494	SSPN	ENSG00000123096	0.833

hsa-miR-1291	RNF152	ENSG00000176641	0.833
hsa-miR-4659b-5p	NR4A3	ENSG00000119508	0.833
hsa-miR-15b-5p	KIF18B	ENSG00000186185	0.833
hsa-miR-5680	KCNJ2	ENSG00000123700	0.833
hsa-miR-935	HEY2	ENSG00000135547	0.833
hsa-miR-4433a-3p	DBF4	ENSG00000006634	0.833
hsa-miR-539-5p	CTU1	ENSG00000142544	0.833
hsa-miR-204-5p	CNDP1	ENSG00000150656	0.833
hsa-miR-3150a-3p	ZNF703	ENSG00000183779	0.832
hsa-miR-4425	RGS20	ENSG00000147509	0.832
hsa-miR-365a-5p	PRLR	ENSG00000113494	0.832
hsa-miR-544b	PER1	ENSG00000179094	0.832
hsa-miR-32-5p	PDE4D	ENSG00000113448	0.832
hsa-miR-206	KLHL15	ENSG00000174010	0.832
hsa-miR-410-3p	KLHL15	ENSG00000174010	0.832
hsa-miR-500a-5p	CCSER1	ENSG00000184305	0.832
hsa-miR-454-3p	AHRR	ENSG00000063438	0.832
hsa-miR-3613-5p	TENM1	ENSG00000009694	0.831
hsa-miR-103a-2-5p	SLC23A3	ENSG00000213901	0.831
hsa-miR-4677-5p	SKA3	ENSG00000165480	0.831
hsa-miR-539-5p	PRLR	ENSG00000113494	0.831
hsa-miR-5695	GIMAP1	ENSG00000213203	0.831
hsa-miR-504-5p	FZD7	ENSG00000155760	0.831
hsa-miR-98-5p	ZBTB10	ENSG00000205189	0.83
hsa-miR-625-5p	SRGAP1	ENSG00000196935	0.83
hsa-miR-3065-5p	SIAH1	ENSG00000196470	0.83
hsa-miR-513b-5p	PRLR	ENSG00000113494	0.83
hsa-miR-15a-5p	NRARP	ENSG00000198435	0.83
hsa-miR-4775	MIXL1	ENSG00000185155	0.83
hsa-miR-340-5p	LRRIQ3	ENSG00000162620	0.83
hsa-miR-1299	KLHL15	ENSG00000174010	0.83
hsa-miR-193a-5p	FPR3	ENSG00000187474	0.83
hsa-miR-20a-5p	TENM1	ENSG00000009694	0.829
hsa-miR-548x-5p	SYNM	ENSG00000182253	0.829
hsa-miR-30a-3p	PRLR	ENSG00000113494	0.829
hsa-miR-548ah-5p	FEM1C	ENSG00000145780	0.829
hsa-miR-432-5p	CCSER1	ENSG00000184305	0.829
hsa-miR-3180	GRIN1	ENSG00000176884	0.828
hsa-miR-548a-5p	FEM1C	ENSG00000145780	0.828
hsa-miR-4677-5p	ZBTB21	ENSG00000173276	0.827
hsa-miR-548a-5p	ZBTB10	ENSG00000205189	0.827
hsa-miR-1299	XCR1	ENSG00000173578	0.827
hsa-miR-365b-5p	TREML2	ENSG00000112195	0.827
hsa-miR-548ah-5p	SRGAP1	ENSG00000196935	0.827
hsa-miR-551b-5p	RHOB	ENSG00000143878	0.827
hsa-miR-5680	RGS20	ENSG00000147509	0.827
hsa-miR-15a-5p	OTX1	ENSG00000115507	0.827

hsa-miR-590-5p	KLHL15	ENSG00000174010	0.827
hsa-miR-580-3p	HIF1A	ENSG00000100644	0.827
hsa-miR-371b-5p	ENC1	ENSG00000171617	0.827
hsa-miR-641	STEAP4	ENSG00000127954	0.826
hsa-miR-92a-3p	PIGA	ENSG00000165195	0.826
hsa-miR-3180-5p	NCR3LG1	ENSG00000188211	0.826
hsa-miR-513a-5p	MET	ENSG00000105976	0.826
hsa-miR-4775	IL1RL1	ENSG00000115602	0.826
hsa-miR-935	GJA3	ENSG00000121743	0.826
hsa-miR-5680	GALNT5	ENSG00000136542	0.826
hsa-miR-4746-5p	CDKN1C	ENSG00000129757	0.826
hsa-miR-548ah-5p	XCR1	ENSG00000173578	0.825
hsa-miR-5695	SMIM11A	ENSG00000205670	0.825
hsa-miR-4724-5p	RNF152	ENSG00000176641	0.825
hsa-miR-1972	RHBDF1	ENSG00000007384	0.825
hsa-miR-3605-5p	PAQR8	ENSG00000170915	0.825
hsa-miR-206	NR4A2	ENSG00000153234	0.825
hsa-miR-616-5p	GCSAM	ENSG00000174500	0.825
hsa-miR-935	CFL2	ENSG00000165410	0.825
hsa-miR-889-3p	ZNF804A	ENSG00000170396	0.824
hsa-miR-3143	ZBTB21	ENSG00000173276	0.824
hsa-miR-147a	VIT	ENSG00000205221	0.824
hsa-miR-92a-3p	SKOR1	ENSG00000188779	0.824
hsa-miR-942-5p	SKA3	ENSG00000165480	0.824
hsa-miR-548a-5p	RORB	ENSG00000198963	0.824
hsa-miR-190b	RLIM	ENSG00000131263	0.824
hsa-miR-371b-5p	PRR7	ENSG00000131188	0.824
hsa-miR-340-5p	OSM	ENSG00000099985	0.824
hsa-miR-616-3p	KIAA1324	ENSG00000116299	0.824
hsa-miR-340-5p	FZD7	ENSG00000155760	0.824
hsa-miR-144-3p	FOS	ENSG00000170345	0.824
hsa-miR-597-5p	FOS	ENSG00000170345	0.824
hsa-miR-3143	EREG	ENSG00000124882	0.824
hsa-miR-410-3p	CDKN1C	ENSG00000129757	0.824
hsa-miR-942-5p	TNNT1	ENSG00000105048	0.823
hsa-miR-548ah-5p	TNFRSF9	ENSG00000049249	0.823
hsa-miR-539-5p	GCSAM	ENSG00000174500	0.823
hsa-miR-15b-5p	ARL5B	ENSG00000165997	0.823
hsa-miR-501-5p	ZBTB10	ENSG00000205189	0.822
hsa-miR-432-5p	RORB	ENSG00000198963	0.822
hsa-miR-548ah-3p	RORB	ENSG00000198963	0.822
hsa-miR-616-5p	PDE4D	ENSG00000113448	0.822
hsa-miR-362-5p	NCR3LG1	ENSG00000188211	0.822
hsa-miR-199a-5p	GPR68	ENSG00000119714	0.822
hsa-miR-548a-5p	SYNM	ENSG00000182253	0.821
hsa-miR-664b-3p	SRGAP1	ENSG00000196935	0.821
hsa-miR-4717-5p	RORB	ENSG00000198963	0.821

hsa-miR-4775	PAQR8	ENSG00000170915	0.821
hsa-miR-3684	NR4A3	ENSG00000119508	0.821
hsa-miR-548ah-3p	NR1D2	ENSG00000174738	0.821
hsa-miR-876-5p	IFIT3	ENSG00000119917	0.821
hsa-miR-144-3p	GFPT2	ENSG00000131459	0.821
hsa-miR-1299	ERMN	ENSG00000136541	0.821
hsa-miR-340-5p	DIAPH3	ENSG00000139734	0.821
hsa-miR-3074-3p	CAV1	ENSG00000105974	0.821
hsa-miR-15a-5p	ARL5B	ENSG00000165997	0.821
hsa-miR-4999-5p	ARL5B	ENSG00000165997	0.821
hsa-miR-188-5p	YOD1	ENSG00000180667	0.82
hsa-miR-513b-5p	TREML2	ENSG00000112195	0.82
hsa-miR-449a	SOX5	ENSG00000134532	0.82
hsa-miR-590-5p	RFX8	ENSG00000196460	0.82
hsa-miR-98-5p	P4HA2	ENSG00000072682	0.82
hsa-miR-548ah-3p	LMOD2	ENSG00000170807	0.82
hsa-miR-4775	GRIK4	ENSG00000149403	0.82
hsa-miR-548a-5p	FNDC7	ENSG00000143107	0.82
hsa-miR-340-5p	E2F8	ENSG00000129173	0.82
hsa-miR-628-3p	DEPDC1	ENSG00000024526	0.82
hsa-miR-708-5p	DBF4	ENSG00000006634	0.82
hsa-miR-4999-5p	ZBTB43	ENSG00000169155	0.819
hsa-miR-196a-5p	RGPD6	ENSG00000183054	0.819
hsa-miR-103a-3p	RANBP17	ENSG00000204764	0.819
hsa-miR-708-5p	PRLR	ENSG00000113494	0.819
hsa-miR-103a-3p	PIK3R1	ENSG00000145675	0.819
hsa-miR-506-3p	NR4A3	ENSG00000119508	0.819
hsa-miR-374a-5p	NEK2	ENSG00000117650	0.819
hsa-miR-3065-5p	MET	ENSG00000105976	0.819
hsa-miR-506-3p	KIF18B	ENSG00000186185	0.819
hsa-miR-660-5p	HIF1A	ENSG00000100644	0.819
hsa-miR-664a-3p	DIAPH3	ENSG00000139734	0.819
hsa-miR-204-5p	TENM1	ENSG00000009694	0.818
hsa-miR-506-3p	TENM1	ENSG00000009694	0.818
hsa-miR-548ah-3p	SH3D19	ENSG00000109686	0.818
hsa-miR-889-3p	SCML1	ENSG00000047634	0.818
hsa-miR-374a-5p	RORB	ENSG00000198963	0.818
hsa-miR-506-3p	PRLR	ENSG00000113494	0.818
hsa-miR-548ah-3p	PPP1R1A	ENSG00000135447	0.818
hsa-miR-374a-5p	GCSAM	ENSG00000174500	0.818
hsa-miR-513b-5p	FAM217A	ENSG00000145975	0.818
hsa-miR-124-5p	CHD1	ENSG00000153922	0.818
hsa-miR-340-5p	CFL2	ENSG00000165410	0.818
hsa-miR-125b-5p	CACNA1E	ENSG00000198216	0.818
hsa-miR-5680	AHRR	ENSG00000063438	0.818
hsa-miR-548a-5p	YES1	ENSG00000176105	0.817
hsa-miR-574-5p	TIPARP	ENSG00000163659	0.817

hsa-miR-4775	STEAP4	ENSG00000127954	0.817
hsa-miR-340-5p	SCML1	ENSG00000047634	0.817
hsa-miR-124-3p	PRLR	ENSG00000113494	0.817
hsa-miR-5680	LRRN3	ENSG00000173114	0.817
hsa-miR-1972	LRRC70	ENSG00000186105	0.817
hsa-miR-3176	ABCB9	ENSG00000150967	0.817
hsa-miR-124-3p	TNFSF11	ENSG00000120659	0.816
hsa-miR-506-5p	STBD1	ENSG00000118804	0.816
hsa-miR-544b	SOX5	ENSG00000134532	0.816
hsa-miR-5680	PRICKLE2	ENSG00000163637	0.816
hsa-miR-513a-5p	PDK4	ENSG00000004799	0.816
hsa-miR-1299	KISS1R	ENSG00000116014	0.816
hsa-miR-548ah-3p	IL1RL1	ENSG00000115602	0.816
hsa-miR-548x-5p	FEM1C	ENSG00000145780	0.816
hsa-miR-4424	FASLG	ENSG00000117560	0.816
hsa-miR-153-3p	ERRFI1	ENSG00000116285	0.816
hsa-miR-506-3p	DEPDC1	ENSG00000024526	0.816
hsa-miR-616-5p	BTG3	ENSG00000154640	0.816
hsa-miR-641	ARL5B	ENSG00000165997	0.816
hsa-miR-1299	YOD1	ENSG00000180667	0.815
hsa-miR-103a-3p	RHBDF1	ENSG00000007384	0.815
hsa-miR-129-5p	OTX1	ENSG00000115507	0.815
hsa-miR-129-5p	KLHL15	ENSG00000174010	0.815
hsa-miR-548x-5p	KLHL15	ENSG00000174010	0.815
hsa-miR-548ah-5p	ZNF805	ENSG00000204524	0.814
hsa-miR-147a	TICRR	ENSG00000140534	0.814
hsa-miR-548ah-3p	TENM1	ENSG00000009694	0.814
hsa-miR-548x-5p	SDC1	ENSG00000115884	0.814
hsa-miR-3684	KLHL15	ENSG00000174010	0.814
hsa-miR-885-5p	GPR68	ENSG00000119714	0.814
hsa-miR-32-5p	DUSP1	ENSG00000120129	0.814
hsa-miR-17-3p	ZNF547	ENSG00000152433	0.813
hsa-miR-628-5p	ZMAT4	ENSG00000165061	0.813
hsa-miR-20a-5p	ZBTB43	ENSG00000169155	0.813
hsa-miR-206	ZBTB21	ENSG00000173276	0.813
hsa-miR-30a-3p	STBD1	ENSG00000118804	0.813
hsa-miR-4425	SLC23A3	ENSG00000213901	0.813
hsa-miR-664b-3p	RGPD5	ENSG00000015568	0.813
hsa-miR-942-5p	FPR3	ENSG00000187474	0.813
hsa-miR-539-5p	CCNT1	ENSG00000129315	0.813
hsa-miR-551b-5p	CACNA1E	ENSG00000198216	0.813
hsa-miR-3065-5p	BTN1A1	ENSG00000124557	0.813
hsa-miR-544b	VIT	ENSG00000205221	0.812
hsa-miR-92a-3p	TNFAIP6	ENSG00000123610	0.812
hsa-miR-4659b-5p	TLR10	ENSG00000174123	0.812
hsa-miR-4775	TEX12	ENSG00000150783	0.812
hsa-miR-5680	SYN1	ENSG00000008056	0.812

hsa-miR-664a-3p	SPP1	ENSG00000118785	0.812
hsa-miR-548a-5p	SLC12A1	ENSG00000074803	0.812
hsa-miR-4425	SH3D19	ENSG00000109686	0.812
hsa-miR-340-5p	REL	ENSG00000162924	0.812
hsa-miR-4425	NPTX2	ENSG00000106236	0.812
hsa-miR-544a	LMOD2	ENSG00000170807	0.812
hsa-miR-4775	DEPDC1	ENSG00000024526	0.812
hsa-miR-548ah-3p	ZNF331	ENSG00000130844	0.811
hsa-miR-544a	STBD1	ENSG00000118804	0.811
hsa-miR-147a	SSPN	ENSG00000123096	0.811
hsa-miR-3613-5p	SOX5	ENSG00000134532	0.811
hsa-miR-10a-5p	RTN4R	ENSG00000040608	0.811
hsa-miR-103a-3p	RNF152	ENSG00000176641	0.811
hsa-miR-5695	RNF152	ENSG00000176641	0.811
hsa-miR-758-3p	PIK3R1	ENSG00000145675	0.811
hsa-miR-548ah-3p	HIF1A	ENSG00000100644	0.811
hsa-miR-574-5p	FAM81B	ENSG00000153347	0.811
hsa-miR-552-3p	ENC1	ENSG00000171617	0.811
hsa-miR-5582-5p	EML5	ENSG00000165521	0.811
hsa-miR-410-3p	DIAPH3	ENSG00000139734	0.811
hsa-miR-4491	ANKRD18A	ENSG00000180071	0.811
hsa-miR-4474-5p	TNFSF11	ENSG00000120659	0.81
hsa-miR-889-3p	SERTAD2	ENSG00000179833	0.81
hsa-miR-101-3p	SEMA3G	ENSG00000010319	0.81
hsa-miR-3180-5p	PGAP1	ENSG00000197121	0.81
hsa-miR-3180-5p	DBF4	ENSG00000006634	0.81
hsa-miR-513a-5p	CFL2	ENSG00000165410	0.81
hsa-miR-371b-5p	CEBPB	ENSG00000172216	0.81
hsa-miR-4494	CCNT1	ENSG00000129315	0.81
hsa-miR-4494	CACNA1E	ENSG00000198216	0.81
hsa-miR-758-3p	ZBTB21	ENSG00000173276	0.809
hsa-miR-129-5p	TLR10	ENSG00000174123	0.809
hsa-miR-374a-5p	SMAD9	ENSG00000120693	0.809
hsa-miR-4775	SIAH1	ENSG00000196470	0.809
hsa-miR-374a-5p	SERTAD2	ENSG00000179833	0.809
hsa-miR-4724-5p	PDK4	ENSG00000004799	0.809
hsa-miR-548ah-3p	NEXMIF	ENSG00000050030	0.809
hsa-miR-17-3p	LRFN1	ENSG00000128011	0.809
hsa-miR-502-5p	FLRT1	ENSG00000126500	0.809
hsa-miR-616-5p	ERMN	ENSG00000136541	0.809
hsa-miR-153-3p	CCSER1	ENSG00000184305	0.809
hsa-miR-3150a-3p	B3GNT7	ENSG00000156966	0.809
hsa-miR-660-5p	AOC3	ENSG00000131471	0.809
hsa-miR-20a-5p	TMEM255A	ENSG00000125355	0.808
hsa-miR-340-5p	TEX12	ENSG00000150783	0.808
hsa-miR-410-3p	SLC1A2	ENSG00000110436	0.808
hsa-miR-548ah-5p	SKOR1	ENSG00000188779	0.808

hsa-miR-144-3p	RNF152	ENSG00000176641	0.808
hsa-miR-4802-5p	PRICKLE2	ENSG00000163637	0.808
hsa-miR-664b-3p	PDK4	ENSG00000004799	0.808
hsa-miR-539-5p	HEY2	ENSG00000135547	0.808
hsa-miR-548ah-5p	EREG	ENSG00000124882	0.808
hsa-miR-9-5p	CHRNB2	ENSG00000160716	0.808
hsa-miR-10a-5p	ANKRD18A	ENSG00000180071	0.808
hsa-miR-153-3p	ZNF703	ENSG00000183779	0.807
hsa-miR-513b-5p	TPO	ENSG00000115705	0.807
hsa-miR-15b-5p	OTX1	ENSG00000115507	0.807
hsa-miR-500a-5p	NR4A3	ENSG00000119508	0.807
hsa-miR-548x-5p	FZD7	ENSG00000155760	0.807
hsa-miR-454-5p	FFAR2	ENSG00000126262	0.807
hsa-miR-876-5p	ARL5B	ENSG00000165997	0.807
hsa-miR-641	ZNF804A	ENSG00000170396	0.806
hsa-miR-362-5p	TMEM170B	ENSG00000205269	0.806
hsa-miR-193a-5p	TIPARP	ENSG00000163659	0.806
hsa-miR-4423-5p	SRGAP1	ENSG00000196935	0.806
hsa-miR-15b-5p	RNF152	ENSG00000176641	0.806
hsa-miR-4425	PPP1R9A	ENSG00000158528	0.806
hsa-miR-4482-5p	KCNJ2	ENSG00000123700	0.806
hsa-miR-454-3p	IER5L	ENSG00000188483	0.806
hsa-miR-548a-5p	CCSER1	ENSG00000184305	0.806
hsa-miR-664b-3p	ZNF805	ENSG00000204524	0.805
hsa-miR-556-5p	XCR1	ENSG00000173578	0.805
hsa-miR-101-3p	NR4A3	ENSG00000119508	0.805
hsa-miR-30c-5p	NR1D2	ENSG00000174738	0.805
hsa-miR-218-5p	KCNJ2	ENSG00000123700	0.805
hsa-miR-4775	HEY2	ENSG00000135547	0.805
hsa-miR-3065-5p	DBF4	ENSG00000006634	0.805
hsa-miR-103a-3p	CNDP1	ENSG00000150656	0.805
hsa-miR-30c-5p	ZNF547	ENSG00000152433	0.804
hsa-miR-1291	XCR1	ENSG00000173578	0.804
hsa-miR-885-5p	SKA3	ENSG00000165480	0.804
hsa-miR-362-5p	NEXMIF	ENSG00000050030	0.804
hsa-miR-124-5p	KCNJ2	ENSG00000123700	0.804
hsa-miR-3064-5p	ZBTB10	ENSG00000205189	0.803
hsa-miR-4425	SOX5	ENSG00000134532	0.803
hsa-miR-548s	SOX5	ENSG00000134532	0.803
hsa-miR-4474-5p	SLC1A2	ENSG00000110436	0.803
hsa-miR-580-3p	SLC1A2	ENSG00000110436	0.803
hsa-miR-548a-5p	RBM44	ENSG00000177483	0.803
hsa-miR-371b-5p	PDE4D	ENSG00000113448	0.803
hsa-miR-374a-5p	MET	ENSG00000105976	0.803
hsa-miR-3180-5p	ARL5B	ENSG00000165997	0.803
hsa-miR-3143	ZNF805	ENSG00000204524	0.802
hsa-miR-5582-5p	ZNF331	ENSG00000130844	0.802

hsa-miR-3179	SORCS3	ENSG00000156395	0.802
hsa-miR-20a-5p	SMAD6	ENSG00000137834	0.802
hsa-miR-616-3p	PRLR	ENSG00000113494	0.802
hsa-miR-500a-5p	PGAP1	ENSG00000197121	0.802
hsa-miR-506-3p	PAQR8	ENSG00000170915	0.802
hsa-miR-1299	HES1	ENSG00000114315	0.802
hsa-miR-5695	BTN1A1	ENSG00000124557	0.802
hsa-miR-3150a-3p	ANKRD18A	ENSG00000180071	0.802
hsa-miR-889-3p	ZNF331	ENSG00000130844	0.801
hsa-miR-758-3p	TKTL2	ENSG00000151005	0.801
hsa-miR-15a-5p	SLC1A2	ENSG00000110436	0.801
hsa-miR-9-5p	RBM11	ENSG00000185272	0.801
hsa-miR-17-3p	PPP1R1A	ENSG00000135447	0.801
hsa-miR-125b-5p	GJB7	ENSG00000164411	0.801
hsa-miR-5680	CTU1	ENSG00000142544	0.801
hsa-miR-188-5p	CNDP1	ENSG00000150656	0.801
hsa-miR-889-3p	STEAP4	ENSG00000127954	0.8
hsa-miR-4775	SEC14L2	ENSG00000100003	0.8
hsa-miR-374a-5p	SCML1	ENSG00000047634	0.8
hsa-miR-3143	NEXMIF	ENSG00000050030	0.8
hsa-miR-3143	CCDC121	ENSG00000176714	0.8

ST_10	liver_tissue_miRNA/mRNA_pairs		
miRNA	mRNA_gene_symbol	mRNA_acc	pairScore
hsa-miR-101-3p	BIRC5	ENSG00000089685	1
hsa-miR-101-3p	DUSP1	ENSG00000120129	1
hsa-miR-101-3p	GFPT2	ENSG00000131459	1
hsa-miR-101-3p	ZBTB21	ENSG00000173276	1
hsa-miR-101-3p	TMEM170B	ENSG00000205269	1
hsa-miR-101-3p	RNF152	ENSG00000176641	1
hsa-miR-101-3p	MET	ENSG00000105976	1
hsa-miR-101-3p	REL	ENSG00000162924	1
hsa-miR-103a-2-5p	CEBPD	ENSG00000221869	1
hsa-miR-103a-2-5p	EREG	ENSG00000124882	1
hsa-miR-103a-3p	CCNT1	ENSG00000129315	1
hsa-miR-103a-3p	PIK3R1	ENSG00000145675	1
hsa-miR-103a-3p	CAV1	ENSG00000105974	1
hsa-miR-103a-3p	PER1	ENSG00000179094	1
hsa-miR-103a-3p	FEM1C	ENSG00000145780	1
hsa-miR-103a-3p	PDE4D	ENSG00000113448	1
hsa-miR-103a-3p	REL	ENSG00000162924	1
hsa-miR-103a-3p	CD180	ENSG00000134061	1
hsa-miR-103a-3p	ZBTB10	ENSG00000205189	1
hsa-miR-103a-3p	PDK4	ENSG00000004799	1
hsa-miR-103a-3p	TMEM255A	ENSG00000125355	1
hsa-miR-10a-5p	RLIM	ENSG00000131263	1
hsa-miR-10a-5p	YES1	ENSG00000176105	1
hsa-miR-10a-5p	LRFN1	ENSG00000128011	1
hsa-miR-10a-5p	ZBTB10	ENSG00000205189	1
hsa-miR-10a-5p	ERMN	ENSG00000136541	1
hsa-miR-10a-5p	BIRC5	ENSG00000089685	1
hsa-miR-10a-5p	NR1D2	ENSG00000174738	1
hsa-miR-10a-5p	YOD1	ENSG00000180667	1
hsa-miR-1179	ATF3	ENSG00000162772	1
hsa-miR-1179	ARHGAP39	ENSG00000147799	1
hsa-miR-1179	ARL5B	ENSG00000165997	1
hsa-miR-122-5p	TMEM250	ENSG00000238227	1
hsa-miR-122-5p	REL	ENSG00000162924	1
hsa-miR-122-5p	SOCS1	ENSG00000185338	1
hsa-miR-122-5p	BIRC5	ENSG00000089685	1
hsa-miR-122-5p	CLEC11A	ENSG00000105472	1
hsa-miR-122-5p	CD83	ENSG00000112149	1
hsa-miR-122-5p	CNDP1	ENSG00000150656	1
hsa-miR-122-5p	BATF2	ENSG00000168062	1
hsa-miR-122-5p	MASTL	ENSG00000120539	1
hsa-miR-122-5p	SLC7A5	ENSG00000103257	1
hsa-miR-122-5p	CCR6	ENSG00000112486	1
hsa-miR-1228-3p	KLF2	ENSG00000127528	1
hsa-miR-1228-3p	TCTE1	ENSG00000146221	1

hsa-miR-1228-3p	CEBPD	ENSG00000221869	1
hsa-miR-1228-3p	PER1	ENSG00000179094	1
hsa-miR-1234-3p	FOS	ENSG00000170345	1
hsa-miR-1234-3p	ZBTB43	ENSG00000169155	1
hsa-miR-124-3p	AHRR	ENSG00000063438	1
hsa-miR-124-3p	ZNF483	ENSG00000173258	1
hsa-miR-124-3p	ADAMTS1	ENSG00000154734	1
hsa-miR-124-3p	AURKA	ENSG00000087586	1
hsa-miR-124-3p	COL1A1	ENSG00000108821	1
hsa-miR-124-3p	ID1	ENSG00000125968	1
hsa-miR-124-3p	IFIT3	ENSG00000119917	1
hsa-miR-124-3p	EREG	ENSG00000124882	1
hsa-miR-124-3p	TOM1L1	ENSG00000141198	1
hsa-miR-124-3p	GFPT2	ENSG00000131459	1
hsa-miR-124-3p	ZNF626	ENSG00000188171	1
hsa-miR-124-3p	GCSAML	ENSG00000169224	1
hsa-miR-124-3p	TNFRSF12A	ENSG00000006327	1
hsa-miR-124-3p	VIT	ENSG00000205221	1
hsa-miR-124-3p	SDE2	ENSG00000143751	1
hsa-miR-124-3p	SC5D	ENSG00000109929	1
hsa-miR-124-3p	DGAT2	ENSG00000062282	1
hsa-miR-124-3p	CAV1	ENSG00000105974	1
hsa-miR-124-3p	DEPDC1	ENSG00000024526	1
hsa-miR-124-3p	RHBDF1	ENSG00000007384	1
hsa-miR-124-3p	ARHGAP29	ENSG00000137962	1
hsa-miR-124-3p	KLF2	ENSG00000127528	1
hsa-miR-124-3p	CCDC121	ENSG00000176714	1
hsa-miR-124-3p	BTG3	ENSG00000154640	1
hsa-miR-124-3p	SERTAD2	ENSG00000179833	1
hsa-miR-124-3p	NR4A3	ENSG00000119508	1
hsa-miR-124-3p	ARL5B	ENSG00000165997	1
hsa-miR-124-3p	GDF10	ENSG00000266524	1
hsa-miR-124-3p	RPP25L	ENSG00000164967	1
hsa-miR-124-3p	IFIT2	ENSG00000119922	1
hsa-miR-124-3p	CXCL8	ENSG00000169429	1
hsa-miR-1249-3p	PRLR	ENSG00000113494	1
hsa-miR-1249-5p	ARL5B	ENSG00000165997	1
hsa-miR-1249-5p	SLC7A5	ENSG00000103257	1
hsa-miR-125b-5p	TNFAIP3	ENSG00000118503	1
hsa-miR-125b-5p	YOD1	ENSG00000180667	1
hsa-miR-125b-5p	GJB7	ENSG00000164411	1
hsa-miR-125b-5p	NRARP	ENSG00000198435	1
hsa-miR-125b-5p	AHRR	ENSG00000063438	1
hsa-miR-125b-5p	ZNF483	ENSG00000173258	1
hsa-miR-129-5p	KLHL15	ENSG00000174010	1
hsa-miR-129-5p	DUSP1	ENSG00000120129	1
hsa-miR-129-5p	CCR6	ENSG00000112486	1

hsa-miR-129-5p	YES1	ENSG00000176105	1
hsa-miR-129-5p	ZNF285	ENSG00000267508	1
hsa-miR-129-5p	ICOSLG	ENSG00000160223	1
hsa-miR-129-5p	ZNF703	ENSG00000183779	1
hsa-miR-129-5p	CKAP2L	ENSG00000169607	1
hsa-miR-1299	KLHL15	ENSG00000174010	1
hsa-miR-1299	YOD1	ENSG00000180667	1
hsa-miR-1303	MET	ENSG00000105976	1
hsa-miR-1303	NLRP9	ENSG00000185792	1
hsa-miR-1303	KIR3DX1	ENSG00000104970	1
hsa-miR-1303	YOD1	ENSG00000180667	1
hsa-miR-144-3p	ARL5B	ENSG00000165997	1
hsa-miR-144-3p	MET	ENSG00000105976	1
hsa-miR-144-3p	YOD1	ENSG00000180667	1
hsa-miR-144-5p	PIGA	ENSG00000165195	1
hsa-miR-153-3p	ZBTB43	ENSG00000169155	1
hsa-miR-153-3p	ZNF703	ENSG00000183779	1
hsa-miR-153-3p	FEM1C	ENSG00000145780	1
hsa-miR-15a-5p	TNFSF9	ENSG00000125657	1
hsa-miR-15a-5p	SLC7A5	ENSG00000103257	1
hsa-miR-15a-5p	PRICKLE2	ENSG00000163637	1
hsa-miR-15a-5p	PDE4D	ENSG00000113448	1
hsa-miR-15a-5p	HSPA1A	ENSG00000204389	1
hsa-miR-15a-5p	CD180	ENSG00000134061	1
hsa-miR-15a-5p	CCNT1	ENSG00000129315	1
hsa-miR-15a-5p	SOX5	ENSG00000134532	1
hsa-miR-15a-5p	KLHL15	ENSG00000174010	1
hsa-miR-15a-5p	REL	ENSG00000162924	1
hsa-miR-15a-5p	SIK1	ENSG00000142178	1
hsa-miR-15a-5p	RGPD5	ENSG00000015568	1
hsa-miR-15a-5p	ZBTB10	ENSG00000205189	1
hsa-miR-15a-5p	PIK3R1	ENSG00000145675	1
hsa-miR-15b-5p	TNFSF9	ENSG00000125657	1
hsa-miR-15b-5p	KLHL15	ENSG00000174010	1
hsa-miR-15b-5p	SIK1	ENSG00000142178	1
hsa-miR-15b-5p	REL	ENSG00000162924	1
hsa-miR-15b-5p	RLIM	ENSG00000131263	1
hsa-miR-15b-5p	CD180	ENSG00000134061	1
hsa-miR-15b-5p	PDE4D	ENSG00000113448	1
hsa-miR-15b-5p	PIK3R1	ENSG00000145675	1
hsa-miR-15b-5p	CCNT1	ENSG00000129315	1
hsa-miR-15b-5p	ZBTB10	ENSG00000205189	1
hsa-miR-15b-5p	PRICKLE2	ENSG00000163637	1
hsa-miR-15b-5p	SLC7A5	ENSG00000103257	1
hsa-miR-17-5p	SMAD6	ENSG00000137834	1
hsa-miR-17-5p	C14orf119	ENSG00000179933	1
hsa-miR-17-5p	YOD1	ENSG00000180667	1

hsa-miR-17-5p	YES1	ENSG00000176105	1
hsa-miR-17-5p	HIF1A	ENSG00000100644	1
hsa-miR-17-5p	SIK1	ENSG00000142178	1
hsa-miR-17-5p	NAT8L	ENSG00000185818	1
hsa-miR-17-5p	BTG3	ENSG00000154640	1
hsa-miR-17-5p	CFL2	ENSG00000165410	1
hsa-miR-17-5p	RLIM	ENSG00000131263	1
hsa-miR-17-5p	ATF3	ENSG00000162772	1
hsa-miR-17-5p	UBE2C	ENSG00000175063	1
hsa-miR-17-5p	PER1	ENSG00000179094	1
hsa-miR-17-5p	KLHL15	ENSG00000174010	1
hsa-miR-17-5p	DEPDC1	ENSG00000024526	1
hsa-miR-17-5p	MASTL	ENSG00000120539	1
hsa-miR-17-5p	MIXL1	ENSG00000185155	1
hsa-miR-17-5p	PKMYT1	ENSG00000127564	1
hsa-miR-17-5p	FEM1C	ENSG00000145780	1
hsa-miR-17-5p	ZNF805	ENSG00000204524	1
hsa-miR-17-5p	FTH1	ENSG00000167996	1
hsa-miR-17-5p	CAV1	ENSG00000105974	1
hsa-miR-187-3p	CENPA	ENSG00000115163	1
hsa-miR-187-5p	FOS	ENSG00000170345	1
hsa-miR-187-5p	ZNF805	ENSG00000204524	1
hsa-miR-188-5p	TMEM170B	ENSG00000205269	1
hsa-miR-188-5p	CDC20	ENSG00000117399	1
hsa-miR-188-5p	ADRB1	ENSG00000043591	1
hsa-miR-188-5p	REL	ENSG00000162924	1
hsa-miR-193a-3p	BUB1	ENSG00000169679	1
hsa-miR-193a-3p	SLC7A5	ENSG00000103257	1
hsa-miR-193a-3p	AURKA	ENSG00000087586	1
hsa-miR-193b-3p	BUB1	ENSG00000169679	1
hsa-miR-193b-3p	CKAP2L	ENSG00000169607	1
hsa-miR-193b-3p	TICRR	ENSG00000140534	1
hsa-miR-193b-3p	CHCHD10	ENSG00000250479	1
hsa-miR-193b-3p	CDC20	ENSG00000117399	1
hsa-miR-193b-3p	NAT8L	ENSG00000185818	1
hsa-miR-193b-3p	NRARP	ENSG00000198435	1
hsa-miR-193b-3p	UBE2C	ENSG00000175063	1
hsa-miR-193b-3p	MCM10	ENSG00000065328	1
hsa-miR-193b-3p	SKA3	ENSG00000165480	1
hsa-miR-193b-3p	ZBTB43	ENSG00000169155	1
hsa-miR-193b-3p	TMEM204	ENSG00000131634	1
hsa-miR-193b-3p	SLC7A5	ENSG00000103257	1
hsa-miR-193b-3p	APOBEC3B	ENSG00000179750	1
hsa-miR-193b-3p	PKMYT1	ENSG00000127564	1
hsa-miR-193b-3p	ERRFI1	ENSG00000116285	1
hsa-miR-193b-3p	SPC25	ENSG00000152253	1
hsa-miR-196a-5p	KCNJ2	ENSG00000123700	1

hsa-miR-196a-5p	UBE2C	ENSG00000175063	1
hsa-miR-196a-5p	BUB1	ENSG00000169679	1
hsa-miR-196a-5p	YOD1	ENSG00000180667	1
hsa-miR-196a-5p	KIF18B	ENSG00000186185	1
hsa-miR-196a-5p	GRIK4	ENSG00000149403	1
hsa-miR-196a-5p	CKAP2L	ENSG00000169607	1
hsa-miR-199a-5p	APOE	ENSG00000130203	1
hsa-miR-199a-5p	HIF1A	ENSG00000100644	1
hsa-miR-199a-5p	CAV1	ENSG00000105974	1
hsa-miR-199a-5p	RND1	ENSG00000172602	1
hsa-miR-199b-5p	HIF1A	ENSG00000100644	1
hsa-miR-199b-5p	HES1	ENSG00000114315	1
hsa-miR-19a-5p	RLIM	ENSG00000131263	1
hsa-miR-19a-5p	NR4A3	ENSG00000119508	1
hsa-miR-19a-5p	FOS	ENSG00000170345	1
hsa-miR-19a-5p	KLHL15	ENSG00000174010	1
hsa-miR-19a-5p	TNFAIP6	ENSG00000123610	1
hsa-miR-204-5p	ARHGAP29	ENSG00000137962	1
hsa-miR-204-5p	PRLR	ENSG00000113494	1
hsa-miR-204-5p	CXCL8	ENSG00000169429	1
hsa-miR-204-5p	STEAP4	ENSG00000127954	1
hsa-miR-204-5p	RORB	ENSG00000198963	1
hsa-miR-204-5p	SRGAP1	ENSG00000196935	1
hsa-miR-204-5p	ZBTB43	ENSG00000169155	1
hsa-miR-204-5p	PDF	ENSG00000258429	1
hsa-miR-204-5p	SMAD6	ENSG00000137834	1
hsa-miR-206	MET	ENSG00000105976	1
hsa-miR-206	KCNJ2	ENSG00000123700	1
hsa-miR-20a-5p	ZNF331	ENSG00000130844	1
hsa-miR-20a-5p	MIXL1	ENSG00000185155	1
hsa-miR-20a-5p	CAV1	ENSG00000105974	1
hsa-miR-20a-5p	YOD1	ENSG00000180667	1
hsa-miR-20a-5p	KLHL15	ENSG00000174010	1
hsa-miR-20a-5p	C14orf119	ENSG00000179933	1
hsa-miR-20a-5p	SIK1	ENSG00000142178	1
hsa-miR-20a-5p	MASTL	ENSG00000120539	1
hsa-miR-20a-5p	RLIM	ENSG00000131263	1
hsa-miR-20a-5p	BTG3	ENSG00000154640	1
hsa-miR-20a-5p	HIF1A	ENSG00000100644	1
hsa-miR-20a-5p	ZNF805	ENSG00000204524	1
hsa-miR-20a-5p	SMAD6	ENSG00000137834	1
hsa-miR-20a-5p	FEM1C	ENSG00000145780	1
hsa-miR-20a-5p	UBE2C	ENSG00000175063	1
hsa-miR-20a-5p	CFL2	ENSG00000165410	1
hsa-miR-20a-5p	PKMYT1	ENSG00000127564	1
hsa-miR-21-5p	PIK3R1	ENSG00000145675	1
hsa-miR-21-5p	YOD1	ENSG00000180667	1

hsa-miR-21-5p	SOX5	ENSG00000134532	1
hsa-miR-21-5p	RNF103	ENSG00000239305	1
hsa-miR-21-5p	DUSP8	ENSG00000184545	1
hsa-miR-21-5p	PRICKLE2	ENSG00000163637	1
hsa-miR-21-5p	HIF1A	ENSG00000100644	1
hsa-miR-21-5p	FASLG	ENSG00000117560	1
hsa-miR-21-5p	KLHL15	ENSG00000174010	1
hsa-miR-21-5p	RHOB	ENSG00000143878	1
hsa-miR-218-5p	CCDC74A	ENSG00000163040	1
hsa-miR-218-5p	ASB2	ENSG00000100628	1
hsa-miR-218-5p	SEMA3G	ENSG00000010319	1
hsa-miR-218-5p	BIRC5	ENSG00000089685	1
hsa-miR-218-5p	MET	ENSG00000105976	1
hsa-miR-218-5p	RHOB	ENSG00000143878	1
hsa-miR-218-5p	HJURP	ENSG00000123485	1
hsa-miR-218-5p	PRLR	ENSG00000113494	1
hsa-miR-218-5p	DGAT2	ENSG00000062282	1
hsa-miR-219a-5p	CEP19	ENSG00000174007	1
hsa-miR-24-1-5p	SLC23A3	ENSG00000213901	1
hsa-miR-24-3p	YOD1	ENSG00000180667	1
hsa-miR-24-3p	SCML1	ENSG00000047634	1
hsa-miR-24-3p	RHOF	ENSG00000139725	1
hsa-miR-24-3p	MCM10	ENSG00000065328	1
hsa-miR-24-3p	YES1	ENSG00000176105	1
hsa-miR-24-3p	DBF4	ENSG00000006634	1
hsa-miR-24-3p	PDF	ENSG00000258429	1
hsa-miR-24-3p	UBE2C	ENSG00000175063	1
hsa-miR-24-3p	SLC1A2	ENSG00000110436	1
hsa-miR-24-3p	AURKA	ENSG00000087586	1
hsa-miR-24-3p	KLHL15	ENSG00000174010	1
hsa-miR-24-3p	KIF18B	ENSG00000186185	1
hsa-miR-24-3p	TNFAIP3	ENSG00000118503	1
hsa-miR-24-3p	HBQ1	ENSG00000086506	1
hsa-miR-24-3p	DEPDC1	ENSG00000024526	1
hsa-miR-24-3p	ARHGAP39	ENSG00000147799	1
hsa-miR-24-3p	ATF3	ENSG00000162772	1
hsa-miR-301a-3p	DEPDC1	ENSG00000024526	1
hsa-miR-301a-3p	MASTL	ENSG00000120539	1
hsa-miR-301a-3p	CFL2	ENSG00000165410	1
hsa-miR-301a-3p	CCR6	ENSG00000112486	1
hsa-miR-301a-3p	SIK1	ENSG00000142178	1
hsa-miR-301a-3p	PIGA	ENSG00000165195	1
hsa-miR-301a-3p	RLIM	ENSG00000131263	1
hsa-miR-3074-3p	PER1	ENSG00000179094	1
hsa-miR-3074-3p	HIF1A	ENSG00000100644	1
hsa-miR-3074-3p	TMEM170B	ENSG00000205269	1
hsa-miR-30a-3p	ATF3	ENSG00000162772	1

hsa-miR-30a-3p	HSPA6	ENSG00000173110	1
hsa-miR-30a-3p	CACNA1E	ENSG00000198216	1
hsa-miR-30a-3p	SLC1A2	ENSG00000110436	1
hsa-miR-30a-3p	ARL2BP	ENSG00000102931	1
hsa-miR-30c-5p	BIRC5	ENSG00000089685	1
hsa-miR-30c-5p	SDE2	ENSG00000143751	1
hsa-miR-30c-5p	GCSAM	ENSG00000174500	1
hsa-miR-30c-5p	TXNDC5	ENSG00000239264	1
hsa-miR-30c-5p	CFL2	ENSG00000165410	1
hsa-miR-30c-5p	MYBL2	ENSG00000101057	1
hsa-miR-30c-5p	KLHL15	ENSG00000174010	1
hsa-miR-30c-5p	FAM81B	ENSG00000153347	1
hsa-miR-30c-5p	DBF4	ENSG00000006634	1
hsa-miR-30c-5p	SOCS1	ENSG00000185338	1
hsa-miR-30c-5p	SLC7A5	ENSG00000103257	1
hsa-miR-30c-5p	CHD1	ENSG00000153922	1
hsa-miR-3150a-3p	ICOSLG	ENSG00000160223	1
hsa-miR-3150a-3p	ASCL2	ENSG00000183734	1
hsa-miR-3150a-5p	PIM3	ENSG00000198355	1
hsa-miR-3176	SLC1A2	ENSG00000110436	1
hsa-miR-3180	ASCL2	ENSG00000183734	1
hsa-miR-3180	FAM43A	ENSG00000185112	1
hsa-miR-3180-5p	ATF3	ENSG00000162772	1
hsa-miR-3180-5p	TMEM169	ENSG00000163449	1
hsa-miR-3180-5p	ARL5B	ENSG00000165997	1
hsa-miR-32-5p	NLRP9	ENSG00000185792	1
hsa-miR-32-5p	RNF103	ENSG00000239305	1
hsa-miR-32-5p	REL	ENSG00000162924	1
hsa-miR-32-5p	GFPT2	ENSG00000131459	1
hsa-miR-32-5p	SERTAD2	ENSG00000179833	1
hsa-miR-32-5p	SMAD6	ENSG00000137834	1
hsa-miR-32-5p	SIK1	ENSG00000142178	1
hsa-miR-32-5p	AURKA	ENSG00000087586	1
hsa-miR-32-5p	KLHL15	ENSG00000174010	1
hsa-miR-32-5p	FASLG	ENSG00000117560	1
hsa-miR-340-3p	MRPL12	ENSG00000262814	1
hsa-miR-340-5p	SERTAD2	ENSG00000179833	1
hsa-miR-340-5p	ARL5B	ENSG00000165997	1
hsa-miR-340-5p	BTG3	ENSG00000154640	1
hsa-miR-340-5p	MET	ENSG00000105976	1
hsa-miR-340-5p	PDE4D	ENSG00000113448	1
hsa-miR-340-5p	BUB1	ENSG00000169679	1
hsa-miR-340-5p	KLHL15	ENSG00000174010	1
hsa-miR-340-5p	DEPDC1	ENSG00000024526	1
hsa-miR-340-5p	PIM3	ENSG00000198355	1
hsa-miR-3605-5p	MRPL12	ENSG00000262814	1
hsa-miR-3605-5p	DDT	ENSG00000099977	1

hsa-miR-3613-5p	NCR3LG1	ENSG00000188211	1
hsa-miR-362-5p	CCNT1	ENSG00000129315	1
hsa-miR-362-5p	CHD1	ENSG00000153922	1
hsa-miR-362-5p	ZBTB10	ENSG00000205189	1
hsa-miR-365a-3p	KLHL15	ENSG00000174010	1
hsa-miR-365a-3p	REL	ENSG00000162924	1
hsa-miR-365a-5p	SPRED3	ENSG00000188766	1
hsa-miR-365a-5p	MASTL	ENSG00000120539	1
hsa-miR-365b-5p	MASTL	ENSG00000120539	1
hsa-miR-365b-5p	SPRED3	ENSG00000188766	1
hsa-miR-371a-3p	SYNM	ENSG00000182253	1
hsa-miR-371b-5p	CEBPB	ENSG00000172216	1
hsa-miR-371b-5p	ZNF285	ENSG00000267508	1
hsa-miR-371b-5p	SH3D19	ENSG00000109686	1
hsa-miR-371b-5p	CCDC121	ENSG00000176714	1
hsa-miR-371b-5p	MCM10	ENSG00000065328	1
hsa-miR-371b-5p	KLF2	ENSG00000127528	1
hsa-miR-374a-5p	TNFSF9	ENSG00000125657	1
hsa-miR-374a-5p	TNFAIP3	ENSG00000118503	1
hsa-miR-374a-5p	CEBPB	ENSG00000172216	1
hsa-miR-374a-5p	AVPR1A	ENSG00000166148	1
hsa-miR-374a-5p	DUSP8	ENSG00000184545	1
hsa-miR-374a-5p	YOD1	ENSG00000180667	1
hsa-miR-374a-5p	SPC25	ENSG00000152253	1
hsa-miR-374a-5p	KLHL15	ENSG00000174010	1
hsa-miR-376a-2-5p	ZNF805	ENSG00000204524	1
hsa-miR-376a-2-5p	ARL5B	ENSG00000165997	1
hsa-miR-3945	DDX47	ENSG00000213782	1
hsa-miR-432-5p	CENPA	ENSG00000115163	1
hsa-miR-4423-5p	DEPDC1	ENSG00000024526	1
hsa-miR-4433a-3p	MIXL1	ENSG00000185155	1
hsa-miR-4433a-3p	MRPL12	ENSG00000262814	1
hsa-miR-4433a-3p	NLRP6	ENSG00000174885	1
hsa-miR-4433a-3p	ZNF805	ENSG00000204524	1
hsa-miR-4433a-3p	SRGAP1	ENSG00000196935	1
hsa-miR-4433a-3p	CEBPD	ENSG00000221869	1
hsa-miR-4433a-5p	REL	ENSG00000162924	1
hsa-miR-4433a-5p	KLHL15	ENSG00000174010	1
hsa-miR-4482-5p	SDE2	ENSG00000143751	1
hsa-miR-4482-5p	REL	ENSG00000162924	1
hsa-miR-4485-5p	CNDP1	ENSG00000150656	1
hsa-miR-4485-5p	SH3D19	ENSG00000109686	1
hsa-miR-4494	PIM3	ENSG00000198355	1
hsa-miR-4494	GIMAP1	ENSG00000213203	1
hsa-miR-449a	MET	ENSG00000105976	1
hsa-miR-454-3p	DEPDC1	ENSG00000024526	1
hsa-miR-454-3p	PIGA	ENSG00000165195	1

hsa-miR-454-3p	ZNF805	ENSG00000204524	1
hsa-miR-454-3p	MASTL	ENSG00000120539	1
hsa-miR-454-3p	CCR6	ENSG00000112486	1
hsa-miR-454-3p	SIK1	ENSG00000142178	1
hsa-miR-454-3p	CFL2	ENSG00000165410	1
hsa-miR-454-3p	RLIM	ENSG00000131263	1
hsa-miR-454-3p	KLHL15	ENSG00000174010	1
hsa-miR-454-5p	KLF2	ENSG00000127528	1
hsa-miR-4632-3p	NHLRC4	ENSG00000257108	1
hsa-miR-4632-5p	ZMAT4	ENSG00000165061	1
hsa-miR-4632-5p	AURKA	ENSG00000087586	1
hsa-miR-4645-5p	CEBPD	ENSG00000221869	1
hsa-miR-4645-5p	CAV1	ENSG00000105974	1
hsa-miR-4645-5p	TMEM170B	ENSG00000205269	1
hsa-miR-4659b-5p	NR4A3	ENSG00000119508	1
hsa-miR-4717-5p	KLHL15	ENSG00000174010	1
hsa-miR-4746-5p	PIGA	ENSG00000165195	1
hsa-miR-4775	CTU1	ENSG00000142544	1
hsa-miR-4775	GRIK4	ENSG00000149403	1
hsa-miR-4775	SSPN	ENSG00000123096	1
hsa-miR-490-3p	ICOSLG	ENSG00000160223	1
hsa-miR-490-5p	FOS	ENSG00000170345	1
hsa-miR-500a-3p	KLHL15	ENSG00000174010	1
hsa-miR-500a-3p	ZBTB43	ENSG00000169155	1
hsa-miR-500a-3p	MIXL1	ENSG00000185155	1
hsa-miR-500a-5p	SLC1A2	ENSG00000110436	1
hsa-miR-500a-5p	REL	ENSG00000162924	1
hsa-miR-500a-5p	CCNT1	ENSG00000129315	1
hsa-miR-500a-5p	CKAP2L	ENSG00000169607	1
hsa-miR-501-5p	CHD1	ENSG00000153922	1
hsa-miR-501-5p	REL	ENSG00000162924	1
hsa-miR-501-5p	ZMAT4	ENSG00000165061	1
hsa-miR-501-5p	SLC1A2	ENSG00000110436	1
hsa-miR-502-5p	SSPN	ENSG00000123096	1
hsa-miR-502-5p	BHLHA15	ENSG00000180535	1
hsa-miR-506-3p	ZNF626	ENSG00000188171	1
hsa-miR-506-3p	SDE2	ENSG00000143751	1
hsa-miR-513a-5p	RNF152	ENSG00000176641	1
hsa-miR-513a-5p	ARL5B	ENSG00000165997	1
hsa-miR-513a-5p	DDT	ENSG00000099977	1
hsa-miR-513a-5p	BTG3	ENSG00000154640	1
hsa-miR-513a-5p	TXNDC5	ENSG00000239264	1
hsa-miR-513a-5p	NR1D2	ENSG00000174738	1
hsa-miR-513b-5p	NCR3LG1	ENSG00000188211	1
hsa-miR-513b-5p	KLHL15	ENSG00000174010	1
hsa-miR-513b-5p	GIMAP7	ENSG00000179144	1
hsa-miR-513b-5p	BTG3	ENSG00000154640	1

hsa-miR-539-5p	ZNF579	ENSG00000218891	1
hsa-miR-539-5p	BUB1	ENSG00000169679	1
hsa-miR-548a-5p	YOD1	ENSG00000180667	1
hsa-miR-548a-5p	FEM1C	ENSG00000145780	1
hsa-miR-548a-5p	ZBTB43	ENSG00000169155	1
hsa-miR-548a-5p	SIK1	ENSG00000142178	1
hsa-miR-548a-5p	SPC25	ENSG00000152253	1
hsa-miR-548ah-3p	ZBTB10	ENSG00000205189	1
hsa-miR-548ah-3p	YES1	ENSG00000176105	1
hsa-miR-548ah-3p	RHOB	ENSG00000143878	1
hsa-miR-548ah-3p	KIAA1324	ENSG00000116299	1
hsa-miR-548ah-3p	GRIK4	ENSG00000149403	1
hsa-miR-548ah-3p	FEM1C	ENSG00000145780	1
hsa-miR-548ah-3p	SPC25	ENSG00000152253	1
hsa-miR-548ah-3p	TNFAIP3	ENSG00000118503	1
hsa-miR-548ah-3p	ICOSLG	ENSG00000160223	1
hsa-miR-548ah-5p	BTG3	ENSG00000154640	1
hsa-miR-548ah-5p	FEM1C	ENSG00000145780	1
hsa-miR-548ah-5p	ZNF805	ENSG00000204524	1
hsa-miR-548ah-5p	CAV1	ENSG00000105974	1
hsa-miR-548ah-5p	HIF1A	ENSG00000100644	1
hsa-miR-548ah-5p	RLIM	ENSG00000131263	1
hsa-miR-548ah-5p	DGAT2	ENSG00000062282	1
hsa-miR-548ah-5p	KLHL15	ENSG00000174010	1
hsa-miR-548ah-5p	PRICKLE2	ENSG00000163637	1
hsa-miR-548ah-5p	SIK1	ENSG00000142178	1
hsa-miR-548ai	FTH1	ENSG00000167996	1
hsa-miR-548ba	FTH1	ENSG00000167996	1
hsa-miR-548s	KLF2	ENSG00000127528	1
hsa-miR-548s	SOX5	ENSG00000134532	1
hsa-miR-548s	ZNF285	ENSG00000267508	1
hsa-miR-548s	MRPL12	ENSG00000262814	1
hsa-miR-548s	PDF	ENSG00000258429	1
hsa-miR-548x-5p	E2F8	ENSG00000129173	1
hsa-miR-548x-5p	KCNJ2	ENSG00000123700	1
hsa-miR-548x-5p	CFL2	ENSG00000165410	1
hsa-miR-548x-5p	AVPR1A	ENSG00000166148	1
hsa-miR-548x-5p	ADRB1	ENSG00000043591	1
hsa-miR-548x-5p	ARL5B	ENSG00000165997	1
hsa-miR-551b-5p	BUB1	ENSG00000169679	1
hsa-miR-551b-5p	YOD1	ENSG00000180667	1
hsa-miR-551b-5p	FTH1	ENSG00000167996	1
hsa-miR-551b-5p	SLC7A5	ENSG00000103257	1
hsa-miR-551b-5p	ZBTB10	ENSG00000205189	1
hsa-miR-556-5p	CAV1	ENSG00000105974	1
hsa-miR-574-5p	SLC7A5	ENSG00000103257	1
hsa-miR-574-5p	BUB1	ENSG00000169679	1

hsa-miR-574-5p	TCTE1	ENSG00000146221	1
hsa-miR-574-5p	TREML2	ENSG00000112195	1
hsa-miR-574-5p	RNF152	ENSG00000176641	1
hsa-miR-574-5p	SSPN	ENSG00000123096	1
hsa-miR-574-5p	SOX5	ENSG00000134532	1
hsa-miR-574-5p	GIMAP1	ENSG00000213203	1
hsa-miR-574-5p	E2F8	ENSG00000129173	1
hsa-miR-574-5p	SMAD9	ENSG00000120693	1
hsa-miR-574-5p	NEXMIF	ENSG00000050030	1
hsa-miR-590-5p	KLHL15	ENSG00000174010	1
hsa-miR-616-3p	SIK1	ENSG00000142178	1
hsa-miR-616-5p	KLF2	ENSG00000127528	1
hsa-miR-616-5p	CEBPB	ENSG00000172216	1
hsa-miR-616-5p	MCM10	ENSG00000065328	1
hsa-miR-616-5p	ZNF285	ENSG00000267508	1
hsa-miR-616-5p	SH3D19	ENSG00000109686	1
hsa-miR-616-5p	CCDC121	ENSG00000176714	1
hsa-miR-624-3p	ARL5B	ENSG00000165997	1
hsa-miR-625-5p	JUND	ENSG00000130522	1
hsa-miR-625-5p	CITED4	ENSG00000179862	1
hsa-miR-625-5p	EFNA3	ENSG00000143590	1
hsa-miR-651-5p	HSPA12B	ENSG00000132622	1
hsa-miR-6513-5p	MANSC1	ENSG00000111261	1
hsa-miR-6513-5p	ZNF703	ENSG00000183779	1
hsa-miR-6513-5p	RHOF	ENSG00000139725	1
hsa-miR-6513-5p	PDF	ENSG00000258429	1
hsa-miR-6513-5p	SLC1A2	ENSG00000110436	1
hsa-miR-664b-3p	ZNF331	ENSG00000130844	1
hsa-miR-664b-3p	TENM1	ENSG00000009694	1
hsa-miR-664b-3p	SYNM	ENSG00000182253	1
hsa-miR-708-5p	SLC7A5	ENSG00000103257	1
hsa-miR-708-5p	BIRC5	ENSG00000089685	1
hsa-miR-877-5p	YOD1	ENSG00000180667	1
hsa-miR-877-5p	HSPA12B	ENSG00000132622	1
hsa-miR-877-5p	TMEM170B	ENSG00000205269	1
hsa-miR-877-5p	DDX47	ENSG00000213782	1
hsa-miR-885-5p	BUB1	ENSG00000169679	1
hsa-miR-885-5p	SYNM	ENSG00000182253	1
hsa-miR-9-5p	KCNJ2	ENSG00000123700	1
hsa-miR-9-5p	ZMAT4	ENSG00000165061	1
hsa-miR-9-5p	ARHGEF10	ENSG00000104728	1
hsa-miR-9-5p	P4HA2	ENSG00000072682	1
hsa-miR-9-5p	SDC1	ENSG00000115884	1
hsa-miR-92a-3p	RNF103	ENSG00000239305	1
hsa-miR-92a-3p	FKBPL	ENSG00000204315	1
hsa-miR-92a-3p	SERTAD2	ENSG00000179833	1
hsa-miR-92a-3p	KLF2	ENSG00000127528	1

hsa-miR-92a-3p	HES1	ENSG00000114315	1
hsa-miR-92a-3p	ZNF703	ENSG00000183779	1
hsa-miR-92a-3p	ADAMTS1	ENSG00000154734	1
hsa-miR-92a-3p	SMAD6	ENSG00000137834	1
hsa-miR-92a-3p	KLHL15	ENSG00000174010	1
hsa-miR-92a-3p	NLRP9	ENSG00000185792	1
hsa-miR-92a-3p	SIK1	ENSG00000142178	1
hsa-miR-92a-3p	GFPT2	ENSG00000131459	1
hsa-miR-92a-3p	TOM1L1	ENSG00000141198	1
hsa-miR-92a-3p	TMEM160	ENSG00000130748	1
hsa-miR-92a-3p	NEK2	ENSG00000117650	1
hsa-miR-92a-3p	REL	ENSG00000162924	1
hsa-miR-92a-3p	AURKA	ENSG00000087586	1
hsa-miR-92a-3p	KIF18B	ENSG00000186185	1
hsa-miR-92a-3p	TLR10	ENSG00000174123	1
hsa-miR-92a-3p	CHCHD10	ENSG00000250479	1
hsa-miR-92a-3p	MYBL2	ENSG00000101057	1
hsa-miR-92a-3p	CDC20	ENSG00000117399	1
hsa-miR-92a-3p	FASLG	ENSG00000117560	1
hsa-miR-92b-5p	SRGAP1	ENSG00000196935	1
hsa-miR-92b-5p	DDT	ENSG00000099977	1
hsa-miR-92b-5p	ARHGAP39	ENSG00000147799	1
hsa-miR-92b-5p	SLC7A5	ENSG00000103257	1
hsa-miR-93-5p	FEM1C	ENSG00000145780	1
hsa-miR-93-5p	ZNF805	ENSG00000204524	1
hsa-miR-93-5p	HIF1A	ENSG00000100644	1
hsa-miR-93-5p	PKMYT1	ENSG00000127564	1
hsa-miR-93-5p	ZBTB21	ENSG00000173276	1
hsa-miR-93-5p	SERTAD2	ENSG00000179833	1
hsa-miR-93-5p	MASTL	ENSG00000120539	1
hsa-miR-93-5p	KLHL15	ENSG00000174010	1
hsa-miR-93-5p	EREG	ENSG00000124882	1
hsa-miR-93-5p	YOD1	ENSG00000180667	1
hsa-miR-93-5p	BIRC5	ENSG00000089685	1
hsa-miR-93-5p	CXCL8	ENSG00000169429	1
hsa-miR-93-5p	BTG3	ENSG00000154640	1
hsa-miR-93-5p	RLIM	ENSG00000131263	1
hsa-miR-93-5p	SMAD6	ENSG00000137834	1
hsa-miR-93-5p	MIXL1	ENSG00000185155	1
hsa-miR-93-5p	CFL2	ENSG00000165410	1
hsa-miR-93-5p	SIK1	ENSG00000142178	1
hsa-miR-93-5p	C14orf119	ENSG00000179933	1
hsa-miR-93-5p	CAV1	ENSG00000105974	1
hsa-miR-93-5p	DUSP8	ENSG00000184545	1
hsa-miR-98-5p	ZBTB10	ENSG00000205189	1
hsa-miR-98-5p	YOD1	ENSG00000180667	1
hsa-miR-98-5p	SOCS1	ENSG00000185338	1

hsa-miR-98-5p	CEBPD	ENSG00000221869	1
hsa-miR-98-5p	ICOSLG	ENSG00000160223	1
hsa-miR-98-5p	SERTAD2	ENSG00000179833	1
hsa-miR-98-5p	DBF4	ENSG00000006634	1
hsa-miR-98-5p	NAT8L	ENSG00000185818	1
hsa-miR-98-5p	REL	ENSG00000162924	1
hsa-miR-98-5p	TNFSF9	ENSG00000125657	1
hsa-miR-98-5p	IL13	ENSG00000169194	1
hsa-miR-98-5p	SEMA3G	ENSG00000010319	1
hsa-miR-98-5p	FAM43A	ENSG00000185112	1
hsa-miR-98-5p	CHD1	ENSG00000153922	1
hsa-miR-98-5p	DUSP1	ENSG00000120129	1
hsa-miR-98-5p	TNFRSF9	ENSG00000049249	1
hsa-miR-98-5p	MRPL12	ENSG00000262814	1
hsa-miR-99a-5p	STEAP4	ENSG00000127954	1
hsa-miR-101-3p	NR4A3	ENSG00000119508	0.725
hsa-miR-101-3p	PRLR	ENSG00000113494	0.985
hsa-miR-101-3p	NR1D2	ENSG00000174738	0.87
hsa-miR-101-3p	REL	ENSG00000162924	0.793
hsa-miR-101-3p	TENM1	ENSG00000009694	0.97
hsa-miR-101-3p	ZBTB10	ENSG00000205189	0.995
hsa-miR-101-3p	YES1	ENSG00000176105	0.808
hsa-miR-101-3p	SCN3B	ENSG00000166257	0.831
hsa-miR-101-3p	FA2H	ENSG00000103089	0.717
hsa-miR-101-3p	SKOR1	ENSG00000188779	0.689
hsa-miR-101-3p	ZNF804A	ENSG00000170396	0.593
hsa-miR-101-3p	FEM1C	ENSG00000145780	0.964
hsa-miR-101-3p	FOS	ENSG00000170345	0.312
hsa-miR-101-3p	PDE4D	ENSG00000113448	0.792
hsa-miR-101-3p	ADRB1	ENSG00000043591	0.443
hsa-miR-101-3p	NAT8L	ENSG00000185818	0.834
hsa-miR-101-3p	NEXMIF	ENSG00000050030	0.891
hsa-miR-101-3p	DUSP1	ENSG00000120129	0.319
hsa-miR-101-3p	IL13	ENSG00000169194	0.661
hsa-miR-101-3p	ZBTB21	ENSG00000173276	0.715
hsa-miR-101-3p	KLF2	ENSG00000127528	0.607
hsa-miR-101-3p	ATP1B2	ENSG00000129244	0.962
hsa-miR-101-3p	RASGRF1	ENSG00000058335	0.916
hsa-miR-101-3p	CHD1	ENSG00000153922	0.976
hsa-miR-101-3p	SORCS3	ENSG00000156395	0.815
hsa-miR-101-3p	TMEM170B	ENSG00000205269	0.835
hsa-miR-101-3p	SRGAP1	ENSG00000196935	0.802
hsa-miR-101-3p	E2F8	ENSG00000129173	0.776
hsa-miR-101-3p	MET	ENSG00000105976	0.97
hsa-miR-101-3p	HEY2	ENSG00000135547	0.955
hsa-miR-101-3p	TNFSF11	ENSG00000120659	0.747
hsa-miR-101-3p	PPP1R9A	ENSG00000158528	0.995

hsa-miR-103a-3p	ISL2	ENSG00000159556	0.987
hsa-miR-103a-3p	NR1D2	ENSG00000174738	0.922
hsa-miR-103a-3p	SKOR1	ENSG00000188779	0.763
hsa-miR-103a-3p	RNF152	ENSG00000176641	0.874
hsa-miR-103a-3p	RORB	ENSG00000198963	0.787
hsa-miR-103a-3p	SRGAP1	ENSG00000196935	0.823
hsa-miR-103a-3p	NR4A3	ENSG00000119508	0.892
hsa-miR-103a-3p	EML5	ENSG00000165521	0.814
hsa-miR-103a-3p	PIK3R1	ENSG00000145675	0.833
hsa-miR-103a-3p	PDE4D	ENSG00000113448	0.82
hsa-miR-103a-3p	NAT8L	ENSG00000185818	0.827
hsa-miR-103a-3p	CHD1	ENSG00000153922	0.532
hsa-miR-103a-3p	TNFAIP3	ENSG00000118503	0.855
hsa-miR-103a-3p	PDK4	ENSG00000004799	0.652
hsa-miR-103a-3p	ZBTB10	ENSG00000205189	0.628
hsa-miR-103a-3p	ATP1B2	ENSG00000129244	0.925
hsa-miR-103a-3p	TMEM250	ENSG00000238227	0.893
hsa-miR-103a-3p	CNDP1	ENSG00000150656	0.759
hsa-miR-10a-5p	NR4A3	ENSG00000119508	0.767
hsa-miR-10a-5p	SH3D19	ENSG00000109686	0.765
hsa-miR-10a-5p	ZBTB43	ENSG00000169155	0.956
hsa-miR-10a-5p	RORB	ENSG00000198963	0.672
hsa-miR-10a-5p	SDC1	ENSG00000115884	0.632
hsa-miR-10a-5p	TMEM170B	ENSG00000205269	0.863
hsa-miR-122-5p	ZNF703	ENSG00000183779	1
hsa-miR-122-5p	PDK4	ENSG00000004799	0.592
hsa-miR-124-3p	GOLGA8M	ENSG00000188626	0.645
hsa-miR-124-3p	JAM2	ENSG00000154721	0.568
hsa-miR-124-3p	PIM3	ENSG00000198355	0.598
hsa-miR-124-3p	GFPT2	ENSG00000131459	0.693
hsa-miR-124-3p	TENM1	ENSG00000009694	0.853
hsa-miR-124-3p	PAQR8	ENSG00000170915	0.347
hsa-miR-124-3p	TXNDC5	ENSG00000239264	0.749
hsa-miR-124-3p	EML5	ENSG00000165521	0.687
hsa-miR-124-3p	NR1D2	ENSG00000174738	0.929
hsa-miR-124-3p	SIK1	ENSG00000142178	0.838
hsa-miR-124-3p	CFL2	ENSG00000165410	0.628
hsa-miR-124-3p	P4HA2	ENSG00000072682	0.571
hsa-miR-124-3p	YOD1	ENSG00000180667	0.445
hsa-miR-124-3p	KCNJ2	ENSG00000123700	0.65
hsa-miR-124-3p	SERTAD2	ENSG00000179833	0.657
hsa-miR-124-3p	TMEM170B	ENSG00000205269	0.873
hsa-miR-124-3p	DEPDC1	ENSG00000024526	0.683
hsa-miR-124-3p	NR4A3	ENSG00000119508	0.896
hsa-miR-124-3p	PRLR	ENSG00000113494	0.873
hsa-miR-124-3p	PPP1R9A	ENSG00000158528	0.845
hsa-miR-124-3p	PRICKLE2	ENSG00000163637	0.98

hsa-miR-124-3p	NAT8L	ENSG00000185818	0.449
hsa-miR-124-3p	CHD1	ENSG00000153922	0.472
hsa-miR-124-3p	CAV1	ENSG00000105974	0.767
hsa-miR-124-3p	CACNA1E	ENSG00000198216	0.852
hsa-miR-124-3p	AHRR	ENSG00000063438	0.766
hsa-miR-124-3p	RLIM	ENSG00000131263	0.545
hsa-miR-124-3p	GRASP	ENSG00000161835	0.721
hsa-miR-124-3p	HIC1	ENSG00000177374	0.643
hsa-miR-124-3p	DGAT2	ENSG00000062282	0.938
hsa-miR-124-3p	LRFN1	ENSG00000128011	0.583
hsa-miR-124-3p	ARHGAP39	ENSG00000147799	0.771
hsa-miR-124-3p	RHBDF1	ENSG00000007384	0.622
hsa-miR-124-3p	SSPN	ENSG00000123096	0.994
hsa-miR-124-3p	SRGAP1	ENSG00000196935	0.717
hsa-miR-124-3p	MOCS1	ENSG00000124615	0.698
hsa-miR-124-3p	PDE4D	ENSG00000113448	0.905
hsa-miR-124-3p	MCM10	ENSG00000065328	0.841
hsa-miR-124-3p	TNFSF11	ENSG00000120659	0.68
hsa-miR-124-3p	ARL5B	ENSG00000165997	0.818
hsa-miR-124-3p	PGAP1	ENSG00000197121	0.907
hsa-miR-124-3p	ROR2	ENSG00000169071	0.684
hsa-miR-1249-3p	SKOR1	ENSG00000188779	0.457
hsa-miR-125b-5p	YES1	ENSG00000176105	0.85
hsa-miR-125b-5p	TNFAIP3	ENSG00000118503	0.515
hsa-miR-125b-5p	KIF18B	ENSG00000186185	0.793
hsa-miR-125b-5p	RORB	ENSG00000198963	0.772
hsa-miR-125b-5p	YOD1	ENSG00000180667	0.833
hsa-miR-125b-5p	RASGRF1	ENSG00000058335	0.716
hsa-miR-125b-5p	TMEM170B	ENSG00000205269	0.874
hsa-miR-125b-5p	SCARF2	ENSG00000244486	0.98
hsa-miR-125b-5p	MOCS1	ENSG00000124615	0.864
hsa-miR-125b-5p	NIPAL4	ENSG00000172548	0.609
hsa-miR-125b-5p	C4orf19	ENSG00000154274	0.765
hsa-miR-125b-5p	BORCS6	ENSG00000196544	0.702
hsa-miR-125b-5p	CCDC85C	ENSG00000205476	0.869
hsa-miR-129-5p	COL1A1	ENSG00000108821	0.98
hsa-miR-129-5p	CDKN1C	ENSG00000129757	0.724
hsa-miR-129-5p	TMEM250	ENSG00000238227	0.882
hsa-miR-129-5p	NR4A2	ENSG00000153234	0.747
hsa-miR-129-5p	RBFOX3	ENSG00000167281	0.829
hsa-miR-129-5p	KLHL15	ENSG00000174010	0.953
hsa-miR-129-5p	TXNDC5	ENSG00000239264	1
hsa-miR-129-5p	ZMAT4	ENSG00000165061	0.98
hsa-miR-129-5p	TIPARP	ENSG00000163659	0.821
hsa-miR-129-5p	ZBTB10	ENSG00000205189	0.96
hsa-miR-129-5p	ZNF703	ENSG00000183779	0.968
hsa-miR-129-5p	OTX1	ENSG00000115507	0.82

hsa-miR-129-5p	SIAH1	ENSG00000196470	0.969
hsa-miR-129-5p	P4HA2	ENSG00000072682	0.99
hsa-miR-144-3p	MET	ENSG00000105976	0.99
hsa-miR-144-3p	E2F8	ENSG00000129173	0.698
hsa-miR-144-3p	RORB	ENSG00000198963	0.812
hsa-miR-144-3p	RASGRF1	ENSG00000058335	0.958
hsa-miR-144-3p	FOS	ENSG00000170345	0.855
hsa-miR-144-3p	SORCS3	ENSG00000156395	0.716
hsa-miR-144-3p	ZBTB21	ENSG00000173276	0.873
hsa-miR-144-3p	ADRB1	ENSG00000043591	0.884
hsa-miR-144-3p	DUSP1	ENSG00000120129	0.89
hsa-miR-144-3p	NR1D2	ENSG00000174738	0.987
hsa-miR-144-3p	TNFSF11	ENSG00000120659	0.552
hsa-miR-144-3p	PDE4D	ENSG00000113448	0.937
hsa-miR-144-3p	HEY2	ENSG00000135547	0.982
hsa-miR-144-3p	TENM1	ENSG00000009694	0.97
hsa-miR-144-3p	SRGAP1	ENSG00000196935	0.992
hsa-miR-144-3p	ATP1B2	ENSG00000129244	0.986
hsa-miR-144-3p	CFL2	ENSG00000165410	0.71
hsa-miR-153-3p	EFNA3	ENSG00000143590	0.631
hsa-miR-153-3p	PDE4D	ENSG00000113448	0.882
hsa-miR-153-3p	ZNF703	ENSG00000183779	0.892
hsa-miR-153-3p	CFL2	ENSG00000165410	0.562
hsa-miR-153-3p	NRARP	ENSG00000198435	0.925
hsa-miR-153-3p	PIGA	ENSG00000165195	0.811
hsa-miR-153-3p	FEM1C	ENSG00000145780	0.499
hsa-miR-153-3p	PIK3R1	ENSG00000145675	0.791
hsa-miR-153-3p	ZBTB10	ENSG00000205189	0.868
hsa-miR-153-3p	SYN1	ENSG00000008056	0.786
hsa-miR-153-3p	CHD1	ENSG00000153922	0.976
hsa-miR-153-3p	SERTAD2	ENSG00000179833	0.497
hsa-miR-153-3p	GFPT2	ENSG00000131459	0.462
hsa-miR-153-3p	ZBTB43	ENSG00000169155	0.802
hsa-miR-153-3p	RNF152	ENSG00000176641	0.956
hsa-miR-153-3p	CCNT1	ENSG00000129315	0.875
hsa-miR-153-3p	HEY2	ENSG00000135547	0.865
hsa-miR-153-3p	TMEM170B	ENSG00000205269	0.936
hsa-miR-153-3p	SCML1	ENSG00000047634	0.7
hsa-miR-153-3p	GRIK4	ENSG00000149403	0.678
hsa-miR-153-3p	ZBTB21	ENSG00000173276	0.742
hsa-miR-15a-5p	CCDC85C	ENSG00000205476	0.773
hsa-miR-15a-5p	CDC42EP2	ENSG00000149798	0.371
hsa-miR-15a-5p	RNF152	ENSG00000176641	0.876
hsa-miR-15a-5p	BORCS6	ENSG00000196544	0.357
hsa-miR-15a-5p	NAT8L	ENSG00000185818	0.819
hsa-miR-15a-5p	OTX1	ENSG00000115507	0.667
hsa-miR-15a-5p	PIM3	ENSG00000198355	0.868

hsa-miR-15a-5p	SLC23A3	ENSG00000213901	0.824
hsa-miR-15a-5p	CNIH2	ENSG00000174871	0.719
hsa-miR-15a-5p	NRARP	ENSG00000198435	0.973
hsa-miR-15a-5p	ARL5B	ENSG00000165997	0.816
hsa-miR-15a-5p	BTN1A1	ENSG00000124557	0.447
hsa-miR-15a-5p	GRIN1	ENSG00000176884	0.98
hsa-miR-15a-5p	AOC1	ENSG00000002726	0.805
hsa-miR-15a-5p	ZBTB10	ENSG00000205189	0.786
hsa-miR-15a-5p	TMEM255A	ENSG00000125355	0.648
hsa-miR-15a-5p	SOX5	ENSG00000134532	0.739
hsa-miR-15a-5p	ZBTB43	ENSG00000169155	0.792
hsa-miR-15a-5p	TMEM170B	ENSG00000205269	0.834
hsa-miR-15a-5p	LRRN3	ENSG00000173114	0.764
hsa-miR-15a-5p	YOD1	ENSG00000180667	0.957
hsa-miR-15a-5p	RLIM	ENSG00000131263	0.945
hsa-miR-15a-5p	RASGEF1B	ENSG00000138670	0.14
hsa-miR-15a-5p	SIAH1	ENSG00000196470	0.709
hsa-miR-15a-5p	MOCS1	ENSG00000124615	0.952
hsa-miR-15a-5p	TMEM250	ENSG00000238227	0.878
hsa-miR-15a-5p	RGPD6	ENSG00000183054	0.755
hsa-miR-15a-5p	SKOR1	ENSG00000188779	0.82
hsa-miR-15a-5p	RORB	ENSG00000198963	0.819
hsa-miR-15a-5p	CACNA1E	ENSG00000198216	0.97
hsa-miR-15a-5p	KLHL15	ENSG00000174010	0.811
hsa-miR-15a-5p	CD180	ENSG00000134061	1
hsa-miR-15a-5p	KCNJ2	ENSG00000123700	0.501
hsa-miR-15a-5p	SIK1	ENSG00000142178	0.602
hsa-miR-15a-5p	SRGAP1	ENSG00000196935	0.987
hsa-miR-15a-5p	TCTE1	ENSG00000146221	0.765
hsa-miR-15a-5p	PIK3R1	ENSG00000145675	0.775
hsa-miR-15a-5p	PDK4	ENSG00000004799	0.434
hsa-miR-15b-5p	SIK1	ENSG00000142178	0.613
hsa-miR-15b-5p	SLC23A3	ENSG00000213901	0.813
hsa-miR-15b-5p	PIM3	ENSG00000198355	0.868
hsa-miR-15b-5p	CDC42EP2	ENSG00000149798	0.36
hsa-miR-15b-5p	OTX1	ENSG00000115507	0.667
hsa-miR-15b-5p	CACNA1E	ENSG00000198216	0.97
hsa-miR-15b-5p	MOCS1	ENSG00000124615	0.958
hsa-miR-15b-5p	NRARP	ENSG00000198435	0.972
hsa-miR-15b-5p	RGPD6	ENSG00000183054	0.755
hsa-miR-15b-5p	PIK3R1	ENSG00000145675	0.775
hsa-miR-15b-5p	SOX5	ENSG00000134532	0.739
hsa-miR-15b-5p	SRGAP1	ENSG00000196935	0.987
hsa-miR-15b-5p	KLHL15	ENSG00000174010	0.811
hsa-miR-15b-5p	CCDC85C	ENSG00000205476	0.782
hsa-miR-15b-5p	SIAH1	ENSG00000196470	0.709
hsa-miR-15b-5p	CD180	ENSG00000134061	1

hsa-miR-15b-5p	TMEM250	ENSG00000238227	0.878
hsa-miR-15b-5p	RORB	ENSG00000198963	0.835
hsa-miR-15b-5p	TCTE1	ENSG00000146221	0.765
hsa-miR-15b-5p	LRRN3	ENSG00000173114	0.764
hsa-miR-15b-5p	TMEM170B	ENSG00000205269	0.834
hsa-miR-15b-5p	SKOR1	ENSG00000188779	0.82
hsa-miR-15b-5p	RNF152	ENSG00000176641	0.885
hsa-miR-15b-5p	GRIN1	ENSG00000176884	0.98
hsa-miR-15b-5p	KCNJ2	ENSG00000123700	0.489
hsa-miR-15b-5p	RLIM	ENSG00000131263	0.956
hsa-miR-15b-5p	YOD1	ENSG00000180667	0.957
hsa-miR-15b-5p	ARL5B	ENSG00000165997	0.816
hsa-miR-15b-5p	ZBTB43	ENSG00000169155	0.781
hsa-miR-15b-5p	CNIH2	ENSG00000174871	0.719
hsa-miR-15b-5p	RASGEF1B	ENSG00000138670	0.148
hsa-miR-15b-5p	BTN1A1	ENSG00000124557	0.455
hsa-miR-15b-5p	PDK4	ENSG00000004799	0.456
hsa-miR-15b-5p	BORCS6	ENSG00000196544	0.39
hsa-miR-15b-5p	ZBTB10	ENSG00000205189	0.786
hsa-miR-15b-5p	TMEM255A	ENSG00000125355	0.659
hsa-miR-15b-5p	AOC1	ENSG00000002726	0.794
hsa-miR-15b-5p	NAT8L	ENSG00000185818	0.819
hsa-miR-17-5p	SERTAD2	ENSG00000179833	0.825
hsa-miR-17-5p	OSM	ENSG00000099985	0.604
hsa-miR-17-5p	BTG3	ENSG00000154640	0.332
hsa-miR-17-5p	ZBTB43	ENSG00000169155	0.799
hsa-miR-17-5p	TIPARP	ENSG00000163659	0.863
hsa-miR-17-5p	PER1	ENSG00000179094	0.964
hsa-miR-17-5p	SMAD6	ENSG00000137834	0.969
hsa-miR-17-5p	TNFSF11	ENSG00000120659	0.724
hsa-miR-17-5p	SRGAP1	ENSG00000196935	0.894
hsa-miR-17-5p	MASTL	ENSG00000120539	0.468
hsa-miR-17-5p	SLC1A2	ENSG00000110436	0.98
hsa-miR-17-5p	SIK1	ENSG00000142178	0.72
hsa-miR-17-5p	FEM1C	ENSG00000145780	0.647
hsa-miR-17-5p	DUSP8	ENSG00000184545	0.895
hsa-miR-17-5p	ARHGEF10	ENSG00000104728	0.759
hsa-miR-17-5p	YOD1	ENSG00000180667	0.85
hsa-miR-17-5p	KLHL15	ENSG00000174010	0.767
hsa-miR-17-5p	YES1	ENSG00000176105	0.827
hsa-miR-17-5p	HIF1A	ENSG00000100644	0.796
hsa-miR-17-5p	PIK3R1	ENSG00000145675	0.981
hsa-miR-17-5p	PANX2	ENSG00000073150	0.816
hsa-miR-17-5p	TENM1	ENSG00000009694	0.874
hsa-miR-17-5p	SYNM	ENSG00000182253	0.924
hsa-miR-17-5p	RLIM	ENSG00000131263	0.668
hsa-miR-17-5p	AHRR	ENSG00000063438	0.983

hsa-miR-17-5p	RORB	ENSG00000198963	0.917
hsa-miR-17-5p	CHST7	ENSG00000147119	0.999
hsa-miR-17-5p	ZNF703	ENSG00000183779	1
hsa-miR-17-5p	PFKFB3	ENSG00000170525	0.822
hsa-miR-17-5p	ZBTB21	ENSG00000173276	0.91
hsa-miR-17-5p	SKOR1	ENSG00000188779	0.695
hsa-miR-17-5p	NR4A2	ENSG00000153234	0.692
hsa-miR-17-5p	CXCL8	ENSG00000169429	0.572
hsa-miR-17-5p	CFL2	ENSG00000165410	0.559
hsa-miR-17-5p	NR4A3	ENSG00000119508	0.58
hsa-miR-17-5p	EREG	ENSG00000124882	0.671
hsa-miR-17-5p	ZNF805	ENSG00000204524	0.935
hsa-miR-187-3p	LRFN1	ENSG00000128011	0.556
hsa-miR-188-5p	ISL2	ENSG00000159556	1
hsa-miR-188-5p	SPRED3	ENSG00000188766	0.974
hsa-miR-188-5p	ARL5B	ENSG00000165997	0.827
hsa-miR-190a-5p	CCSER1	ENSG00000184305	0.98
hsa-miR-190a-5p	RLIM	ENSG00000131263	0.925
hsa-miR-190a-5p	TENM1	ENSG00000009694	0.958
hsa-miR-190b	TENM1	ENSG00000009694	0.969
hsa-miR-190b	RLIM	ENSG00000131263	0.892
hsa-miR-190b	CCSER1	ENSG00000184305	0.939
hsa-miR-193a-3p	SOX5	ENSG00000134532	0.777
hsa-miR-193a-3p	KLHL15	ENSG00000174010	0.926
hsa-miR-193a-3p	SIAH1	ENSG00000196470	0.601
hsa-miR-193a-3p	SYN1	ENSG00000008056	0.852
hsa-miR-193a-3p	KCNJ2	ENSG00000123700	0.529
hsa-miR-193a-3p	ARHGAP39	ENSG00000147799	0.812
hsa-miR-193a-5p	COL1A1	ENSG00000108821	0.824
hsa-miR-193a-5p	SMAD9	ENSG00000120693	0.928
hsa-miR-193a-5p	SPRED3	ENSG00000188766	0.776
hsa-miR-193a-5p	GOLGA8M	ENSG00000188626	0.82
hsa-miR-193b-3p	KCNJ2	ENSG00000123700	0.521
hsa-miR-193b-3p	ARHGAP39	ENSG00000147799	0.812
hsa-miR-193b-3p	SYN1	ENSG00000008056	0.852
hsa-miR-193b-3p	SIAH1	ENSG00000196470	0.601
hsa-miR-193b-3p	KLHL15	ENSG00000174010	0.926
hsa-miR-193b-3p	SOX5	ENSG00000134532	0.789
hsa-miR-196a-5p	PRLR	ENSG00000113494	0.925
hsa-miR-196a-5p	SMAD6	ENSG00000137834	0.668
hsa-miR-196a-5p	FLRT1	ENSG00000126500	0.712
hsa-miR-196a-5p	KLHL15	ENSG00000174010	0.979
hsa-miR-196a-5p	OTX1	ENSG00000115507	0.776
hsa-miR-196a-5p	KCNJ2	ENSG00000123700	0.529
hsa-miR-196a-5p	B3GNT7	ENSG00000156966	0.831
hsa-miR-196a-5p	YOD1	ENSG00000180667	0.718
hsa-miR-196a-5p	ZBTB10	ENSG00000205189	0.922

hsa-miR-196a-5p	ABCB9	ENSG00000150967	0.639
hsa-miR-196a-5p	COL1A1	ENSG00000108821	0.777
hsa-miR-199a-5p	PPP1R9A	ENSG00000158528	0.757
hsa-miR-199a-5p	RLIM	ENSG00000131263	0.648
hsa-miR-199a-5p	ZNF579	ENSG00000218891	0.514
hsa-miR-199a-5p	RORB	ENSG00000198963	0.849
hsa-miR-199a-5p	HIF1A	ENSG00000100644	0.758
hsa-miR-199a-5p	ARHGAP29	ENSG00000137962	0.813
hsa-miR-199a-5p	CDKN1C	ENSG00000129757	0.73
hsa-miR-199a-5p	PDE4D	ENSG00000113448	0.708
hsa-miR-199a-5p	SPRED3	ENSG00000188766	0.918
hsa-miR-199a-5p	ZNF547	ENSG00000152433	-0.413
hsa-miR-199a-5p	SORCS3	ENSG00000156395	0.615
hsa-miR-199a-5p	CAV1	ENSG00000105974	0.819
hsa-miR-199a-5p	ZNF703	ENSG00000183779	1
hsa-miR-199a-5p	OTX1	ENSG00000115507	0.698
hsa-miR-199b-5p	ZNF703	ENSG00000183779	1
hsa-miR-199b-5p	ZNF547	ENSG00000152433	-0.412
hsa-miR-199b-5p	RORB	ENSG00000198963	0.838
hsa-miR-199b-5p	HIF1A	ENSG00000100644	0.769
hsa-miR-199b-5p	PDE4D	ENSG00000113448	0.708
hsa-miR-199b-5p	RLIM	ENSG00000131263	0.648
hsa-miR-199b-5p	ARHGAP29	ENSG00000137962	0.813
hsa-miR-199b-5p	CDKN1C	ENSG00000129757	0.73
hsa-miR-199b-5p	PPP1R9A	ENSG00000158528	0.757
hsa-miR-199b-5p	OTX1	ENSG00000115507	0.706
hsa-miR-199b-5p	SPRED3	ENSG00000188766	0.939
hsa-miR-199b-5p	SORCS3	ENSG00000156395	0.623
hsa-miR-199b-5p	ZNF579	ENSG00000218891	0.514
hsa-miR-199b-5p	CAV1	ENSG00000105974	0.819
hsa-miR-204-5p	CCSER1	ENSG00000184305	0.828
hsa-miR-204-5p	ARHGAP29	ENSG00000137962	0.759
hsa-miR-204-5p	PPP1R9A	ENSG00000158528	0.977
hsa-miR-204-5p	NEXMIF	ENSG00000050030	0.888
hsa-miR-204-5p	NR4A2	ENSG00000153234	0.689
hsa-miR-204-5p	ISL2	ENSG00000159556	0.978
hsa-miR-204-5p	TMEM255A	ENSG00000125355	0.86
hsa-miR-204-5p	SMAD6	ENSG00000137834	1
hsa-miR-204-5p	RORB	ENSG00000198963	0.98
hsa-miR-204-5p	PDF	ENSG00000258429	1
hsa-miR-204-5p	TENM1	ENSG00000009694	0.92
hsa-miR-204-5p	ARL5B	ENSG00000165997	0.926
hsa-miR-204-5p	NRARP	ENSG00000198435	0.97
hsa-miR-204-5p	ZBTB21	ENSG00000173276	0.898
hsa-miR-204-5p	CD180	ENSG00000134061	1
hsa-miR-206	FZD7	ENSG00000155760	0.775
hsa-miR-206	KLHL15	ENSG00000174010	0.849

hsa-miR-206	ZBTB21	ENSG00000173276	0.846
hsa-miR-206	KCNJ2	ENSG00000123700	0.767
hsa-miR-206	ISL2	ENSG00000159556	0.893
hsa-miR-206	YES1	ENSG00000176105	0.864
hsa-miR-206	NETO1	ENSG00000166342	0.779
hsa-miR-206	SRXN1	ENSG00000271303	0.914
hsa-miR-206	NEXMIF	ENSG00000050030	0.897
hsa-miR-206	SOX5	ENSG00000134532	0.963
hsa-miR-206	PPP1R9A	ENSG00000158528	0.926
hsa-miR-206	MET	ENSG00000105976	0.73
hsa-miR-206	JUND	ENSG00000130522	0.722
hsa-miR-206	NR4A2	ENSG00000153234	0.76
hsa-miR-206	NR4A3	ENSG00000119508	0.834
hsa-miR-20a-5p	EREG	ENSG00000124882	0.657
hsa-miR-20a-5p	CHST7	ENSG00000147119	0.999
hsa-miR-20a-5p	TENM1	ENSG00000009694	0.86
hsa-miR-20a-5p	AHRR	ENSG00000063438	0.983
hsa-miR-20a-5p	PFKFB3	ENSG00000170525	0.808
hsa-miR-20a-5p	CXCL8	ENSG00000169429	0.593
hsa-miR-20a-5p	NR4A3	ENSG00000119508	0.563
hsa-miR-20a-5p	SYNM	ENSG00000182253	0.915
hsa-miR-20a-5p	PANX2	ENSG00000073150	0.802
hsa-miR-20a-5p	YES1	ENSG00000176105	0.814
hsa-miR-20a-5p	BTG3	ENSG00000154640	0.329
hsa-miR-20a-5p	ZNF805	ENSG00000204524	0.949
hsa-miR-20a-5p	PIK3R1	ENSG00000145675	0.976
hsa-miR-20a-5p	YOD1	ENSG00000180667	0.844
hsa-miR-20a-5p	ZNF703	ENSG00000183779	1
hsa-miR-20a-5p	SERTAD2	ENSG00000179833	0.828
hsa-miR-20a-5p	ARHGEF10	ENSG00000104728	0.778
hsa-miR-20a-5p	SKOR1	ENSG00000188779	0.73
hsa-miR-20a-5p	PER1	ENSG00000179094	0.96
hsa-miR-20a-5p	SLC1A2	ENSG00000110436	0.98
hsa-miR-20a-5p	ZBTB21	ENSG00000173276	0.923
hsa-miR-20a-5p	SMAD6	ENSG00000137834	0.967
hsa-miR-20a-5p	KLHL15	ENSG00000174010	0.753
hsa-miR-20a-5p	NR4A2	ENSG00000153234	0.69
hsa-miR-20a-5p	ZBTB43	ENSG00000169155	0.752
hsa-miR-20a-5p	HIF1A	ENSG00000100644	0.761
hsa-miR-20a-5p	SIK1	ENSG00000142178	0.741
hsa-miR-20a-5p	MASTL	ENSG00000120539	0.481
hsa-miR-20a-5p	RLIM	ENSG00000131263	0.632
hsa-miR-20a-5p	FEM1C	ENSG00000145780	0.667
hsa-miR-20a-5p	CFL2	ENSG00000165410	0.536
hsa-miR-20a-5p	DUSP8	ENSG00000184545	0.883
hsa-miR-20a-5p	SRGAP1	ENSG00000196935	0.885
hsa-miR-20a-5p	TNFSF11	ENSG00000120659	0.721

hsa-miR-20a-5p	RORB	ENSG00000198963	0.925
hsa-miR-20a-5p	TIPARP	ENSG00000163659	0.849
hsa-miR-20a-5p	OSM	ENSG00000099985	0.633
hsa-miR-21-5p	PIK3R1	ENSG00000145675	0.778
hsa-miR-21-5p	SOX5	ENSG00000134532	0.591
hsa-miR-21-5p	KLHL15	ENSG00000174010	0.947
hsa-miR-21-5p	ESM1	ENSG00000164283	0.826
hsa-miR-21-5p	RHOB	ENSG00000143878	0.877
hsa-miR-21-5p	YOD1	ENSG00000180667	0.583
hsa-miR-21-5p	FASLG	ENSG00000117560	0.36
hsa-miR-21-5p	DUSP8	ENSG00000184545	0.656
hsa-miR-218-5p	GRM3	ENSG00000198822	0.729
hsa-miR-218-5p	NEXMIF	ENSG00000050030	0.856
hsa-miR-218-5p	ARL5B	ENSG00000165997	0.917
hsa-miR-218-5p	RORB	ENSG00000198963	0.773
hsa-miR-218-5p	NR1D2	ENSG00000174738	0.929
hsa-miR-218-5p	ADRB1	ENSG00000043591	0.859
hsa-miR-218-5p	NAT8L	ENSG00000185818	0.863
hsa-miR-218-5p	ZBTB10	ENSG00000205189	0.939
hsa-miR-218-5p	TXNDC5	ENSG00000239264	0.784
hsa-miR-218-5p	COL1A1	ENSG00000108821	0.742
hsa-miR-218-5p	RNF152	ENSG00000176641	0.885
hsa-miR-218-5p	RLIM	ENSG00000131263	0.782
hsa-miR-218-5p	PIK3R1	ENSG00000145675	0.779
hsa-miR-218-5p	SERTAD2	ENSG00000179833	0.625
hsa-miR-218-5p	SH3D19	ENSG00000109686	0.761
hsa-miR-218-5p	SOX5	ENSG00000134532	0.793
hsa-miR-218-5p	RNF103	ENSG00000239305	0.461
hsa-miR-218-5p	PRLR	ENSG00000113494	0.906
hsa-miR-218-5p	ZMAT4	ENSG00000165061	0.727
hsa-miR-218-5p	RGS20	ENSG00000147509	0.886
hsa-miR-219a-5p	DIAPH3	ENSG00000139734	0.874
hsa-miR-219a-5p	KCNJ2	ENSG00000123700	0.713
hsa-miR-219a-5p	CFL2	ENSG00000165410	0.991
hsa-miR-219a-5p	C4orf19	ENSG00000154274	0.804
hsa-miR-219a-5p	CACNA1E	ENSG00000198216	0.872
hsa-miR-219a-5p	SOX5	ENSG00000134532	0.822
hsa-miR-219a-5p	RNF152	ENSG00000176641	0.887
hsa-miR-219a-5p	RORB	ENSG00000198963	0.115
hsa-miR-219a-5p	PDE4D	ENSG00000113448	0.807
hsa-miR-24-3p	KCNJ2	ENSG00000123700	0.771
hsa-miR-24-3p	CACNA1E	ENSG00000198216	0.97
hsa-miR-24-3p	YOD1	ENSG00000180667	0.73
hsa-miR-24-3p	COMTD1	ENSG00000165644	0.994
hsa-miR-24-3p	SCML1	ENSG00000047634	0.439
hsa-miR-24-3p	PER1	ENSG00000179094	0.781
hsa-miR-24-3p	CHI3L1	ENSG00000133048	0.786

hsa-miR-24-3p	KLHL15	ENSG00000174010	0.96
hsa-miR-24-3p	MOCS1	ENSG00000124615	0.97
hsa-miR-24-3p	DUSP8	ENSG00000184545	0.791
hsa-miR-24-3p	ATP1B2	ENSG00000129244	0.601
hsa-miR-24-3p	HIC1	ENSG00000177374	0.889
hsa-miR-24-3p	FASLG	ENSG00000117560	0.673
hsa-miR-24-3p	CCDC85C	ENSG00000205476	0.893
hsa-miR-24-3p	SLC23A3	ENSG00000213901	0.777
hsa-miR-24-3p	ABCB9	ENSG00000150967	0.718
hsa-miR-24-3p	RHBDL1	ENSG00000103269	0.589
hsa-miR-24-3p	CDKN1C	ENSG00000129757	0.762
hsa-miR-24-3p	TNFSF9	ENSG00000125657	0.587
hsa-miR-24-3p	CITED4	ENSG00000179862	0.418
hsa-miR-24-3p	FCRLB	ENSG00000162746	0.865
hsa-miR-24-3p	TENM1	ENSG00000009694	0.901
hsa-miR-301a-3p	NEXMIF	ENSG00000050030	0.743
hsa-miR-301a-3p	TMEM170B	ENSG00000205269	0.651
hsa-miR-301a-3p	PPP1R9A	ENSG00000158528	0.936
hsa-miR-301a-3p	DEPDC1	ENSG00000024526	0.786
hsa-miR-301a-3p	PDE4D	ENSG00000113448	0.824
hsa-miR-301a-3p	CFL2	ENSG00000165410	0.905
hsa-miR-301a-3p	DIAPH3	ENSG00000139734	0.845
hsa-miR-301a-3p	COL9A3	ENSG00000092758	0.986
hsa-miR-301a-3p	KLHL15	ENSG00000174010	0.884
hsa-miR-301a-3p	TENM1	ENSG00000009694	0.97
hsa-miR-301a-3p	RLIM	ENSG00000131263	0.807
hsa-miR-301a-3p	RORB	ENSG00000198963	0.94
hsa-miR-301a-3p	PFKFB3	ENSG00000170525	0.904
hsa-miR-301a-3p	TMEM250	ENSG00000238227	0.502
hsa-miR-301a-3p	PRICKLE2	ENSG00000163637	0.85
hsa-miR-301a-3p	EREG	ENSG00000124882	0.406
hsa-miR-301a-3p	SIK1	ENSG00000142178	0.817
hsa-miR-301a-3p	NRARP	ENSG00000198435	0.866
hsa-miR-301a-3p	FAM43A	ENSG00000185112	0.694
hsa-miR-301a-3p	PIGA	ENSG00000165195	0.657
hsa-miR-301a-3p	MET	ENSG00000105976	0.596
hsa-miR-301a-3p	SYNM	ENSG00000182253	0.878
hsa-miR-301a-3p	SH3D19	ENSG00000109686	0.394
hsa-miR-301a-3p	SOX5	ENSG00000134532	0.937
hsa-miR-3064-5p	CCNT1	ENSG00000129315	0.957
hsa-miR-3064-5p	RORB	ENSG00000198963	0.905
hsa-miR-3064-5p	CHST7	ENSG00000147119	0.993
hsa-miR-3064-5p	CHD1	ENSG00000153922	0.985
hsa-miR-3064-5p	TCTE1	ENSG00000146221	0.934
hsa-miR-3064-5p	MOCS1	ENSG00000124615	0.917
hsa-miR-30c-5p	GLDC	ENSG00000178445	0.757
hsa-miR-30c-5p	RLIM	ENSG00000131263	0.776

hsa-miR-30c-5p	SEC14L2	ENSG00000100003	0.947
hsa-miR-30c-5p	SORCS3	ENSG00000156395	0.905
hsa-miR-30c-5p	PDE4D	ENSG00000113448	0.858
hsa-miR-30c-5p	ADRB1	ENSG00000043591	0.861
hsa-miR-30c-5p	GFPT2	ENSG00000131459	0.713
hsa-miR-30c-5p	RASGEF1B	ENSG00000138670	0.852
hsa-miR-30c-5p	ZBTB10	ENSG00000205189	0.813
hsa-miR-30c-5p	MYBL2	ENSG00000101057	0.653
hsa-miR-30c-5p	PPP1R9A	ENSG00000158528	0.857
hsa-miR-30c-5p	CCSER1	ENSG00000184305	1
hsa-miR-30c-5p	TENM1	ENSG00000009694	0.97
hsa-miR-30c-5p	NR4A2	ENSG00000153234	0.585
hsa-miR-30c-5p	SOCS1	ENSG00000185338	0.48
hsa-miR-30c-5p	ERRFI1	ENSG00000116285	0.855
hsa-miR-30c-5p	RHOB	ENSG00000143878	0.814
hsa-miR-30c-5p	RTN4R	ENSG00000040608	0.724
hsa-miR-30c-5p	EFNA3	ENSG00000143590	0.697
hsa-miR-30c-5p	P4HA2	ENSG00000072682	0.699
hsa-miR-30c-5p	CFL2	ENSG00000165410	0.685
hsa-miR-30c-5p	FAM43A	ENSG00000185112	0.522
hsa-miR-30c-5p	COL9A3	ENSG00000092758	0.618
hsa-miR-30c-5p	PRLR	ENSG00000113494	0.908
hsa-miR-30c-5p	TXNDC5	ENSG00000239264	0.784
hsa-miR-30c-5p	YES1	ENSG00000176105	0.85
hsa-miR-30c-5p	GRM3	ENSG00000198822	0.86
hsa-miR-30c-5p	JAM2	ENSG00000154721	0.921
hsa-miR-30c-5p	DBF4	ENSG00000006634	0.558
hsa-miR-30c-5p	PIGA	ENSG00000165195	0.772
hsa-miR-30c-5p	YOD1	ENSG00000180667	0.665
hsa-miR-30c-5p	TMEM170B	ENSG00000205269	0.761
hsa-miR-30c-5p	CHD1	ENSG00000153922	0.547
hsa-miR-30c-5p	C4orf19	ENSG00000154274	0.743
hsa-miR-32-5p	CDC42EP2	ENSG00000149798	0.838
hsa-miR-32-5p	CDKN1C	ENSG00000129757	0.597
hsa-miR-32-5p	TENM1	ENSG00000009694	0.97
hsa-miR-32-5p	SH3D19	ENSG00000109686	0.811
hsa-miR-32-5p	SKOR1	ENSG00000188779	0.729
hsa-miR-32-5p	CHCHD10	ENSG00000250479	0.51
hsa-miR-32-5p	ARHGEF10	ENSG00000104728	0.767
hsa-miR-32-5p	SMAD6	ENSG00000137834	0.956
hsa-miR-32-5p	CFL2	ENSG00000165410	0.868
hsa-miR-32-5p	NR4A3	ENSG00000119508	0.672
hsa-miR-32-5p	SIK1	ENSG00000142178	0.77
hsa-miR-32-5p	KLHL15	ENSG00000174010	0.593
hsa-miR-32-5p	TMEM255A	ENSG00000125355	0.731
hsa-miR-32-5p	ARHGAP29	ENSG00000137962	0.724
hsa-miR-32-5p	PPP1R9A	ENSG00000158528	0.968

hsa-miR-32-5p	ZBTB10	ENSG00000205189	0.908
hsa-miR-32-5p	ARL5B	ENSG00000165997	0.957
hsa-miR-32-5p	KLF2	ENSG00000127528	0.487
hsa-miR-32-5p	ZNF804A	ENSG00000170396	0.778
hsa-miR-32-5p	DUSP1	ENSG00000120129	0.831
hsa-miR-32-5p	ADRB1	ENSG00000043591	0.522
hsa-miR-32-5p	NETO1	ENSG00000166342	0.797
hsa-miR-32-5p	GOLGA8M	ENSG00000188626	0.812
hsa-miR-32-5p	AURKA	ENSG00000087586	0.634
hsa-miR-32-5p	GFPT2	ENSG00000131459	0.662
hsa-miR-32-5p	CHST7	ENSG00000147119	0.518
hsa-miR-32-5p	SERTAD2	ENSG00000179833	0.799
hsa-miR-32-5p	SORCS3	ENSG00000156395	0.94
hsa-miR-32-5p	FASLG	ENSG00000117560	0.749
hsa-miR-32-5p	INAFM1	ENSG00000257704	0.817
hsa-miR-340-5p	SRGAP1	ENSG00000196935	0.981
hsa-miR-340-5p	SERTAD2	ENSG00000179833	0.975
hsa-miR-340-5p	RNF103	ENSG00000239305	0.891
hsa-miR-340-5p	RGPD6	ENSG00000183054	0.97
hsa-miR-340-5p	AVPR1A	ENSG00000166148	0.991
hsa-miR-340-5p	RLIM	ENSG00000131263	0.98
hsa-miR-340-5p	PDK4	ENSG00000004799	0.949
hsa-miR-340-5p	DBF4	ENSG00000006634	0.921
hsa-miR-340-5p	GFPT2	ENSG00000131459	0.99
hsa-miR-340-5p	YOD1	ENSG00000180667	0.995
hsa-miR-340-5p	NR1D2	ENSG00000174738	0.988
hsa-miR-340-5p	FA2H	ENSG00000103089	0.99
hsa-miR-340-5p	HIF1A	ENSG00000100644	0.99
hsa-miR-340-5p	ARL5B	ENSG00000165997	0.979
hsa-miR-340-5p	YES1	ENSG00000176105	0.99
hsa-miR-340-5p	OSM	ENSG00000099985	0.944
hsa-miR-340-5p	NRARP	ENSG00000198435	0.995
hsa-miR-340-5p	FEM1C	ENSG00000145780	0.981
hsa-miR-340-5p	MET	ENSG00000105976	0.99
hsa-miR-340-5p	ZBTB10	ENSG00000205189	0.99
hsa-miR-340-5p	ARHGAP29	ENSG00000137962	0.983
hsa-miR-340-5p	TMEM170B	ENSG00000205269	0.955
hsa-miR-340-5p	GPR15	ENSG00000154165	0.97
hsa-miR-340-5p	ZBTB43	ENSG00000169155	0.955
hsa-miR-340-5p	NEXMIF	ENSG00000050030	0.95
hsa-miR-340-5p	RNF152	ENSG00000176641	0.995
hsa-miR-340-5p	KLHL15	ENSG00000174010	0.998
hsa-miR-340-5p	NPTX2	ENSG00000106236	0.993
hsa-miR-340-5p	ADAMTS1	ENSG00000154734	0.978
hsa-miR-340-5p	NR4A3	ENSG00000119508	0.92
hsa-miR-340-5p	RORB	ENSG00000198963	0.99
hsa-miR-340-5p	ZBTB21	ENSG00000173276	0.993

hsa-miR-340-5p	CFL2	ENSG00000165410	0.998
hsa-miR-362-5p	ZBTB10	ENSG00000205189	0.855
hsa-miR-362-5p	CHD1	ENSG00000153922	0.989
hsa-miR-362-5p	PAQR8	ENSG00000170915	0.709
hsa-miR-362-5p	RLIM	ENSG00000131263	0.9
hsa-miR-362-5p	CCSER1	ENSG00000184305	0.801
hsa-miR-365a-3p	SRGAP1	ENSG00000196935	0.745
hsa-miR-365a-3p	REL	ENSG00000162924	0.99
hsa-miR-365a-3p	KCNJ2	ENSG00000123700	0.665
hsa-miR-365a-3p	RGS20	ENSG00000147509	0.926
hsa-miR-365a-3p	NR1D2	ENSG00000174738	0.766
hsa-miR-365a-3p	CCSER1	ENSG00000184305	0.814
hsa-miR-365a-3p	PDE4D	ENSG00000113448	0.882
hsa-miR-365a-3p	ADAMTS1	ENSG00000154734	0.648
hsa-miR-365a-3p	RNF152	ENSG00000176641	0.641
hsa-miR-365a-3p	SERTAD2	ENSG00000179833	0.869
hsa-miR-365a-3p	NR4A2	ENSG00000153234	0.746
hsa-miR-374a-5p	ARL2BP	ENSG00000102931	0.945
hsa-miR-374a-5p	ZBTB10	ENSG00000205189	0.98
hsa-miR-374a-5p	TENM1	ENSG00000009694	0.98
hsa-miR-374a-5p	SMAD6	ENSG00000137834	0.697
hsa-miR-374a-5p	NR4A3	ENSG00000119508	0.981
hsa-miR-374a-5p	JAM2	ENSG00000154721	0.903
hsa-miR-374a-5p	CEBPB	ENSG00000172216	0.782
hsa-miR-374a-5p	ADRB1	ENSG00000043591	0.952
hsa-miR-374a-5p	YOD1	ENSG00000180667	0.95
hsa-miR-374a-5p	PDE4D	ENSG00000113448	0.98
hsa-miR-374a-5p	SRGAP1	ENSG00000196935	0.987
hsa-miR-374a-5p	SERTAD2	ENSG00000179833	0.996
hsa-miR-374a-5p	RORB	ENSG00000198963	0.98
hsa-miR-374a-5p	DGAT2	ENSG00000062282	0.868
hsa-miR-374a-5p	NR4A2	ENSG00000153234	0.98
hsa-miR-374a-5p	ZBTB43	ENSG00000169155	0.97
hsa-miR-374a-5p	NPTX2	ENSG00000106236	0.916
hsa-miR-374a-5p	HES1	ENSG00000114315	0.852
hsa-miR-449a	CHD1	ENSG00000153922	0.693
hsa-miR-449a	ZNF579	ENSG00000218891	0.797
hsa-miR-449a	CDKN1C	ENSG00000129757	0.727
hsa-miR-449a	SLC23A3	ENSG00000213901	0.745
hsa-miR-449a	RNF152	ENSG00000176641	0.877
hsa-miR-449a	MET	ENSG00000105976	0.367
hsa-miR-449a	CACNA1E	ENSG00000198216	0.825
hsa-miR-449a	NR4A2	ENSG00000153234	0.635
hsa-miR-449a	EML5	ENSG00000165521	0.605
hsa-miR-449a	NETO1	ENSG00000166342	0.536
hsa-miR-449a	TMEM250	ENSG00000238227	0.526
hsa-miR-449a	TMEM255A	ENSG00000125355	0.967

hsa-miR-454-3p	PRICKLE2	ENSG00000163637	0.825
hsa-miR-454-3p	RORB	ENSG00000198963	0.915
hsa-miR-454-3p	PPP1R9A	ENSG00000158528	0.915
hsa-miR-454-3p	SOX5	ENSG00000134532	0.946
hsa-miR-454-3p	PIGA	ENSG00000165195	0.608
hsa-miR-454-3p	KLHL15	ENSG00000174010	0.894
hsa-miR-454-3p	TMEM250	ENSG00000238227	0.539
hsa-miR-454-3p	NEXMIF	ENSG00000050030	0.776
hsa-miR-454-3p	FAM43A	ENSG00000185112	0.702
hsa-miR-454-3p	TMEM170B	ENSG00000205269	0.672
hsa-miR-454-3p	DEPDC1	ENSG00000024526	0.794
hsa-miR-454-3p	NRARP	ENSG00000198435	0.864
hsa-miR-454-3p	DIAPH3	ENSG00000139734	0.849
hsa-miR-454-3p	MET	ENSG00000105976	0.625
hsa-miR-454-3p	SH3D19	ENSG00000109686	0.422
hsa-miR-454-3p	TENM1	ENSG00000009694	0.97
hsa-miR-454-3p	PFKFB3	ENSG00000170525	0.868
hsa-miR-454-3p	SYNM	ENSG00000182253	0.843
hsa-miR-454-3p	COL9A3	ENSG00000092758	0.987
hsa-miR-454-3p	EREG	ENSG00000124882	0.411
hsa-miR-454-3p	SIK1	ENSG00000142178	0.803
hsa-miR-454-3p	CFL2	ENSG00000165410	0.909
hsa-miR-454-3p	RLIM	ENSG00000131263	0.815
hsa-miR-454-3p	PDE4D	ENSG00000113448	0.853
hsa-miR-490-3p	TMEM170B	ENSG00000205269	0.864
hsa-miR-490-3p	AURKA	ENSG00000087586	0.749
hsa-miR-504-5p	SDC1	ENSG00000115884	0.836
hsa-miR-504-5p	RORB	ENSG00000198963	0.861
hsa-miR-504-5p	NR4A3	ENSG00000119508	0.843
hsa-miR-504-5p	AOC3	ENSG00000131471	0.91
hsa-miR-504-5p	ZBTB43	ENSG00000169155	0.659
hsa-miR-504-5p	FZD7	ENSG00000155760	0.882
hsa-miR-506-3p	JAM2	ENSG00000154721	0.902
hsa-miR-506-3p	PPP1R9A	ENSG00000158528	0.863
hsa-miR-506-3p	RLIM	ENSG00000131263	0.679
hsa-miR-506-3p	PRLR	ENSG00000113494	0.902
hsa-miR-506-3p	GOLGA8M	ENSG00000188626	0.729
hsa-miR-506-3p	LRFN1	ENSG00000128011	0.903
hsa-miR-506-3p	CHD1	ENSG00000153922	0.72
hsa-miR-506-3p	CFL2	ENSG00000165410	0.646
hsa-miR-506-3p	YOD1	ENSG00000180667	0.657
hsa-miR-506-3p	EML5	ENSG00000165521	0.838
hsa-miR-506-3p	PAQR8	ENSG00000170915	0.833
hsa-miR-506-3p	PDE4D	ENSG00000113448	0.979
hsa-miR-506-3p	HIC1	ENSG00000177374	0.953
hsa-miR-506-3p	DEPDC1	ENSG00000024526	0.719
hsa-miR-506-3p	MOCS1	ENSG00000124615	0.98

hsa-miR-506-3p	TXNDC5	ENSG00000239264	0.845
hsa-miR-506-3p	MCM10	ENSG00000065328	0.871
hsa-miR-506-3p	NR1D2	ENSG00000174738	0.936
hsa-miR-506-3p	P4HA2	ENSG00000072682	0.791
hsa-miR-506-3p	PRICKLE2	ENSG00000163637	0.98
hsa-miR-506-3p	KCNJ2	ENSG00000123700	0.775
hsa-miR-590-5p	SOX5	ENSG00000134532	0.659
hsa-miR-590-5p	YOD1	ENSG00000180667	0.601
hsa-miR-590-5p	KLHL15	ENSG00000174010	0.962
hsa-miR-590-5p	FASLG	ENSG00000117560	0.444
hsa-miR-590-5p	RHOB	ENSG00000143878	0.912
hsa-miR-590-5p	PIK3R1	ENSG00000145675	0.893
hsa-miR-590-5p	DUSP8	ENSG00000184545	0.721
hsa-miR-590-5p	ESM1	ENSG00000164283	0.91
hsa-miR-708-5p	SRGAP1	ENSG00000196935	0.937
hsa-miR-708-5p	CD180	ENSG00000134061	1
hsa-miR-708-5p	OTOF	ENSG00000115155	0.814
hsa-miR-758-3p	DUSP1	ENSG00000120129	0.909
hsa-miR-758-3p	ZNF613	ENSG00000176024	0.78
hsa-miR-758-3p	RBFOX3	ENSG00000167281	0.884
hsa-miR-758-3p	CFL2	ENSG00000165410	0.972
hsa-miR-758-3p	SYNM	ENSG00000182253	0.953
hsa-miR-758-3p	OTX1	ENSG00000115507	0.961
hsa-miR-877-5p	RNF152	ENSG00000176641	0.833
hsa-miR-877-5p	OTOF	ENSG00000115155	0.98
hsa-miR-877-5p	NR4A3	ENSG00000119508	0.965
hsa-miR-9-5p	PRLR	ENSG00000113494	0.969
hsa-miR-9-5p	GJA3	ENSG00000121743	0.748
hsa-miR-9-5p	KCNJ2	ENSG00000123700	0.38
hsa-miR-9-5p	MCM10	ENSG00000065328	0.926
hsa-miR-9-5p	RORB	ENSG00000198963	0.901
hsa-miR-9-5p	TMEM170B	ENSG00000205269	0.791
hsa-miR-9-5p	SIK1	ENSG00000142178	0.752
hsa-miR-9-5p	ZBTB21	ENSG00000173276	0.924
hsa-miR-9-5p	TENM1	ENSG00000009694	0.944
hsa-miR-9-5p	SDC1	ENSG00000115884	0.836
hsa-miR-9-5p	PIM3	ENSG00000198355	0.865
hsa-miR-9-5p	TXNDC5	ENSG00000239264	0.865
hsa-miR-9-5p	EML5	ENSG00000165521	0.934
hsa-miR-9-5p	P4HA2	ENSG00000072682	0.689
hsa-miR-9-5p	CCSER1	ENSG00000184305	0.909
hsa-miR-9-5p	ISL2	ENSG00000159556	1
hsa-miR-9-5p	ARHGAP39	ENSG00000147799	0.98
hsa-miR-9-5p	HES1	ENSG00000114315	0.799
hsa-miR-9-5p	RANBP17	ENSG00000204764	0.466
hsa-miR-9-5p	PGAP1	ENSG00000197121	0.985
hsa-miR-9-5p	PDK4	ENSG00000004799	0.459

hsa-miR-9-5p	CACNA1E	ENSG00000198216	0.972
hsa-miR-9-5p	CCNT1	ENSG00000129315	0.9
hsa-miR-9-5p	HIC1	ENSG00000177374	0.819
hsa-miR-9-5p	DUSP8	ENSG00000184545	0.982
hsa-miR-92a-3p	SERTAD2	ENSG00000179833	0.805
hsa-miR-92a-3p	NETO1	ENSG00000166342	0.845
hsa-miR-92a-3p	SKOR1	ENSG00000188779	0.729
hsa-miR-92a-3p	ARHGAP29	ENSG00000137962	0.779
hsa-miR-92a-3p	TENM1	ENSG00000009694	0.97
hsa-miR-92a-3p	INAFM1	ENSG00000257704	0.793
hsa-miR-92a-3p	GOLGA8M	ENSG00000188626	0.78
hsa-miR-92a-3p	ARHGEF10	ENSG00000104728	0.743
hsa-miR-92a-3p	CDC42EP2	ENSG00000149798	0.802
hsa-miR-92a-3p	NR4A3	ENSG00000119508	0.672
hsa-miR-92a-3p	ARL5B	ENSG00000165997	0.957
hsa-miR-92a-3p	CHCHD10	ENSG00000250479	0.499
hsa-miR-92a-3p	GFPT2	ENSG00000131459	0.67
hsa-miR-92a-3p	TMEM255A	ENSG00000125355	0.707
hsa-miR-92a-3p	CDKN1C	ENSG00000129757	0.608
hsa-miR-92a-3p	FASLG	ENSG00000117560	0.771
hsa-miR-92a-3p	SH3D19	ENSG00000109686	0.811
hsa-miR-92a-3p	AURKA	ENSG00000087586	0.642
hsa-miR-92a-3p	PPP1R9A	ENSG00000158528	0.975
hsa-miR-92a-3p	SORCS3	ENSG00000156395	0.916
hsa-miR-92a-3p	SMAD6	ENSG00000137834	0.963
hsa-miR-92a-3p	DUSP1	ENSG00000120129	0.819
hsa-miR-92a-3p	KLHL15	ENSG00000174010	0.617
hsa-miR-92a-3p	CHST7	ENSG00000147119	0.54
hsa-miR-92a-3p	SIK1	ENSG00000142178	0.758
hsa-miR-92a-3p	KLF2	ENSG00000127528	0.503
hsa-miR-92a-3p	ZNF804A	ENSG00000170396	0.826
hsa-miR-92a-3p	ZBTB10	ENSG00000205189	0.98
hsa-miR-92a-3p	CFL2	ENSG00000165410	0.856
hsa-miR-92a-3p	ADRB1	ENSG00000043591	0.522
hsa-miR-93-5p	FEM1C	ENSG00000145780	0.647
hsa-miR-93-5p	YES1	ENSG00000176105	0.848
hsa-miR-93-5p	TNFSF11	ENSG00000120659	0.757
hsa-miR-93-5p	ZNF703	ENSG00000183779	1
hsa-miR-93-5p	KLHL15	ENSG00000174010	0.767
hsa-miR-93-5p	BTG3	ENSG00000154640	0.332
hsa-miR-93-5p	RLIM	ENSG00000131263	0.701
hsa-miR-93-5p	SYNM	ENSG00000182253	0.931
hsa-miR-93-5p	NR4A2	ENSG00000153234	0.725
hsa-miR-93-5p	SLC1A2	ENSG00000110436	0.98
hsa-miR-93-5p	AHRR	ENSG00000063438	0.983
hsa-miR-93-5p	OSM	ENSG00000099985	0.628
hsa-miR-93-5p	HIF1A	ENSG00000100644	0.796

hsa-miR-93-5p	SMAD6	ENSG00000137834	0.969
hsa-miR-93-5p	PER1	ENSG00000179094	0.964
hsa-miR-93-5p	SRGAP1	ENSG00000196935	0.894
hsa-miR-93-5p	CFL2	ENSG00000165410	0.559
hsa-miR-93-5p	TIPARP	ENSG00000163659	0.863
hsa-miR-93-5p	SKOR1	ENSG00000188779	0.707
hsa-miR-93-5p	ZBTB21	ENSG00000173276	0.91
hsa-miR-93-5p	SERTAD2	ENSG00000179833	0.825
hsa-miR-93-5p	MASTL	ENSG00000120539	0.476
hsa-miR-93-5p	PANX2	ENSG00000073150	0.805
hsa-miR-93-5p	ZNF805	ENSG00000204524	0.935
hsa-miR-93-5p	TENM1	ENSG00000009694	0.874
hsa-miR-93-5p	EREG	ENSG00000124882	0.693
hsa-miR-93-5p	NR4A3	ENSG00000119508	0.624
hsa-miR-93-5p	CXCL8	ENSG00000169429	0.572
hsa-miR-93-5p	SIK1	ENSG00000142178	0.72
hsa-miR-93-5p	ZBTB43	ENSG00000169155	0.799
hsa-miR-93-5p	PFKFB3	ENSG00000170525	0.822
hsa-miR-93-5p	RORB	ENSG00000198963	0.961
hsa-miR-93-5p	ARHGEF10	ENSG00000104728	0.773
hsa-miR-93-5p	CHST7	ENSG00000147119	0.999
hsa-miR-93-5p	YOD1	ENSG00000180667	0.852
hsa-miR-93-5p	PIK3R1	ENSG00000145675	0.981
hsa-miR-93-5p	DUSP8	ENSG00000184545	0.905
hsa-miR-98-5p	ZBTB10	ENSG00000205189	0.784
hsa-miR-98-5p	TNFAIP3	ENSG00000118503	0.887
hsa-miR-98-5p	YOD1	ENSG00000180667	0.205
hsa-miR-98-5p	ARL5B	ENSG00000165997	0.969
hsa-miR-98-5p	ADAMTS1	ENSG00000154734	0.823
hsa-miR-98-5p	P4HA2	ENSG00000072682	0.771
hsa-miR-98-5p	NAT8L	ENSG00000185818	0.686
hsa-miR-98-5p	PANX2	ENSG00000073150	0.758
hsa-miR-98-5p	TNFSF9	ENSG00000125657	0.363
hsa-miR-98-5p	IL13	ENSG00000169194	0.478
hsa-miR-98-5p	CACNA1E	ENSG00000198216	0.851
hsa-miR-98-5p	TMEM255A	ENSG00000125355	0.757
hsa-miR-98-5p	B3GNT7	ENSG00000156966	0.7
hsa-miR-98-5p	SRGAP1	ENSG00000196935	0.866
hsa-miR-98-5p	NIPAL4	ENSG00000172548	0.722
hsa-miR-98-5p	CFL2	ENSG00000165410	0.703
hsa-miR-98-5p	FASLG	ENSG00000117560	0.571
hsa-miR-98-5p	OTOF	ENSG00000115155	0.887
hsa-miR-98-5p	SOCS1	ENSG00000185338	0.583
hsa-miR-98-5p	SPRED3	ENSG00000188766	0.795
hsa-miR-98-5p	PRLR	ENSG00000113494	0.729
hsa-miR-98-5p	COL9A3	ENSG00000092758	0.727
hsa-miR-98-5p	PIGA	ENSG00000165195	0.589

hsa-miR-98-5p	CEBPD	ENSG00000221869	0.691
hsa-miR-98-5p	HIC1	ENSG00000177374	0.777
hsa-miR-98-5p	RNF152	ENSG00000176641	0.806
hsa-miR-98-5p	REL	ENSG00000162924	0.97
hsa-miR-98-5p	COL1A1	ENSG00000108821	0.727
hsa-miR-98-5p	FAM43A	ENSG00000185112	0.801
hsa-miR-98-5p	NEXMIF	ENSG00000050030	0.783
hsa-miR-98-5p	ABCB9	ENSG00000150967	0.556
hsa-miR-98-5p	DUSP1	ENSG00000120129	0.662
hsa-miR-98-5p	ADRB1	ENSG00000043591	0.633
hsa-miR-98-5p	YOD1	ENSG00000180667	1
hsa-miR-449a	TMEM255A	ENSG00000125355	1
hsa-miR-30c-5p	TMEM170B	ENSG00000205269	1
hsa-miR-548x-5p	TENM1	ENSG00000009694	1
hsa-miR-30c-5p	SOCS1	ENSG00000185338	1
hsa-miR-20a-5p	SKOR1	ENSG00000188779	1
hsa-miR-1299	SKA3	ENSG00000165480	1
hsa-miR-20a-5p	SIK1	ENSG00000142178	1
hsa-miR-153-3p	SERTAD2	ENSG00000179833	1
hsa-miR-129-5p	RBFOX3	ENSG00000167281	1
hsa-miR-513a-5p	PKD2L2	ENSG00000078795	1
hsa-miR-454-3p	MET	ENSG00000105976	1
hsa-miR-153-3p	GFPT2	ENSG00000131459	1
hsa-miR-20a-5p	CXCL8	ENSG00000169429	1
hsa-miR-20a-5p	BTG3	ENSG00000154640	1
hsa-miR-20a-5p	YOD1	ENSG00000180667	0.999
hsa-miR-21-5p	YOD1	ENSG00000180667	0.999
hsa-miR-147a	TCTE1	ENSG00000146221	0.999
hsa-miR-21-5p	SOX5	ENSG00000134532	0.999
hsa-miR-219a-5p	SOX5	ENSG00000134532	0.999
hsa-miR-513b-5p	SOX5	ENSG00000134532	0.999
hsa-miR-454-3p	SH3D19	ENSG00000109686	0.999
hsa-miR-30c-5p	RHOB	ENSG00000143878	0.999
hsa-miR-20a-5p	OSM	ENSG00000099985	0.999
hsa-miR-30c-5p	NR4A2	ENSG00000153234	0.999
hsa-miR-365a-3p	NCR3LG1	ENSG00000188211	0.999
hsa-miR-4717-5p	NCR3LG1	ENSG00000188211	0.999
hsa-miR-30c-5p	MYBL2	ENSG00000101057	0.999
hsa-miR-4775	LRG1	ENSG00000171236	0.999
hsa-miR-98-5p	IL13	ENSG00000169194	0.999
hsa-miR-101-3p	ZBTB21	ENSG00000173276	0.998
hsa-miR-374a-5p	YOD1	ENSG00000180667	0.998
hsa-miR-574-5p	TM4SF19	ENSG00000145107	0.998
hsa-miR-340-5p	SRGAP1	ENSG00000196935	0.998
hsa-miR-144-3p	SMAD9	ENSG00000120693	0.998
hsa-miR-92a-3p	SIK1	ENSG00000142178	0.998
hsa-miR-371b-5p	RLIM	ENSG00000131263	0.998

hsa-miR-616-5p	RLIM	ENSG00000131263	0.998
hsa-miR-144-3p	REL	ENSG00000162924	0.998
hsa-miR-1299	PRLR	ENSG00000113494	0.998
hsa-miR-193a-3p	PIGA	ENSG00000165195	0.998
hsa-miR-147a	NR4A2	ENSG00000153234	0.998
hsa-miR-125b-5p	NCR3LG1	ENSG00000188211	0.998
hsa-miR-20a-5p	NCR3LG1	ENSG00000188211	0.998
hsa-miR-9-5p	KCNJ2	ENSG00000123700	0.998
hsa-miR-153-3p	HEY2	ENSG00000135547	0.998
hsa-miR-30c-5p	GLDC	ENSG00000178445	0.998
hsa-miR-625-5p	GIMAP1	ENSG00000213203	0.998
hsa-miR-30c-5p	GFPT2	ENSG00000131459	0.998
hsa-miR-32-5p	GFPT2	ENSG00000131459	0.998
hsa-miR-101-3p	FEM1C	ENSG00000145780	0.998
hsa-miR-147a	C3orf35	ENSG00000198590	0.998
hsa-miR-340-5p	ADAMTS1	ENSG00000154734	0.998
hsa-miR-144-3p	ZBTB21	ENSG00000173276	0.997
hsa-miR-513a-5p	SLC1A2	ENSG00000110436	0.997
hsa-miR-548ah-3p	SERTAD2	ENSG00000179833	0.997
hsa-miR-101-3p	PDE4D	ENSG00000113448	0.997
hsa-miR-374a-5p	NCR3LG1	ENSG00000188211	0.997
hsa-miR-92a-3p	GFPT2	ENSG00000131459	0.997
hsa-miR-101-3p	DUSP1	ENSG00000120129	0.997
hsa-miR-147a	AOC1	ENSG00000002726	0.997
hsa-miR-590-5p	YOD1	ENSG00000180667	0.996
hsa-miR-9-5p	TENM1	ENSG00000009694	0.996
hsa-miR-4717-5p	KIAA1324	ENSG00000116299	0.996
hsa-miR-9-5p	GCSAM	ENSG00000174500	0.996
hsa-miR-30c-5p	EFNA3	ENSG00000143590	0.996
hsa-miR-624-5p	YES1	ENSG00000176105	0.995
hsa-miR-129-5p	TNFSF11	ENSG00000120659	0.995
hsa-miR-92a-3p	TMEM255A	ENSG00000125355	0.995
hsa-miR-144-3p	SORCS3	ENSG00000156395	0.995
hsa-miR-340-5p	NCR3LG1	ENSG00000188211	0.995
hsa-miR-551b-5p	ZNF805	ENSG00000204524	0.994
hsa-miR-9-5p	RGPD6	ENSG00000183054	0.994
hsa-miR-30c-5p	PIGA	ENSG00000165195	0.994
hsa-miR-548s	PIGA	ENSG00000165195	0.994
hsa-miR-340-5p	NEXMIF	ENSG00000050030	0.994
hsa-miR-548ah-3p	EREG	ENSG00000124882	0.994
hsa-miR-454-3p	DIAPH3	ENSG00000139734	0.994
hsa-miR-548ah-3p	SOX5	ENSG00000134532	0.993
hsa-miR-548x-5p	PRLR	ENSG00000113494	0.993
hsa-miR-193b-3p	PIGA	ENSG00000165195	0.993
hsa-miR-20a-5p	NR4A2	ENSG00000153234	0.993
hsa-miR-301a-3p	MET	ENSG00000105976	0.993
hsa-miR-513a-5p	ENC1	ENSG00000171617	0.993

hsa-miR-551b-5p	ZBTB21	ENSG00000173276	0.992
hsa-miR-4775	YOD1	ENSG00000180667	0.992
hsa-miR-506-3p	SERTAD2	ENSG00000179833	0.992
hsa-miR-17-5p	NR4A3	ENSG00000119508	0.992
hsa-miR-20a-5p	MASTL	ENSG00000120539	0.992
hsa-miR-758-3p	EML5	ENSG00000165521	0.992
hsa-miR-32-5p	ARL5B	ENSG00000165997	0.992
hsa-miR-30c-5p	YOD1	ENSG00000180667	0.991
hsa-miR-24-3p	TENM1	ENSG00000009694	0.991
hsa-miR-199b-5p	SORCS3	ENSG00000156395	0.991
hsa-miR-32-5p	SIK1	ENSG00000142178	0.991
hsa-miR-548ah-3p	PDE4D	ENSG00000113448	0.991
hsa-miR-1299	MANSC1	ENSG00000111261	0.991
hsa-miR-101-3p	FOS	ENSG00000170345	0.991
hsa-miR-30c-5p	FAM43A	ENSG00000185112	0.991
hsa-miR-24-3p	CITED4	ENSG00000179862	0.991
hsa-miR-206	ZNF547	ENSG00000152433	0.99
hsa-miR-30c-5p	TENM1	ENSG00000009694	0.99
hsa-miR-4423-5p	SKA3	ENSG00000165480	0.99
hsa-miR-92a-3p	SH3D19	ENSG00000109686	0.99
hsa-miR-500a-5p	SERTAD2	ENSG00000179833	0.99
hsa-miR-30c-5p	RTN4R	ENSG00000040608	0.99
hsa-miR-206	NEXMIF	ENSG00000050030	0.99
hsa-miR-374a-5p	NETO1	ENSG00000166342	0.99
hsa-miR-548ah-3p	KLHL15	ENSG00000174010	0.99
hsa-miR-20a-5p	FEM1C	ENSG00000145780	0.99
hsa-miR-4645-5p	EREG	ENSG00000124882	0.99
hsa-miR-92a-3p	ARL5B	ENSG00000165997	0.99
hsa-miR-218-5p	SERTAD2	ENSG00000179833	0.989
hsa-miR-9-5p	RGPD5	ENSG00000015568	0.989
hsa-miR-218-5p	PRLR	ENSG00000113494	0.989
hsa-miR-20a-5p	ARHGEF10	ENSG00000104728	0.989
hsa-miR-15b-5p	PIK3R1	ENSG00000145675	0.988
hsa-miR-20a-5p	NR4A3	ENSG00000119508	0.988
hsa-miR-3150a-3p	ICOSLG	ENSG00000160223	0.988
hsa-miR-548ah-3p	CCNT1	ENSG00000129315	0.988
hsa-miR-340-5p	ZBTB10	ENSG00000205189	0.987
hsa-miR-124-3p	SERTAD2	ENSG00000179833	0.987
hsa-miR-548a-5p	SERTAD2	ENSG00000179833	0.987
hsa-miR-15a-5p	PIK3R1	ENSG00000145675	0.987
hsa-miR-374a-5p	PGAP1	ENSG00000197121	0.987
hsa-miR-340-5p	KLHL15	ENSG00000174010	0.987
hsa-miR-193a-3p	KCNJ2	ENSG00000123700	0.987
hsa-miR-98-5p	IFITM10	ENSG00000244242	0.987
hsa-miR-374a-5p	FPR3	ENSG00000187474	0.987
hsa-miR-454-3p	EREG	ENSG00000124882	0.987
hsa-miR-628-3p	ADAMTS1	ENSG00000154734	0.987

hsa-miR-92a-3p	ZNF804A	ENSG00000170396	0.986
hsa-miR-147a	TREML2	ENSG00000112195	0.986
hsa-miR-144-3p	TNFSF11	ENSG00000120659	0.986
hsa-miR-1972	TNFAIP3	ENSG00000118503	0.986
hsa-miR-548ah-3p	SIK1	ENSG00000142178	0.986
hsa-miR-432-5p	RASGRF1	ENSG00000058335	0.986
hsa-miR-30c-5p	PRLR	ENSG00000113494	0.986
hsa-miR-340-5p	NRARP	ENSG00000198435	0.986
hsa-miR-3074-5p	NR4A3	ENSG00000119508	0.986
hsa-miR-93-5p	NR4A3	ENSG00000119508	0.986
hsa-miR-513b-5p	NEXMIF	ENSG00000050030	0.986
hsa-miR-548ah-5p	NCR3LG1	ENSG00000188211	0.986
hsa-miR-548ah-3p	KCNJ2	ENSG00000123700	0.986
hsa-miR-4775	FEM1C	ENSG00000145780	0.986
hsa-miR-513b-5p	FASLG	ENSG00000117560	0.986
hsa-miR-20a-5p	CFL2	ENSG00000165410	0.986
hsa-miR-129-5p	CDKN1C	ENSG00000129757	0.986
hsa-miR-4775	ARL5B	ENSG00000165997	0.986
hsa-miR-98-5p	TNFSF9	ENSG00000125657	0.985
hsa-miR-124-3p	PGAP1	ENSG00000197121	0.985
hsa-miR-548ai	NCR3LG1	ENSG00000188211	0.985
hsa-miR-32-5p	KLF2	ENSG00000127528	0.985
hsa-miR-92a-3p	ZBTB10	ENSG00000205189	0.984
hsa-miR-24-3p	U2AF1	ENSG00000160201	0.984
hsa-miR-101-3p	SRGAP1	ENSG00000196935	0.984
hsa-miR-625-5p	SERTAD2	ENSG00000179833	0.984
hsa-miR-3074-5p	PDE4D	ENSG00000113448	0.984
hsa-miR-10a-5p	NR4A3	ENSG00000119508	0.984
hsa-miR-513b-5p	FA2H	ENSG00000103089	0.984
hsa-miR-449a	CACNA1E	ENSG00000198216	0.984
hsa-miR-3150a-3p	ARHGAP39	ENSG00000147799	0.984
hsa-miR-32-5p	ZBTB10	ENSG00000205189	0.983
hsa-miR-628-3p	NEXMIF	ENSG00000050030	0.983
hsa-miR-30c-5p	CFL2	ENSG00000165410	0.983
hsa-miR-664b-3p	YOD1	ENSG00000180667	0.982
hsa-miR-340-5p	PDE4D	ENSG00000113448	0.982
hsa-miR-4775	NR1D2	ENSG00000174738	0.982
hsa-miR-125b-5p	FPR3	ENSG00000187474	0.982
hsa-miR-301a-3p	EREG	ENSG00000124882	0.982
hsa-miR-10a-5p	TMEM170B	ENSG00000205269	0.981
hsa-miR-551b-5p	PPP1R9A	ENSG00000158528	0.981
hsa-miR-548x-5p	NR4A3	ENSG00000119508	0.981
hsa-miR-513a-5p	GALNT5	ENSG00000136542	0.981
hsa-miR-548s	EML5	ENSG00000165521	0.981
hsa-miR-199a-5p	SORCS3	ENSG00000156395	0.98
hsa-miR-301a-3p	PIGA	ENSG00000165195	0.98
hsa-miR-9-5p	PDK4	ENSG00000004799	0.98

hsa-miR-1299	NCR3LG1	ENSG00000188211	0.98
hsa-miR-30c-5p	GRM3	ENSG00000198822	0.98
hsa-miR-340-5p	SIAH1	ENSG00000196470	0.979
hsa-miR-21-5p	FASLG	ENSG00000117560	0.979
hsa-miR-153-3p	EFNA3	ENSG00000143590	0.979
hsa-miR-4775	E2F8	ENSG00000129173	0.979
hsa-miR-664a-3p	TMEM255A	ENSG00000125355	0.978
hsa-miR-24-3p	SCML1	ENSG00000047634	0.978
hsa-miR-4775	NEXMIF	ENSG00000050030	0.978
hsa-miR-188-5p	STEAP4	ENSG00000127954	0.977
hsa-miR-340-5p	PGAP1	ENSG00000197121	0.977
hsa-miR-506-3p	PDE4D	ENSG00000113448	0.977
hsa-miR-3064-5p	NCR3LG1	ENSG00000188211	0.977
hsa-miR-548a-5p	FZD7	ENSG00000155760	0.977
hsa-miR-340-5p	YOD1	ENSG00000180667	0.976
hsa-miR-30c-5p	NCR3LG1	ENSG00000188211	0.976
hsa-miR-21-5p	KLHL15	ENSG00000174010	0.976
hsa-miR-92a-3p	KLF2	ENSG00000127528	0.976
hsa-miR-432-5p	KIAA1324	ENSG00000116299	0.976
hsa-miR-548ah-3p	GCSAML	ENSG00000169224	0.976
hsa-miR-340-5p	CCSER1	ENSG00000184305	0.976
hsa-miR-125b-5p	TNFAIP3	ENSG00000118503	0.975
hsa-miR-144-3p	PDE4D	ENSG00000113448	0.975
hsa-miR-548a-5p	HEY2	ENSG00000135547	0.975
hsa-miR-147a	E2F8	ENSG00000129173	0.975
hsa-miR-548x-5p	TMEM255A	ENSG00000125355	0.974
hsa-miR-32-5p	SKOR1	ENSG00000188779	0.974
hsa-miR-20a-5p	SERTAD2	ENSG00000179833	0.974
hsa-miR-551b-5p	RLIM	ENSG00000131263	0.974
hsa-miR-506-3p	PGAP1	ENSG00000197121	0.974
hsa-miR-1303	OTOF	ENSG00000115155	0.974
hsa-miR-129-5p	NCR3LG1	ENSG00000188211	0.974
hsa-miR-365b-5p	NCR3LG1	ENSG00000188211	0.974
hsa-miR-129-5p	ZBTB10	ENSG00000205189	0.973
hsa-miR-4775	TNFSF11	ENSG00000120659	0.973
hsa-miR-501-5p	NEXMIF	ENSG00000050030	0.973
hsa-miR-301a-3p	FAM43A	ENSG00000185112	0.973
hsa-miR-548ah-3p	BTG3	ENSG00000154640	0.973
hsa-miR-548ah-3p	ZNF805	ENSG00000204524	0.972
hsa-miR-551b-5p	YOD1	ENSG00000180667	0.972
hsa-miR-551b-5p	RNF152	ENSG00000176641	0.972
hsa-miR-500a-5p	ZNF805	ENSG00000204524	0.971
hsa-miR-664a-3p	ZNF781	ENSG00000196381	0.971
hsa-miR-340-5p	ZBTB21	ENSG00000173276	0.971
hsa-miR-32-5p	SERTAD2	ENSG00000179833	0.971
hsa-miR-501-5p	NR4A3	ENSG00000119508	0.971
hsa-miR-9-5p	NCR3LG1	ENSG00000188211	0.971

hsa-miR-664a-3p	NAT2	ENSG00000156006	0.971
hsa-miR-548ah-3p	COL1A1	ENSG00000108821	0.971
hsa-miR-548ah-3p	YES1	ENSG00000176105	0.97
hsa-miR-4482-5p	RORB	ENSG00000198963	0.97
hsa-miR-124-3p	PDE4D	ENSG00000113448	0.97
hsa-miR-3613-5p	NCR3LG1	ENSG00000188211	0.97
hsa-miR-365a-5p	NCR3LG1	ENSG00000188211	0.97
hsa-miR-664a-3p	LRRIQ3	ENSG00000162620	0.97
hsa-miR-500a-5p	TMEM170B	ENSG00000205269	0.969
hsa-miR-301a-3p	SH3D19	ENSG00000109686	0.969
hsa-miR-32-5p	SH3D19	ENSG00000109686	0.969
hsa-miR-30c-5p	P4HA2	ENSG00000072682	0.969
hsa-miR-103a-3p	SRGAP1	ENSG00000196935	0.968
hsa-miR-539-5p	SLC1A2	ENSG00000110436	0.968
hsa-miR-548a-5p	SCML1	ENSG00000047634	0.968
hsa-miR-506-3p	MOCS1	ENSG00000124615	0.968
hsa-miR-24-3p	KCNJ2	ENSG00000123700	0.968
hsa-miR-513b-5p	IER5L	ENSG00000188483	0.968
hsa-miR-374a-5p	CEBPB	ENSG00000172216	0.968
hsa-miR-548ah-3p	ARHGAP29	ENSG00000137962	0.968
hsa-miR-1299	ZNF331	ENSG00000130844	0.967
hsa-miR-340-5p	TMEM170B	ENSG00000205269	0.967
hsa-miR-153-3p	GRIK4	ENSG00000149403	0.967
hsa-miR-4775	CFL2	ENSG00000165410	0.967
hsa-miR-20a-5p	ZBTB21	ENSG00000173276	0.966
hsa-miR-30c-5p	SCML1	ENSG00000047634	0.966
hsa-miR-449a	MET	ENSG00000105976	0.966
hsa-miR-32-5p	KLHL15	ENSG00000174010	0.966
hsa-miR-500a-3p	ZBTB43	ENSG00000169155	0.965
hsa-miR-664a-3p	YES1	ENSG00000176105	0.965
hsa-miR-4775	TMEM255A	ENSG00000125355	0.965
hsa-miR-539-5p	SOX5	ENSG00000134532	0.965
hsa-miR-660-5p	SOX5	ENSG00000134532	0.965
hsa-miR-129-5p	NEXMIF	ENSG00000050030	0.965
hsa-miR-124-3p	MOCS1	ENSG00000124615	0.965
hsa-miR-340-5p	IER5L	ENSG00000188483	0.965
hsa-miR-144-3p	E2F8	ENSG00000129173	0.965
hsa-miR-30c-5p	DBF4	ENSG00000006634	0.965
hsa-miR-20a-5p	ZNF805	ENSG00000204524	0.964
hsa-miR-664b-3p	ZBTB10	ENSG00000205189	0.964
hsa-miR-153-3p	PIK3R1	ENSG00000145675	0.964
hsa-miR-101-3p	NEXMIF	ENSG00000050030	0.964
hsa-miR-4775	KCNJ2	ENSG00000123700	0.964
hsa-miR-548ah-5p	YES1	ENSG00000176105	0.963
hsa-miR-340-5p	SLC1A2	ENSG00000110436	0.963
hsa-miR-664a-3p	GCSAML	ENSG00000169224	0.963
hsa-miR-616-5p	ZNF805	ENSG00000204524	0.962

hsa-miR-548a-5p	PRLR	ENSG00000113494	0.962
hsa-miR-92a-3p	PPP1R9A	ENSG00000158528	0.962
hsa-miR-187-3p	GRIN1	ENSG00000176884	0.962
hsa-miR-3180-5p	EML5	ENSG00000165521	0.962
hsa-miR-32-5p	ZNF804A	ENSG00000170396	0.961
hsa-miR-374a-5p	SMAD6	ENSG00000137834	0.961
hsa-miR-129-5p	SIAH1	ENSG00000196470	0.961
hsa-miR-1228-3p	RBFOX3	ENSG00000167281	0.961
hsa-miR-193b-3p	KLHL15	ENSG00000174010	0.961
hsa-miR-3074-5p	EML5	ENSG00000165521	0.961
hsa-miR-513b-5p	ZBTB21	ENSG00000173276	0.96
hsa-miR-548a-5p	SOX5	ENSG00000134532	0.96
hsa-miR-24-3p	RBM11	ENSG00000185272	0.96
hsa-miR-664b-3p	PRLR	ENSG00000113494	0.96
hsa-miR-4659b-5p	NCR3LG1	ENSG00000188211	0.96
hsa-miR-3074-5p	MET	ENSG00000105976	0.96
hsa-miR-548x-5p	KCNJ2	ENSG00000123700	0.96
hsa-miR-548ah-5p	GJA3	ENSG00000121743	0.96
hsa-miR-32-5p	CDKN1C	ENSG00000129757	0.96
hsa-miR-17-5p	SRGAP1	ENSG00000196935	0.959
hsa-miR-590-5p	SOX5	ENSG00000134532	0.959
hsa-miR-9-5p	RANBP17	ENSG00000204764	0.959
hsa-miR-3150a-3p	NCR3LG1	ENSG00000188211	0.959
hsa-miR-301a-3p	DIAPH3	ENSG00000139734	0.959
hsa-miR-625-5p	COL1A1	ENSG00000108821	0.959
hsa-miR-664b-3p	NR4A3	ENSG00000119508	0.958
hsa-miR-9-5p	CCNT1	ENSG00000129315	0.958
hsa-miR-144-3p	TENM1	ENSG00000009694	0.957
hsa-miR-20a-5p	SRGAP1	ENSG00000196935	0.957
hsa-miR-4645-5p	OSM	ENSG00000099985	0.957
hsa-miR-103a-2-5p	IFITM10	ENSG00000244242	0.957
hsa-miR-340-5p	ZBED6	ENSG00000257315	0.956
hsa-miR-93-5p	STBD1	ENSG00000118804	0.956
hsa-miR-1299	RLIM	ENSG00000131263	0.956
hsa-miR-30c-5p	RASGEF1B	ENSG00000138670	0.956
hsa-miR-32-5p	PPP1R9A	ENSG00000158528	0.956
hsa-miR-454-3p	PIGA	ENSG00000165195	0.956
hsa-miR-664b-3p	CD180	ENSG00000134061	0.956
hsa-miR-340-5p	SOX5	ENSG00000134532	0.955
hsa-miR-92a-3p	KLHL15	ENSG00000174010	0.955
hsa-miR-548ah-3p	ARL5B	ENSG00000165997	0.955
hsa-miR-190a-5p	TENM1	ENSG00000009694	0.954
hsa-miR-218-5p	SOX5	ENSG00000134532	0.954
hsa-miR-4645-5p	SOCS1	ENSG00000185338	0.954
hsa-miR-1972	SCARF2	ENSG00000244486	0.954
hsa-miR-513a-5p	GCSAM	ENSG00000174500	0.954
hsa-miR-548ah-5p	DGAT2	ENSG00000062282	0.954

hsa-miR-125b-5p	BORCS6	ENSG00000196544	0.954
hsa-miR-340-5p	ARL5B	ENSG00000165997	0.954
hsa-miR-340-5p	ZNF805	ENSG00000204524	0.953
hsa-miR-129-5p	PPP1R9A	ENSG00000158528	0.953
hsa-miR-548ah-3p	PDK4	ENSG00000004799	0.953
hsa-miR-664a-3p	CXCL8	ENSG00000169429	0.953
hsa-miR-513b-5p	ZBTB43	ENSG00000169155	0.952
hsa-miR-664a-3p	RASGRF1	ENSG00000058335	0.952
hsa-miR-340-5p	PRLR	ENSG00000113494	0.952
hsa-miR-1303	MANSC1	ENSG00000111261	0.952
hsa-miR-590-5p	FASLG	ENSG00000117560	0.952
hsa-miR-153-3p	CFL2	ENSG00000165410	0.952
hsa-miR-548a-5p	RASGEF1B	ENSG00000138670	0.951
hsa-miR-548a-5p	PDE4D	ENSG00000113448	0.951
hsa-miR-506-3p	JAM2	ENSG00000154721	0.951
hsa-miR-21-5p	DUSP8	ENSG00000184545	0.951
hsa-miR-340-5p	DBF4	ENSG00000006634	0.951
hsa-miR-1299	CCDC173	ENSG00000154479	0.951
hsa-miR-548a-5p	TENM1	ENSG00000009694	0.95
hsa-miR-20a-5p	STBD1	ENSG00000118804	0.95
hsa-miR-548ah-3p	SORCS3	ENSG00000156395	0.95
hsa-miR-15a-5p	BORCS6	ENSG00000196544	0.95
hsa-miR-190b	ADGRE3	ENSG00000131355	0.95
hsa-miR-548a-5p	ADAMTS1	ENSG00000154734	0.95
hsa-miR-371b-5p	ZNF805	ENSG00000204524	0.949
hsa-miR-4494	SRGAP1	ENSG00000196935	0.949
hsa-miR-548ah-3p	RHOB	ENSG00000143878	0.949
hsa-miR-506-3p	LRFN1	ENSG00000128011	0.949
hsa-miR-15b-5p	KCNJ2	ENSG00000123700	0.949
hsa-miR-15b-5p	BORCS6	ENSG00000196544	0.949
hsa-miR-340-5p	TNFAIP3	ENSG00000118503	0.948
hsa-miR-664b-3p	TMEM255A	ENSG00000125355	0.948
hsa-miR-548ah-3p	RLIM	ENSG00000131263	0.948
hsa-miR-199a-5p	P4HA2	ENSG00000072682	0.948
hsa-miR-548ah-3p	SLC1A2	ENSG00000110436	0.947
hsa-miR-30c-5p	PDE4D	ENSG00000113448	0.947
hsa-miR-129-5p	NR4A2	ENSG00000153234	0.947
hsa-miR-124-3p	JAM2	ENSG00000154721	0.947
hsa-miR-4775	ARHGEF10	ENSG00000104728	0.947
hsa-miR-4775	TM4SF19	ENSG00000145107	0.946
hsa-miR-10a-5p	RLIM	ENSG00000131263	0.946
hsa-miR-513b-5p	PGAP1	ENSG00000197121	0.946
hsa-miR-103a-3p	PDK4	ENSG00000004799	0.946
hsa-miR-513a-5p	CD83	ENSG00000112149	0.946
hsa-miR-340-5p	BTG3	ENSG00000154640	0.946
hsa-miR-93-5p	SRGAP1	ENSG00000196935	0.945
hsa-miR-548ah-3p	SIAH1	ENSG00000196470	0.945

hsa-miR-548ai	PPP1R9A	ENSG00000158528	0.945
hsa-miR-92a-3p	NR4A3	ENSG00000119508	0.945
hsa-miR-301a-3p	MASTL	ENSG00000120539	0.945
hsa-miR-21-5p	LRRIQ3	ENSG00000162620	0.945
hsa-miR-15a-5p	KCNJ2	ENSG00000123700	0.945
hsa-miR-1299	GJA3	ENSG00000121743	0.945
hsa-miR-4775	TMEM170B	ENSG00000205269	0.944
hsa-miR-664a-3p	SLC1A2	ENSG00000110436	0.944
hsa-miR-17-3p	RLIM	ENSG00000131263	0.944
hsa-miR-129-5p	PIK3R1	ENSG00000145675	0.944
hsa-miR-1299	NDUFV2	ENSG00000178127	0.944
hsa-miR-17-5p	STBD1	ENSG00000118804	0.943
hsa-miR-3605-5p	RNF152	ENSG00000176641	0.943
hsa-miR-20a-5p	RLIM	ENSG00000131263	0.943
hsa-miR-92a-3p	SERTAD2	ENSG00000179833	0.942
hsa-miR-10a-5p	SDC1	ENSG00000115884	0.942
hsa-miR-551b-5p	PRLR	ENSG00000113494	0.942
hsa-miR-204-5p	PPP1R9A	ENSG00000158528	0.942
hsa-miR-199b-5p	P4HA2	ENSG00000072682	0.942
hsa-miR-374a-5p	NEXMIF	ENSG00000050030	0.942
hsa-miR-625-5p	CX3CR1	ENSG00000168329	0.942
hsa-miR-129-5p	SLC1A2	ENSG00000110436	0.941
hsa-miR-548ah-3p	PIK3R1	ENSG00000145675	0.941
hsa-miR-4423-5p	GIMAP1	ENSG00000213203	0.941
hsa-miR-15a-5p	RLIM	ENSG00000131263	0.94
hsa-miR-4775	LRRIQ3	ENSG00000162620	0.94
hsa-miR-196a-5p	KCNJ2	ENSG00000123700	0.94
hsa-miR-340-5p	FEM1C	ENSG00000145780	0.94
hsa-miR-432-5p	RASGEF1B	ENSG00000138670	0.939
hsa-miR-340-5p	MET	ENSG00000105976	0.939
hsa-miR-432-5p	E2F8	ENSG00000129173	0.939
hsa-miR-190a-5p	ADGRE3	ENSG00000131355	0.938
hsa-miR-4645-5p	IFITM10	ENSG00000244242	0.937
hsa-miR-551b-5p	TMEM170B	ENSG00000205269	0.936
hsa-miR-548ah-5p	PGAP1	ENSG00000197121	0.936
hsa-miR-15a-5p	NCR3LG1	ENSG00000188211	0.936
hsa-miR-3150a-3p	COL1A1	ENSG00000108821	0.936
hsa-miR-513a-5p	ACKR4	ENSG00000129048	0.936
hsa-miR-432-5p	CD83	ENSG00000112149	0.935
hsa-miR-103a-3p	BTN1A1	ENSG00000124557	0.935
hsa-miR-513b-5p	RANBP17	ENSG00000204764	0.934
hsa-miR-4775	KLHL15	ENSG00000174010	0.934
hsa-miR-374a-5p	HES1	ENSG00000114315	0.934
hsa-miR-24-3p	CISH	ENSG00000114737	0.934
hsa-miR-1299	CACNA1E	ENSG00000198216	0.934
hsa-miR-190b	TENM1	ENSG00000009694	0.933
hsa-miR-93-5p	SERTAD2	ENSG00000179833	0.933

hsa-miR-340-5p	NR1D2	ENSG00000174738	0.933
hsa-miR-548ai	NR1D2	ENSG00000174738	0.933
hsa-miR-3144-5p	COL1A1	ENSG00000108821	0.933
hsa-miR-340-5p	TLR10	ENSG00000174123	0.932
hsa-miR-539-5p	TENM1	ENSG00000009694	0.932
hsa-miR-15b-5p	RLIM	ENSG00000131263	0.932
hsa-miR-340-5p	NR4A2	ENSG00000153234	0.932
hsa-miR-15b-5p	NCR3LG1	ENSG00000188211	0.932
hsa-miR-548ah-3p	CCSER1	ENSG00000184305	0.932
hsa-miR-4717-5p	TNFAIP3	ENSG00000118503	0.931
hsa-miR-616-5p	SERTAD2	ENSG00000179833	0.931
hsa-miR-4775	PRLR	ENSG00000113494	0.931
hsa-miR-548ah-3p	HEY2	ENSG00000135547	0.931
hsa-miR-548ah-5p	GIMAP1	ENSG00000213203	0.931
hsa-miR-4659b-5p	CCSER1	ENSG00000184305	0.931
hsa-miR-513b-5p	ZNF804A	ENSG00000170396	0.93
hsa-miR-506-3p	TNFSF11	ENSG00000120659	0.93
hsa-miR-628-3p	LINGO2	ENSG00000174482	0.93
hsa-miR-432-5p	DEPDC1	ENSG00000024526	0.93
hsa-miR-664a-3p	CFL2	ENSG00000165410	0.93
hsa-miR-3150a-3p	CCR6	ENSG00000112486	0.93
hsa-miR-371b-5p	BTG3	ENSG00000154640	0.93
hsa-miR-4717-5p	ATP1B2	ENSG00000129244	0.93
hsa-miR-454-3p	TMEM250	ENSG00000238227	0.929
hsa-miR-449a	TENM1	ENSG00000009694	0.929
hsa-miR-92b-5p	SLC7A5	ENSG00000103257	0.929
hsa-miR-9-5p	PRLR	ENSG00000113494	0.929
hsa-miR-3150a-3p	PER1	ENSG00000179094	0.929
hsa-miR-1299	MCOLN3	ENSG00000055732	0.929
hsa-miR-548s	SCARF2	ENSG00000244486	0.928
hsa-miR-708-5p	PKD2L2	ENSG00000078795	0.928
hsa-miR-24-3p	NCR3LG1	ENSG00000188211	0.928
hsa-miR-548x-5p	FAM217A	ENSG00000145975	0.927
hsa-miR-92a-3p	AURKA	ENSG00000087586	0.927
hsa-miR-92a-3p	ADRB1	ENSG00000043591	0.927
hsa-miR-193a-5p	ZNF628	ENSG00000197483	0.926
hsa-miR-196a-5p	PFKFB3	ENSG00000170525	0.926
hsa-miR-371a-3p	NCR3LG1	ENSG00000188211	0.926
hsa-miR-20a-5p	KLHL15	ENSG00000174010	0.926
hsa-miR-24-3p	FASLG	ENSG00000117560	0.926
hsa-miR-93-5p	ARHGEF10	ENSG00000104728	0.926
hsa-miR-4717-5p	YES1	ENSG00000176105	0.925
hsa-miR-21-5p	SC5D	ENSG00000109929	0.925
hsa-miR-548ah-3p	PIGA	ENSG00000165195	0.925
hsa-miR-548s	NCR3LG1	ENSG00000188211	0.925
hsa-miR-365a-3p	KCNJ2	ENSG00000123700	0.925
hsa-miR-548x-5p	FAM43A	ENSG00000185112	0.925

hsa-miR-103a-3p	CDKN1C	ENSG00000129757	0.925
hsa-miR-204-5p	ADGRE3	ENSG00000131355	0.925
hsa-miR-513a-5p	ZBTB10	ENSG00000205189	0.924
hsa-miR-548ah-3p	ZBTB10	ENSG00000205189	0.924
hsa-miR-30c-5p	SLC1A2	ENSG00000110436	0.924
hsa-miR-17-5p	SERTAD2	ENSG00000179833	0.924
hsa-miR-4775	PDE4D	ENSG00000113448	0.924
hsa-miR-664b-3p	RASGEF1B	ENSG00000138670	0.923
hsa-miR-10a-5p	PRLR	ENSG00000113494	0.923
hsa-miR-129-5p	PGAP1	ENSG00000197121	0.923
hsa-miR-548a-5p	PGAP1	ENSG00000197121	0.923
hsa-miR-513a-5p	BTN1A1	ENSG00000124557	0.923
hsa-miR-98-5p	ARHGAP8	ENSG00000241484	0.923
hsa-miR-193b-3p	KCNJ2	ENSG00000123700	0.922
hsa-miR-4659b-5p	KCNJ2	ENSG00000123700	0.922
hsa-miR-32-5p	IFIT2	ENSG00000119922	0.922
hsa-miR-129-5p	IER5L	ENSG00000188483	0.922
hsa-miR-30c-5p	GCSAM	ENSG00000174500	0.922
hsa-miR-92a-3p	CHST7	ENSG00000147119	0.922
hsa-miR-504-5p	YES1	ENSG00000176105	0.921
hsa-miR-500a-3p	TMEM170B	ENSG00000205269	0.921
hsa-miR-144-3p	RASGRF1	ENSG00000058335	0.921
hsa-miR-490-3p	AURKA	ENSG00000087586	0.921
hsa-miR-30a-3p	TENM1	ENSG00000009694	0.92
hsa-miR-548x-5p	RBM11	ENSG00000185272	0.92
hsa-miR-124-5p	RANBP17	ENSG00000204764	0.92
hsa-miR-98-5p	NCR3LG1	ENSG00000188211	0.92
hsa-miR-3180	JUND	ENSG00000130522	0.92
hsa-miR-1299	ZNF285	ENSG00000267508	0.919
hsa-miR-196a-5p	TKTL2	ENSG00000151005	0.919
hsa-miR-551b-5p	KLHL15	ENSG00000174010	0.919
hsa-miR-574-5p	C3orf35	ENSG00000198590	0.919
hsa-miR-513a-5p	TMEM170B	ENSG00000205269	0.918
hsa-miR-340-5p	SERTAD2	ENSG00000179833	0.918
hsa-miR-454-3p	MASTL	ENSG00000120539	0.918
hsa-miR-153-3p	IDO1	ENSG00000131203	0.918
hsa-miR-153-3p	SCML1	ENSG00000047634	0.917
hsa-miR-885-5p	DEPDC1	ENSG00000024526	0.917
hsa-miR-548a-5p	ARL5B	ENSG00000165997	0.917
hsa-miR-32-5p	ARHGEF10	ENSG00000104728	0.917
hsa-miR-103a-3p	ZBTB10	ENSG00000205189	0.916
hsa-miR-548ah-3p	TMEM170B	ENSG00000205269	0.916
hsa-miR-153-3p	PIGA	ENSG00000165195	0.916
hsa-miR-3150a-3p	NETO1	ENSG00000166342	0.916
hsa-miR-1299	KCNJ2	ENSG00000123700	0.916
hsa-miR-98-5p	ERRFI1	ENSG00000116285	0.916
hsa-miR-664a-3p	YOD1	ENSG00000180667	0.915

hsa-miR-502-5p	TNFRSF9	ENSG00000049249	0.915
hsa-miR-147a	TIPARP	ENSG00000163659	0.915
hsa-miR-17-3p	TENM1	ENSG00000009694	0.915
hsa-miR-539-5p	SYN1	ENSG00000008056	0.915
hsa-miR-624-5p	SOX5	ENSG00000134532	0.915
hsa-miR-199a-5p	RASGEF1B	ENSG00000138670	0.915
hsa-miR-513a-5p	PPP1R9A	ENSG00000158528	0.915
hsa-miR-4645-5p	NCR3LG1	ENSG00000188211	0.915
hsa-miR-4775	DBF4	ENSG00000006634	0.915
hsa-miR-362-5p	ZNF805	ENSG00000204524	0.914
hsa-miR-628-5p	SH3D19	ENSG00000109686	0.914
hsa-miR-548ah-5p	SERTAD2	ENSG00000179833	0.914
hsa-miR-628-5p	ERRFI1	ENSG00000116285	0.914
hsa-miR-101-3p	ATP1B2	ENSG00000129244	0.914
hsa-miR-124-5p	TMEM170B	ENSG00000205269	0.913
hsa-miR-204-5p	STEAP4	ENSG00000127954	0.913
hsa-miR-301a-3p	STEAP4	ENSG00000127954	0.913
hsa-miR-3064-5p	SLC23A3	ENSG00000213901	0.913
hsa-miR-362-5p	SERTAD2	ENSG00000179833	0.913
hsa-miR-1303	RNF152	ENSG00000176641	0.913
hsa-miR-101-3p	PRLR	ENSG00000113494	0.913
hsa-miR-625-5p	PRLR	ENSG00000113494	0.913
hsa-miR-374a-5p	TNFAIP3	ENSG00000118503	0.912
hsa-miR-548a-5p	NR4A3	ENSG00000119508	0.912
hsa-miR-340-5p	KCNJ2	ENSG00000123700	0.912
hsa-miR-664a-3p	FAM81B	ENSG00000153347	0.912
hsa-miR-125b-5p	CTU1	ENSG00000142544	0.912
hsa-miR-616-5p	CFL2	ENSG00000165410	0.912
hsa-miR-3074-3p	ZNF804A	ENSG00000170396	0.911
hsa-miR-340-5p	STEAP4	ENSG00000127954	0.911
hsa-miR-153-3p	SLC26A5	ENSG00000170615	0.911
hsa-miR-660-5p	KCNJ2	ENSG00000123700	0.911
hsa-miR-664b-3p	DIAPH3	ENSG00000139734	0.911
hsa-miR-362-5p	CCSER1	ENSG00000184305	0.911
hsa-miR-15a-5p	CACNA1E	ENSG00000198216	0.911
hsa-miR-30c-5p	C4orf19	ENSG00000154274	0.911
hsa-miR-574-5p	BATF2	ENSG00000168062	0.911
hsa-miR-24-3p	RANBP17	ENSG00000204764	0.91
hsa-miR-664b-3p	NEXMIF	ENSG00000050030	0.91
hsa-miR-122-5p	BEND5	ENSG00000162373	0.91
hsa-miR-664b-3p	PRICKLE2	ENSG00000163637	0.909
hsa-miR-539-5p	GALNT5	ENSG00000136542	0.909
hsa-miR-432-5p	CCDC74A	ENSG00000163040	0.909
hsa-miR-548ah-3p	BIRC5	ENSG00000089685	0.909
hsa-miR-4775	YES1	ENSG00000176105	0.908
hsa-miR-574-5p	TCTE1	ENSG00000146221	0.908
hsa-miR-548ah-5p	RLIM	ENSG00000131263	0.908

hsa-miR-3074-5p	RGPD6	ENSG00000183054	0.908
hsa-miR-193a-3p	EML5	ENSG00000165521	0.908
hsa-miR-548a-5p	CFL2	ENSG00000165410	0.908
hsa-miR-17-5p	ARHGEF10	ENSG00000104728	0.908
hsa-miR-9-5p	ZBTB21	ENSG00000173276	0.907
hsa-miR-193a-3p	KLHL15	ENSG00000174010	0.907
hsa-miR-548a-5p	HES1	ENSG00000114315	0.907
hsa-miR-664b-3p	GCSAML	ENSG00000169224	0.907
hsa-miR-501-5p	DBF4	ENSG00000006634	0.907
hsa-miR-340-5p	YES1	ENSG00000176105	0.906
hsa-miR-432-5p	SYN1	ENSG00000008056	0.906
hsa-miR-374a-5p	STEAP4	ENSG00000127954	0.906
hsa-miR-3180-5p	SRGAP1	ENSG00000196935	0.906
hsa-miR-454-3p	NEXMIF	ENSG00000050030	0.906
hsa-miR-93-5p	FEM1C	ENSG00000145780	0.906
hsa-miR-187-5p	CCDC173	ENSG00000154479	0.906
hsa-miR-15b-5p	CACNA1E	ENSG00000198216	0.906
hsa-miR-374a-5p	ARL5B	ENSG00000165997	0.906
hsa-miR-301a-3p	SIK1	ENSG00000142178	0.905
hsa-miR-129-5p	SC5D	ENSG00000109929	0.905
hsa-miR-548ah-3p	ZNF626	ENSG00000188171	0.904
hsa-miR-500a-3p	RHBDF1	ENSG00000007384	0.904
hsa-miR-9-5p	PGAP1	ENSG00000197121	0.904
hsa-miR-122-5p	MIXL1	ENSG00000185155	0.904
hsa-miR-3176	KCNJ2	ENSG00000123700	0.904
hsa-miR-513b-5p	FEM1C	ENSG00000145780	0.904
hsa-miR-144-3p	ZNF804A	ENSG00000170396	0.903
hsa-miR-3074-5p	U2AF1	ENSG00000160201	0.903
hsa-miR-548ah-5p	TMEM170B	ENSG00000205269	0.903
hsa-miR-92a-3p	SMIM11A	ENSG00000205670	0.903
hsa-miR-664b-3p	SCN3B	ENSG00000166257	0.903
hsa-miR-124-3p	LRFN1	ENSG00000128011	0.903
hsa-miR-1303	JAM2	ENSG00000154721	0.903
hsa-miR-449a	EML5	ENSG00000165521	0.903
hsa-miR-548x-5p	NR1D2	ENSG00000174738	0.902
hsa-miR-124-3p	KCNJ2	ENSG00000123700	0.902
hsa-miR-32-5p	ADRB1	ENSG00000043591	0.902
hsa-miR-4775	ZBTB43	ENSG00000169155	0.901
hsa-miR-432-5p	NCR3LG1	ENSG00000188211	0.901
hsa-miR-103a-3p	BORCS6	ENSG00000196544	0.901
hsa-miR-101-3p	ZNF804A	ENSG00000170396	0.9
hsa-miR-103a-2-5p	SOX5	ENSG00000134532	0.9
hsa-miR-147a	SMAD9	ENSG00000120693	0.9
hsa-miR-3150a-3p	SLC1A2	ENSG00000110436	0.9
hsa-miR-9-5p	SIK1	ENSG00000142178	0.9
hsa-miR-1303	SCN3B	ENSG00000166257	0.9
hsa-miR-548a-5p	RLIM	ENSG00000131263	0.9

hsa-miR-548ah-3p	PGAP1	ENSG00000197121	0.9
hsa-miR-574-5p	NCR3LG1	ENSG00000188211	0.9
hsa-miR-500a-5p	KLHL15	ENSG00000174010	0.9
hsa-miR-371b-5p	GCSAML	ENSG00000169224	0.9
hsa-miR-4775	GCSAML	ENSG00000169224	0.9
hsa-miR-548a-5p	BTG3	ENSG00000154640	0.9
hsa-miR-490-3p	SLC26A5	ENSG00000170615	0.899
hsa-miR-374a-5p	PDE4D	ENSG00000113448	0.899
hsa-miR-129-5p	LRRIQ3	ENSG00000162620	0.899
hsa-miR-129-5p	LINGO2	ENSG00000174482	0.899
hsa-miR-625-5p	OTX1	ENSG00000115507	0.898
hsa-miR-340-5p	NR4A3	ENSG00000119508	0.898
hsa-miR-624-5p	CNDP1	ENSG00000150656	0.898
hsa-miR-548ah-3p	RNF152	ENSG00000176641	0.897
hsa-miR-3064-5p	NR4A3	ENSG00000119508	0.897
hsa-miR-144-5p	NCR3LG1	ENSG00000188211	0.897
hsa-miR-32-5p	TMEM255A	ENSG00000125355	0.896
hsa-miR-144-3p	PRLR	ENSG00000113494	0.896
hsa-miR-449a	NR4A2	ENSG00000153234	0.896
hsa-miR-513a-5p	CEP19	ENSG00000174007	0.896
hsa-miR-124-3p	SRGAP1	ENSG00000196935	0.895
hsa-miR-103a-2-5p	RLIM	ENSG00000131263	0.895
hsa-miR-3150a-3p	PRLR	ENSG00000113494	0.895
hsa-miR-20a-5p	PGAP1	ENSG00000197121	0.895
hsa-miR-144-3p	NEXMIF	ENSG00000050030	0.895
hsa-miR-449a	MBLAC1	ENSG00000214309	0.895
hsa-miR-454-3p	KRT23	ENSG00000108244	0.895
hsa-miR-616-5p	GCSAML	ENSG00000169224	0.895
hsa-miR-432-5p	CACNA1E	ENSG00000198216	0.895
hsa-miR-340-5p	ASCL2	ENSG00000183734	0.895
hsa-miR-548ah-3p	ZBTB21	ENSG00000173276	0.894
hsa-miR-548a-5p	TMEM255A	ENSG00000125355	0.894
hsa-miR-548ai	SORCS3	ENSG00000156395	0.894
hsa-miR-502-5p	RGS20	ENSG00000147509	0.894
hsa-miR-548ah-3p	HJURP	ENSG00000123485	0.894
hsa-miR-153-3p	FEM1C	ENSG00000145780	0.894
hsa-miR-374a-5p	DEPDC1	ENSG00000024526	0.894
hsa-miR-449a	CNIH2	ENSG00000174871	0.894
hsa-miR-4659b-5p	CFL2	ENSG00000165410	0.894
hsa-miR-92a-3p	ARHGEF10	ENSG00000104728	0.894
hsa-miR-506-3p	SRGAP1	ENSG00000196935	0.893
hsa-miR-616-5p	SKA3	ENSG00000165480	0.893
hsa-miR-513a-5p	RNF152	ENSG00000176641	0.893
hsa-miR-93-5p	RBM34	ENSG00000188739	0.893
hsa-miR-32-5p	CHST7	ENSG00000147119	0.893
hsa-miR-548a-5p	CAV1	ENSG00000105974	0.893
hsa-miR-101-3p	SMAD9	ENSG00000120693	0.892

hsa-miR-3074-3p	NCR3LG1	ENSG00000188211	0.892
hsa-miR-206	MET	ENSG00000105976	0.892
hsa-miR-144-3p	DUSP1	ENSG00000120129	0.892
hsa-miR-365b-5p	CACNA1E	ENSG00000198216	0.892
hsa-miR-454-3p	STEAP4	ENSG00000127954	0.891
hsa-miR-502-5p	SLC26A5	ENSG00000170615	0.891
hsa-miR-625-5p	SIAH1	ENSG00000196470	0.891
hsa-miR-548a-5p	PPP1R9A	ENSG00000158528	0.891
hsa-miR-628-5p	NAT2	ENSG00000156006	0.891
hsa-miR-340-5p	IL1RL1	ENSG00000115602	0.891

ST_11	core_module_miRNA/mRNA_pairs	
miRNA	mRNA	Pair topological strenghth
hsa-miR-17-5p	KLHL15	0.105743161
hsa-miR-17-5p	YOD1	0.100359822
hsa-miR-20a-5p	KLHL15	0.148000196
hsa-miR-20a-5p	YOD1	0.142616857
hsa-miR-20a-5p	SERTAD2	0.130189084
hsa-miR-20a-5p	FEM1C	0.119796518
hsa-miR-20a-5p	RLIM	0.118487026
hsa-miR-20a-5p	CFL2	0.118310773
hsa-miR-20a-5p	SRGAP1	0.116774381
hsa-miR-20a-5p	SMAD6	0.115853599
hsa-miR-20a-5p	YES1	0.115469898
hsa-miR-20a-5p	NR4A3	0.114279156
hsa-miR-20a-5p	ZNF805	0.113425637
hsa-miR-20a-5p	SIK1	0.113101764
hsa-miR-20a-5p	ZBTB21	0.10985332
hsa-miR-20a-5p	BTG3	0.109323074
hsa-miR-20a-5p	PIK3R1	0.108726344
hsa-miR-20a-5p	NR4A2	0.108673034
hsa-miR-20a-5p	RORB	0.108365443
hsa-miR-20a-5p	HIF1A	0.108346978
hsa-miR-20a-5p	ZBTB43	0.107821124
hsa-miR-20a-5p	ARHGEF10	0.107241302
hsa-miR-20a-5p	TENM1	0.106949835
hsa-miR-20a-5p	MASTL	0.10565161
hsa-miR-20a-5p	NCR3LG1	0.10534803
hsa-miR-20a-5p	DUSP8	0.105282545
hsa-miR-20a-5p	SLC1A2	0.104938134
hsa-miR-20a-5p	SKOR1	0.104646449
hsa-miR-20a-5p	OSM	0.104511024
hsa-miR-20a-5p	ZNF703	0.103658802
hsa-miR-20a-5p	PER1	0.103303326
hsa-miR-20a-5p	CXCL8	0.103278556
hsa-miR-20a-5p	EREG	0.103187794
hsa-miR-20a-5p	TMEM255A	0.103181868
hsa-miR-20a-5p	CHST7	0.103001589
hsa-miR-20a-5p	PGAP1	0.102708839
hsa-miR-20a-5p	CAV1	0.102505147
hsa-miR-20a-5p	TIPARP	0.102443381
hsa-miR-20a-5p	C14orf119	0.102369481
hsa-miR-20a-5p	MIXL1	0.10236398
hsa-miR-20a-5p	PKMYT1	0.1022111
hsa-miR-20a-5p	AHRR	0.1021693
hsa-miR-20a-5p	TNFSF11	0.102064244
hsa-miR-20a-5p	STBD1	0.101916411
hsa-miR-20a-5p	SYNM	0.101876067
hsa-miR-20a-5p	PANX2	0.101810365
hsa-miR-20a-5p	PFKFB3	0.101763291
hsa-miR-20a-5p	RBM34	0.101750275

hsa-miR-20a-5p	UBE2C	0.101606667
hsa-miR-20a-5p	FZD7	0.101293506
hsa-miR-20a-5p	GJA3	0.101021205
hsa-miR-20a-5p	ZNF331	0.100867978
hsa-miR-20a-5p	ZNF484	0.100818254
hsa-miR-340-5p	KLHL15	0.13499804
hsa-miR-340-5p	YOD1	0.129614701
hsa-miR-340-5p	SERTAD2	0.117186927
hsa-miR-340-5p	FEM1C	0.106794361
hsa-miR-340-5p	RLIM	0.10548487
hsa-miR-340-5p	CFL2	0.105308617
hsa-miR-340-5p	SRGAP1	0.103772225
hsa-miR-340-5p	YES1	0.102467742
hsa-miR-340-5p	ZBTB10	0.102274267
hsa-miR-340-5p	NR4A3	0.101277
hsa-miR-340-5p	ZNF805	0.100423481
hsa-miR-340-5p	ZBTB21	0.096851163
hsa-miR-340-5p	BTG3	0.096320918
hsa-miR-340-5p	ARL5B	0.095777545
hsa-miR-340-5p	NR4A2	0.095670878
hsa-miR-340-5p	RORB	0.095363287
hsa-miR-340-5p	HIF1A	0.095344822
hsa-miR-340-5p	ZBTB43	0.094818968
hsa-miR-340-5p	PDE4D	0.094582884
hsa-miR-340-5p	NCR3LG1	0.092345873
hsa-miR-340-5p	TMEM170B	0.092090537
hsa-miR-340-5p	SLC1A2	0.091935978
hsa-miR-340-5p	OSM	0.091508867
hsa-miR-340-5p	SOX5	0.091398631
hsa-miR-340-5p	DBF4	0.091149017
hsa-miR-340-5p	PRLR	0.090934029
hsa-miR-340-5p	NEXMIF	0.090905969
hsa-miR-340-5p	MET	0.090243364
hsa-miR-340-5p	NR1D2	0.089947188
hsa-miR-340-5p	NRARP	0.089921078
hsa-miR-340-5p	REL	0.089753627
hsa-miR-340-5p	PDK4	0.089734438
hsa-miR-340-5p	PGAP1	0.089706683
hsa-miR-340-5p	GFPT2	0.089662916
hsa-miR-340-5p	TNFAIP3	0.089631712
hsa-miR-340-5p	ARHGAP29	0.089558009
hsa-miR-340-5p	SIAH1	0.089454939
hsa-miR-340-5p	RNF152	0.089454423
hsa-miR-340-5p	KCNJ2	0.089388262
hsa-miR-340-5p	DEPDC1	0.08899522
hsa-miR-340-5p	CCNT1	0.088930949
hsa-miR-340-5p	CCSER1	0.088885144
hsa-miR-340-5p	ADAMTS1	0.088619328
hsa-miR-340-5p	STEAP4	0.088561588
hsa-miR-93-5p	KLHL15	0.109947085

hsa-miR-93-5p	YOD1	0.104563746

ST_12	pathways_both_mRNA			
Term	Description	Functional category	P value	Density
R-HSA-2428933	SHC-related events triggered by IGF1R	Apoptosis	0.0272	0.02499
R-HSA-9617629	Regulation of FOXO transcriptional activity by acetylation	Apoptosis	0.0019	0.05714
R-HSA-9614657	FOXO-mediated transcription of cell death genes	Apoptosis	0.0133	0.01666
R-HSA-389948	PD-1 signaling	Apoptosis	0.0132	0.00714
R-HSA-9614085	FOXO-mediated transcription	Apoptosis	0.004	0.01621
R-HSA-2122947	NOTCH1 Intracellular Domain Regulates Transcription	Apoptosis	0.0241	0.02692
R-HSA-9013695	NOTCH4 Intracellular Domain Regulates Transcription	Apoptosis	0.0434	0.02058
R-HSA-9013508	NOTCH3 Intracellular Domain Regulates Transcription	Apoptosis	0.0067	0.02368
R-HSA-9013694	Signaling by NOTCH4	Apoptosis	0.0381	0.02105
R-HSA-1980143	Signaling by NOTCH1	Apoptosis	0.0306	0.02045
R-HSA-9012852	Signaling by NOTCH3	Apoptosis	0.0256	0.01447
R-HSA-1912408	Pre-NOTCH Transcription and Translation	Apoptosis	0.0131	0.0119
R-HSA-1912422	Pre-NOTCH Expression and Processing	Apoptosis	0.0132	0.01136
R-HSA-8941856	RUNX3 regulates Notch signaling	Apoptosis	0.0215	0.02499
R-HSA-8951936	RUNX3 regulates p14-ARF	Apoptosis	0.0406	0.025
R-HSA-8940973	RUNX2 regulates osteoblast differentiation	Development	0.0087	0.025
R-HSA-2892247	POU5F1 (OCT4), SOX2, NANOG activate genes related to proliferation	Development	2.00E-04	0.02692
R-HSA-525793	Myogenesis	Development	0.04	0.01111
R-HSA-452723	Transcriptional regulation of pluripotent stem cells	Development	0	0.01451
R-HSA-8853659	RET signaling	Development	0.0025	0.01219
R-HSA-5362517	Signaling by Retinoic Acid	Development	0	0.01744
R-HSA-9006115	Signaling by NTRK2 (TRKB)	Development	0.0302	0.02
R-HSA-8866907	Activation of the TFAP2 (AP-2) family of transcription factors	Development	0.0023	0.02499
R-HSA-8864260	Transcriptional regulation by the AP-2 (TFAP2) family of transcription factors	Development	0.0406	0.00945
R-HSA-162791	Attachment of GPI anchor to uPAR	Development	0.0166	0.01428
R-HSA-167044	Signalling to RAS	Development	0.048	0.01538
R-HSA-1643713	Signaling by EGFR in Cancer	Fibrosis	0.0122	0.016
R-HSA-177929	Signaling by EGFR	Fibrosis	0.0058	0.01276
R-HSA-9665348	Signaling by ERBB2 ECD mutants	Fibrosis	0.0069	0.01875
R-HSA-9665686	Signaling by ERBB2 TMD/JMD mutants	Fibrosis	0.0197	0.01136
R-HSA-9664565	Signaling by ERBB2 KD Mutants	Fibrosis	0.0088	0.012
R-HSA-1227990	Signaling by ERBB2 in Cancer	Fibrosis	0.0102	0.01153
R-HSA-1227986	Signaling by ERBB2	Fibrosis	0.0131	0.01326

R-HSA-1236394	Signaling by ERBB4	Fibrosis	0.0359	0.00918
R-HSA-9634285	Constitutive Signaling by Overexpressed ERBB2	Fibrosis	0.0137	0.01818
R-HSA-1250347	SHC1 events in ERBB4 signaling	Fibrosis	0.0421	0.01071
R-HSA-5637812	Signaling by EGFRvIII in Cancer	Fibrosis	0.0058	0.02
R-HSA-5637810	Constitutive Signaling by EGFRvIII	Fibrosis	0.0066	0.02
R-HSA-5637815	Signaling by Ligand-Responsive EGFR Variants in Cancer	Fibrosis	0.011	0.02105
R-HSA-1236382	Constitutive Signaling by Ligand-Responsive EGFR Cancer Variants	Fibrosis	0.0121	0.02105
R-HSA-912631	Regulation of signaling by CBL	Fibrosis	0.0054	0.02619
R-HSA-8853334	Signaling by FGFR3 fusions in cancer	Fibrosis	0.0392	0.015
R-HSA-5655291	Signaling by FGFR4 in disease	Fibrosis	0.0149	0.01818
R-HSA-8851805	MET activates RAS signaling	Fibrosis	0.0331	0.01363
R-HSA-1433559	Regulation of KIT signaling	Fibrosis	0.0288	0.02499
R-HSA-180292	GAB1 signalosome	Fibrosis	0.0327	0.0147
R-HSA-1839117	Signaling by cytosolic FGFR1 fusion mutants	Fibrosis	6.00E-04	0.02222
R-HSA-5654704	SHC-mediated cascade:FGFR3	Fibrosis	0.0401	0.00833
R-HSA-5654719	SHC-mediated cascade:FGFR4	Fibrosis	0.0454	0.0075
R-HSA-9670439	Signaling by phosphorylated juxtamembrane, extracellular and kinase domain KIT mutants	Fibrosis	1.00E-04	0.03499
R-HSA-9669938	Signaling by KIT in disease	Fibrosis	2.00E-04	0.03499
R-HSA-5654688	SHC-mediated cascade:FGFR1	Fibrosis	0.0127	0.00952
R-HSA-5654689	PI-3K cascade:FGFR1	Fibrosis	0.016	0.00952
R-HSA-5655332	Signaling by FGFR3 in disease	Fibrosis	0.026	0.00909
R-HSA-8853338	Signaling by FGFR3 point mutants in cancer	Fibrosis	0.0266	0.00909
R-HSA-5654693	FRS-mediated FGFR1 signaling	Fibrosis	0.0167	0.00869
R-HSA-5654708	Downstream signaling of activated FGFR3	Fibrosis	0.0055	0.012
R-HSA-5654716	Downstream signaling of activated FGFR4	Fibrosis	0.0048	0.01111
R-HSA-5655253	Signaling by FGFR2 in disease	Fibrosis	0.0422	0.0074
R-HSA-5654696	Downstream signaling of activated FGFR2	Fibrosis	0.007	0.01
R-HSA-1839124	FGFR1 mutant receptor activation	Fibrosis	5.00E-04	0.01451
R-HSA-5654687	Downstream signaling of activated FGFR1	Fibrosis	0.0017	0.01129
R-HSA-5655302	Signaling by FGFR1 in disease	Fibrosis	1.00E-04	0.01447
R-HSA-1433557	Signaling by SCF-KIT	Fibrosis	0	0.02682
R-HSA-1226099	Signaling by FGFR in disease	Fibrosis	3.00E-04	0.0117
R-HSA-5654736	Signaling by FGFR1	Fibrosis	0.0352	0.01
R-HSA-8939246	RUNX1 regulates transcription of genes involved in differentiation of myeloid cells	Immune	0.0398	0.02142

R-HSA-210990	PECAM1 interactions	Immune	0.0119	0.03333
R-HSA-918233	TRAF3-dependent IRF activation pathway	Immune	0.0243	0.01785
R-HSA-5621575	CD209 (DC-SIGN) signaling	Immune	0.0034	0.02142
R-HSA-5621480	Dectin-2 family	Immune	0.0369	0.00769
R-HSA-202427	Phosphorylation of CD3 and TCR zeta chains	Immune	0.049	0.00555
R-HSA-2424491	DAP12 signaling	Immune	0	0.02241
R-HSA-2172127	DAP12 interactions	Immune	1.00E-04	0.01477
R-HSA-1368082	RORA activates gene expression	Immune	0	0.04722
R-HSA-9660826	Purinergic signaling in leishmaniasis infection	Immune	0.0161	0.01041
R-HSA-4090294	SUMOylation of intracellular receptors	Inflammation	0	0.05925
R-HSA-912526	Interleukin receptor SHC signaling	Inflammation	3.00E-04	0.01481
R-HSA-350054	Notch-HLH transcription pathway	Inflammation	0.0457	0.01428
R-HSA-448706	Interleukin-1 processing	Inflammation	0.0372	0.01875
R-HSA-8985947	Interleukin-9 signaling	Inflammation	6.00E-04	0.03333
R-HSA-9020933	Interleukin-23 signaling	Inflammation	0.0019	0.02222
R-HSA-9020958	Interleukin-21 signaling	Inflammation	6.00E-04	0.03
R-HSA-9020956	Interleukin-27 signaling	Inflammation	2.00E-04	0.02727
R-HSA-1059683	Interleukin-6 signaling	Inflammation	9.00E-04	0.03181
R-HSA-8984722	Interleukin-35 Signalling	Inflammation	2.00E-04	0.02499
R-HSA-9020558	Interleukin-2 signaling	Inflammation	0.0427	0.01666
R-HSA-8983432	Interleukin-15 signaling	Inflammation	0	0.03076
R-HSA-844456	The NLRP3 inflammasome	Inflammation	0.0278	0.01333
R-HSA-622312	Inflammasomes	Inflammation	0.0472	0.01
R-HSA-9008059	Interleukin-37 signaling	Inflammation	0.0056	0.01428
R-HSA-6783589	Interleukin-6 family signaling	Inflammation	0.001	0.01458
R-HSA-9664424	Cell recruitment (pro-inflammatory response)	Inflammation	0.0175	0.01041
R-HSA-8854691	Interleukin-20 family signaling	Inflammation	2.00E-04	0.01538
R-HSA-451927	Interleukin-2 family signaling	Inflammation	0	0.02023
R-HSA-512988	Interleukin-3, Interleukin-5 and GM-CSF signaling	Inflammation	0	0.02127
R-HSA-1234158	Regulation of gene expression by Hypoxia-inducible Factor	Inflammation	1.00E-04	0.0409
R-HSA-9634600	Regulation of glycolysis by fructose 2,6-bisphosphate Metabolism	Metabolism	0.001	0.025
R-HSA-9031528	NR1H2 & NR1H3 regulate gene expression linked to triglyceride lipolysis in adipose	Metabolism	0	0.08
R-HSA-9031525	NR1H2 & NR1H3 regulate gene expression to limit cholesterol uptake	Metabolism	0	0.08
R-HSA-9632974	NR1H2 & NR1H3 regulate gene expression linked to gluconeogenesis	Metabolism	0	0.06999
R-HSA-433137	Sodium-coupled sulphate, di- and tri-carboxylate transporters	Metabolism	6.00E-04	0.02

R-HSA-9623433	NR1H2 & NR1H3 regulate gene expression to control bile acid homeostasis	Metabolism	0	0.05555
R-HSA-9029558	NR1H2 & NR1H3 regulate gene expression linked to lipogenesis	Metabolism	0	0.05
R-HSA-196791	Vitamin D (calciferol) Metabolism	Metabolism	0.0103	0.01363
R-HSA-1474151	Tetrahydrobiopterin (BH4) synthesis, recycling, salvage and regulation	Metabolism	0.0435	0.01363
R-HSA-200425	Carnitine Metabolism	Metabolism	0.0012	0.01428
R-HSA-193807	Synthesis of bile acids and bile salts via 27-hydroxycholesterol	Metabolism	7.00E-04	0.01666
R-HSA-159418	Recycling of bile acids and salts	Metabolism	0.002	0.0125
R-HSA-204174	Regulation of pyruvate dehydrogenase (PDH) complex	Metabolism	0.0052	0.0125
R-HSA-162710	Synthesis of glycosylphosphatidylinositol (GPI)	Metabolism	0	0.01764
R-HSA-193368	Synthesis of bile acids and bile salts via 7alpha-hydroxycholesterol	Metabolism	0.0019	0.01041
R-HSA-211976	Endogenous sterols	Metabolism	1.00E-04	0.01296
R-HSA-70268	Pyruvate Metabolism	Metabolism	0.0084	0.00689
R-HSA-192105	Synthesis of bile acids and bile salts	Metabolism	0.0019	0.00735
R-HSA-9029569	NR1H3 & NR1H2 regulate gene expression linked to cholesterol transport and efflux	Metabolism	1.00E-04	0.01756
R-HSA-2426168	Activation of gene expression by SREBF (SREBP)	Metabolism	0	0.01428
R-HSA-194068	Bile acid and bile salt Metabolism	Metabolism	0.004	0.00581
R-HSA-9024446	NR1H2 and NR1H3-mediated signaling	Metabolism	0	0.01489
R-HSA-1655829	Regulation of cholesterol biosynthesis by SREBP (SREBF)	Metabolism	0	0.01276
R-HSA-210500	Glutamate Neurotransmitter Release Cycle	Others	8.00E-04	0.01041
R-HSA-1368108	BMAL1:CLOCK,NPAS2 activates circadian gene expression	Others	0.0298	0.01875
R-HSA-3371568	Attenuation phase	Others	4.00E-04	0.01785
R-HSA-3371511	HSF1 activation	Others	0.011	0.01129
R-HSA-400253	Circadian Clock	Others	0	0.03552
R-HSA-3371571	HSF1-dependent transactivation	Others	0.0026	0.01315
R-HSA-3134973	LRR FLII-interacting protein 1 (LRRFIP1) activates type I IFN production	Virus	0.0151	0.06
R-HSA-912694	Regulation of IFNA signaling	Virus	0.013	0.00833
R-HSA-8937144	Aryl hydrocarbon receptor signalling	Virus	0.0048	0.02857
R-HSA-9031628	NGF-stimulated transcription	Wound-healing	0.0172	0.01
R-HSA-9027284	Erythropoietin activates RAS	Wound-healing	0.0043	0.03
R-HSA-9673767	Signaling by PDGFRA transmembrane, juxtamembrane and kinase domain mutants	Wound-healing	0	0.03333

R-HSA-9673770	Signaling by PDGFRA extracellular domain mutants	Wound-healing	1.00E-04	0.03333
R-HSA-9006335	Signaling by Erythropoietin	Wound-healing	0.0042	0.02058
R-HSA-210993	Tie2 Signaling	Wound-healing	0	0.02222
R-HSA-3928664	Ephrin signaling	Wound-healing	0.0304	0.01578
R-HSA-9671555	Signaling by PDGFR in disease	Wound-healing	2.00E-04	0.02
R-HSA-354192	Integrin signaling	Wound-healing	0.0151	0.01481
R-HSA-114604	GPVI-mediated activation cascade	Wound-healing	5.00E-04	0.01911
R-HSA-76009	Platelet Aggregation (Plug Formation)	Wound-healing	0.0229	0.01081
R-HSA-3928662	EPHB-mediated forward signaling	Wound-healing	0.047	0.01282
R-HSA-3928665	EPH-ephrin mediated repulsion of cells	Wound-healing	0.0024	0.011
R-HSA-8849474	PTK6 Activates STAT3	Wound-healing	0.0011	0.05
R-HSA-8849468	PTK6 Regulates Proteins Involved in RNA Processing	Wound-healing	0.0383	0.03
R-HSA-8849473	PTK6 Expression	Wound-healing	1.00E-04	0.05
R-HSA-8849471	PTK6 Regulates RHO GTPases, RAS GTPase and MAP kinases	Wound-healing	0.0428	0.01428

ST_13	pathways_20a5p_mRNA			
Term	Description	Functional category	P value	Density
R-HSA-6806003	Regulation of TP53 Expression and Degradation	Apoptosis	0.0376	0.01422
R-HSA-5673000	RAF activation	Apoptosis	0.0268	0.00877
R-HSA-6804757	Regulation of TP53 Degradation	Apoptosis	0.0374	0.01461
R-HSA-8952158	RUNX3 regulates BCL2L11 (BIM) transcription	Apoptosis	0.0209	0.03157
R-HSA-9013694	Signaling by NOTCH4	Apoptosis	0.0012	0.02216
R-HSA-9013695	NOTCH4 Intracellular Domain Regulates Transcription	Apoptosis	0.0014	0.02476
R-HSA-2660825	Signaling by NOTCH1 t(7;9)(NOTCH1:M1580_K2555) Translocation Mutant	Apoptosis	0.0153	0.02255
R-HSA-2660826	Constitutive Signaling by NOTCH1 t(7;9)(NOTCH1:M1580_K2555) Translocation Mutant	Apoptosis	0.0157	0.02255
R-HSA-5083630	Defective LFNG causes SCDO3	Apoptosis	0.0415	0.02105
R-HSA-6791312	TP53 Regulates Transcription of Cell Cycle Genes	Apoptosis	0.0306	0.01288
R-HSA-6804116	TP53 Regulates Transcription of Genes Involved in G1 Cell Cycle Arrest	Apoptosis	0.0413	0.02255
R-HSA-5627123	RHO GTPases activate PAKs	Apoptosis	0.0033	0.01601
R-HSA-69231	Cyclin D associated events in G1	Cell Cycle	0.0494	0.01343
R-HSA-9675126	Diseases of mitotic cell cycle	Cell Cycle	7.00E-04	0.01754
R-HSA-9687139	Aberrant regulation of mitotic cell cycle due to RB1 defects	Cell Cycle	6.00E-04	0.01754
R-HSA-69273	Cyclin A/B1/B2 associated events during G2/M transition	Cell Cycle	0.0037	0.02526
R-HSA-174048	APC/C:Cdc20 mediated degradation of Cyclin B	Cell Cycle	0.0076	0.01754
R-HSA-141430	Inactivation of APC/C via direct inhibition of the APC/C complex	Cell Cycle	0.0011	0.01754
R-HSA-141405	Inhibition of the proteolytic activity of APC/C required for the onset of anaphase by mitotic spindle checkpoint components	Cell Cycle	7.00E-04	0.01754
R-HSA-176412	Phosphorylation of the APC/C	Cell Cycle	0.0026	0.02105
R-HSA-176407	Conversion from APC/C:Cdc20 to APC/C:Cdh1 in late anaphase	Cell Cycle	1.00E-04	0.01842
R-HSA-9617828	FOXO-mediated transcription of cell cycle genes	Cell Cycle	0.0035	0.02392
R-HSA-5626467	RHO GTPases activate IQGAPs	Cell Cycle	0.0443	0.00619
R-HSA-179409	APC-Cdc20 mediated degradation of Nek2A	Cell Cycle	0.0025	0.01821

R-HSA-9687136	Aberrant regulation of mitotic exit in cancer due to RB1 defects	Cell Cycle	0.0139	0.01315
R-HSA-9659787	Aberrant regulation of mitotic G1/S transition in cancer due to RB1 defects	Cell Cycle	0.0319	0.02167
R-HSA-9661069	Defective binding of RB1 mutants to E2F1,(E2F2, E2F3)	Cell Cycle	0.033	0.02167
R-HSA-75035	Chk1/Chk2(Cds1) mediated inactivation of Cyclin B:Cdk1 complex	Cell Cycle	0.0315	0.02192
R-HSA-2980767	Activation of NIMA Kinases NEK9, NEK6, NEK7	Cell Cycle	0.0111	0.04511
R-HSA-176417	Phosphorylation of Emi1	Cell Cycle	0.0062	0.0614
R-HSA-69478	G2/M DNA replication checkpoint	Cell Cycle	0.0446	0.0421
R-HSA-445355	Smooth Muscle Contraction	Development	0.0124	0.00773
R-HSA-452723	Transcriptional regulation of pluripotent stem cells	Development	0.017	0.00848
R-HSA-8941326	RUNX2 regulates bone development	Development	0.0292	0.01754
R-HSA-190861	Gap junction assembly	Development	0.0394	0.00277
R-HSA-399954	Sema3A PAK dependent Axon repulsion	Development	0.0302	0.01754
R-HSA-201688	WNT mediated activation of DVL	Development	0.033	0.01973
R-HSA-111957	Cam-PDE 1 activation	Development	0.0126	0.01754
R-HSA-201451	Signaling by BMP	Development	0	0.01879
R-HSA-1181150	Signaling by NODAL	Development	3.00E-04	0.04385
R-HSA-179812	GRB2 events in EGFR signaling	Fibrosis	0.029	0.01214
R-HSA-212718	EGFR interacts with phospholipase C-gamma	Fibrosis	0.0165	0.01754
R-HSA-5638302	Signaling by Overexpressed Wild-Type EGFR in Cancer	Fibrosis	0.001	0.01973
R-HSA-5638303	Inhibition of Signaling by Overexpressed EGFR	Fibrosis	0.0019	0.01973
R-HSA-180336	SHC1 events in EGFR signaling	Fibrosis	0.0493	0.01127
R-HSA-445144	Signal transduction by L1	Fibrosis	0.0012	0.02506
R-HSA-1566977	Fibronectin matrix formation	Fibrosis	0.0422	0.01754
R-HSA-2173793	Transcriptional activity of SMAD2/SMAD3:SMAD4 heterotrimer	Fibrosis	2.00E-04	0.02033
R-HSA-2173796	SMAD2/SMAD3:SMAD4 heterotrimer regulates transcription	Fibrosis	0.0011	0.02138
R-HSA-2173789	TGF-beta receptor signaling activates SMADs	Fibrosis	0.0037	0.02059
R-HSA-2173795	Downregulation of SMAD2/3:SMAD4 transcriptional activity	Fibrosis	0.0016	0.02392
R-HSA-2173788	Downregulation of TGF-beta receptor signaling	Fibrosis	0.0423	0.01973
R-HSA-3304351	Signaling by TGF-beta Receptor Complex in Cancer	Fibrosis	3.00E-04	0.04605
R-HSA-3656534	Loss of Function of TGFBR1 in Cancer	Fibrosis	0.0033	0.03759
R-HSA-3304349	Loss of Function of SMAD2/3 in Cancer	Fibrosis	0.0013	0.04511

R-HSA-3304356	SMAD2/3 Phosphorylation Motif Mutants in Cancer	Fibrosis	0.0133	0.03508
R-HSA-3656532	TGFBR1 KD Mutants in Cancer	Fibrosis	0.0146	0.03508
R-HSA-1502540	Signaling by Activin	Fibrosis	0.0028	0.02024
R-HSA-9679191	Potential therapeutics for SARS	Immune	0.0244	0.00995
R-HSA-9664420	Killing mechanisms	Immune	0.0399	0.01315
R-HSA-5603041	IRAK4 deficiency (TLR2/4)	Immune	0.0024	0.01913
R-HSA-5602498	MyD88 deficiency (TLR2/4)	Immune	0.0019	0.02105
R-HSA-5210891	Uptake and function of anthrax toxins	Immune	0.0151	0.02024
R-HSA-9673324	WNT5:FZD7-mediated leishmania damping	Inflammation	0.0414	0.01315
R-HSA-1236973	Cross-presentation of particulate exogenous antigens (phagosomes)	Inflammation	0.0401	0.01315
R-HSA-444473	Formyl peptide receptors bind formyl peptides and many other ligands	Inflammation	0.0299	0.01315
R-HSA-70221	Glycogen breakdown (glycogenolysis)	Metabolism	0.0276	0.00657
R-HSA-8978934	Metabolism of cofactors	Metabolism	0.0095	0.00986
R-HSA-5620916	VxPx cargo-targeting to cilium	Metabolism	0.0167	0.00751
R-HSA-2022870	Chondroitin sulfate biosynthesis	Metabolism	0.0086	0.00526
R-HSA-1474151	Tetrahydrobiopterin (BH4) synthesis, recycling, salvage and regulation	Metabolism	0.0087	0.01435
R-HSA-264876	Insulin processing	Metabolism	0.0092	0.00751
R-HSA-418359	Reduction of cytosolic Ca++ levels	Metabolism	0.0048	0.01503
R-HSA-75205	Dissolution of Fibrin Clot	Metabolism	0.005	0.01214
R-HSA-425561	Sodium/Calcium exchangers	Metabolism	0.0112	0.01214
R-HSA-5576892	Phase 0 - rapid depolarisation	Others	0.023	0.00598
R-HSA-1296072	Voltage gated Potassium channels	Others	0.0496	0.00358
R-HSA-5576890	Phase 3 - rapid repolarisation	Others	0	0.03289
R-HSA-5576894	Phase 1 - inactivation of fast Na+ channels	Others	0.0062	0.01503
R-HSA-997272	Inhibition of voltage gated Ca2+ channels via Gbeta/gamma subunits	Others	0.0438	0.00362
R-HSA-5576893	Phase 2 - plateau phase	Others	0	0.01473
R-HSA-2514859	Inactivation, recovery and regulation of the phototransduction cascade	Others	0.0388	0.00318
R-HSA-1296041	Activation of G protein gated Potassium channels	Others	0.0448	0.00362
R-HSA-400253	Circadian Clock	Others	0.0026	0.01246
R-HSA-2514856	The phototransduction cascade	Others	0.05	0.00309
R-HSA-1296059	G protein gated Potassium channels	Others	0.045	0.00362
R-HSA-1368108	BMAL1:CLOCK,NPAS2 activates circadian gene expression	Others	0	0.03947
R-HSA-9620244	Long-term potentiation	Others	0.0121	0.02631
R-HSA-418360	Platelet calcium homeostasis	Wound-healing	0.0127	0.00779
R-HSA-9634638	Estrogen-dependent nuclear events downstream of ESR-membrane signaling	Wound-healing	0.0358	0.01144

R-HSA-5218920	VEGFR2 mediated vascular permeability	Wound-healing	0.0327	0.0127
R-HSA-3928664	Ephrin signaling	Wound-healing	0.0231	0.01662
R-HSA-203615	eNOS activation	Wound-healing	0.0197	0.01214
R-HSA-5675221	Negative regulation of MAPK pathway	Wound-healing	0	0.01591
R-HSA-450282	MAPK targets/ Nuclear events mediated by MAP kinases	Wound-healing	0.0125	0.01691
R-HSA-112409	RAF-independent MAPK1/3 activation	Wound-healing	0	0.02517
R-HSA-198753	ERK/MAPK targets	Wound-healing	0.005	0.02216
R-HSA-202670	ERKs are inactivated	Wound-healing	0	0.02834
R-HSA-9652817	Signaling by MAPK mutants	Wound-healing	1.00E-04	0.03007

ST_14	pathways_3405p_mRNA			
Term	Description	Functional category	P value	Density
R-HSA-6804116	TP53 Regulates Transcription of Genes Involved in G1 Cell Cycle Arrest	Apoptosis	0.0462	0.01785
R-HSA-9614085	FOXO-mediated transcription	Apoptosis	0.0172	0.01126
R-HSA-3899300	SUMOylation of transcription cofactors	Apoptosis	0.0457	0.00914
R-HSA-2032785	YAP1- and WWTR1 (TAZ)-stimulated gene expression	Apoptosis	0	0.02083
R-HSA-8849470	PTK6 Regulates Cell Cycle	Cell cycle	0.0287	0.03472
R-HSA-9659787	Aberrant regulation of mitotic G1/S transition in cancer due to RB1 defects	Cell cycle	0.0023	0.02696
R-HSA-9661069	Defective binding of RB1 mutants to E2F1,(E2F2, E2F3)	Cell cycle	0.0034	0.02696
R-HSA-69273	Cyclin A/B1/B2 associated events during G2/M transition	Cell cycle	0.0468	0.01333
R-HSA-9675126	Diseases of mitotic cell cycle	Cell cycle	0.0077	0.01388
R-HSA-9687139	Aberrant regulation of mitotic cell cycle due to RB1 defects	Cell cycle	0.0071	0.01388
R-HSA-69236	G1 Phase	Cell cycle	0.001	0.01861
R-HSA-69231	Cyclin D associated events in G1	Cell cycle	4.00E-04	0.01861
R-HSA-77075	RNA Pol II CTD phosphorylation and interaction with CE	Cell cycle	0.0448	0.00771
R-HSA-113418	Formation of the Early Elongation Complex	Cell cycle	4.00E-04	0.01388
R-HSA-8866910	TFAP2 (AP-2) family regulates transcription of growth factors and their receptors	Development	0.0442	0.00833
R-HSA-8864260	Transcriptional regulation by the AP-2 (TFAP2) family of transcription factors	Development	0.0238	0.009
R-HSA-5362517	Signaling by Retinoic Acid	Development	0	0.01259
R-HSA-8865999	MET activates PTPN11	Development	0.0115	0.025
R-HSA-2892247	POU5F1 (OCT4), SOX2, NANOG activate genes related to proliferation	Development	0.0464	0.00961
R-HSA-8951430	RUNX3 regulates WNT signaling	Development	0.0246	0.02604
R-HSA-4411364	Binding of TCF/LEF:CTNNB1 to target gene promoters	Development	0.0472	0.02083
R-HSA-193692	Regulated proteolysis of p75NTR	Development	0.0131	0.01515
R-HSA-8853884	Transcriptional Regulation by VENTX	Development	0.0427	0.00813
R-HSA-9665251	Resistance of ERBB2 KD mutants to lapatinib	Fibrosis	0.0289	0.02083
R-HSA-9665246	Resistance of ERBB2 KD mutants to neratinib	Fibrosis	0.0283	0.02083
R-HSA-9665230	Drug resistance in ERBB2 KD mutants	Fibrosis	0.0271	0.02083
R-HSA-9665247	Resistance of ERBB2 KD mutants to osimertinib	Fibrosis	0.0309	0.02083

R-HSA-9665250	Resistance of ERBB2 KD mutants to AEE788	Fibrosis	0.0294	0.02083
R-HSA-9665233	Resistance of ERBB2 KD mutants to trastuzumab	Fibrosis	0.032	0.02083
R-HSA-9652282	Drug-mediated inhibition of ERBB2 signaling	Fibrosis	0.0304	0.02083
R-HSA-9665249	Resistance of ERBB2 KD mutants to afatinib	Fibrosis	0.0279	0.02083
R-HSA-9665245	Resistance of ERBB2 KD mutants to tesevatinib	Fibrosis	0.0308	0.02083
R-HSA-9665737	Drug resistance in ERBB2 TMD/JMD mutants	Fibrosis	0.0287	0.02083
R-HSA-9665244	Resistance of ERBB2 KD mutants to sapitinib	Fibrosis	0.0266	0.02083
R-HSA-1306955	GRB7 events in ERBB2 signaling	Fibrosis	0.0324	0.01666
R-HSA-6785631	ERBB2 Regulates Cell Motility	Fibrosis	0.0343	0.00833
R-HSA-1963642	PI3K events in ERBB2 signaling	Fibrosis	0.0139	0.01041
R-HSA-9665686	Signaling by ERBB2 TMD/JMD mutants	Fibrosis	0.0195	0.00946
R-HSA-9664565	Signaling by ERBB2 KD Mutants	Fibrosis	0.032	0.00833
R-HSA-1227990	Signaling by ERBB2 in Cancer	Fibrosis	0.0336	0.00801
R-HSA-8863795	Downregulation of ERBB2 signaling	Fibrosis	0.0372	0.01005
R-HSA-8847993	ERBB2 Activates PTK6 Signaling	Fibrosis	0.024	0.00961
R-HSA-416550	Sema4D mediated inhibition of cell attachment and migration	Fibrosis	0.0263	0.01562
R-HSA-400685	Sema4D in semaphorin signaling	Fibrosis	0.0469	0.01111
R-HSA-2173796	SMAD2/SMAD3:SMAD4 heterotrimer regulates transcription	Fibrosis	0.0415	0.01562
R-HSA-2173793	Transcriptional activity of SMAD2/SMAD3:SMAD4 heterotrimer	Fibrosis	0.025	0.01325
R-HSA-4755510	SUMOylation of immune response proteins	Immune	8.00E-04	0.01893
R-HSA-5260271	Diseases of Immune System	Immune	0.0025	0.01215
R-HSA-5621575	CD209 (DC-SIGN) signaling	Immune	0.0032	0.01984
R-HSA-5603029	IkBA variant leads to EDA-ID	Immune	6.00E-04	0.03571
R-HSA-5607763	CLEC7A (Dectin-1) induces NFAT activation	Immune	0.0179	0.01388
R-HSA-2025928	Calcineurin activates NFAT	Immune	0.0156	0.01388
R-HSA-9616222	Transcriptional regulation of granulopoiesis	Immune	3.00E-04	0.02546
R-HSA-9660826	Purinergic signaling in leishmaniasis infection	Immune	0.0012	0.01215
R-HSA-209560	NF-kB is activated and signals survival	Immune	0.0249	0.02243
R-HSA-193639	p75NTR signals via NF-kB	Immune	0.0323	0.01822
R-HSA-1810476	RIP-mediated NFkB activation via ZBP1	Immune	0	0.0245
R-HSA-933542	TRAF6 mediated NF-kB activation	Immune	2.00E-04	0.01562
R-HSA-168927	TICAM1, RIP1-mediated IKK complex recruitment	Immune	0.0021	0.02083

R-HSA-937041	IKK complex recruitment mediated by RIP1	Immune	0.0026	0.01666
R-HSA-445989	TAK1 activates NFkB by phosphorylation and activation of IKKs complex	Immune	0.0029	0.01388
R-HSA-5660668	CLEC7A/inflammasome pathway	Inflammation	0.009	0.02777
R-HSA-448706	Interleukin-1 processing	Inflammation	4.00E-04	0.02604
R-HSA-844456	The NLRP3 inflammasome	Inflammation	3.00E-04	0.01944
R-HSA-622312	Inflammasomes	Inflammation	7.00E-04	0.01458
R-HSA-9664424	Cell recruitment (pro-inflammatory response)	Inflammation	3.00E-04	0.01215
R-HSA-4090294	SUMOylation of intracellular receptors	Inflammation	0	0.0216
R-HSA-5357956	TNFR1-induced NFkappaB signaling pathway	Inflammation	0.0062	0.01388
R-HSA-5357905	Regulation of TNFR1 signaling	Inflammation	0.0022	0.01388
R-HSA-75893	TNF signaling	Inflammation	0.005	0.01065
R-HSA-5626978	TNFR1-mediated ceramide production	Inflammation	0.0218	0.02083
R-HSA-5602358	Diseases associated with the TLR signaling cascade	Inflammation	0.002	0.01215
R-HSA-5576894	Phase 1 - inactivation of fast Na+ channels	Others	0.0076	0.0119
R-HSA-5576890	Phase 3 - rapid repolarisation	Others	0	0.02604
R-HSA-5576893	Phase 2 - plateau phase	Others	0	0.01333
R-HSA-1296041	Activation of G protein gated Potassium channels	Others	0	0.00862
R-HSA-997272	Inhibition of voltage gated Ca2+ channels via Gbeta/gamma subunits	Others	0	0.00862
R-HSA-1296059	G protein gated Potassium channels	Others	0	0.00862
R-HSA-1296065	Inwardly rectifying K+ channels	Others	0	0.00952
R-HSA-5576892	Phase 0 - rapid depolarisation	Others	4.00E-04	0.00757
R-HSA-1296072	Voltage gated Potassium channels	Others	0.0167	0.00378
R-HSA-1296025	ATP sensitive Potassium channels	Others	0.0016	0.02083
R-HSA-112308	Presynaptic depolarization and calcium channel opening	Others	0.0472	0.00694
R-HSA-977444	GABA B receptor activation	Others	1.00E-04	0.00581
R-HSA-991365	Activation of GABAB receptors	Others	2.00E-04	0.00581
R-HSA-9031525	NR1H2 & NR1H3 regulate gene expression to limit cholesterol uptake	Metabolism	6.00E-04	0.03333
R-HSA-9632974	NR1H2 & NR1H3 regulate gene expression linked to gluconeogenesis	Metabolism	0.005	0.025
R-HSA-9031528	NR1H2 & NR1H3 regulate gene expression linked to triglyceride lipolysis in adipose	Metabolism	7.00E-04	0.03333
R-HSA-193807	Synthesis of bile acids and bile salts via 27-hydroxycholesterol	Metabolism	0.0141	0.00833
R-HSA-159418	Recycling of bile acids and salts	Metabolism	0.01	0.00781

R-HSA-193368	Synthesis of bile acids and bile salts via 7alpha-hydroxycholesterol	Metabolism	0.0407	0.0052
R-HSA-70268	Pyruvate metabolism	Metabolism	0	0.0158
R-HSA-389661	Glyoxylate metabolism and glycine degradation	Metabolism	0.0067	0.00555
R-HSA-917977	Transferrin endocytosis and recycling	Metabolism	0.0137	0.00403
R-HSA-9029569	NR1H3 & NR1H2 regulate gene expression linked to cholesterol transport and efflux	Metabolism	0.0484	0.00788
R-HSA-2426168	Activation of gene expression by SREBF (SREBP)	Metabolism	0.0136	0.00595
R-HSA-1655829	Regulation of cholesterol biosynthesis by SREBP (SREBF)	Metabolism	0.021	0.00531
R-HSA-392154	Nitric oxide stimulates guanylate cyclase	Metabolism	0.0085	0.00892
R-HSA-446210	Synthesis of UDP-N-acetyl-glucosamine	Metabolism	0.0046	0.01041
R-HSA-9623433	NR1H2 & NR1H3 regulate gene expression to control bile acid homeostasis	Metabolism	0.0024	0.02314
R-HSA-9029558	NR1H2 & NR1H3 regulate gene expression linked to lipogenesis	Metabolism	0.0024	0.01851
R-HSA-204174	Regulation of pyruvate dehydrogenase (PDH) complex	Metabolism	0	0.02864
R-HSA-1368082	RORA activates gene expression	Others	0.0031	0.01851
R-HSA-400253	Circadian Clock	Others	0.0011	0.01315
R-HSA-5578768	Physiological factors	Others	1.00E-04	0.0243
R-HSA-167172	Transcription of the HIV genome	Virus	0.0084	0.01268
R-HSA-167200	Formation of HIV-1 elongation complex containing HIV-1 Tat	Virus	0.0076	0.01268
R-HSA-167169	HIV Transcription Elongation	Virus	0.0079	0.01268
R-HSA-167246	Tat-mediated elongation of the HIV-1 transcript	Virus	0.0067	0.01268
R-HSA-3134963	DEx/H-box helicases activate type I IFN and inflammatory cytokines production	Virus	5.00E-04	0.04166
R-HSA-1606322	ZBP1(DAI) mediated induction of type I IFNs	Virus	0	0.01984
R-HSA-5689896	Ovarian tumor domain proteases	Virus	0.037	0.00986
R-HSA-933541	TRAF6 mediated IRF7 activation	Virus	0.0459	0.00718

ST_15	functionalCategoriesAssignmentMap	
Term	Description	Functional category
R-HSA-1912408	Pre-NOTCH Transcription and Translation	Apoptosis
R-HSA-1912422	Pre-NOTCH Expression and Processing	Apoptosis
R-HSA-1980143	Signaling by NOTCH1	Apoptosis
R-HSA-2032785	YAP1- and WWTR1 (TAZ)-stimulated gene expression	Apoptosis
R-HSA-2122947	NOTCH1 Intracellular Domain Regulates Transcription	Apoptosis
R-HSA-2428933	SHC-related events triggered by IGF1R	Apoptosis
R-HSA-2660825	Signaling by NOTCH1 t(7;9)(NOTCH1:M1580_K2555) Translocation Mutant	Apoptosis
R-HSA-2660826	Constitutive Signaling by NOTCH1 t(7;9)(NOTCH1:M1580_K2555) Translocation Mutant	Apoptosis
R-HSA-3899300	SUMOylation of transcription cofactors	Apoptosis
R-HSA-389948	PD-1 signaling	Apoptosis
R-HSA-5083630	Defective LFNG causes SCDO3	Apoptosis
R-HSA-5673000	RAF activation	Apoptosis
R-HSA-6804116	TP53 Regulates Transcription of Genes Involved in G1 Cell Cycle Arrest	Apoptosis
R-HSA-6804757	Regulation of TP53 Degradation	Apoptosis
R-HSA-6806003	Regulation of TP53 Expression and Degradation	Apoptosis
R-HSA-8941856	RUNX3 regulates Notch signaling	Apoptosis
R-HSA-8951936	RUNX3 regulates p14-ARF	Apoptosis
R-HSA-8952158	RUNX3 regulates BCL2L11 (BIM) transcription	Apoptosis
R-HSA-9012852	Signaling by NOTCH3	Apoptosis
R-HSA-9013508	NOTCH3 Intracellular Domain Regulates Transcription	Apoptosis
R-HSA-9013694	Signaling by NOTCH4	Apoptosis
R-HSA-9013694	Signaling by NOTCH4	Apoptosis
R-HSA-9013695	NOTCH4 Intracellular Domain Regulates Transcription	Apoptosis
R-HSA-9013695	NOTCH4 Intracellular Domain Regulates Transcription	Apoptosis
R-HSA-9614085	FOXO-mediated transcription	Apoptosis
R-HSA-9614085	FOXO-mediated transcription	Apoptosis
R-HSA-9614657	FOXO-mediated transcription of cell death genes	Apoptosis
R-HSA-9617629	Regulation of FOXO transcriptional activity by acetylation	Apoptosis
R-HSA-113418	Formation of the Early Elongation Complex	Cell cycle

R-HSA-141405	Inhibition of the proteolytic activity of APC/C required for the onset of anaphase by mitotic spindle checkpoint components	Cell Cycle
R-HSA-141430	Inactivation of APC/C via direct inhibition of the APC/C complex	Cell Cycle
R-HSA-174048	APC/C:Cdc20 mediated degradation of Cyclin B	Cell Cycle
R-HSA-176407	Conversion from APC/C:Cdc20 to APC/C:Cdh1 in late anaphase	Cell Cycle
R-HSA-176412	Phosphorylation of the APC/C	Cell Cycle
R-HSA-176417	Phosphorylation of Emi1	Cell Cycle
R-HSA-179409	APC-Cdc20 mediated degradation of Nek2A	Cell Cycle
R-HSA-2980767	Activation of NIMA Kinases NEK9, NEK6, NEK7	Cell Cycle
R-HSA-5576892	Phase 0 - rapid depolarisation	Cell Cycle
R-HSA-5626467	RHO GTPases activate IQGAPs	Cell Cycle
R-HSA-6791312	TP53 Regulates Transcription of Cell Cycle Genes	Cell Cycle
R-HSA-6804116	TP53 Regulates Transcription of Genes Involved in G1 Cell Cycle Arrest	Cell Cycle
R-HSA-69231	Cyclin D associated events in G1	Cell Cycle
R-HSA-69231	Cyclin D associated events in G1	Cell cycle
R-HSA-69236	G1 Phase	Cell cycle
R-HSA-69273	Cyclin A/B1/B2 associated events during G2/M transition	Cell Cycle
R-HSA-69273	Cyclin A/B1/B2 associated events during G2/M transition	Cell cycle
R-HSA-69478	G2/M DNA replication checkpoint	Cell Cycle
R-HSA-75035	Chk1/Chk2(Cds1) mediated inactivation of Cyclin B:Cdk1 complex	Cell Cycle
R-HSA-77075	RNA Pol II CTD phosphorylation and interaction with CE	Cell cycle
R-HSA-8849470	PTK6 Regulates Cell Cycle	Cell cycle
R-HSA-9617828	FOXO-mediated transcription of cell cycle genes	Cell Cycle
R-HSA-9659787	Aberrant regulation of mitotic G1/S transition in cancer due to RB1 defects	Cell Cycle
R-HSA-9659787	Aberrant regulation of mitotic G1/S transition in cancer due to RB1 defects	Cell cycle
R-HSA-9661069	Defective binding of RB1 mutants to E2F1,(E2F2, E2F3)	Cell Cycle
R-HSA-9661069	Defective binding of RB1 mutants to E2F1,(E2F2, E2F3)	Cell cycle
R-HSA-9675126	Diseases of mitotic cell cycle	Cell Cycle
R-HSA-9675126	Diseases of mitotic cell cycle	Cell cycle

R-HSA-9687136	Aberrant regulation of mitotic exit in cancer due to RB1 defects	Cell Cycle
R-HSA-9687139	Aberrant regulation of mitotic cell cycle due to RB1 defects	Cell Cycle
R-HSA-9687139	Aberrant regulation of mitotic cell cycle due to RB1 defects	Cell cycle
R-HSA-111957	Cam-PDE 1 activation	Development
R-HSA-162791	Attachment of GPI anchor to uPAR	Development
R-HSA-167044	Signalling to RAS	Development
R-HSA-190861	Gap junction assembly	Development
R-HSA-193692	Regulated proteolysis of p75NTR	Development
R-HSA-201451	Signaling by BMP	Development
R-HSA-201688	WNT mediated activation of DVL	Development
R-HSA-2892247	POU5F1 (OCT4), SOX2, NANOG activate genes related to proliferation	Development
R-HSA-2892247	POU5F1 (OCT4), SOX2, NANOG activate genes related to proliferation	Development
R-HSA-399954	Sema3A PAK dependent Axon repulsion	Development
R-HSA-4090294	SUMOylation of intracellular receptors	Development
R-HSA-4411364	Binding of TCF/LEF:CTNNB1 to target gene promoters	Development
R-HSA-445144	Signal transduction by L1	Development
R-HSA-445355	Smooth Muscle Contraction	Development
R-HSA-452723	Transcriptional regulation of pluripotent stem cells	Development
R-HSA-452723	Transcriptional regulation of pluripotent stem cells	Development
R-HSA-525793	Myogenesis	Development
R-HSA-5362517	Signaling by Retinoic Acid	Development
R-HSA-5362517	Signaling by Retinoic Acid	Development
R-HSA-5627123	RHO GTPases activate PAKs	Development
R-HSA-8853659	RET signaling	Development
R-HSA-8853884	Transcriptional Regulation by VENTX	Development
R-HSA-8864260	Transcriptional regulation by the AP-2 (TFAP2) family of transcription factors	Development
R-HSA-8864260	Transcriptional regulation by the AP-2 (TFAP2) family of transcription factors	Development
R-HSA-8865999	MET activates PTPN11	Development
R-HSA-8866907	Activation of the TFAP2 (AP-2) family of transcription factors	Development
R-HSA-8866910	TFAP2 (AP-2) family regulates transcription of growth factors and their receptors	Development
R-HSA-8940973	RUNX2 regulates osteoblast differentiation	Development
R-HSA-8941326	RUNX2 regulates bone development	Development

R-HSA-8951430	RUNX3 regulates WNT signaling	Development
R-HSA-9006115	Signaling by NTRK2 (TRKB)	Development
R-HSA-9634638	Estrogen-dependent nuclear events downstream of ESR-membrane signaling	Development
R-HSA-1181150	Signaling by NODAL	Fibrosis
R-HSA-1226099	Signaling by FGFR in disease	Fibrosis
R-HSA-1227986	Signaling by ERBB2	Fibrosis
R-HSA-1227990	Signaling by ERBB2 in Cancer	Fibrosis
R-HSA-1227990	Signaling by ERBB2 in Cancer	Fibrosis
R-HSA-1236382	Constitutive Signaling by Ligand-Responsive EGFR Cancer Variants	Fibrosis
R-HSA-1236394	Signaling by ERBB4	Fibrosis
R-HSA-1250347	SHC1 events in ERBB4 signaling	Fibrosis
R-HSA-1306955	GRB7 events in ERBB2 signaling	Fibrosis
R-HSA-1433557	Signaling by SCF-KIT	Fibrosis
R-HSA-1433559	Regulation of KIT signaling	Fibrosis
R-HSA-1502540	Signaling by Activin	Fibrosis
R-HSA-1566977	Fibronectin matrix formation	Fibrosis
R-HSA-1643713	Signaling by EGFR in Cancer	Fibrosis
R-HSA-177929	Signaling by EGFR	Fibrosis
R-HSA-179812	GRB2 events in EGFR signaling	Fibrosis
R-HSA-180292	GAB1 signalosome	Fibrosis
R-HSA-180336	SHC1 events in EGFR signaling	Fibrosis
R-HSA-1839117	Signaling by cytosolic FGFR1 fusion mutants	Fibrosis
R-HSA-1839124	FGFR1 mutant receptor activation	Fibrosis
R-HSA-1963642	PI3K events in ERBB2 signaling	Fibrosis
R-HSA-212718	EGFR interacts with phospholipase C-gamma	Fibrosis
R-HSA-2173788	Downregulation of TGF-beta receptor signaling	Fibrosis
R-HSA-2173789	TGF-beta receptor signaling activates SMADs	Fibrosis
R-HSA-2173793	Transcriptional activity of SMAD2/SMAD3:SMAD4 heterotrimer	Fibrosis
R-HSA-2173793	Transcriptional activity of SMAD2/SMAD3:SMAD4 heterotrimer	Fibrosis
R-HSA-2173795	Downregulation of SMAD2/3:SMAD4 transcriptional activity	Fibrosis
R-HSA-2173796	SMAD2/SMAD3:SMAD4 heterotrimer regulates transcription	Fibrosis
R-HSA-2173796	SMAD2/SMAD3:SMAD4 heterotrimer regulates transcription	Fibrosis
R-HSA-3304349	Loss of Function of SMAD2/3 in Cancer	Fibrosis
R-HSA-3304351	Signaling by TGF-beta Receptor Complex in Cancer	Fibrosis

R-HSA-3304356	SMAD2/3 Phosphorylation Motif Mutants in Cancer	Fibrosis
R-HSA-3656532	TGFBR1 KD Mutants in Cancer	Fibrosis
R-HSA-3656534	Loss of Function of TGFBR1 in Cancer	Fibrosis
R-HSA-400685	Sema4D in semaphorin signaling	Fibrosis
R-HSA-416550	Sema4D mediated inhibition of cell attachment and migration	Fibrosis
R-HSA-5637810	Constitutive Signaling by EGFRvIII	Fibrosis
R-HSA-5637812	Signaling by EGFRvIII in Cancer	Fibrosis
R-HSA-5637815	Signaling by Ligand-Responsive EGFR Variants in Cancer	Fibrosis
R-HSA-5638302	Signaling by Overexpressed Wild-Type EGFR in Cancer	Fibrosis
R-HSA-5638303	Inhibition of Signaling by Overexpressed EGFR	Fibrosis
R-HSA-5654687	Downstream signaling of activated FGFR1	Fibrosis
R-HSA-5654688	SHC-mediated cascade:FGFR1	Fibrosis
R-HSA-5654689	PI-3K cascade:FGFR1	Fibrosis
R-HSA-5654693	FRS-mediated FGFR1 signaling	Fibrosis
R-HSA-5654696	Downstream signaling of activated FGFR2	Fibrosis
R-HSA-5654704	SHC-mediated cascade:FGFR3	Fibrosis
R-HSA-5654708	Downstream signaling of activated FGFR3	Fibrosis
R-HSA-5654716	Downstream signaling of activated FGFR4	Fibrosis
R-HSA-5654719	SHC-mediated cascade:FGFR4	Fibrosis
R-HSA-5654736	Signaling by FGFR1	Fibrosis
R-HSA-5655253	Signaling by FGFR2 in disease	Fibrosis
R-HSA-5655291	Signaling by FGFR4 in disease	Fibrosis
R-HSA-5655302	Signaling by FGFR1 in disease	Fibrosis
R-HSA-5655332	Signaling by FGFR3 in disease	Fibrosis
R-HSA-6785631	ERBB2 Regulates Cell Motility	Fibrosis
R-HSA-8847993	ERBB2 Activates PTK6 Signaling	Fibrosis
R-HSA-8851805	MET activates RAS signaling	Fibrosis
R-HSA-8853334	Signaling by FGFR3 fusions in cancer	Fibrosis
R-HSA-8853338	Signaling by FGFR3 point mutants in cancer	Fibrosis
R-HSA-8863795	Downregulation of ERBB2 signaling	Fibrosis
R-HSA-912631	Regulation of signaling by CBL	Fibrosis
R-HSA-9634285	Constitutive Signaling by Overexpressed ERBB2	Fibrosis
R-HSA-9652282	Drug-mediated inhibition of ERBB2 signaling	Fibrosis
R-HSA-9664565	Signaling by ERBB2 KD Mutants	Fibrosis
R-HSA-9664565	Signaling by ERBB2 KD Mutants	Fibrosis

R-HSA-9665230	Drug resistance in ERBB2 KD mutants	Fibrosis
R-HSA-9665233	Resistance of ERBB2 KD mutants to trastuzumab	Fibrosis
R-HSA-9665244	Resistance of ERBB2 KD mutants to sapitinib	Fibrosis
R-HSA-9665245	Resistance of ERBB2 KD mutants to tesevatinib	Fibrosis
R-HSA-9665246	Resistance of ERBB2 KD mutants to neratinib	Fibrosis
R-HSA-9665247	Resistance of ERBB2 KD mutants to osimertinib	Fibrosis
R-HSA-9665249	Resistance of ERBB2 KD mutants to afatinib	Fibrosis
R-HSA-9665250	Resistance of ERBB2 KD mutants to AEE788	Fibrosis
R-HSA-9665251	Resistance of ERBB2 KD mutants to lapatinib	Fibrosis
R-HSA-9665348	Signaling by ERBB2 ECD mutants	Fibrosis
R-HSA-9665686	Signaling by ERBB2 TMD/JMD mutants	Fibrosis
R-HSA-9665686	Signaling by ERBB2 TMD/JMD mutants	Fibrosis
R-HSA-9665737	Drug resistance in ERBB2 TMD/JMD mutants	Fibrosis
R-HSA-9669938	Signaling by KIT in disease	Fibrosis
R-HSA-9670439	Signaling by phosphorylated juxtamembrane, extracellular and kinase domain KIT mutants	Fibrosis
R-HSA-1236973	Cross-presentation of particulate exogenous antigens (phagosomes)	Immune
R-HSA-1368082	RORA activates gene expression	Immune
R-HSA-167169	HIV Transcription Elongation	Immune
R-HSA-167172	Transcription of the HIV genome	Immune
R-HSA-167200	Formation of HIV-1 elongation complex containing HIV-1 Tat	Immune
R-HSA-167246	Tat-mediated elongation of the HIV-1 transcript	Immune
R-HSA-202427	Phosphorylation of CD3 and TCR zeta chains	Immune
R-HSA-2025928	Calcineurin activates NFAT	Immune
R-HSA-210990	PECAM1 interactions	Immune
R-HSA-2172127	DAP12 interactions	Immune
R-HSA-2424491	DAP12 signaling	Immune
R-HSA-444473	Formyl peptide receptors bind formyl peptides and many other ligands	Immune
R-HSA-4755510	SUMOylation of immune response proteins	Immune
R-HSA-5210891	Uptake and function of anthrax toxins	Immune
R-HSA-5260271	Diseases of Immune System	Immune

R-HSA-5602498	MyD88 deficiency (TLR2/4)	Immune
R-HSA-5603029	IkBA variant leads to EDA-ID	Immune
R-HSA-5603041	IRAK4 deficiency (TLR2/4)	Immune
R-HSA-5607763	CLEC7A (Dectin-1) induces NFAT activation	Immune
R-HSA-5621480	Dectin-2 family	Immune
R-HSA-5621575	CD209 (DC-SIGN) signaling	Immune
R-HSA-5621575	CD209 (DC-SIGN) signaling	Immune
R-HSA-8939246	RUNX1 regulates transcription of genes involved in differentiation of myeloid cells	Immune
R-HSA-918233	TRAF3-dependent IRF activation pathway	Immune
R-HSA-9616222	Transcriptional regulation of granulopoiesis	Immune
R-HSA-9664420	Killing mechanisms	Immune
R-HSA-9673324	WNT5:FZD7-mediated leishmania damping	Immune
R-HSA-9679191	Potential therapeutics for SARS	Immune
R-HSA-1059683	Interleukin-6 signaling	Inflammation
R-HSA-1234158	Regulation of gene expression by Hypoxia-inducible Factor	Inflammation
R-HSA-1810476	RIP-mediated NFkB activation via ZBP1	Inflammation
R-HSA-193639	p75NTR signals via NF-kB	Inflammation
R-HSA-209560	NF-kB is activated and signals survival	Inflammation
R-HSA-350054	Notch-HLH transcription pathway	Inflammation
R-HSA-4090294	SUMOylation of intracellular receptors	Inflammation
R-HSA-448706	Interleukin-1 processing	Inflammation
R-HSA-448706	Interleukin-1 processing	Inflammation
R-HSA-451927	Interleukin-2 family signaling	Inflammation
R-HSA-512988	Interleukin-3, Interleukin-5 and GM-CSF signaling	Inflammation
R-HSA-5357905	Regulation of TNFR1 signaling	Inflammation
R-HSA-5357956	TNFR1-induced NFkappaB signaling pathway	Inflammation
R-HSA-5626978	TNFR1-mediated ceramide production	Inflammation
R-HSA-5660668	CLEC7A/inflammasome pathway	Inflammation
R-HSA-622312	Inflammasomes	Inflammation
R-HSA-622312	Inflammasomes	Inflammation
R-HSA-6783589	Interleukin-6 family signaling	Inflammation
R-HSA-75893	TNF signaling	Inflammation
R-HSA-844456	The NLRP3 inflammasome	Inflammation
R-HSA-844456	The NLRP3 inflammasome	Inflammation
R-HSA-8854691	Interleukin-20 family signaling	Inflammation
R-HSA-8983432	Interleukin-15 signaling	Inflammation
R-HSA-8984722	Interleukin-35 Signalling	Inflammation
R-HSA-8985947	Interleukin-9 signaling	Inflammation

R-HSA-9008059	Interleukin-37 signaling	Inflammation
R-HSA-9020558	Interleukin-2 signaling	Inflammation
R-HSA-9020933	Interleukin-23 signaling	Inflammation
R-HSA-9020956	Interleukin-27 signaling	Inflammation
R-HSA-9020958	Interleukin-21 signaling	Inflammation
R-HSA-912526	Interleukin receptor SHC signaling	Inflammation
R-HSA-933542	TRAF6 mediated NF-kB activation	Inflammation
R-HSA-9660826	Purinergic signaling in leishmaniasis infection	Inflammation
R-HSA-9660826	Purinergic signaling in leishmaniasis infection	Inflammation
R-HSA-9664424	Cell recruitment (pro-inflammatory response)	Inflammation
R-HSA-9664424	Cell recruitment (pro-inflammatory response)	Inflammation
R-HSA-112308	Presynaptic depolarization and calcium channel opening	Metabolism
R-HSA-1296025	ATP sensitive Potassium channels	Metabolism
R-HSA-1296041	Activation of G protein gated Potassium channels	Metabolism
R-HSA-1296041	Activation of G protein gated Potassium channels	Metabolism
R-HSA-1296059	G protein gated Potassium channels	Metabolism
R-HSA-1296065	Inwardly rectifying K+ channels	Metabolism
R-HSA-1296072	Voltage gated Potassium channels	Metabolism
R-HSA-1296072	Voltage gated Potassium channels	Metabolism
R-HSA-1474151	Tetrahydrobiopterin (BH4) synthesis, recycling, salvage and regulation	Metabolism
R-HSA-1474151	Tetrahydrobiopterin (BH4) synthesis, recycling, salvage and regulation	Metabolism
R-HSA-159418	Recycling of bile acids and salts	Metabolism
R-HSA-159418	Recycling of bile acids and salts	Metabolism
R-HSA-162710	Synthesis of glycosylphosphatidylinositol (GPI)	Metabolism
R-HSA-1655829	Regulation of cholesterol biosynthesis by SREBP (SREBF)	Metabolism
R-HSA-1655829	Regulation of cholesterol biosynthesis by SREBP (SREBF)	Metabolism
R-HSA-192105	Synthesis of bile acids and bile salts	Metabolism
R-HSA-193368	Synthesis of bile acids and bile salts via 7alpha-hydroxycholesterol	Metabolism
R-HSA-193368	Synthesis of bile acids and bile salts via 7alpha-hydroxycholesterol	Metabolism
R-HSA-193807	Synthesis of bile acids and bile salts via 27-hydroxycholesterol	Metabolism
R-HSA-193807	Synthesis of bile acids and bile salts via 27-hydroxycholesterol	Metabolism

R-HSA-194068	Bile acid and bile salt Metabolism	Metabolism
R-HSA-196791	Vitamin D (calciferol) Metabolism	Metabolism
R-HSA-200425	Carnitine Metabolism	Metabolism
R-HSA-2022870	Chondroitin sulfate biosynthesis	Metabolism
R-HSA-204174	Regulation of pyruvate dehydrogenase (PDH) complex	Metabolism
R-HSA-204174	Regulation of pyruvate dehydrogenase (PDH) complex	Metabolism
R-HSA-211976	Endogenous sterols	Metabolism
R-HSA-2426168	Activation of gene expression by SREBF (SREBP)	Metabolism
R-HSA-2426168	Activation of gene expression by SREBF (SREBP)	Metabolism
R-HSA-2514859	Inactivation, recovery and regulation of the phototransduction cascade	Metabolism
R-HSA-264876	Insulin processing	Metabolism
R-HSA-389661	Glyoxylate metabolism and glycine degradation	Metabolism
R-HSA-392154	Nitric oxide stimulates guanylate cyclase	Metabolism
R-HSA-418359	Reduction of cytosolic Ca++ levels	Metabolism
R-HSA-418360	Platelet calcium homeostasis	Metabolism
R-HSA-425561	Sodium/Calcium exchangers	Metabolism
R-HSA-433137	Sodium-coupled sulphate, di- and tri-carboxylate transporters	Metabolism
R-HSA-446210	Synthesis of UDP-N-acetyl-glucosamine	Metabolism
R-HSA-5576890	Phase 3 - rapid repolarisation	Metabolism
R-HSA-5576890	Phase 3 - rapid repolarisation	Metabolism
R-HSA-5576892	Phase 0 - rapid depolarisation	Metabolism
R-HSA-5576893	Phase 2 - plateau phase	Metabolism
R-HSA-5576893	Phase 2 - plateau phase	Metabolism
R-HSA-5576894	Phase 1 - inactivation of fast Na+ channels	Metabolism
R-HSA-5576894	Phase 1 - inactivation of fast Na+ channels	Metabolism
R-HSA-5620916	VxPx cargo-targeting to cilium	Metabolism
R-HSA-70221	Glycogen breakdown (glycogenolysis)	Metabolism
R-HSA-70268	Pyruvate Metabolism	Metabolism
R-HSA-70268	Pyruvate metabolism	Metabolism
R-HSA-75205	Dissolution of Fibrin Clot	Metabolism
R-HSA-8978934	Metabolism of cofactors	Metabolism
R-HSA-9024446	NR1H2 and NR1H3-mediated signaling	Metabolism
R-HSA-9029558	NR1H2 & NR1H3 regulate gene expression linked to lipogenesis	Metabolism
R-HSA-9029558	NR1H2 & NR1H3 regulate gene expression linked to lipogenesis	Metabolism

R-HSA-9029569	NR1H3 & NR1H2 regulate gene expression linked to cholesterol transport and efflux	Metabolism
R-HSA-9029569	NR1H3 & NR1H2 regulate gene expression linked to cholesterol transport and efflux	Metabolism
R-HSA-9031525	NR1H2 & NR1H3 regulate gene expression to limit cholesterol uptake	Metabolism
R-HSA-9031525	NR1H2 & NR1H3 regulate gene expression to limit cholesterol uptake	Metabolism
R-HSA-9031528	NR1H2 & NR1H3 regulate gene expression linked to triglyceride lipolysis in adipose	Metabolism
R-HSA-9031528	NR1H2 & NR1H3 regulate gene expression linked to triglyceride lipolysis in adipose	Metabolism
R-HSA-917977	Transferrin endocytosis and recycling	Metabolism
R-HSA-9623433	NR1H2 & NR1H3 regulate gene expression to control bile acid homeostasis	Metabolism
R-HSA-9623433	NR1H2 & NR1H3 regulate gene expression to control bile acid homeostasis	Metabolism
R-HSA-9632974	NR1H2 & NR1H3 regulate gene expression linked to gluconeogenesis	Metabolism
R-HSA-9632974	NR1H2 & NR1H3 regulate gene expression linked to gluconeogenesis	Metabolism
R-HSA-9634600	Regulation of glycolysis by fructose 2,6-bisphosphate Metabolism	Metabolism
R-HSA-977444	GABA B receptor activation	Metabolism
R-HSA-991365	Activation of GABAB receptors	Metabolism
R-HSA-997272	Inhibition of voltage gated Ca2+ channels via Gbeta/gamma subunits	Metabolism
R-HSA-997272	Inhibition of voltage gated Ca2+ channels via Gbeta/gamma subunits	Metabolism
R-HSA-1296059	G protein gated Potassium channels	Others
R-HSA-1368082	RORA activates gene expression	Others
R-HSA-1368108	BMAL1:CLOCK,NPAS2 activates circadian gene expression	Others
R-HSA-1368108	BMAL1:CLOCK,NPAS2 activates circadian gene expression	Others
R-HSA-210500	Glutamate Neurotransmitter Release Cycle	Others
R-HSA-2514856	The phototransduction cascade	Others
R-HSA-3371511	HSF1 activation	Others
R-HSA-3371568	Attenuation phase	Others
R-HSA-3371571	HSF1-dependent transactivation	Others

R-HSA-400253	Circadian Clock	Others
R-HSA-400253	Circadian Clock	Others
R-HSA-400253	Circadian Clock	Others
R-HSA-5578768	Physiological factors	Others
R-HSA-9620244	Long-term potentiation	Others
R-HSA-1606322	ZBP1(DAI) mediated induction of type I IFNs	Virus
R-HSA-168927	TICAM1, RIP1-mediated IKK complex recruitment	Virus
R-HSA-3134963	DEx/H-box helicases activate type I IFN and inflammatory cytokines production	Virus
R-HSA-3134973	LRR FLII-interacting protein 1 (LRRFIP1) activates type I IFN production	Virus
R-HSA-445989	TAK1 activates NFkB by phosphorylation and activation of IKKs complex	Virus
R-HSA-5602358	Diseases associated with the TLR signaling cascade	Virus
R-HSA-5689896	Ovarian tumor domain proteases	Virus
R-HSA-8937144	Aryl hydrocarbon receptor signalling	Virus
R-HSA-912694	Regulation of IFNA signaling	Virus
R-HSA-933541	TRAF6 mediated IRF7 activation	Virus
R-HSA-937041	IKK complex recruitment mediated by RIP1	Virus
R-HSA-112409	RAF-independent MAPK1/3 activation	Wound-healing
R-HSA-114604	GPVI-mediated activation cascade	Wound-healing
R-HSA-198753	ERK/MAPK targets	Wound-healing
R-HSA-202670	ERKs are inactivated	Wound-healing
R-HSA-203615	eNOS activation	Wound-healing
R-HSA-210993	Tie2 Signaling	Wound-healing
R-HSA-354192	Integrin signaling	Wound-healing
R-HSA-3928662	EPHB-mediated forward signaling	Wound-healing
R-HSA-3928664	Ephrin signaling	Wound-healing
R-HSA-3928664	Ephrin signaling	Wound-healing
R-HSA-3928665	EPH-ephrin mediated repulsion of cells	Wound-healing
R-HSA-450282	MAPK targets/ Nuclear events mediated by MAP kinases	Wound-healing
R-HSA-5218920	VEGFR2 mediated vascular permeability	Wound-healing
R-HSA-5675221	Negative regulation of MAPK pathway	Wound-healing
R-HSA-76009	Platelet Aggregation (Plug Formation)	Wound-healing
R-HSA-8849468	PTK6 Regulates Proteins Involved in RNA Processing	Wound-healing
R-HSA-8849471	PTK6 Regulates RHO GTPases, RAS GTPase and MAP kinases	Wound-healing
R-HSA-8849473	PTK6 Expression	Wound-healing
R-HSA-8849474	PTK6 Activates STAT3	Wound-healing

R-HSA-9006335	Signaling by Erythropoietin	Wound-healing
R-HSA-9027284	Erythropoietin activates RAS	Wound-healing
R-HSA-9031628	NGF-stimulated transcription	Wound-healing
R-HSA-9652817	Signaling by MAPK mutants	Wound-healing
R-HSA-9671555	Signaling by PDGFR in disease	Wound-healing
R-HSA-9673767	Signaling by PDGFRA transmembrane, juxtamembrane and kinase domain mutants	Wound-healing
R-HSA-9673770	Signaling by PDGFRA extracellular domain mutants	Wound-healing

ST_16	DE_mRNA_in_core_modules_within_THP1/LX2								
Index	Gene	NC vs. thp-1		NC vs. thp-1		NC vs. lx-2		NC vs. lx-2	
	symbol	miR20a_mimics		miR340_mimics		miR20a_mimics		miR20a_mimics	
		FC*	padj	FC*	padj	FC*	padj	FC*	padj
1	ADAMTS1	-2.16	7.15E-269	-1.95	1.99E-244	-0.57	4.88E-03	-0.67	4.01E-11
2	AHRR	-	-	-	-	-	-	-	-
3	ARHGAP29	4.03	1.54E-03	3	4.87E-02	-0.31	6.94E-02	-1	1.08E-31
4	ARHGEF10	-	-	-	-	-	-	-	-
5	ARL5B	1.92	0.00E+00	1.68	7.15E-227	-0.11	6.50E-01	-0.94	4.48E-13
6	BTG3	0.63	1.21E-22	0.6	1.01E-21	-0.52	1.72E-03	-0.49	3.23E-04
7	C14orf119	-	-	0.22	5.24E-04	-0.16	4.11E-01	-0.14	1.82E-01
8	CAV1	-1.56	1.98E-34	-1.33	9.60E-25	-0.1	6.26E-01	-0.46	1.10E-06
9	CCNT1	-0.15	6.02E-03	-0.17	3.86E-03	-0.66	4.98E-05	-1.15	1.11E-25
10	CCSER1	0.21	3.66E-02	0.27	2.23E-01	0.81	3.05E-05	1.02	7.74E-28
11	CFL2	-	-	-0.08	3.94E-01	-0.07	8.11E-01	-0.31	2.83E-02
12	CHST7	0.6	4.86E-09	0.64	1.61E-11	0.59	1.78E-03	0.82	2.42E-13
13	CXCL8	2.47	2.79E-71	2.28	4.33E-250	1.3	8.22E-04	0.97	1.05E-02
14	DBF4	-0.5	1.21E-13	-0.6	6.23E-16	-0.11	5.40E-01	-0.48	3.33E-06
15	DEPDC1	-1.4	2.70E-28	-1.18	1.13E-23	-0.24	3.13E-01	-0.74	9.61E-11
16	DUSP8	2.77	2.65E-111	2.68	2.66E-103	0.62	8.32E-04	1.04	1.43E-17
17	EREG	-	-	2.07	4.08E-02	0.27	-	0.53	7.86E-01
18	FEM1C	1.08	1.15E-84	1.05	2.47E-97	-0.93	3.73E-08	-1.17	3.48E-19
19	FZD7	0.73	1.05E-30	0.78	4.12E-28	-0.09	6.69E-01	-0.02	9.04E-01
20	GFPT2	-	-	0.49	2.14E-01	0.2	2.61E-01	0.26	1.57E-02
21	GJA3	0.26	1.52E-04	0.24	3.30E-03	0.03	9.22E-01	-0.36	6.70E-02
22	HIF1A	1.2	3.28E-04	0.88	7.11E-03	-0.13	7.87E-01	-1.02	1.55E-02
23	KCNJ2	-	-	0	9.90E-01	-0.44	-	-1.31	3.61E-01
24	KLHL15	0.54	1.24E-12	0.38	1.12E-08	-0.52	4.96E-03	-1.02	1.15E-17
25	MASTL	1.81	5.72E-156	1.74	1.36E-155	-0.47	3.18E-03	-0.85	1.05E-13
26	MET	1.28	7.67E-36	1.26	9.53E-36	-0.5	6.99E-04	-0.74	2.32E-15
27	MIXL1	-	-	-0.28	9.15E-01	0.43	-	1.05	3.81E-01
28	NCR3LG1	-0.53	1.78E-18	-0.49	3.57E-15	-0.83	1.38E-04	-0.93	3.85E-09
29	NEXMIF	0.43	3.13E-02	-0.42	7.03E-02	0.49	-	-0.8	-
30	NR1D2	0.55	8.64E-10	0.45	2.82E-05	-0.45	8.57E-03	-0.87	2.90E-11
31	NR4A2	0.8	1.17E-18	0.78	7.52E-18	0.31	4.89E-01	0.42	3.04E-01
32	NR4A3	-	-	-	-	-	-	-	-
33	NRARP	-0.82	1.87E-03	-0.61	1.65E-02	0.78	8.06E-02	1.31	3.29E-04
34	OSM	-0.89	7.88E-09	-0.58	3.74E-05	-0.38	-	1.92	2.66E-01
35	PANX2	-	-	0.27	3.83E-02	0.47	4.29E-02	1.25	1.60E-15
36	PDE4D	-0.18	3.01E-02	-0.12	1.73E-01	-0.08	7.17E-01	-0.79	4.67E-11
37	PDK4	-	-	-	-	-	-	-	-
38	PER1	-	-	-0.03	7.86E-01	0.32	4.86E-02	0.79	4.21E-20
39	PFKFB3	0.34	1.55E-11	0.35	1.50E-14	-0.07	6.92E-01	0.13	1.98E-01
40	PGAP1	2.03	5.01E-149	1.82	1.55E-94	-0.36	7.88E-02	-0.74	6.97E-09
41	PIK3R1	1.4	2.11E-235	1.31	2.18E-194	-0.58	4.04E-03	-1.07	5.25E-16
42	PKMYT1	-1.42	2.47E-40	-1.18	1.01E-36	0.18	2.93E-01	0.83	1.16E-19
43	PRLR	0.5	2.81E-03	0.27	1.66E-01	-0.57	2.34E-01	-0.27	6.26E-01
44	RBM34	-	-	0.1	8.56E-01	-0.22	-	-1.13	3.10E-01
45	REL	0.5	1.21E-17	0.36	6.81E-07	-0.53	3.37E-02	-0.98	9.53E-07
46	RLIM	0.1	4.93E-02	0.01	9.01E-01	-0.62	4.75E-05	-1.08	6.96E-20

47	RNF152	-0.84	1.30E-05	-0.77	9.99E-05	0.29	2.52E-01	-0.05	8.78E-01
48	RORB	3.12	2.23E-83	2.86	8.37E-62	0.18	-	-3.34	-
49	SERTAD2	0.18	7.91E-05	0.18	2.43E-04	-0.38	1.53E-02	-0.46	4.14E-06
50	SIAH1	-	-	-0.13	3.77E-01	-0.48	1.30E-01	-0.9	5.98E-03
51	SIK1	0.94	2.80E-02	0.8	8.11E-02	0	9.94E-01	0.44	1.11E-03
52	SKOR1	-	-	0.22	7.64E-01	0.39	4.81E-01	0.65	2.05E-01
53	SLC1A2	2.89	8.77E-03	1.96	1.98E-01	-0.81	-	0.65	7.04E-01
54	SMAD6	0.19	6.68E-03	0.22	1.48E-03	-0.04	8.46E-01	0.2	6.76E-02
55	SOX5	1.31	7.47E-85	1.13	3.36E-78	0.34	4.44E-01	-0.7	1.79E-01
56	SRGAP1	-1.3	1.12E-44	-1.13	1.77E-34	-0.47	2.45E-03	-0.74	3.80E-09
57	STBD1	-	-	-	-	-	-	-	-
58	STEAP4	-	-	3.24	-	-	-	-	-
59	SYNM	-0.5	1.15E-09	-0.33	8.13E-05	-0.39	2.90E-02	-0.6	5.52E-05
60	TENM1	-	-	-0.81	1.60E-01	-0.34	3.43E-01	-0.74	3.15E-02
61	TIPARP	1.15	6.60E-99	1	8.94E-85	-0.5	2.36E-03	-0.86	4.42E-17
62	TMEM170B	-	-	0.02	8.49E-01	-0.05	7.93E-01	-0.3	4.66E-02
63	TMEM255A	0.74	8.14E-65	0.67	5.88E-47	-0.26	1.16E-01	-0.38	1.27E-04
64	TNFAIP3	1.66	4.53E-255	1.59	1.41E-269	0.05	8.35E-01	-0.02	8.85E-01
65	TNFSF11	-	-	0.01	9.87E-01	1.29	-	3.08	2.67E-01
66	UBE2C	-0.78	1.86E-19	-0.53	2.83E-08	0.4	2.26E-02	0.89	7.47E-28
67	YES1	-0.2	7.29E-04	-0.39	2.00E-07	-0.12	5.11E-01	-0.51	1.56E-06
68	YOD1	-	-	-	-	-0.26	2.59E-01	-0.72	3.07E-05
69	ZBTB10	-0.65	3.33E-13	-0.63	2.13E-14	-	-	-	-
70	ZBTB21	0.36	3.90E-08	0.27	1.05E-04	-0.5	1.39E-03	-1.06	2.04E-17
71	ZBTB43	1.63	0.00E+00	1.52	3.42E-232	-0.55	1.57E-03	-0.74	3.66E-10
72	ZNF331	-0.66	1.10E-07	-0.81	3.20E-09	-0.47	1.34E-02	-0.69	9.30E-08
73	ZNF484	-	-	-	-	-	-	-	-
74	ZNF703	-0.35	2.97E-05	-0.16	4.16E-02	0.29	6.26E-02	0.82	2.55E-24
75	ZNF805	0.29	2.28E-03	0.18	8.82E-02	-0.56	6.85E-04	-1.13	1.91E-11
